# Bifunctional Aziridines from Photochemically Generated FSO_2_NH_2_ for SuFEx Diversification of Alkenes

**DOI:** 10.1002/anie.202521575

**Published:** 2025-11-20

**Authors:** Avinash Choudhury, Varun Prabhakar, Zakary B. Newman, Quentin Michaudel

**Affiliations:** ^1^ Department of Chemistry Texas A & M University College Station Texas 77843 USA; ^2^ Department of Materials Science and Engineering Texas A & M University College Station Texas 77843 USA

**Keywords:** Aziridination, Rh‐catalyzed nitrene transfer, Scaffold diversification, SuFEx click chemistry

## Abstract

Sulfonyl fluorides have emerged as ubiquitous reactive motifs in fields ranging from medicinal chemistry to materials science, with applications as chemical warheads and as clickable units via Sulfur(VI) Fluoride Exchange (SuFEx). However, methods for introducing sulfamoyl fluorides, the nitrogen‐containing congeners, into functional‐group rich scaffolds remain scarce. Herein, we report the synthesis of fluorosulfonyl aziridines from FSO_2_NH_2_ as a versatile and modular platform for alkene diversification. A safe and practical synthesis of FSO_2_NH_2_ was optimized via photodegradation of fluorosulfonyl azide (FSO_2_N_3_) in solution, enabling the development of a Rh‐catalyzed aziridination with a broad alkene scope that merges the innate reactivity of the strained heterocycle with the modularity of SuFEx click chemistry. The unprecedented bifunctional electrophile was exploited in selective reactions with a wide array of nucleophiles under mild conditions, delivering sulfamoyl fluorides as well as S(VI)‐containing linkages of pharmaceutical relevance, including sulfamides and sulfamate esters.

## Graphical abstract

Photochemically generated FSO_2_NH_2_ serves as a safe, practical nitrene precursor for Rh‐catalyzed aziridination of alkenes, granting access to previously inaccessible fluorosulfonyl aziridines. These bifunctional electrophiles merge aziridine ring strain with SuFEx reactivity to provide a modular platform toward aliphatic sulfamoyl fluoride derivatives and ultimately S(VI)‐containing molecules including sulfamides and sulfamate esters.

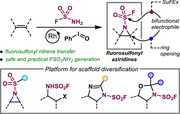

Sulfonyl fluorides (RSO_2_F), broadly defined, have become a prevalent scaffold in drug discovery,^[^ ^1^ ^]^ as well as a modular synthetic platform for the preparation of molecules ranging from fine chemicals to polymers^[^ ^2^, ^3^, ^4^, ^5^, ^6^, ^7^, ^8^ ^]^ via Sulfur(VI) Fluoride Exchange (SuFEx) click chemistry.^[^ ^9^, ^10^ ^]^ The enhanced stability and on‐demand reactivity of S(VI)─F bond, compared to that of other sulfur─halide bonds, have enabled their applications as protease inhibitors and chemical probes,^[^ ^11^, ^12^ ^]^ as well as structurally diverse SuFEx hubs.^[^ ^9^, ^13^ ^]^ Among these, sulfamoyl fluorides (NR_1_R_2_SO_2_F) hold promise as bioactive compounds^[^ ^14^, ^15^, ^16^ ^]^ (Scheme 1a) and as valuable building blocks in polymer synthesis.^[^ ^17^, ^18^, ^19^ ^]^ However, approaches to access such compounds remain scarce, especially for aliphatic substrates. Direct functionalization with SO_2_F_2_ and a suitable base is limited to secondary amines, including four‐membered or larger nitrogen heterocycles (Scheme 1a).^[^ ^20^ ^]^ Sulfamoyl fluorides derived from primary amines are unstable under basic conditions and therefore require the use of bespoke reagents, such as SuFEx‐IT^[^ ^21^ ^]^ or AISF.^[^ ^22^ ^]^ A handful of fluorosulfamoylation methods via C─N bond formation have been reported, but these either rely on the use of F_2_/N_2_,^[^ ^23^, ^24^ ^]^ or are limited to styrenic derivatives and N‐methylated sulfamoyl fluorides.^[^ ^25^, ^26^, ^27^ ^]^ With the goal of transforming ubiquitous alkenes into modular SuFEx handles, we report a practical synthesis of previously inaccessible fluorosulfonyl aziridines, as versatile branching points toward sulfamoyl fluoride derivatives and ultimately S(VI)‐containing molecules including sulfamides and sulfamate esters (Scheme 1b)

**Scheme 1:**
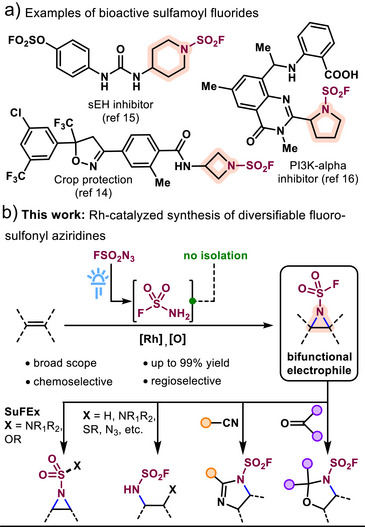
Access to fluorosulfonyl aziridines: a missing scaffold with high synthetic potential.

Alkene aziridination has emerged as a promising strategy to enhance the potency of complex drug scaffold such as epothilones.^[^ ^28^, ^29^, ^30^ ^]^ However, the multi‐step sequence for their installation and derivatization via sulfonylation represents a significant synthetic bottleneck in a drug‐discovery campaign. Although a variety of elegant aziridinations,^[^ ^31^, ^32^ ^]^ including photochemical^[^ ^33^, ^34^, ^35^, ^36^, ^37^, ^38^ ^]^ and electrochemical processes,^[^ ^39^, ^40^, ^41^ ^]^ have recently been reported, few enable derivatization at nitrogen.^[^ ^42^, ^43^, ^44^ ^]^ The advent of metal nitrenoids has democratized the synthesis of sulfonylated aziridines, but the prevalent tosyl (Ts) or nosyl (Ns) groups are dead ends for divergent synthesis and require removal through typically harsh reductive conditions.^[^ ^45^ ^]^ Importantly, while efficient methods have been developed for the direct synthesis of N─H aziridines,^[^ ^46^, ^47^, ^48^ ^]^ fluorosulfonylation of these motifs with SO_2_F_2_ or SuFEx‐IT has proven challenging due to concomitant ring opening^[^ ^49^, ^50^ ^]^ likely owing to the low basicity of aziridines (pKa ∼8 in water).^[^ ^31^ ^]^

We hypothesized that appending a fluorosulfonyl group to a nitrene precursor would both stabilize the nitrene‐transfer reagent and introduce the desired fluorosulfonyl handle into a bifunctional aziridine. To the best of our knowledge, the only reported characterization of a triplet fluorosulfonyl nitrene (FSO_2_N) was made in the gas phase through flash vacuum pyrolysis at 1000 K of fluorosulfonyl azide FSO_2_N_3_.^[^ ^51^ ^]^ Given the documented shock sensitivity of chlorosulfonyl azide (ClSO_2_N_3_)^[^ ^52^ ^]^ and the absence of safety data for its fluorinated analog, we sought to avoid isolating neat FSO_2_N_3_. Instead, FSO_2_N_3_ (**2a**) was generated in situ from SuFEx‐IT and NaN_3_ as an MTBE solution following phase separation (Figure 1a).^[^ ^53^ ^]^ Direct or photosensitized excitation of **2a** to generate the nitrene in the presence of alkenes, however, led to complex mixtures of products with trace amounts or very low yields of desired aziridines (Tables S7 and S8). We surmised that the presence of hydridic bonds in MTBE may explain the formation of undesired products via hydrogen‐atom abstraction (HAT), unfortunately, the screening of solvents to circumvent this pitfall was limited by the preparation of **2a** and its isolation via simple liquid–liquid extraction. For instance, the use of NaN_3_ precluded chlorinated solvents—typical in photochemical triplet nitrene generation—due to the risk of forming explosive species such as bisazidomethane.^[^ ^54^ ^]^ This prompted us to explore the feasibility of accessing a fluorosulfonyl metal nitrenoid from sulfamoyl fluoride **1** as a tamer alternative to FSO_2_N. Unfortunately, although **1** is commercially available, its cost is prohibitive.^[^ ^55^ ^]^ Additionally, known synthetic routes to **1** require reagents that are corrosive, moisture‐sensitive and generate highly toxic byproducts such as hydrogen fluoride (Figure 1a).^[^ ^56^, ^57^, ^58^ ^]^ Motivated by practicality and safety concerns, we explored conditions for the transformation of **2a** into **1**. Typical reducing agents including triphenylphosphine, LiAlH_4_ and NaBH_4_ afforded low yields and poor reproducibility, potentially due to trace amount of water remaining in the MTBE solution. To our delight, building on our initial experiments, photodegradation of **2a** in MTBE under UV irradiation (254 nm) led to the exclusive formation of **1**, obtained in consistent yields following aqueous workup (Figure 1a). The reduction is hypothesized to proceed via HAT from MTBE by the short‐lived triplet nitrene. A similar reduction has been observed as an undesired pathway in the photochemical C─H amination of ethers with nonaflyl azide, which was posited to follow a radical chain mechanism initiated by the corresponding triplet nitrene.^[^ ^59^ ^]^ The main advantage of this two‐step route toward **1** lies in its operational simplicity, involving two open‐to‐air reactions requiring only liquid‐liquid extractions for purification, hereby avoiding the isolation of potentially toxic or explosive intermediates.

**Figure 1:**
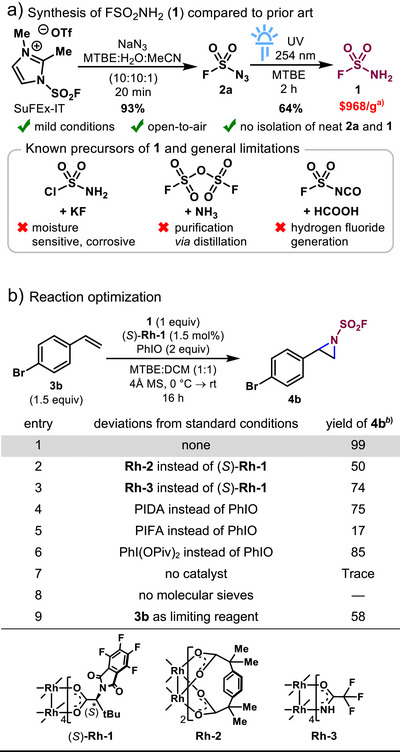
a) UV photodegradation route to 1 from 2a and earlier precursors, with general limitations. b) Reaction optimization. ^a)^Ref. 55 ^b)^Determined via ^1^H NMR using 1,2,4,5‐tetrabromobenzene as an internal standard.

The suitability of **1** as an aziridination reagent was subsequently evaluated using *p*‐bromostyrene (**3b**) as a convenient test substrate with low volatility (Tables S1–S3). While initial catalysts including Ag(I), Cu(I), Cu(II) delivered low yields of desired product, (*S*)‐**Rh‐1** initially reported by Hashimoto for enantioselective C─H amidation^[^ ^60^, ^61^ ^]^ and recently by Dauban^[^ ^62^ ^]^ for enantioselective aziridination, was found to provide quantitative yield of **4b** in the presence of iodosobenzene (PhIO), 4 Å molecular sieves (MS) and a slight excess of **3b** relative to **1** (1.5 equiv) in a 1:1 mixture of DCM and MTBE (Figure 1b, Entry 1). Interestingly, replacing (*S*)**‐Rh‐1** by Du Bois's Rh_2_(esp)_2_ (**Rh‐2**)^[^ ^63^ ^]^ or Rh_2_(OAc)_4_ (Entry 2 and Table S1) led to a dramatic decrease of the yield, while Rh_2_(tfacam)_4_ (**Rh‐3**)^[^ ^64^, ^65^ ^]^ delivered **4b** in 74% yield (Entry 3). Notably, although an asymmetric transformation was not initially targeted, the inclusion of a chiral dirhodium complex in the screening pool led to the optimal conditions. Similar trends were independently observed by Davies and Fokin in carbene‐based Rh‐catalyzed transformations, where chiral catalysts provided higher yields than their achiral counterparts.^[^ ^66^, ^67^, ^68^ ^]^ Although the underlying reasons for the higher aziridination efficiency observed with (*S*)‐**Rh‐1** compared to **Rh‐3** remain unclear, this trend was consistent across multiple substrates (Table S4) and may stem from favorable F–π interactions between the NSO_2_F group and the phthalimide ligands within the hydrophobic pocket.^[^ ^62^, ^69^ ^]^ Oxidants such as PIFA, PIDA, and PhI(OPiv)_2_ combined with MgO delivered **4b** in lower yields (Entries 4–6 and Table S2). Control reactions were performed without catalyst, which only led to trace amount of aziridine **4b** (Entry 7). The addition of MS was critical to remove adventitious water from the stock solution of **1** and avoid quenching of the Rh‐nitrenoid (Entry 8).^[^ ^70^, ^71^ ^]^ Finally, using **3b** as the limiting reagent lowered the aziridine yield to 58% (Entry 9), which is in line with other aziridination reactions.^[^ ^72^ ^]^

With the optimized conditions in hand, we explored the scope of the aziridination (Figure 2). Styrene was transformed in 99% yield as assessed by ^1^H NMR. The resulting fluorosulfonyl aziridine was found to be unstable on silica gel but treatment with thiophenol led to isolable thioether **6** **g** (Supporting Information). Derivatives containing electron‐withdrawing substituents afforded silica‐stable aziridines **4b**–**4d** in excellent yields (93%–99%). In contrast, para‐substituted electron‐rich styrenes (*p*‐Me, *p*‐OMe, and *p*‐Ph) delivered complex mixture of products under the standard conditions likely through ring‐opening of the aziridine.^[^ ^73^ ^]^ Disubstituted and trisubstituted activated alkenes, including β‐methylstyrenes, furnished aziridines **4e**–**4f** in 82%–98% yields. Notably, aziridination of (*E*)‐ and (*Z*)‐β‐methyl styrene was found to be stereospecific, delivering *trans*‐**4e** and *cis*‐**4e**, respectively. Cyclic and terminal aliphatic alkenes were also competent substrates, yielding aziridines **4g**–**4l** in 45%–98% yields. Aziridine **4j** was obtained as a single diastereomer, whose structure was confirmed by X‐ray crystallography. Impressively, tetrasubstituted alkene **3 m** also reacted efficiently, delivering **4 m** in 96% yield. A series of natural products and their derivatives were also tested to determine the functional group tolerance and site‐selectivity of this process. While geraniol containing a free hydroxyl group was not tolerated, protected analogs underwent selective aziridination to afford **4n**–**4p** in 42%–98% yields. The reactions were highly regioselective, favoring more electron‐rich alkene in all three cases.

**Figure 2:**
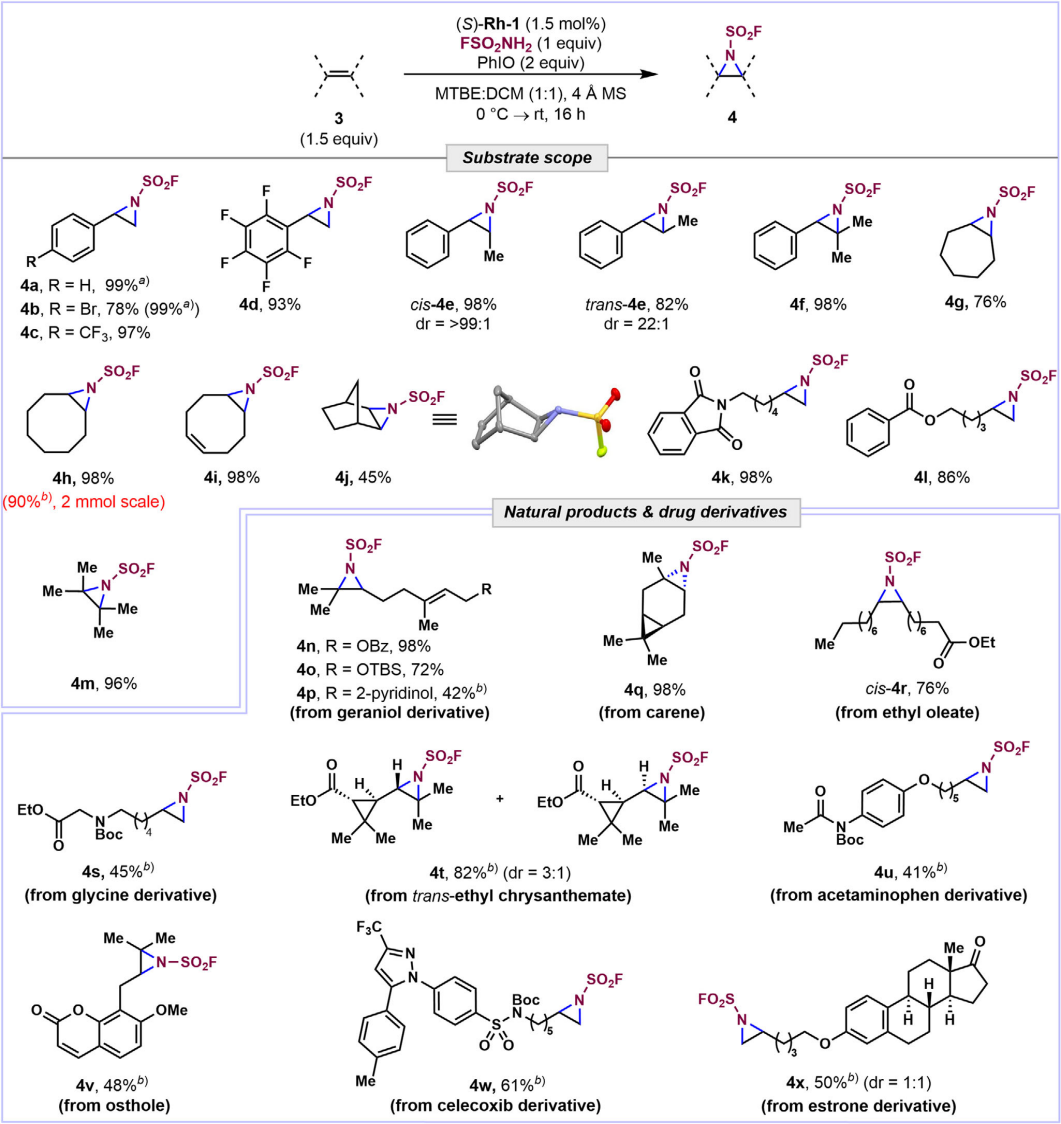
Substrate scope of aziridination. ^a)^Determined by ^1^H NMR using 1,2,4,5‐tetrabromobenzene as internal standard. ^b)^2 equiv of alkene were used.

Reactions with 3‐carene and ethyl oleate delivered aziridines **4q** and *cis*‐**4r** with excellent diastereoselectivity (>99:1) and glycine derivative afforded aziridine **4s** (45%). Similarly, monoterpenoid *trans*‐ethyl chrysanthemate gave product **4t** (82%) as a mixture of two diastereomers (3:1) with the *trans*‐isomer being the major product. Bioactive molecules and drug derivatives, including acetaminophen (**4u**), osthole (**4v**), celecoxib (**4w**), and estrone (**4x**), were all successfully functionalized, highlighting the potential of this aziridination protocol as a late‐stage functionalization method. The reaction was tolerant of various functional groups, including halogens, esters, amides, silyl ethers, trifluoromethyl groups, cyclopropanes (**4t**), and heterocycles such as pyridine (**4p**), pyrazole (**4w**), and coumarin (**4v**).

Having established a robust method for the aziridination of alkenes via metal‐catalyzed fluorosulfonyl nitrene transfer, we turned our attention toward evaluating the synthetic potential of the resulting fluorosulfonyl aziridines. In contrast to conventional sulfonyl protecting groups (e.g., tosyl, nosyl), the sulfamoyl fluoride moiety offers unique reactivity as a branching point, enabling downstream derivatization via SuFEx chemistry. Following a modified protocol developed by Ball and am Ende using Ca(NTf_2_)_2_ as a Lewis acid catalyst,^[^ ^74^ ^]^ several aziridines underwent efficient SuFEx ligation with amine and phenol nucleophiles (Figure 3a). Disubstituted (**4h**) and trisubstituted (**4f**) aziridines underwent SuFEx with various phenol derivatives, including bioactive estrone and ezetimibe, to give products **5a–5c** in good yields. While coupling of **4j** with morpholine gave the expected SuFEx product (**5d**), tetrasubstituted aziridine **4m** afforded cyclic sulfamide **5e** upon treatment with benzylamine. **5e** was likely formed via a tandem sequence involving SuFEx followed by intramolecular aziridine opening, underscoring the bifunctional electrophilicity of these scaffolds. In contrast, terminal aziridines led to intractable mixtures of compounds, likely due to competitive reactivity between the two electrophilic centers. Fortunately, ring‐opening of such aziridines could be effected in high yields with diverse nucleophiles, thereby providing access to primary sulfamoyl fluorides (Figure 3b). For example, treatment of **4b**, *cis*‐**4e** and **4l** or **4a** with aniline, trimethylsilyl azide and thiophenol, respectively, delivered the corresponding β‐functionalized sulfamoyl fluorides (**6a–6c** and **6g**) in good yields. While a regioselective ring‐opening was observed in all cases, aziridines **4a**, **4b,** and *cis*‐**4e** underwent attack at the more substituted benzylic position, whereas the terminal aliphatic aziridine **4l** opened exclusively at the less hindered site. This suggests that an S_N_1‐ or S_N_2‐type mechanism can dominate depending on the aziridine substrate.^[^ ^75^ ^]^ Aziridines *cis*‐**4e** and **4b** were also found to be amenable to [3 + 2] cycloaddition^[^ ^76^ ^]^ with 2‐methoxy benzonitrile and cyclohexanone via C─N bond cleavage in the presence of BF_3_•OEt_2_ (Figure 3c). This expedient sequence furnished sulfamoyl fluoride‐functionalized imidazoline **6d** and oxazolidine **6e**, two SuFEx hubs for the diversification of medicinal‐type scaffolds. To further demonstrate the synthetic versatility of this class of bifunctional electrophile, SO_2_F‐analogs of phenethylamine class drugs, amphetamine (**7a**) and phentermine (**7b**), were prepared through hydrogenation of aziridines *cis*‐**4e** and **4f**, respectively, using Pd/C and H_2_ (1 atm). Subsequent SuFEx coupling with amine containing drugs (e.g., antazoline and mexiletine) afforded the corresponding conjugates **8a** and **8b** in excellent yields (Figure 3d). Finally, the aziridine–SO_2_F platform was applied toward the synthesis of two‐pronged click hubs^[^ ^77^ ^]^ bearing orthogonal SuFEx and Copper‐catalyzed Azide‐Alkyne Cycloaddition (CuAAC) handles (Figure 3e). Ring‐opening of aziridine **4h** with sodium azide yielded **6f** bearing a mono‐substituted sulfamoyl fluoride. Under basic conditions putatively promoting the formation of an azasulfene intermediate,^[^ ^78^ ^]^ addition of benzylamine resulted in the formation of sulfamide **8c** in 84% yield. Standard CuAAC conditions afforded **9** in 86% yield, highlighting the efficient merger of click chemistry enabled by the atom‐economic fluorosulfonyl aziridine motif and the potential of these compounds for late‐stage functionalization from bioconjugation to material synthesis. The broad range of motifs accessible from bifunctional fluorosulfonyl aziridines through sequential and chemoselective nucleophilic additions highlight the synthetic potential of this platform.

**Figure 3:**
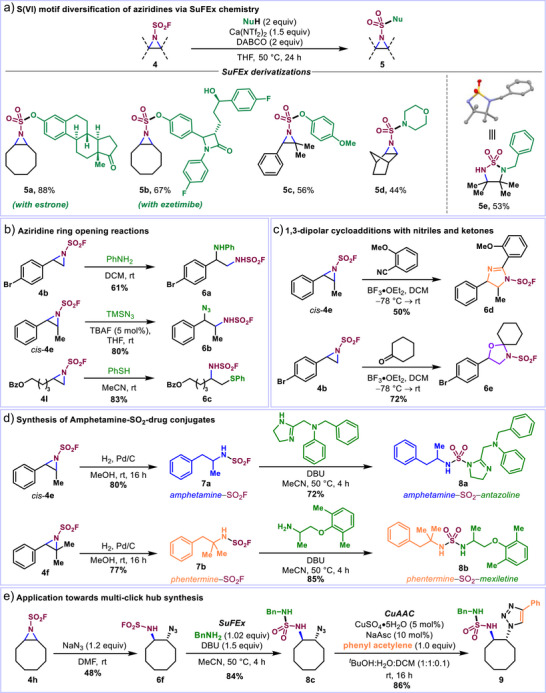
a)–e) Synthetic applications of fluorosulfonyl aziridines.

While our initial catalyst screen focused on optimizing the efficiency of the reaction, the identification of chiral (*S*)‐**Rh‐1** as the best catalyst prompted us to launch a preliminary investigation of the enantioselectivity of this process with a variety of alkene substrates. Notably, trisubstituted styrenic derivative **4f** afforded a high enantiomeric excess (ee) of 90% for the (*R*)‐enantiomer (Figure 4), in line with prior reports.^[^ ^60^, ^78^ ^]^ The absolute configuration of the major enantiomer was established via X‐ray diffraction of sulfamide **5f** (Figure S11) resulting from SuFEx of **4f** with benzylamine. While lower ee values were measured for monosubstituted aziridine **4d** (54%), disubstituted aziridines *cis*‐**4e** and *trans*‐**4e** (30% and 52%, respectively) and trisubstituted aliphatic aziridine **4n** (20%), these results lay the groundwork for the asymmetric installation of enantioenriched fluorosulfonyl aziridines and thereby sulfamoyl fluoride derivatives, which are inaccessible using current radical fluorosulfamoylation methods. ^[^ ^25^, ^26^, ^27^ ^]^

**Figure 4:**
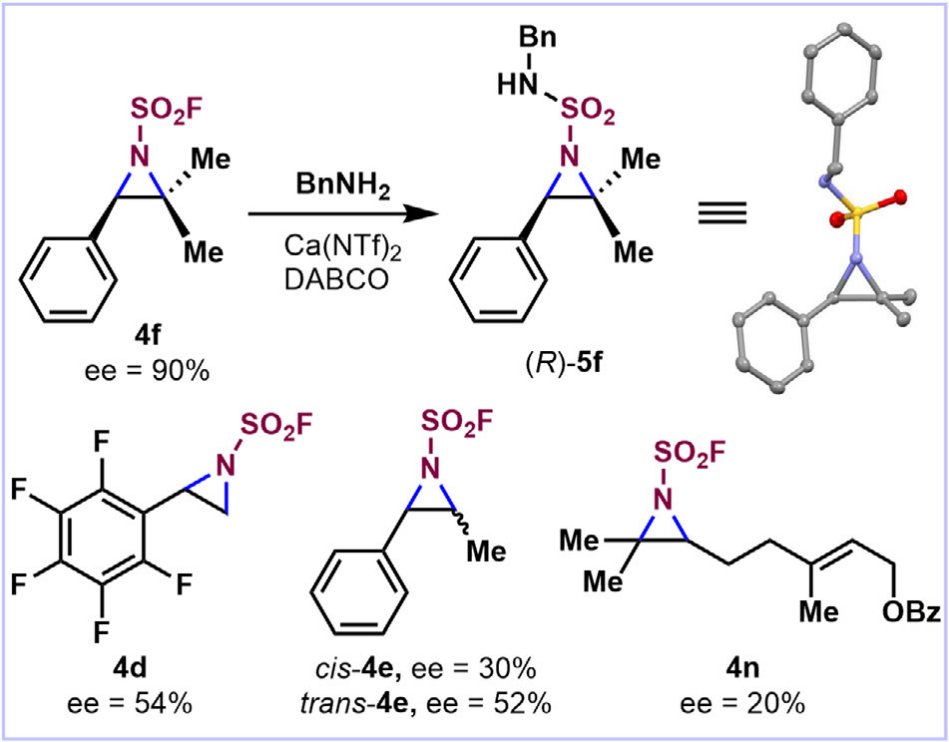
Initial assessment of the enantioselectivity provided by (*S*)‐Rh‐1.

In conclusion, we have developed a general approach to fluorosulfonyl aziridines that provides a modular platform for alkene functionalization, capitalizing on the bifunctional nature of this unique electrophile. The orthogonal reactivity of these intermediates was demonstrated with a broad range of nucleophiles and reaction conditions, granting access to diverse sulfamoyl fluoride scaffolds as well as S(VI) linkages of high relevance to medicinal chemistry, including sulfamides and sulfamate esters. This chemistry also enabled the design of a dual CuAAC/SuFEx click hub for the rapid assembly of complex molecules. Furthermore, the measured ee values for selected substrates using chiral (*S*)‐**Rh‐1** indicate strong potential for the asymmetric installation of these motifs. Finally, the establishment of a safe and practical route to FSO_2_NH_2_ (**1**), a largely underexplored reagent, should open new avenues for a range of transformations.

## Supporting Information

The authors have cited additional references within the Supporting Information.^[^ ^79^, ^80^, ^81^, ^82^, ^83^, ^84^, ^85^, ^86^ ^]^

## Conflict of Interests

The authors declare no conflict of interest.

## Supporting information



Supporting Information

Supporting Information

## Data Availability

The data that support the findings of this study are available in the supplementary material of this article.
