# Supplementary material for: Bifunctional Aziridines from Photochemically Generated FSO2NH2 for SuFEx Diversification of Alkenes
Source: Angew Chem Int Ed Engl. 2025 Nov 20;65(2):e21575. doi: 10.1002/anie.202521575 (PMC12790346; doi:10.1002/anie.202521575)
Supplement: Supplementary file 1 — Supporting Information [file ANIE-65-e21575-s002.pdf]

Supporting Information

Bifunctional Aziridines from Photochemically Generated FSO_2NH_2 for SuFEx Diversification of Alkenes

Avinash Choudhury, Varun Prabhakar, Zakary B. Newman, and Quentin Michaudel*

Department of Chemistry, Texas A&M University, College Station, Texas 77843, United States

Department of Materials Science & Engineering, Texas A&M University, College Station, Texas 77843, United States

*Corresponding Author: quentin.michaudel@chem.tamu.edu

General reagent information…S2

General analytical information…S2

Experimental procedures…S3

Synthesis of FSO_2N_3 (**2a**) and FSO_2NH_2 (**1**)…S3

Preparation of starting materials and alkene substrates…S5

Aziridination reaction optimization…S10

Aziridination: **General Procedure A**…S11

Synthesis and characterization of aziridines… S12

Synthetic applications…S23

Enantioselectivity studies…S33

Photodegradation and reduction of FSO_2N_3 (**2a**) …S36

Attempts for direct and photosensitized aziridination using FSO_2N_3 (**2a**) …S36

X-ray diffraction data…S38

NMR spectra…S53

References…S125

General reagent information

All reactions were performed without any precaution for moisture and oxygen unless otherwise stated. Reagents were purchased at the highest commercial quality and used without further purification, unless otherwise stated. All starting materials were purchased at the highest commercial quality and used without further purification unless otherwise stated. 1- (fluorosulfonyl)-2,3-dimethyl-1H-imidazol-3-ium triflate (SuFEx-IT), alkene substrates (**3k**, **3l**, **3o**, **3x**), calcium bistriflimide ($Ca(NTf_2)_2$), and iodosobenzene (PhIO) were synthesized following previously reported procedures.[1–6] The reactions were heated using an oil bath unless otherwise stated. Yields refer to chromatographically and spectroscopically (^1H NMR) homogeneous material, unless otherwise stated. Reactions were monitored by thin layer chromatography (TLC) carried out on 250 µm SiliCycle SilicaPlate™ silica plates (F254), using UV light as the visualizing agent and an acidic solution of p-anisaldehyde and heat or ninhydrin and heat or phosphomolybdic acid and heat as developing agents. Flash silica gel chromatography was performed using SiliCycle SilicaFlash® Irregular Silica Gel (60 Å, particle size 40–63 µm). The UV reactions were carried out using Rayonet RPR-2537A (λ = 254 nm), Rayonet RPR 3500A (λ = 350 nm) lamps and an 18 mL quartz glass tube (Rayonet RQV-7). The visible light photochemical reactions were carried out using Hepatochem EvoluChemTM PhotoRedOx Box Duo device and irradiated with two EvoluChemTM P303-30-1 LEDs (30 W, λ_{max} = 450 nm) with a cooling fan. All visible light photochemical reactions were performed in borosilicate glass.

General analytical information

^1H Nuclear magnetic resonance (NMR) spectra were recorded on two Bruker Avance NEO 400 MHz and a Bruker Avance 500 MHz; ^{13}C spectra were recorded on a Bruker Avance 500 MHz and a Bruker Avance NEO 400 MHz; ^{19}F spectra were recorded using a Bruker Avance NEO 400 MHz instrument. All ^1H and ^{13}C spectra were calibrated using residual deuterated solvent as an internal reference ($CDCl_3$ @ 7.26 ppm ^1H NMR, 77.16 ppm ^{13}C NMR; CD_3CN @ 1.94 ppm ^1H NMR, 1.32; 118.26 ppm ^{13}C NMR). The following abbreviations were used to explain NMR peak multiplicities: s = singlet, d = doublet, t = triplet, q = quartet, m = multiplet, br = broad. High- resolution mass spectra (HRMS) were recorded on an Agilent LC/MSD TOF mass spectrometer by electrospray ionization time-of-flight (ESI-TOF) reflection experiments. Analytical HPLC was performed with an Agilent 1290 Infinity II HPLC and a Daicel Chiralpak® AD-H column that is 4.6 mm I.D. x 250 mm in length and particle size of 5 µm.

Experimental procedures

Synthesis and titration of fluorosulfonyl azide **2a**

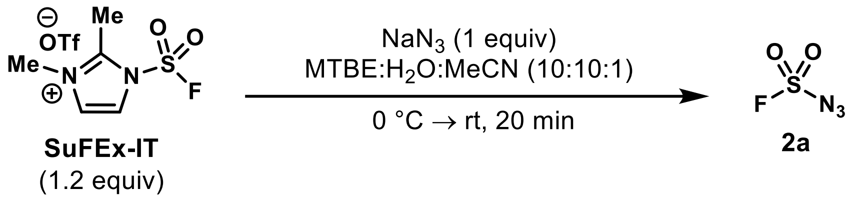

A stock solution of **2a** in MTBE was prepared following a previously reported procedure with minor modifications.[6] A 100 mL cylindrical plastic (PTFE) bottle was charged with an aqueous solution of NaN_3 (C = 0.5 M, 30 mL) prepared from DI water and NaN_3 (1g, 15 mmol) and MTBE (30 ml) and cooled to 0 °C with an ice bath. SuFEx-IT (5.9 g, 18 mmol) was dissolved in MeCN (3 ml), and the resultant viscous solution was slowly added dropwise to the NaN_3/H_2O/MTBE mixture under vigorous stirring. The reaction mixture was stirred vigorously at 0 °C for 20 min in the loosely sealed plastic bottle, then poured into a glass separatory funnel. The mixture was allowed to separate for 45 min at rt. The organic phase containing **2a** was collected and kept in a loosely sealed plastic bottle at room temperature overnight. Any residual water traces clearly visible due to the orange-red color was subsequently removed with a pipette. The colorless organic phase was used as a solution of **2a** in MTBE without further purification.

Titration of **2a**: An aliquot of the solution of **2a** (100 µL) was collected and combined with *para*-toluenesulfonyl fluoride (TsF) (10.6 mg, 61 µmol) as an internal standard for ^{19}F NMR, followed by the addition of $CDCl_3$. The yield of **2a** (93%) was calculated from the ratio between the signal integrals of **2a** and TsF in the ^{19}F NMR spectrum **(Figure S1)** and extrapolated for the mother solution.

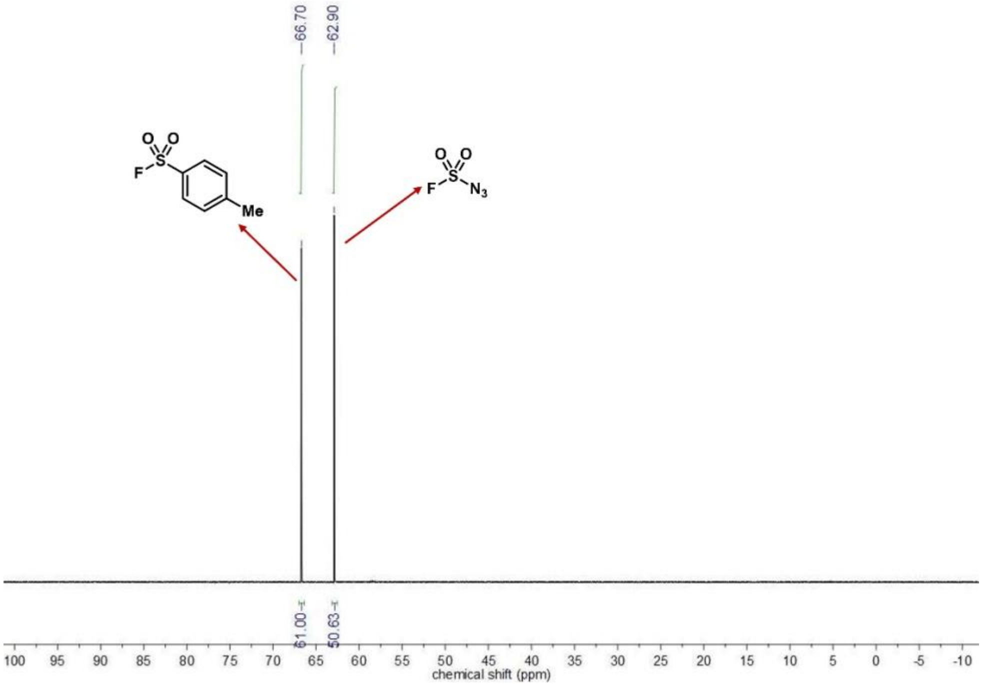

Figure S1. ^{19}F NMR spectrum of FSO_2N_3 (**2a**) with TsF as internal standard

Synthesis and titration of sulfamoyl fluoride **1**:

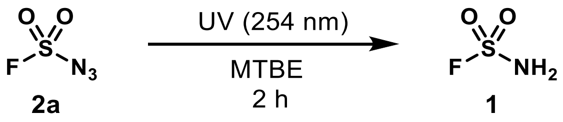

A stock solution of **1** in MTBE (8 mL, C ~ 0.4–0.5 M) was transferred into a 18 mL quartz reaction tube. The quartz tube was closed with a rubber septum and a needle pierced through the septum to avoid pressure buildup due to gas evolution or MTBE evaporation during the reaction. The reaction tube was then irradiated under two 254 nm UV lamps (Rayonet RPR-2537A) placed 1 cm apart from the reaction tube for 2 h **(Figure S2A)**. The conversion of **2a** to **1** was monitored by taking aliquots for ^{19}F NMR in $CDCl_3$. The reaction was generally done in 2 h. The mixture was then transferred into a glass separatory funnel and water (4 mL) was added. The biphasic mixture was shaken vigorously and allowed to separate for 30 minutes. The organic phase containing **1** was collected and kept in a tightly sealed plastic container inside the refrigerator at 4 °C to minimize evaporation of MTBE and maintain the concentration of **1**.

Titration: An aliquot of the solution of **1** (100 µL) was collected and combined with *para*-toluenesulfonyl fluoride (TsF) (14.9 mg, 85 µmol) as an internal standard for ^{19}F NMR, followed by the addition of $CDCl_3$. The yield of **1** (~64% yield) was calculated from the ratio between the signal integrals of **1** and TsF in the ^{19}F NMR spectrum **(Figure S2B)** and extrapolated for the mother solution.

Note: During the UV exposition, the volume of MTBE decreases by 20–25%.

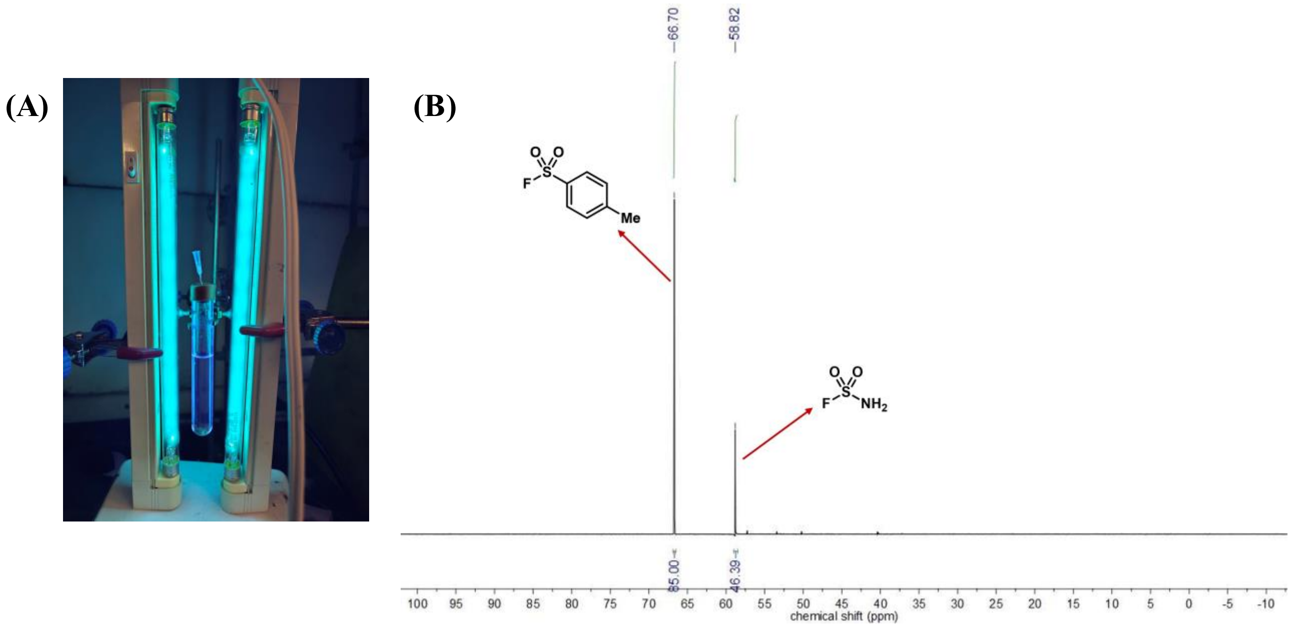

Figure S2. (A) Reaction setup. **(B)** ^{19}F NMR of FSO_2NH_2 **(1)** in $CDCl_3$ with TsF as internal standard.

Preparation of starting materials

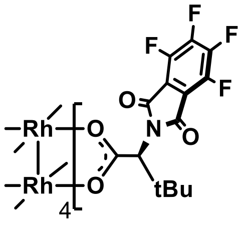

(*S*)-**Rh-1**

Tetrakis [N-tetrafluorophthaloyl-(*S*)-tert-leucinato] dirhodium $Rh_2[(S)tfpttl]_4$ ((*S*)-Rh-1). (*S*)-**Rh-1** was synthesized following a previously reported procedure[7] with minor modifications. To a flame-dried flask, (*S*)-3,3-dimethyl-2-(4,5,6,7-tetrafluoro-1,3-dioxoisoindolin-2-yl) butanoic acid (287 mg, 0.86 mmol, 4.50 equiv) (for synthesis look ref 7) and $Rh_2(OAc)_4$ (80 mg, 0.18 mmol, 1 equiv) were added, followed by chlorobenzene (10 mL). The flask was connected to a Soxhlet extractor containing a cartridge filled with a mixture of Na_2CO_3 and sand (3:1). The solution was heated to 145 °C for 48 h. The reaction mixture was then cooled to room temperature and concentrated *in vacuo*. The solid residues were purified by column chromatography (SiO_2, 10% EtOAc:Hexanes) to provide (*S*)-**Rh-1** as a green solid (220 mg, 79%).

The spectroscopic data for this compound was identical to those reported in the literature.[7] ^{1}H NMR (400 MHz, $CDCl_3$) δ: 4.70 (s, 4 H), 1.08 (s, 36 H) ppm.

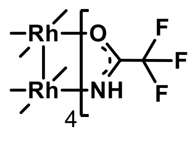

Rh-3

Rhodium (II) trifluoroacetamide $Rh_2(tfacam)_4$ (Rh-3). Rh-3 was synthesized following a slightly modified version of the previously reported procedure.[8] To a 50 mL flame-dried round bottom flask equipped with a stir bar was added a mixture of trifluoroacetamide (68 mg, 0.67 mmol, 10.00 equiv), $Rh_2(OAc)_4$ (27 mg, 0.067 mmol, 1.00 equiv) and chlorobenzene (10 mL). The flask was connected to a Soxhlet extractor containing a catridge filled with a mixture of Na_2CO_3 and sand (3:1). The suspension was refluxed at 155 °C for 48 h. The reaction mixture was concentrated *in vacuo* and the residual viscous solution was precipitated through the addition of DCM (~ 1–5 mL). The resulting green solid residues were filtered in a Büchner funnel equipped with a filter paper, washed thoroughly with DCM and the filtrate was discarded. The solids remaining on the filter were then dissolved in acetone (~10 mL), filtered, and the filtrate was concentrated under reduced pressure. The isolated solid was dried at 50 °C under high vacuum (0.15 mbar) overnight. **Rh-3** was obtained as a dark green powder (31 mg, 79%) and used without further purification.

The spectroscopic data for this compound was identical to those reported in the literature.[8] ^{19}F NMR (376 MHz, CD_3CN) δ: –74.4 ppm.

Preparation of alkene substrates

Synthesis and characterization of **3p**

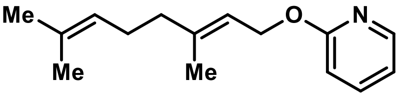

3p

To a flame-dried 25 mL round-bottom flask were added geraniol (500 mg, 3.2 mmol, 1.0 equiv), PPh_3 (1.1 g, 4.2 mmol, 1.3 equiv), 2-pyridinol (308 mg, 3.2 mmol, 1.0 equiv), and THF (4 mL). The mixture was cooled to 0 °C and stirred for 5 min, then DIAD (850 mg, 4.2 mmol, 1.3 equiv) was added dropwise. The reaction was then warmed to room temperature and stirred for 16 h, concentrated under reduced pressure, and diluted with diethyl ether:hexane (1:1, 25 mL). The resultant precipitate was filtered, washed with diethyl ether (50 mL), and the filtrate was concentrated under rotavap. Purification by column chromatography (SiO_2, 5% EtOAc/hexanes) afforded **3p** as a colorless oil (223 mg, 30%).

R_f = 0.6 (10:90 EtOAc:hexanes)

^{1}H NMR (400 MHz, $CDCl_3$) δ: 8.16 (d, J = 4.0 Hz, 1 H), 7.55 (t, J = 6.8 Hz, 1 H), 6.85 (t, J = 6 Hz, 1 H), 6.75 (d, J = 8.4 Hz, 1 H), 5.53 (t, J = 6.8 Hz, 1 H), 5.10 (t, J = 6.7 Hz, 1 H), 4.84 (d, J = 6.8 Hz, 2 H), 2.22 – 1.98 (m, 4 H), 1.75 (s, 3 H), 1.68 (s, 3 H), 1.60 (s, 3 H) ppm.

^{13}C NMR (101 MHz, $CDCl_3$) δ 164.0, 147.0, 141.4, 138.6, 131.9, 124.1, 119.6, 116.7, 111.4, 62.9, 39.8, 26.5, 25.8, 17.8, 16.8 ppm.

HRMS(+ESI) calc'd for $C_{15}H_{21}NO$ $[M+H]^+$ 232.1696, found 232.1692.

Synthesis and characterization of **3s**

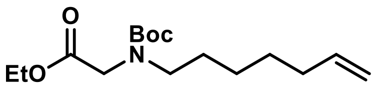

3s

To a flame-dried round-bottom flask equipped with a magnetic stir bar were added glycine ethyl ester hydrochloride (1.2 g, 8.4 mmol, 1.5 equiv), 7-bromo-1-heptene (1 g, 5.6 mmol, 1 equiv), K_2CO_3 (3.5 g, 25.2 mmol, 4.5 equiv), and DMF (10 mL). The reaction mixture was stirred at rt for 24 h. Upon completion as monitored by TLC, the mixture was diluted with water (50 mL) and extracted with EtOAc (3 × 50 mL). The combined organic layers were washed with brine (100 mL), dried over Na_2SO_4, filtered, and concentrated under reduced pressure to give crude product. The crude was dissolved in DCM (10 mL), and Et_3N (850 mg, 8.4 mmol, 1.5 equiv) and DMAP (73 mg, 0.6 mmol, 0.1 equiv) were added. The solution was cooled to 0 °C, and Boc_2O (1.8 g, 8.4 mmol, 1.5 equiv) was added dropwise. The reaction was allowed to warm to room temperature and stirred for 12 h. The mixture was quenched with saturated NH_4Cl (50 mL) and extracted with DCM (3 x 50 mL). The combined organic extracts were washed with brine (50 mL), dried over Na_2SO_4, filtered, and concentrated under reduced pressure. Purification by column chromatography (SiO_2, 15% EtOAc/hexanes) afforded **3s** as a colorless liquid (235 mg, 15% over two steps).

R_f = 0.63 (30:70 EtOAc: hexanes)

^{1}H NMR (CDCl$_3$, 400 MHz) δ: 5.87 – 5.71 (m, 1 H), 5.04 – 4.89 (m, 2 H), 4.18 (m, 2H), 3.93 (s, 1 H), 3.83 (s, 1 H), 3.25 (dt, J = 14.9, 7.4 Hz, 2 H), 2.10 – 1.99 (m, 2 H), 1.54 – 1.36 (m, 15 H), 1.30 – 1.23 (m, 3 H) ppm.

^{13}C NMR (126 MHz, CDCl$_3$) δ: 170.4, 156.0, 155.3, 139.0, 114.5, 81.2, 80.1, 61.1, 49.6, 48.9, 48.5, 33.8, 28.8, 28.7, 28.5, 28.4, 28.2, 28.1, 26.4, 14.4 ppm.

HRMS(+ESI) calc'd for $C_{14}H_{27}NO_4$ $[M+H]^+$ 300.2169, found 300.2162.

<u>Synthesis and characterization of **3t**</u>

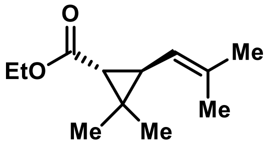

3t

To a flame dried round bottom flask equipped with a magnetic stir bar was added *trans*-chrysanthemic acid (186 µL, 1.18 mmol, 1 equiv), DMAP (144 mg, 1.18 mmol, 1 equiv), 1-Ethyl-3-(3-dimethylaminopropyl)carbodiimide hydrochloride (EDC.HCl) (339 mg, 1.77 mmol, 1.5 equiv), EtOH (1.5 ml, 24 mmol, 20 equiv), DCM (6 ml). and stirred at rt for 16 h. The mixture was diluted with water (15 mL) and extracted with DCM (3 x 20 ml). The combined organic fraction was dried over Na_2SO_4 and evaporated to obtain the crude product. Purification of the crude via column chromatography (SiO_2, 15% EtOAc/Hexanes) afforded **3t** as a colorless liquid (150 mg, 61%).

R_f = 0.60 (10:90 EtOAc:hexanes)

^{1}H NMR (400 MHz, CDCl$_3$) δ: 4.89 (d, J = 7.9 Hz, 1 H), 4.12 (qd, J = 7.2, 5.1 Hz, 2 H), 2.04 (dd, J = 8.0, 5.2 Hz, 1 H), 1.71 (d, J = 3.0 Hz, 6 H), 1.37 (d, J = 5.4 Hz, 1 H), 1.30 – 1.22 (m, 6 H), 1.13 (s, 3 H).

^{13}C NMR (126 MHz, CDCl$_3$) δ: 172.7, 135.6, 121.4, 60.3, 35.1, 32.7, 28.6, 25.7, 22.4, 20.6, 18.6, 14.5 ppm.

HRMS(+ESI) calc'd for $C_{13}H_{22}O_2$ $[M+H]^+$ 197.1541, found 197.1531.

<u>Synthesis and characterization of **3u'** and **3u**</u>

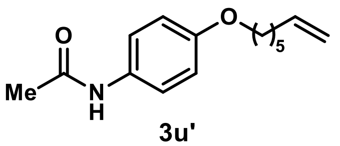

3u'

To a flame dried round bottom flask equipped with a magnetic stir bar was added acetaminophen (342 mg, 2.3 mmol, 1 equiv), 7-Bromo-1-heptene (516 µL, 3.4 mmol, 1.5 equiv), K_2CO_3 (937 g, 6.8 mmol, 3 equiv), and DMF (4.5 mL). The mixture was heated at 60 °C for 24 h. Upon completion, the mixture was cooled to room temperature, diluted with water (25 mL), and extracted with EtOAc (3 x 15 ml). The combined organic fraction was dried over Na_2SO_4, filtered, and evaporated under reduced pressure to obtain the crude product. Purification of the crude via column chromatography (SiO_2, 10–30% EtOAc/Hexanes) afforded **3u'** as a white solid (651 mg, 81%).

R_f = 0.53 (50:50 EtOAc: hexanes)

^{1}H NMR ($CDCl_3$, 400 MHz) δ: 7.41 – 7.32 (m, 2 H), 7.05 (s, 1 H), 6.91 – 6.80 (m, 2 H), 5.82 (ddt, J = 16.9, 10.2, 6.6 Hz, 1 H), 5.01 – 4.91 (m, 2 H), 3.93 (t, J = 6.5 Hz, 2 H), 2.15 (s, 3 H), 2.08 (m, 2 H), 1.77 (m, 2 H), 1.47 (m, 4 H) ppm.

^{13}C NMR ($CDCl_3$, 101 MHz) δ: 168.0, 156.1, 138.8, 130.8, 121.9, 114.8, 114.4, 68.2, 33.7, 29.1, 28.7, 25.5, 24.4 ppm.

HRMS(+ESI) calc'd for $C_{15}H_{21}NO_2$ $[M+H]^+$ 248.1643, found 248.1645.

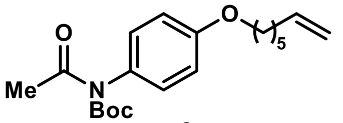

3u

In a flame dried 25 mL round bottom flask equipped with a magnetic stir bar was added **3u'** (300 mg, 1.2 mmol, 1 equiv), Et_3N (0.25 mL, 1.5 mmol, 1.2 equiv), DMAP (14.8 mg, 0.12 mmol, 0.1 equiv), and DCM (3.60 mL) were added. The reaction mixture was cooled to 0 °C and Boc_2O (0.33 ml, 1.5 mmol, 1.2 equiv) was added. The mixture was stirred at rt for 12 h. Upon completion, the reaction was quenched with saturated NH_4Cl (10 mL). The mixture was diluted with water (10 mL), extracted with DCM (3 x 15 mL), and the combined organic fraction was washed with brine. The organic fraction was dried over Na_2SO_4 and evaporated to obtain the crude product. Purification of the crude via column chromatography (SiO_2, 5-15% EtOAc/Hexanes) afforded **3u** as a colorless liquid (340 mg, 81%).
R_f = 0.79 (30:70 EtOAc: hexanes)

^{1}H NMR ($CDCl_3$, 400 MHz) δ: 7.01 – 6.93 (m, 2 H), 6.92 – 6.84 (m, 2 H), 5.82 (m, 1 H), 5.06 – 4.91 (m, 2 H), 3.95 (t, J = 6.5 Hz, 2 H), 2.54 (s, 3 H), 2.14 – 2.02 (m, 2H), 1.79 (m, 2 H), 1.56 – 1.40 (m, 4 H), 1.39 (s, 9 H) ppm.

^{13}C NMR ($CDCl_3$, 101 MHz) δ: 173.2, 158.4, 153.1, 138.8, 131.4, 129.0, 114.7, 114.5, 83.0, 68.1, 33.7, 29.1, 28.7, 27.9, 26.5, 25.6 ppm.

HRMS(+ESI) calc'd for $C_{20}H_{29}NO_4$ $[M+H]^+$ 348.2169, found 348.2165.

<u>Synthesis and characterization of **3w'** and **3w**</u>

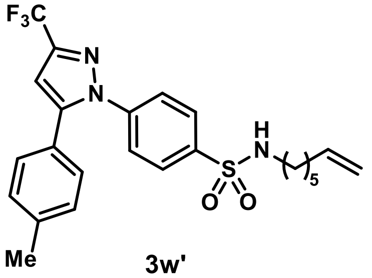

3w'

In a flame dried 50 mL round bottom flask equipped with a stir bar was added celecoxib (1.3 g, 3.4 mmol, 1.2 equiv), K_2CO_3 (1.4 g, 10 mmol, 3 equiv), DMF (15 mL) and stirred for 5 min. Then 7-bromo-heptene (500 mg, 2.8 mmol, 1.0 equiv) was added and the reaction mixture was heated at 70 °C for 24 h. The reaction was diluted with water (50 mL) and extracted with EtOAc (3 x 50 mL). The combined organic layers were washed with brine (50 mL), dried over $MgSO_4$ and concentrated under a rotavap to obtain the crude

product. Purification of the crude via column chromatography (SiO_2, 10% EtOAc/hexanes) afforded **3w'** as a white solid (618 mg, 46%).

R_f = 0.13 (15:85 EtOAc:hexanes)

^{1}H NMR (400 MHz, $CDCl_3$) δ: 7.84 (d, J = 8 Hz, 2 H), 7.47 (d, J = 8 Hz, 2 H), 7.17 (d, J = 8 Hz, 2 H), 7.10 (d, J = 8 Hz, 2 H), 6.74 (s, 1 H), 5.75 (ddt, J = 16.9, 10.1, 6.7 Hz, 1 H), 5.02 – 4.89 (m, 2 H), 4.41 (t, J = 6.1 Hz, 1 H), 2.93 (q, J = 6 Hz, 2 H), 2.01 (q, J = 6.9 Hz, 2 H), 1.53 – 1.41 (m, 2 H), 1.40 – 1.21 (m, 4 H) ppm.

^{13}C NMR (400 MHz, $CDCl_3$) δ: 145.4, 144.2 (q, J = 40 Hz), 142.6, 139.9, 139.6, 138.6, 129.9, 128.8, 128.2, 125.8, 125.7, 122.5, 119.8, 114.8, 106.4, 77.5, 77.4, 77.2, 76.8, 43.4, 33.6, 29.6, 28.4, 26.1, 21.5 ppm.

^{19}F NMR (377 MHz, $CDCl_3$) δ: –62.5 ppm

HRMS (+ESI) calc'd for $C_{24}H_{26}F_3N_3O_2S$ $[M+H]^+$ 478.1781, found 478.1786.

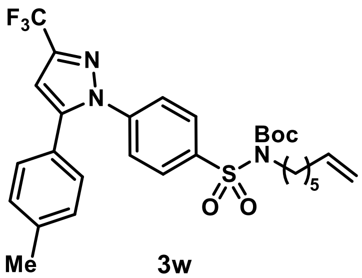

3w

In a flame dried 50 mL round bottom flask equipped with a stir bar was charged **3w'** (500 mg, 1 mmol, 1 equiv), Boc_2O (460 mg, 2.1 mmol, 2 equiv), DMAP (25 mg, 0.2 mmol, 0.2 equiv), DCM (20 mL) and stirred for 5 minutes at rt. The solution was cooled to 0 °C and Et_3N (210 mg, 2.1 mmol, 2 equiv) was added dropwise. The reaction was allowed to warm to room temperature and stirred for 12 h. The mixture was quenched with saturated NH_4Cl (50 mL) and extracted with DCM (3 × 50 mL). The combined organic extracts were washed with brine (50 mL), dried over Na_2SO_4, filtered, and concentrated under reduced pressure. Purification by column chromatography (SiO_2, 10% EtOAc/hexanes) afforded **3w** as a white solid (600 mg, 99%).

R_f = 0.40 (10:90 EtOAc:hexanes)

^{1}H NMR ($CDCl_3$, 400 MHz) δ: 7.91 – 7.85 (m, 2 H), 7.50 – 7.43 (m, 2 H), 7.17 (d, J = 8.0 Hz, 2 H), 7.10 (d, J = 8.2 Hz, 2 H), 6.74 (s, 1H), 5.80 (ddt, J = 16.9, 10.2, 6.7 Hz, 1 H), 5.04 – 4.91 (m, 2 H), 3.83 – 3.76 (m, 2 H), 2.38 (s, 3 H), 2.11 – 2.03 (m, 2 H), 1.74 (p, J = 7.6 Hz, 2 H), 1.49 – 1.42 (m, 2 H), 1.35 (m, 11 H) ppm.

^{13}C NMR ($CDCl_3$, 126 MHz) δ: 150.7, 145.3, 144.2 (q, J = 38.6 Hz), 142.8, 139.8, 139.7, 129.8, 128.9, 128.8, 125.8, 124.9, 122.1, 120.0, 114.6, 106.5, 84.5, 47.4, 33.6, 30.0, 28.4, 27.9, 26.1, 21.3 ppm.

^{19}F NMR (377 MHz, $CDCl_3$) δ: –62.5 ppm

HRMS(+ESI) calc'd for $C_{29}H_{34}F_3N_3O_4S$ $[M+H]^+$ 578.2295, found 578.2289

Aziridination reaction optimization

Table S1. Catalyst screening[a]

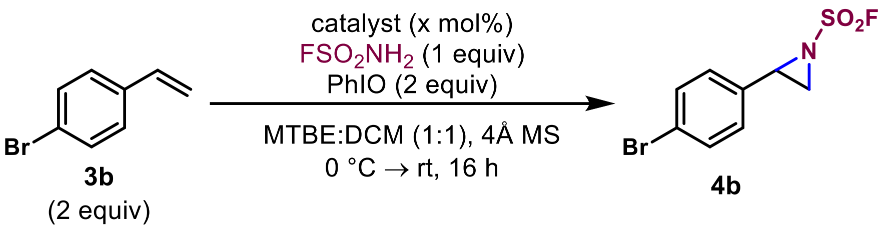

Entry	Catalyst (x mol%)	Yield (%)[b]
1	–	Trace
2	CuI (10 mol%)	6
3	CuBr (10 mol%)	10
4	$Cu[(CH_3CN)_4]PF_6$ (10 mol%)	12
5	$Cu(OTf)_2$ (10 mol%)	12
6	$Cu(OAc)_2$ (10 mol%)	8
7	AgOTf (10 mol%)[c]	36
8	$Rh_2(esp)_2$ (**Rh-2**) (2 mol%)	50
9	$Rh_2(OAc)_4$ (2 mol%)	51
10	$Rh_2(tfacam)_4$ (**Rh-3**) (2 mol%)	74
11	$Rh_2[(S)\text{-tfpttl}]_4$ ((*S*)-**Rh-1**) (2 mol%)	99

[a]Reaction conditions: All reactions performed in 0.2 mmol scale with respect to FSO_2NH_2 (**1**). Using catalyst (x mol%), **3b** (0.4 mmol), solution of **1** in MTBE (0.2 mmol), PhIO (0.4 mmol) and dry DCM under Ar atmosphere at rt in presence of 4Å MS. [b]Yields were determined by ^1H NMR spectroscopy (400 MHz, $CDCl_3$) using 1,2,4,5-tetrabromobenzene as an internal standard. [c]12 mol% ^{t}Bu-bpy used as ligand.

Table S2. Oxidant screening[a]

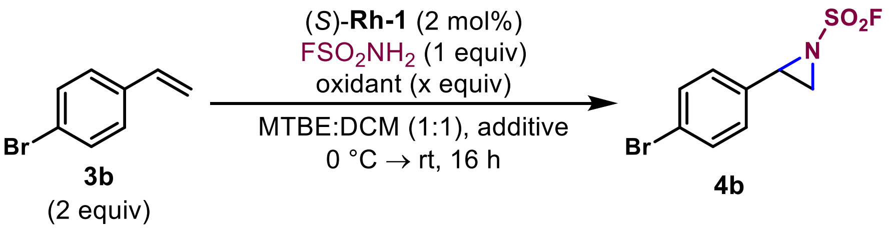

Entry	Oxidant	Equivalence	Additive	Yield (%)[b]
1	PIDA	2	MgO (3 equiv)	75
2	PIFA	2	MgO (3 equiv)	17
3	$PhI(OPiv)_2$	2	MgO (3 equiv)	85
4	PhIO	2	4Å MS	99
5	PhIO	1	4Å MS	40
6	PhIO	2	–	–

[a] Reaction conditions: All reactions performed in 0.2 mmol scale with respect to FSO_2NH_2 (**1**). Using (*S*)-**Rh-1** (2 mol%), **3b** (0.4 mmol), solution of **1** in MTBE (0.2 mmol), oxidant (0.4 mmol) and dry DCM under Ar atmosphere at rt. [b] Yields were determined by ^{1}H NMR spectroscopy (400 MHz, $CDCl_3$) using 1,2,4,5-tetrabromobenzene as internal standard.

Table S3. Effect of alkene equivalence and catalyst loading[a]

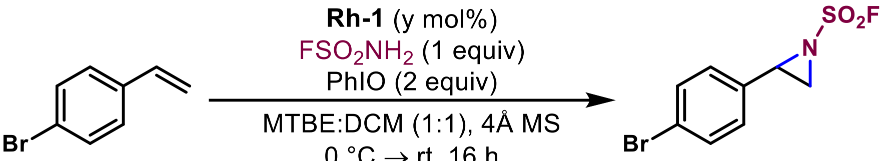

3b
(x equiv)
4b

Entry	**3b** (x equiv)	**Rh-1** (y mol%)	Yield[b]
1	2	2	99
2	1	2	58
3	2	1	68
4	1.5	1.5	99

[a]Reaction conditions: All reactions performed in 0.2 mmol scale with respect to FSO_2NH_2 (**1**). Using (*S*)-**Rh-1** (y mol%), **3b** (x mmol), solution of **1** in MTBE (0.2 mmol), oxidant (0.4 mmol) and dry DCM under Ar atmosphere at rt. [b] Yields were determined by ^{1}H NMR spectroscopy (400 MHz, $CDCl_3$) using 1,2,4,5-tetrabromobenzene as internal standard.

Aziridination method: **General procedure A**

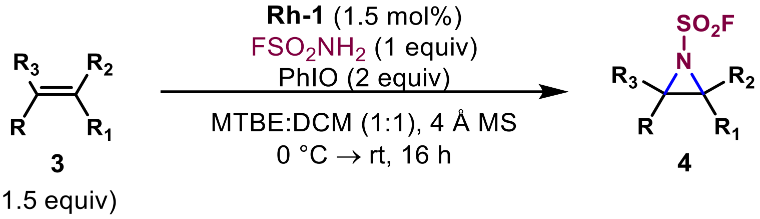

3
(1.5 equiv)
4

A flame-dried 25 mL round bottom flask was charged with a stir bar, activated 4 Å molecular sieves, and (*S*)-**Rh-1** catalyst (4.5 mg, 1.5 mol%). The flask was then evacuated under a high vacuum for 10 min and backfilled with argon. A solution of FSO_2NH_2 (**1**) in MTBE (0.2 mmol, 1 equiv) and the alkene substrate (1.5 equiv) was added under Ar. The solution was diluted with dry DCM (MTBE:DCM 1:1, C = 0.1 M). The reaction vial was then cooled to 0 °C with an ice bath and allowed to stir for 5 min. PhIO (2 equiv) was added in one portion quickly under an Ar flow, then the ice bath was removed, and the reaction mixture was stirred at room temperature for 16 h. The reaction mixture was subsequently diluted with DCM (5 mL) and filtered through a fritted funnel equipped with celite® (~1–1.5 cm). Additional DCM (20 mL) was used to elute the celite® plug. The volatiles were removed *in vacuo*, and crude residues were purified by silica chromatography.

Synthesis and characterization of aziridines

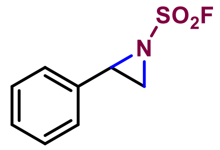

4a

4a was prepared from styrene **3a** (47 mg, 0.45 mmol, 1.5 equiv) and **1** (0.3 mmol) following **general procedure A**. Crude NMR analysis (^{1}H and ^{19}F) confirmed product formation. Yield = 99% determined by ^{1}H NMR analysis using 1,2,4,5-tetramethylbenzene (5.1 mg) as internal standard. Product degraded during column chromatography (SiO_2).

^{1}H NMR crude ($CDCl_3$, 400 MHz) δ: 7.50 – 7.36 (m, 5 H), 3.99 (dd, J = 7.1, 4.9 Hz, 1 H), 3.17 (d, J = 7.2 Hz, 1 H), 2.77 (d, J = 4.9 Hz, 1 H) ppm.

^{19}F NMR crude ($CDCl_3$, 376 MHz) δ: 37.5

HRMS(–ESI) calc'd for $C_8H_8FNO_2S$ $[M–H]^-$ 200.0176, found 200.0181.

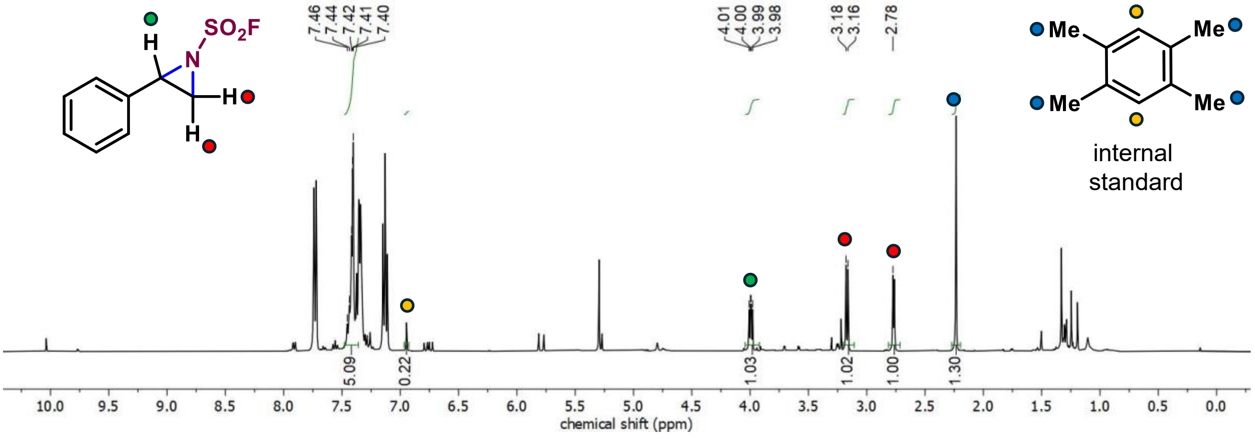

Figure S3. Crude ^{1}H NMR for **4a**

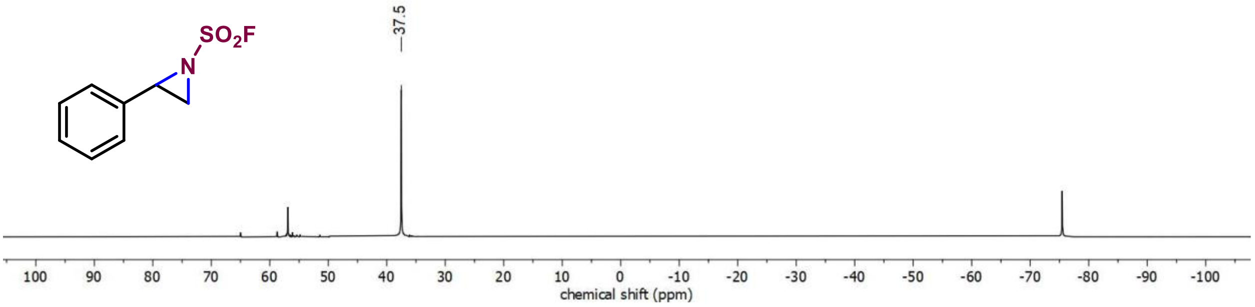

Figure S4. Crude ^{19}F NMR for **4a**

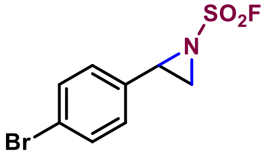

4b

4b was prepared from 4-bromostyrene **3b** (55 mg, 0.3 mmol, 1.5 equiv) following **general procedure A**. Column chromatography (SiO_2, 3:97 EtOAc:hexanes) afforded **4b** as colorless oil (43 mg, 78%).

R_f = 0.45 (5:95 EtOAc:hexanes)

^{1}H NMR ($CDCl_3$, 400 MHz) δ: 7.52 (d, J = 8.1 Hz, 2 H), 7.20 (d, J = 8.0 Hz, 2 H), 3.96–3.90 (m, 1 H), 3.17 (d, J = 7.2 Hz, 1 H), 2.72 (d, J = 4.5 Hz, 1 H) ppm.

^{13}C NMR ($CDCl_3$, 101 MHz) δ: 132.2, 132.0, 128.2, 123.5, 43.4, 38.6 ppm.

^{19}F NMR ($CDCl_3$, 376 MHz) δ: 37.9 ppm.

HRMS(–ESI) calc'd for $C_8H_7BrFNO_2S$ $[M–H]^-$ 277.9281, found 277.9294.

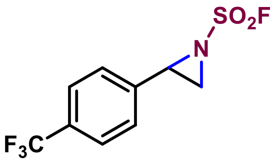

4c

4c was prepared from 4-trifluoromethylstyrene **3c** (52 mg, 0.3 mmol, 1.5 equiv) following **general procedure A**. Column chromatography (SiO_2, 3:97 EtOAc:hexanes) afforded **4c** as colorless oil (52 mg, 97%).

R_f = 0.21 (5:95 EtOAc:hexanes)

^{1}H NMR ($CDCl_3$, 400 MHz) δ: 7.66 (d, J = 8.1 Hz, 2 H), 7.47 (d, J = 8.0 Hz, 2 H), 4.02 (dd, J = 7.2, 4.8 Hz, 1 H), 3.21 (d, J = 7.2 Hz, 1 H), 2.75 (d, J = 4.7 Hz, 1 H) ppm.

^{13}C NMR (101 MHz, $CDCl_3$) δ 137.02, 132.10, 131.78, 131.45, 131.13, 127.94, 127.10, 126.12, 126.08, 126.05, 126.01, 125.24, 122.53, 119.82, 43.16, 38.81 ppm.

^{19}F NMR ($CDCl_3$, 376 MHz) δ: 37.8, -62.9 ppm.

HRMS(-ESI) calc'd for $C_9H_7F_4NO_2S$ $[M+Cl]^-$ 303.9817, found 303.9825.

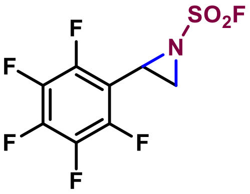

4d

4d was prepared from pentafluorostyrene **3d** (58 mg, 0.3 mmol, 1.5 equiv) following **general procedure A**. Column chromatography (SiO_2, 5:95 EtOAc:hexanes) afforded **4d** as colorless oil (54 mg, 93%).

R_f = 0.42 (5:95 EtOAc:hexanes)

^{1}H NMR ($CDCl_3$, 400 MHz) δ: 4.00 (dd, J = 7.0, 5.1 Hz, 1 H), 3.23 (d, J = 7.3 Hz, 1 H), 3.16 (d, J = 4.8 Hz, 1 H) ppm.

^{13}C NMR ($CDCl_3$, 101 MHz) δ: 147.7–147.2 (m), 145.2–144.8 (m), 143.8–143.2 (m), 141.2–140.7 (m), 139.4–138.7 (m), 136.9–136.3 (m), 107.1 (td, J = 14.6, 4.1 Hz), 35.2 (t, J = 4.1 Hz), 34.7 (d, J = 1.4 Hz) ppm.

^{19}F NMR ($CDCl_3$, 376 MHz) δ: 38.8, -141.50 – -141.88 (m), -150.4 – -150.6 (m), -160.4 (td, J = 19.9, 6.1 Hz) ppm

HRMS(–ESI) calc'd for $C_8H_3F_6NO_2S$ $[(M+MeOH)–H]^-$ 321.9967, found 321.9978.

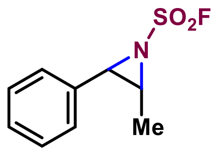

cis-**4e**

cis-**4e** was prepared from *cis*-β-methylstyrene (*cis*-**3e**) (35 mg, 0.3 mmol, 1.5 equiv) following **general procedure A**. Column chromatography (SiO_2, 5:95 EtOAc:hexanes) afforded **4e** as *cis*-diastereomer only. The product appeared as a colorless oil (42 mg, 98%).

R_f = 0.43 (5:95 EtOAc:hexanes)

^{1}H NMR ($CDCl_3$, 400 MHz) δ: 7.46–7.31 (m, 5 H), 4.17 (d, J = 7.3 Hz, 1 H), 3.45–3.32 (m, 1 H), 1.18 (d, J = 5.8 Hz, 3 H) ppm.

^{13}C NMR (101 MHz, $CDCl_3$) δ: 130.76, 128.80, 128.75, 127.49, 49.06, 45.68, 11.80 ppm.

^{19}F NMR ($CDCl_3$, 376 MHz) δ: 37.7 ppm (*cis*-isomer).

HRMS(–ESI) calc'd for $C_9H_{10}FNO_2S$ $[M–H]^-$ 214.0333, found 214.0339.

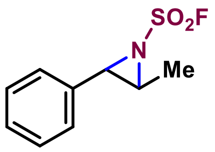

trans-**4e**

trans-**4e** was prepared from *trans*-β-methylstyrene (trans-**3e**) (35 mg, 0.3 mmol, 1.5 equiv) following **general procedure A**. Column chromatography (SiO_2, 5:95 EtOAc:hexanes) afforded *trans*-**4e** as a mixture of *trans* and *cis* diastereomers with *trans* being the major isomer determined by ^{19}F NMR analysis (*trans*:*cis* = 22:1). The product appeared as colorless oil (35 mg, 82%).

R_f = 0.44 (5:95 EtOAc:hexanes)

^{1}H NMR ($CDCl_3$, 400 MHz) δ: 7.46–7.31 (m, 5 H), 4.17 (d, J = 7.3 Hz, 1 H), 3.45–3.32 (m, 1 H), 1.18 (d, J = 5.8 Hz, 3 H) ppm.

^{13}C NMR ($CDCl_3$, 101 MHz) (*trans*-isomer) δ: 133.4, 129.3, 129.0, 126.7, 52.1, 49.3, 13.9 ppm.

^{19}F NMR ($CDCl_3$, 376 MHz) δ: 55.1 ppm (*trans*-isomer), 37.7 ppm (*cis*-isomer).

HRMS(–ESI) calc'd for $C_9H_{10}FNO_2S$ $[M–H]^-$ 214.0333, found 214.0339.

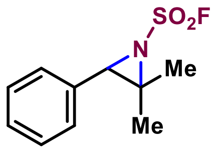

4f

4f was prepared from β, β-dimethyl styrene **3f** (40 mg, 0.3 mmol, 1.5 equiv) following **general procedure A**. Column chromatography (SiO_2, 5:95 EtOAc:hexanes) afforded **4f** as colorless oil (45 mg, 98%).

R_f = 0.52 (5:95 EtOAc:hexanes)

^{1}H NMR ($CDCl_3$, 400 MHz) δ: 7.44–7.31 (m, 5 H), 4.14 (s, 1 H), 1.82 (d, J = 2.3 Hz, 3 H), 1.17 (s, 3 H) ppm.

^{13}C NMR ($CDCl_3$, 101 MHz) δ: 132.3, 128.7, 128.6, 127.1, 56.5, 55.3, 21.0, 20.3 ppm.

^{19}F NMR ($CDCl_3$, 376 MHz) δ: 55.0 ppm.

HRMS(–ESI) calc'd for $C_{10}H_{12}FNO_2S$ $[M–H]^-$ 228.0489, found 228.0496.

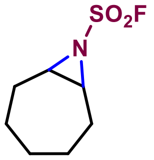

4g

4g was prepared from Cycloheptene **3g** (29 mg, 0.3 mmol, 1.5 equiv) following **general procedure A**. Column chromatography (SiO_2, 5:95 EtOAc:hexanes) afforded **4g** as colorless oil (29 mg, 76%).

R_f = 0.41 (5:95 EtOAc:hexanes)

^{1}H NMR ($CDCl_3$, 400 MHz) δ: 3.24–3.13 (m, 2 H), 2.15–2.05 (m, 2 H), 1.97–1.87 (m, 2 H), 1.72–1.63 (m, 1 H), 1.59–1.47 (m, 4 H), 1.23–1.12 (m, 1 H) ppm.

^{13}C NMR ($CDCl_3$, 101 MHz) δ: 48.2, 30.8, 27.9, 24.9 ppm.

^{19}F NMR ($CDCl_3$, 376 MHz) δ: 36.9 ppm.

HRMS(+ESI) calc'd for $C_7H_{12}FNO_2S$ $[M+H]^+$ 194.0646, found 194.0644.

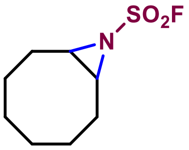

4h

4h was prepared from *cis*-Cyclooctene **3h** (33 mg, 0.3 mmol, 1.5 equiv) following **general procedure A**. Column chromatography (SiO_2, 5:95 EtOAc:hexanes) afforded **4h** as colorless oil (40 mg, 98%). This reaction has been scaled up to 2 mmol (product obtained = 373 mg, 90%).

R_f = 0.43 (5:95 EtOAc:hexanes)

^{1}H NMR ($CDCl_3$, 400 MHz) δ: 3.08–2.90 (m, 2 H), 2.34–2.20 (m, 2 H), 1.73–1.63 (m, 2 H), 1.57–1.39 (m, 8 H) ppm.

^{13}C NMR ($CDCl_3$, 101 MHz) δ: 47.6, 26.2, 26.1, 25.1 ppm.

^{19}F NMR ($CDCl_3$, 376 MHz) δ: 37.7 ppm.

HRMS(+ESI) calc'd for $C_8H_{14}FNO_2S$ $[M+H]^+$ 208.0802, found 208.0801.

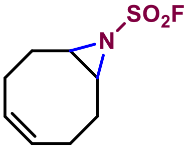

4i

4i was prepared from 1,5-Cyclooctadiene **3i** (32 mg, 0.3 mmol, 1.5 equiv) following **general procedure A**. Column chromatography (SiO_2, 3:97 EtOAc:hexanes) afforded **4i** as colorless oil (40 mg, 98%).

R_f = 0.31 (5:95 EtOAc:hexanes)

^{1}H NMR ($CDCl_3$, 400 MHz) δ: 5.71–5.52 (m, 2 H), 3.17–2.99 (m, 2 H), 2.39–2.51 (m, 2 H), 2.29–2.11 (m, 4 H), 2.11–2.00 (m, 2 H) ppm.

^{13}C NMR ($CDCl_3$, 101 MHz) δ: 129.1, 48.1, 27.2, 23.6 ppm.

^{19}F NMR ($CDCl_3$, 376 MHz) δ: 37.0 ppm.

HRMS(-ESI) calc'd for $C_8H_{12}FNO_2S$ $[M-H]^-$ 206.0646, found 206.0644.

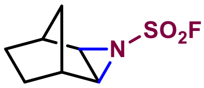

4j

4j was prepared from Bicyclo [2.2.1] hept-2-ene **3j** (28 mg, 0.3 mmol, 1.5 equiv) following **general procedure A**. Column chromatography (SiO_2, 3:97 EtOAc:hexanes) afforded **4j** as a viscous oil (17 mg, 45%) which formed needle shaped crystals on standing.

R_f = 0.44 (5:95 EtOAc:hexanes)

^{1}H NMR ($CDCl_3$, 400 MHz) δ: 3.15 (s, 2H), 2.64 (s, 2H), 1.62–1.54 (m, 2 H), 1.54–1.48 (m, 1 H), 1.34–1.26 (m, 2 H), 0.90 (d, J = 10.1 Hz, 1 H) ppm.

^{13}C NMR ($CDCl_3$, 101 MHz) δ: 45.3, 36.0, 28.0, 25.2 ppm.

^{19}F NMR ($CDCl_3$, 376 MHz) δ: 38.9 ppm.

HRMS(-ESI) calc'd for $C_7H_{10}FNO_2S$ $[M-H]^-$ 190.0333, found 190.0336.

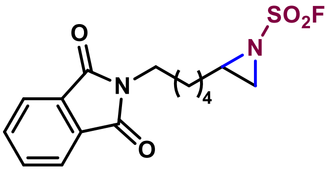

4k

4k was prepared from **3k** (60 mg, 0.3 mmol, 1.5 equiv) following **general procedure A**. Column chromatography (SiO_2, 10:90 EtOAc:hexanes) afforded **4k** as colorless oil (66 mg, 98%).

R_f = 0.3 (20:80 EtOAc:hexanes)

^{1}H NMR (CDCl$_3$, 400 MHz) δ: 7.82 (dd, J = 5.4, 3.1 Hz, 2 H), 7.70 (dd, J = 5.4, 3.0 Hz, 2 H), 3.67 (t, J = 7.1 Hz, 2 H), 3.00–2.87 (m, 1 H), 2.80 (d, J = 7.0 Hz, 1 H), 2.43 (d, J = 4.9 Hz, 1 H), 1.74–1.63 (m, 4 H), 1.59–1.47 (m, 2 H), 1.45–1.35 (m, 2 H) ppm.

^{13}C NMR (CDCl$_3$, 101 MHz) δ: 168.7, 134.1, 132.2, 123.4, 43.7, 37.8, 36.7, 30.9, 28.4, 26.2, 26.0 ppm.

^{19}F NMR (CDCl$_3$, 376 MHz) δ: 36.7 ppm.

HRMS(-ESI) calc'd for $C_{15}H_{17}FN_2O_4S$ $[M+Cl]^-$ 375.0576, found 375.0588.

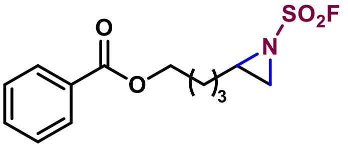

4l

4l was prepared from **3l** (62 mg, 0.3 mmol, 1.5 equiv) following **general procedure A**. Column chromatography (SiO$_2$, 7:93 EtOAc:hexanes) afforded **4l** as colorless oil (52 mg, 86%).

R_f = 0.15 (15% EtOAc:hexanes)

^{1}H NMR (CDCl$_3$, 400 MHz) δ: 8.04 (d, J = 8.3 Hz, 2 H), 7.56 (t, J = 6.8 Hz, 1 H), 7.45 (t, J = 7.7 Hz, 2 H), 4.34 (td, J = 6.4, 2.9 Hz, 2 H), 3.04 – 2.93 (m, 1 H), 2.84 (d, J = 7.0 Hz, 1 H), 2.47 (d, J = 4.9 Hz, 1 H), 1.91 – 1.76 (m, 3 H), 1.76 – 1.51 (m, 3 H) ppm.

^{13}C NMR (CDCl$_3$, 101 MHz) δ: 166.7, 133.1, 130.3, 129.7, 128.5, 64.5, 43.5, 36.7, 30.7, 28.2, 23.2 ppm.

^{19}F NMR (CDCl$_3$, 376 MHz) δ: 36.7 ppm.

HRMS(+ESI) calc'd for $C_{13}H_{16}FNO_4S$ $[M+H]^+$ 302.0857, found 302.0853.

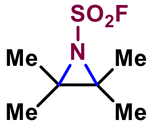

4m

4m was prepared from 2,3-dimethyl-2-butene **3m** (34 mg, 0.3 mmol, 1.5 equiv) following **general procedure A**. Column chromatography (SiO$_2$, 5:95 EtOAc:hexanes) afforded **4m** as a waxy solid (33 mg, 96%).

R_f = 0.42 (5:95 EtOAc:hexanes)

^{1}H NMR (CDCl$_3$, 400 MHz) δ: 1.52 (s, 12 H) ppm.

^{13}C NMR (CDCl$_3$, 101 MHz) δ: 56.0, 20.0 ppm.

^{19}F NMR (CDCl$_3$, 376 MHz) δ: 58.6 ppm.

HRMS(-ESI) calc'd for $C_6H_{12}FNO_2S$ $[M-H]^-$ 180.0489, found 180.0491.

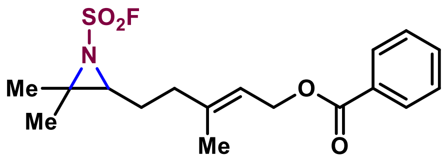

4n

4n was prepared from **3n** (78 mg, 0.3 mmol, 1.5 equiv) following **general procedure A**. Column chromatography (SiO_2, 5–10% EtOAc:hexanes) afforded **4n** as a colorless oil (69 mg, 98%).

R_f = 0.37 (15:85 EtOAc:hexanes)

^{1}H NMR ($CDCl_3$, 400 MHz) δ: 8.04 (d, J = 7.7 Hz, 2 H), 7.55 (t, J = 7.4 Hz, 1 H), 7.43 (t, J = 7.7 Hz, 2 H), 5.54 (t, J = 6.9 Hz, 1 H), 4.91–4.79 (m, 2 H), 2.93 (dd, J = 8.0, 5.3 Hz, 1 H), 2.33–2.19 (m, 2 H), 1.82–1.67 (m, 5 H), 1.62 (d, J = 2.2 Hz, 3 H), 1.38 (s, 3 H) ppm.

^{13}C NMR ($CDCl_3$, 101 MHz) δ: 166.7, 140.1, 133.0, 130.5, 129.7, 128.5, 120.3, 61.7, 55.8, 53.6, 36.6, 25.7, 21.2, 20.5, 16.6 ppm.

^{19}F NMR ($CDCl_3$, 376 MHz) δ: 54.4 ppm.

HRMS(-ESI) calc'd for $C_{17}H_{22}FNO_4S$ $[M-H]^-$ 354.1170, found 354.1183.

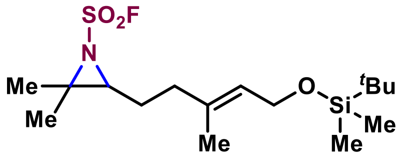

4o

4o was prepared from **3o** (80 mg, 0.3 mmol, 1.5 equiv) following **general procedure A**. Column chromatography (SiO_2, 5–10% EtOAc:hexanes) afforded **4o** as an yellow oil (53 mg, 72%).

R_f = 0.32 (5:95 EtOAc:hexanes)

^{1}H NMR ($CDCl_3$, 400 MHz) δ: 5.36 (td, J = 6.3, 1.1 Hz, 1 H), 4.19 (d, J = 6.3 Hz, 2 H), 2.93 (dd, J = 7.2, 6.2 Hz, 1 H), 2.23–2.13 (m, 2 H), 1.76–1.71 (m, 2 H), 1.66–1.62 (m, 6 H), 1.37 (s, 3 H), 0.90 (s, 9 H), 0.07 (s, 6 H) ppm.

^{13}C NMR ($CDCl_3$, 101 MHz) δ: 134.9, 126.2, 60.2, 56.0, 53.7, 36.5, 26.1, 25.8, 21.3, 20.5, 18.56, 16.4, -4.99 ppm.

^{19}F NMR ($CDCl_3$, 376 MHz) δ: 54.4 ppm.

HRMS(-ESI) calc'd for $C_{16}H_{32}FNO_3SSi$ $[M-H]^-$ 364.1772, found 364.1785.

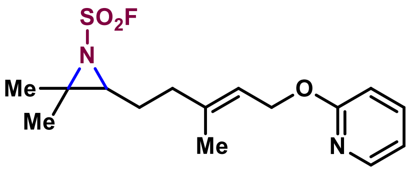

4p

4p was prepared from **3p** (93 mg, 0.4 mmol, 2.0 equiv) following **general procedure A**. Column chromatography (SiO_2, 5–10% EtOAc:hexanes) afforded **4p** as a yellow oil (28 mg, 42%).

R_f = 0.2 (5:95 EtOAc:hexanes)

^{1}H NMR ($CDCl_3$, 400 MHz) δ: 8.15 (dd, J = 5.2, 1.9 Hz, 1 H), 7.59–7.53 (m, 1 H), 6.88–6.83 (m, 1 H), 6.73 (d, J = 8.3 Hz, 1 H), 5.58 (t, J = 8.0 Hz, 1 H), 4.85 (d, J = 6.9 Hz, 2 H), 2.92 (dd, J = 7.3, 6.1 Hz, 1 H), 2.33–2.18 (m, 2 H), 1.81 – 1.73 (m, 5 H), 1.61 (d, J = 2.3 Hz, 3 H), 1.37 (s, 3 H) ppm.

^{13}C NMR ($CDCl_3$, 101 MHz) δ: 163.8, 147.0, 139.0, 138.7, 121.5, 116.8, 111.4, 62.5, 55.9, 53.7, 36.6, 25.8, 21.2, 20.5, 16.7 ppm.

^{19}F NMR ($CDCl_3$, 376 MHz) δ: 54.5 ppm.

HRMS(+ESI) calc'd for $C_{15}H_{21}FN_2O_3S$ $[M+H]^+$ 329.1330, found 329.1322.

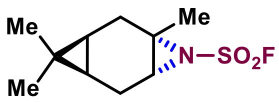

4q

4q was prepared from 3-(*S*)-Carene **3q** (40 mg, 0.3 mmol, 1.5 equiv) following **general procedure A**. Column chromatography (SiO_2, 3:97 EtOAc:hexanes) afforded **3q** as a waxy solid (45 mg, 98%).

R_f = 0.50 (5:95 EtOAc:hexanes)

^{1}H NMR ($CDCl_3$, 400 MHz) δ: 3.06 (t, J = 2.2 Hz, 1 H), 2.45–2.30 (m, 2 H), 1.59–1.53 (m, 4 H), 1.38–1.30 (m, 1 H), 1.03 (s, 3 H), 0.72 (s, 3 H), 0.57 (dtd, J = 29.4, 9.3, 3.3 Hz, 2 H) ppm.

^{13}C NMR ($CDCl_3$, 101 MHz) δ: 50.7, 49.9, 27.6, 24.7, 19.4, 17.9, 16.8, 15.9, 15.2, 13.6 ppm.

^{19}F NMR ($CDCl_3$, 376 MHz) δ: 55.5 ppm.

HRMS(-ESI) calc'd for $C_{10}H_{16}FNO_2S$ $[M-H]^-$ 232.0802, found 232.0801.

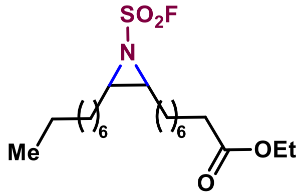

cis-**4r**

cis-**4r** was prepared from ethyl oleate **3r** (93 mg, 0.4 mmol, 1.5 equiv) following **general procedure A**. Column chromatography (SiO_2, 3:97 EtOAc:hexanes) afforded *cis*-**4r** as a colorless oil (60 mg, 76%).

R_f = 0.35 (5:95 EtOAc:hexanes)

^{1}H NMR ($CDCl_3$, 400 MHz) δ: 4.12 (q, *J* = 7.1 Hz, 2 H), 3.02–2.98 (m, 2 H), 2.29 (t, *J* = 7.5 Hz, 2 H), 1.70–1.43 (m, 10 H), 1.42–1.21 (m, 19 H), 0.93–0.84 (m, 3 H) ppm.

^{13}C NMR ($CDCl_3$, 101 MHz) δ: 174.0, 60.3, 49.1, 49.0, 34.4, 31.9, 29.5, 29.3, 29.1, 29.1, 29.0, 27.1, 27.0, 26.6, 26.5, 25.0, 22.8, 14.4, 14.2 ppm.

^{19}F NMR ($CDCl_3$, 376 MHz) δ: 37.2 ppm.

HRMS(-ESI) calc'd for $C_{19}H_{36}FNO_4S$ $[M-H]^-$ 406.2422, found 406.2422.

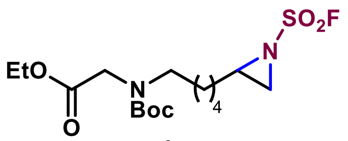

4s

4s was prepared from **3s** (119 mg, 0.4 mmol, 2.0 equiv) following **general procedure A**. Column chromatography (SiO_2, 5–20% EtOAc/hexanes) afforded **4s** as a colorless oil (32 mg, 45%).

R_f = 0.13 (15:85 EtOAc:hexanes)

^{1}H NMR (400 MHz, $CDCl_3$) δ: 4.19 (q, *J* = 7.0 Hz, 2 H), 3.92 (s, 1 H), 3.83 (s, 1 H), 3.26 (dt, *J* = 14.4, 7.2 Hz, 2 H), 3.00 – 2.89 (m, 1 H), 2.82 (t, *J* = 6.1 Hz, 1 H), 2.44 (d, *J* = 4.7 Hz, 1 H), 1.78 – 1.61 (m, 2 H), 1.58 – 1.49 (m, 4 H), 1.46 (s, 3 H), 1.41 (s, 6 H), 1.39 – 1.32 (m, 2 H), 1.31 – 1.25 (m, 3 H) ppm.

13C NMR (101 MHz, CDCl3) δ 170.4, 156.0, 155.4, 80.3, 61.2, 49.6, 49.0, 48.5, 48.2, 43.8, 36.7, 31.0, 28.5, 28.4, 28.0, 27.1, 26.2, 14.4 ppm.

^{19}F NMR ($CDCl_3$, 376 MHz) δ: 36.7 ppm.

HRMS(+ESI) calc'd for $C_{13}H_{25}FN_2O_6S$ $[M+H]^+$ 397.1803, found 397.1794.

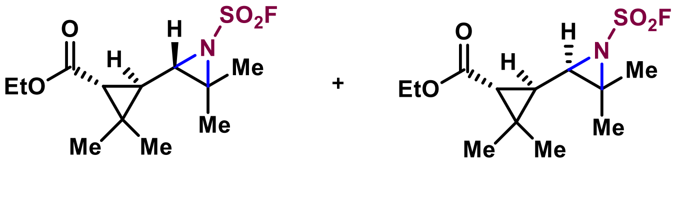

4ta **4tb**

4t was prepared from *trans*-ethyl chrysanthemate **3t** (78 mg, 0.4 mmol, 2.0 equiv) following **general procedure A**. Column chromatography (SiO_2, 5–15% EtOAc/hexanes) afforded **4t** as an inseparable mixture of two diastereomers **4ta:4tb** = **3:1** (combined yield of 47 mg, 82%).

R_f = 0.17 (5:95 EtOAc:hexanes)

^{1}H NMR ($CDCl_3$, 400 MHz) δ: 4.20–4.07 (m, 2 H), 2.95 (d, *J* = 4.0 Hz, 0.3 H), 2.69 (d, *J* = 8.8 Hz, 0.7 H), 1.69–1.59 (m, 4 H), 1.52–1.46 (m, 2 H), 1.46–1.34 (m, 2 H), 1.33 (s, 2 H), 1.30–1.23 (m, 7 H) ppm.

^{13}C NMR ($CDCl_3$, 101 MHz) δ: 171.2, 171.0, 60.9, 60.9, 55.7, 53.7, 53.4, 53.2, 32.8, 32.3, 28.6, 28.4, 27.4, 26.9, 22.0, 21.8, 21.1, 21.0, 21.0, 20.9, 20.3, 20.2, 14.4 ppm.

^{19}F NMR ($CDCl_3$, 376 MHz) δ: 55.9 ppm.

HRMS(-ESI) calc'd for $C_{12}H_{20}FNO_4S$ $[M–H]^-$ 292.1023, found 292.1026.

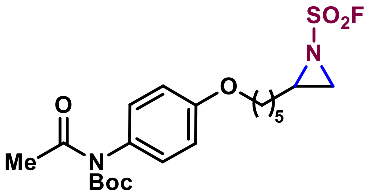

4u

4u was prepared from **3u** (139 mg, 0.4 mmol, 2.0 equiv) following **general procedure A**. Column chromatography (SiO_2, 5-20% EtOAc/hexanes) afforded **4u** as a colorless oil (37 mg, 41%).

R_f = 0.21 (30:70 EtOAc:hexanes)

^1H NMR ($CDCl_3$, 400 MHz) δ: 6.98 (d, J = 8.9 Hz, 2 H), 6.88 (d, J = 8.9 Hz, 2 H), 3.96 (t, J = 6.3 Hz, 2 H), 3.02–2.92 (m, 1 H), 2.84 (d, J = 7.0 Hz, 1 H), 2.54 (s, 3 H), 2.45 (d, J = 4.9 Hz, 1 H), 1.85–1.77 (m, 3H), 1.58–1.51 (m, 5H), 1.39 (s, 9H) ppm.

^{13}C NMR ($CDCl_3$, 101 MHz) δ: 173.3, 158.4, 153.2, 131.7, 129.2, 114.8, 83.2, 67.8, 43.8, 36.7, 31.0, 29.1, 28.0, 26.6, 26.3, 25.7 ppm.

^{19}F NMR ($CDCl_3$, 376 MHz) δ: 36.7 ppm.

HRMS(-ESI) calc'd for $C_{19}H_{27}FN_2O_6S$ $[M-H]^-$ 443.1647, found 443.1640.

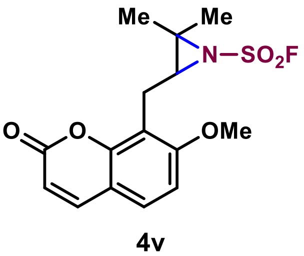

4v

4v was prepared from osthole **3v** (98 mg mg, 0.4 mmol, 2.0 equiv) following **general procedure A**. Column chromatography (SiO_2, 5–20% EtOAc/hexanes) afforded **4v** as a yellow oil (33 mg, 48%).

R_f = 0.16 (25:75 EtOAc:hexanes)

^1H NMR ($CDCl_3$, 400 MHz) δ: 7.65 (d, J = 9.5 Hz, 1 H), 7.40 (d, J = 8.6 Hz, 1 H), 6.89 (d, J = 8.6 Hz, 1 H), 6.26 (d, J = 9.5 Hz, 1 H), 3.95 (s, 3 H), 3.23–3.25 (m, 1 H), 3.14–3.19 (m, 2 H), 1.63 (d, J = 2.0 Hz, 3 H), 1.58 (s, 3 H) ppm.

^{13}C NMR ($CDCl_3$, 101 MHz) δ: 161.0, 160.7, 153.3, 143.9, 128.0, 113.3, 113.1, 113.0, 107.6, 56.3, 55.0, 54.5, 21.2, 21.1, 20.9 ppm.

^{19}F NMR ($CDCl_3$, 376 MHz) δ: 54.4 ppm.

HRMS(-ESI) calc'd for $C_{15}H_{16}FNO_5S$ $[M-H]^-$ 340.0649, found 340.0661.

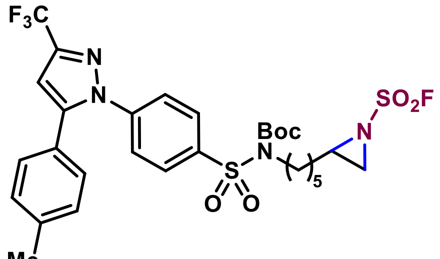

4w

4w was prepared from **3w** (230 mg, 0.4 mmol, 2.0 equiv) following **general procedure A**. Column chromatography (SiO_2, 5–20% EtOAc/hexanes) afforded **4w** as a white crystalline solid (82 mg, 61%).

R_f = 0.13 (15:85 EtOAc:hexanes)

^{1}H NMR ($CDCl_3$, 400 MHz) δ: 7.87 (d, J = 8.7 Hz, 2 H), 7.47 (d, J = 8.7 Hz, 2 H), 7.16 – 7.10 (m, 4 H), 6.73 (s, 1 H), 3.81 (m, 2 H), 2.99–2.90 (m, 1 H), 2.82 (d, J = 6.9 Hz, 1 H), 2.45 (d, J = 4.9 Hz, 1 H), 2.37 (s, 3 H), 1.80–1.69 (m, 3 H), 1.60–1.49 (m, 3 H), 1.47–1.38 (m, 2 H), 1.35 (s, 9 H) ppm.

^{13}C NMR ($CDCl_3$, 101 MHz) δ: 150.77, 145.39, 144.22 (q, J = 38.5 Hz), 142.97, 139.93, 139.65, 129.88, 128.92, 128.84, 125.85, 125.07, 122.49, 119.81, 106.60, 84.79, 47.21, 43.70, 36.73, 30.87, 29.95, 28.00, 26.01, 25.97, 21.42, ppm.

^{19}F NMR ($CDCl_3$, 376 MHz) δ: 36.5, -62.4 ppm.

HRMS(+ESI) calc'd for $C_{29}H_{34}F_4N_4O_6S_2$ $[M+H]^+$ 675.1929, found 675.1925.

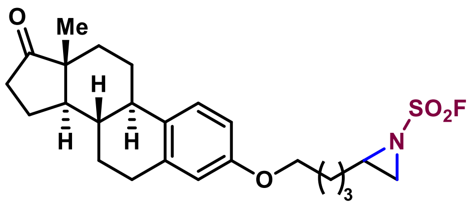

4x

4x was prepared from **3x** (140 mg, 0.4 mmol, 2.0 equiv) following **general procedure A**. Column chromatography (SiO_2, 5-20% EtOAc/hexanes) afforded **4x** as an inseparable mixture of diastereomers (42 mg, 50%).

R_f = 0.15 (15:85 EtOAc:hexanes)

^{1}H NMR ($CDCl_3$, 400 MHz) δ: 7.20 (d, J = 8.8 Hz, 1 H), 6.70 (dd, J = 8.6, 2.9 Hz, 1 H), 6.64 (d, J = 2.9 Hz, 1 H), 3.96 (t, J = 6.1 Hz, 2 H), 3.04 – 2.94 (m, 1 H), 2.93 – 2.87 (m, 2 H), 2.84 (d, J = 7.0 Hz, 1 H), 2.57 – 2.43 (m, 2 H), 2.43 – 2.34 (m, 1 H), 2.30 – 2.21 (m, 1 H), 2.20 – 2.10 (m, 1 H), 2.08 – 1.93 (m, 3 H), 1.89 – 1.75 (m, 3 H), 1.74 – 1.35 (m, 9 H), 0.91 (s, 3 H) ppm.

^{13}C NMR ($CDCl_3$, 101 MHz) δ: 221.4, 157.0, 137.9, 132.2, 126.5, 114.6, 112.2, 67.4, 50.5, 48.2, 44.1, 43.7, 38.5, 36.7, 36.0, 31.7, 30.8, 29.8, 28.7, 26.7, 26.0, 23.3, 21.7, 14.0 ppm.

^{19}F NMR ($CDCl_3$, 376 MHz) δ: 36.7 ppm.

HRMS(–ESI) calc'd for C24H32FNO4S $[M–H]^-$ 448.1952, found 448.1961.

Synthetic applications

SuFEx with aziridines: **General procedure B**

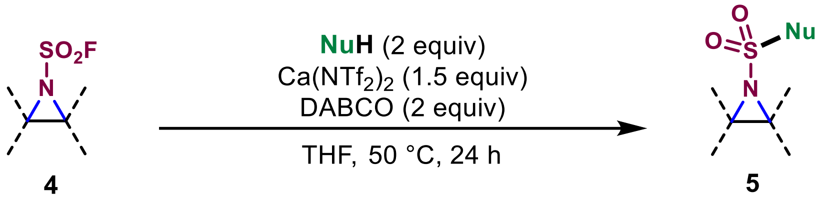

A flame-dried 1 dram vial equipped with a stir bar was charged with the desired aziridne **4** (1 equiv), $Ca(NTf_2)_2$ (1.5 equiv) and DABCO (2 equiv). The reaction vial was evacuated under high vacuum for 10 min and backfilled with argon. Then, the desired nucleophilic click partner (NuH, 2 equiv) was added followed by dry THF (C = 0.5 M). The reaction setup was stirred at 50 °C for 24 h. Upon completion, the mixture was cooled to room temperature, diluted with DCM (5 mL) and filtered through a fritted funnel equipped with celite® (~1–1.5 cm), which was subsequently eluted with additional DCM (20 mL). The filtrate was concentrated *in vacuo* and the crude was purified via silica gel chromatography.

Synthesis and characterization of SuFEx products **5**

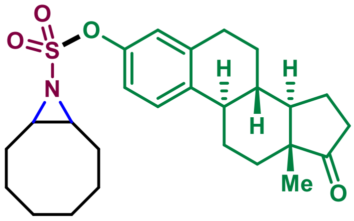

5a

5a was synthesized from aziridine **4h** (30 mg, 0.15 mmol, 1 equiv) and estrone (81 mg, 0.3 mmol, 2 equiv) as the nucleophile following **general procedure B**. Column chromatography (SiO_2, 1–2% MeOH/DCM) afforded **5a** as a white solid (60 mg, 88%).

R_f = 0.60 (3:97 MeOH:DCM)

^1H NMR ($CDCl_3$, 400 MHz) δ: 7.28 (d, J = 8.6 Hz, 1 H), 7.08 (d, J = 8.6 Hz, 1 H), 7.05 (s, 1 H), 2.92 (dd, J = 8.7, 4.0 Hz, 2 H), 2.88 – 2.81 (m, 2 H), 2.50 (dd, J = 18.7, 8.7 Hz, 1 H), 2.43 – 2.35 (m, 1 H), 2.23 – 2.23 (m, 1 H), 2.22 – 1.94 (m, 6 H), 1.72 – 1.28 (m, 16 H), 0.90 (s, 3 H) ppm.

^{13}C NMR ($CDCl_3$, 101 MHz) δ: 220.71, 148.39, 138.90, 138.53, 126.70, 122.06, 119.13, 50.50, 47.99, 45.84, 45.81, 44.23, 37.99, 35.93, 31.62, 29.50, 26.31, 26.24, 25.83, 25.21, 21.67, 13.91 ppm.

HRMS(+ESI) calc'd for $C_{26}H_{35}NO_4S$ $[M+H]^+$ 458.2360, found 458.2342.

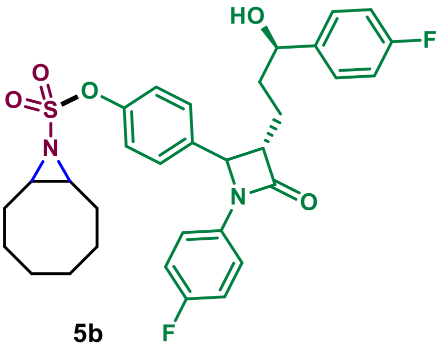

5b

5b was synthesized from aziridine **4h** (32 mg, 0.15 mmol, 1 equiv) and ezetimibe (123 mg, 0.3 mmol, 2 equiv) as the nucleophile following **general procedure B**. Column chromatography (SiO_2, 5–25% EtOAc/hexanes) afforded **5b** as a white solid (60 mg, 67%).

R_f = 0.46 (30:70 EtOAc:hexanes)

^{1}H NMR ($CDCl_3$, 400 MHz) δ: 7.47 (d, J = 8.5 Hz, 2 H), 7.38 (dd, J = 8.4, 5.6 Hz, 2 H), 7.29 (d, J = 8.5 Hz, 2 H), 7.11 (dd, J = 8.8, 4.7 Hz, 2 H), 7.04 (t, J = 8.6 Hz, 2 H), 6.92 (t, J = 8.6 Hz, 2 H), 6.62 (s, 1 H), 4.80 (d, J = 8.1 Hz, 1H), 4.64 (d, J = 12.2 Hz, 1H), 2.90–2.79 (m, 2 H), 2.43–2.30 (m, 2 H), 2.26–2.18 (m, 1 H), 2.17–2.04 (m, 3 H), 1.78–1.66 (m, 1 H), 1.65–1.35 (m, 9 H) ppm.

^{13}C NMR ($CDCl_3$, 101 MHz) δ: 170.7, 163.6, 161.1, 161.0, 158.6, 150.2, 140.0, 138.1, 132.9, 128.0, 127.6, 127.5, 122.7, 122.6, 122.2, 115.8, 115.6, 115.5, 115.2, 81.4, 79.4, 53.3, 46.0, 32.6, 27.8, 26.3, 26.2, 25.2

^{19}F NMR ($CDCl_3$, 377 MHz) δ: -114.9, -117.3.

HRMS(+ESI) calc'd for $C_{32}H_{34}F_2N_2O_5S$ $[M+H]^+$ 597.2229, found 597.2227.

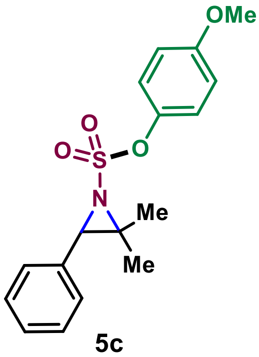

5c

5c was synthesized from aziridine **4f** (48 mg, 0.2 mmol, 1 equiv) and p-hydroxyanisole (50 mg, 0.4 mmol, 2 equiv) as the nucleophile following **general procedure B**. Column chromatography (SiO_2, 5–20% EtOAc/hexanes) afforded **5c** as a white solid (37 mg, 56%).

R_f = 0.35 (15:85 EtOAc:hexanes)

^{1}H NMR ($CDCl_3$, 400 MHz) δ: 7.31–7.37 (m, 4 H), 7.24–7.28 (m, 3 H), 6.90–6.81 (m, 2 H), 4.01 (s, 1 H), 3.79 (s, 3 H), 1.79 (s, 3 H), 1.11 (s, 3 H) ppm.

^{13}C NMR ($CDCl_3$, 101 MHz) δ: 158.3, 144.3, 133.6, 128.5, 128.2, 127.5, 122.9, 114.7, 55.8, 55.5, 53.6, 21.1, 20.5 ppm.

HRMS(+ESI) calc'd for $C_{17}H_{19}NO_4S$ $[M+H]^+$ 334.1108, found 334.1109.

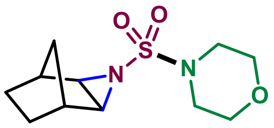
5d

5d was synthesized from aziridine **4j** (30 mg, 0.16 mmol, 1 equiv) and morpholine (28 mg, 0.32 mmol, 2 equiv) as the nucleophile following **general procedure B**. Column chromatography (SiO_2, 5–20% EtOAc/hexanes) afforded **5d** as a waxy solid (18 mg, 44%).

R_f = 0.40 (20:80 EtOAc:hexanes)

^{1}H NMR (400 MHz, $CDCl_3$) δ 3.76 (t, J = 4.0 Hz, 4 H), 3.30 (t, J = 4.0 Hz, 4 H), 2.81 (s, 2 H), 2.51 (s, 2 H), 1.56 – 1.43 (m, 3 H), 1.36 – 1.22 (m, 2 H), 0.81 (d, J = 8.0 Hz, 1 H) ppm.

^{13}C NMR (101 MHz, $CDCl_3$) δ 66.3, 47.0, 41.2, 36.0, 28.4, 25.9 ppm.

HRMS(+ESI) calc'd for $C_{11}H_{18}N_2O_3S$ $[M+H]^+$ 259.1108, found 259.1111.

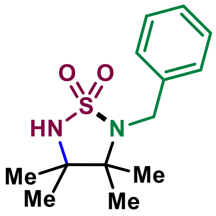
5e

5e was synthesized from aziridine **4m** (25 mg, 0.14 mmol, 1 equiv) and benzyl amine (30 mg, 0.28 mmol, 2 equiv) as the nucleophile following **general procedure B**. Column chromatography (SiO_2, 5–20% EtOAc/hexanes) afforded **5e** as a white solid (19 mg, 53%).

R_f = 0.29 (25:75 EtOAc:hexanes)

^{1}H NMR ($CDCl_3$, 400 MHz) δ: 7.46 (d, J = 7.3 Hz, 2 H), 7.33 (t, J = 7.3 Hz, 2 H), 7.27 (t, J = 7.2 Hz, 1 H), 4.29 (s, 1 H), 4.28 (s, 2 H), 1.33 (s, 6 H), 1.13 (s, 6 H) ppm.

^{13}C NMR ($CDCl_3$, 101 MHz) δ: 137.9, 128.7, 128.1, 127.6, 68.8, 62.9, 44.2, 23.8, 21.0 ppm.

HRMS(+ESI) calc'd for $C_{13}H_{20}N_2O_2S$ $[M+H]^+$ 269.1318, found 269.1316.

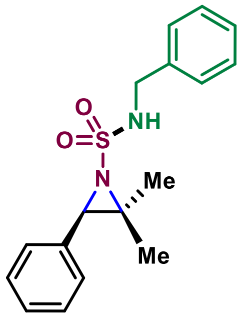
(*R*)-**5f**

(*R*)-**5f** was synthesized from aziridine **4f** (35 mg, 0.15 mmol, 1 equiv) and benzyl amine (32 mg, 0.3 mmol, 2 equiv) as the nucleophile following **general procedure B**. Column chromatography (SiO_2, 5–20% EtOAc/hexanes) afforded (*R*)-**5f** as a white solid (26 mg, 57%).

R_f = 0.23 (15:85 EtOAc:hexanes)

^{1}H NMR ($CDCl_3$, 400 MHz) δ: 7.39 – 7.27 (m, 10 H), 4.62 (t, *J* = 6.0 Hz, 1 H), 4.43 (d, *J* = 6.1 Hz, 2 H), 3.87 (s, 1 H), 1.79 (s, 3 H), 1.10 (s, 3 H) ppm.

^{13}C NMR ($CDCl_3$, 101 MHz) δ: 136.7, 134.6, 128.9, 128.5, 128.3, 128.2, 127.9, 127.3, 53.9, 52.2, 48.2, 20.9, 20.6 ppm.

HRMS(+ESI) calc'd for $C_{17}H_{20}N_2O_2S$ $[M+H]^+$ 317.1318, found 317.1313.

Synthesis and characterization of **6a**

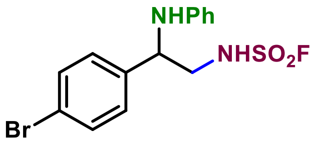

6a

To a solution of aziridine **4b** (18 mg, 0.06 mmol, 1 equiv) in DCM (1 mL), was added aniline (5.5 µL, 0.066 mmol, 1.1 equiv). The reaction mixture was stirred at room temperature for 16 h. Upon completion, the mixture was concentrated *in vacuo,* and the crude mixture was purified via silica chromatography (SiO_2, 10% EtOAc/Hexane). **6a** was obtained as a yellow oil (15 mg, 61%).

R_f = 0.25 (15:85 EtOAc:hexanes)

^{1}H NMR ($CDCl_3$, 400 MHz) δ: 7.51 (d, *J* = 8.5 Hz, 2 H), 7.23 (d, *J* = 8.5 Hz, 2 H), 7.15 (t, *J* = 8.0 Hz, 2 H), 6.78 (t, *J* = 7.3 Hz, 1 H), 6.59 (d, *J* = 8.7 Hz, 2 H), 5.41 (s, 1 H), 4.62 (dd, *J* = 8.2, 4.7 Hz, 1 H), 3.67 (dd, *J* = 13.9, 5.0 Hz, 1 H), 3.54 (dd, *J* = 13.5, 8.2 Hz, 1 H) ppm.

^{13}C NMR ($CDCl_3$, 101 MHz) δ: 145.9, 138.1, 132.6, 129.6, 128.3, 122.5, 119.4, 114.3, 57.1, 49.7 ppm.

^{19}F NMR ($CDCl_3$, 377 MHz) δ: 51.1 ppm.

HRMS(–ESI) calc'd for $C_{14}H_{14}BrFN_2O_2S$ $[M–H]^-$ 370.9860, found 370.9876.

Synthesis and characterization of **6b**

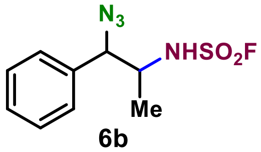

6b

Under argon atmosphere, $TMSN_3$ (5 µL, 0.037 mmol) and TBAF (1 µL, 5 mol%, 1.0 M in THF) were added to a solution of *cis*-**4e** (8.0 mg, 0.037 mmol) in THF (1.0 mL). The mixture was stirred at room temperature for 16 h. Upon completion, the solvent was evaporated and the residues were purified via preparatory TLC (SiO_2, 15% EtOAc:Hexanes) to give product **6b** as a colourless oil (8 mg, 80%).

R_f = 0.33 (15:85 EtOAc:hexanes)

^{1}H NMR ($CDCl_3$, 400 MHz) δ: 7.46 – 7.35 (m, 3 H), 7.31 (dd, *J* = 7.9, 1.7 Hz, 2 H), 5.73 (s, 1 H), 4.49 (dd, *J* = 7.9, 4.5 Hz, 1 H), 3.88 (qd, *J* = 6.6, 4.5 Hz, 1 H), 1.42 (d, *J* = 6.6 Hz, 3 H) ppm.

^{13}C NMR ($CDCl_3$, 101 MHz) δ: 137.2, 129.2, 129.1, 126.8, 63.1, 61.8, 17.4 ppm.

^{19}F NMR ($CDCl_3$, 377 MHz) δ: 54.1 ppm.

HRMS(–ESI) calc'd for $C_9H_{11}FN_4O_2S$ $[M–H]^-$ 257.0503, found 257.0510.

Synthesis and characterization of **6c**

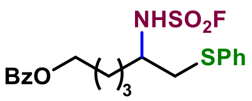

6c

A flame-dried 1 dram vial equipped with a stir bar was charged with aziridine **4l** (30mg, 0.1 mmol, 1 equiv), thiophenol (12 µL, 0.11 mmol, 1.1 equiv) and MeCN (500 µL). The reaction mixture was stirred overnight at room temperature. Upon completion, as monitored by TLC, the solvent was evaporated and the residues were purified directly via column chromatography (SiO_2, 5–20% EtOAc/Hexanes) to afford **6c** as a colorless oil (34 mg, 83%).

R_f = 0.23 (15:85 EtOAc:hexanes)

^1H NMR ($CDCl_3$, 400 MHz) δ: 8.03 (dd, J = 8.3, 1.4 Hz, 2 H), 7.57 (t, J = 7.5 Hz, 1 H), 7.48 – 7.36 (m, 4 H), 7.31 (t, J = 7.4 Hz, 2 H), 7.26 – 7.21 (m, 1 H), 5.55 (s, 1 H), 4.31 (dtd, J = 17.3, 10.9, 6.1 Hz, 2 H), 3.82 – 3.65 (m, 1 H), 3.31 – 3.05 (m, 2 H), 1.90 – 1.62 (m, 4 H), 1.61 – 1.37 (m, 2 H) ppm.

^{13}C NMR ($CDCl_3$, 101 MHz) δ: 167.1, 134.6, 133.2, 130.5, 130.2, 129.7, 129.5, 128.6, 127.4, 64.3, 55.6, 39.4, 33.0, 28.4, 22.0 ppm.

^{19}F NMR ($CDCl_3$, 377 MHz) δ: 54.5 ppm.

HRMS(–ESI) calc'd for $C_{19}H_{22}FNO_4S_2$ $[M–H]^-$ 410.0891, found 410.0898.

Synthesis and characterization of **6d**

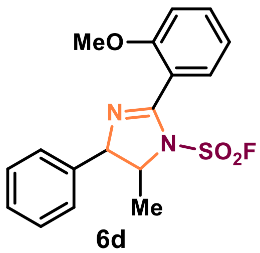

6d

To a flame-dried 1 dram vial equipped with a stir bar was added aziridine *cis*-**4e** (5 mg, 0.023 mmol, 1 equiv). The vial was evacuated and backfilled with argon three times. Dry dichloromethane (150 µL) and 2-methoxybenzonitrile (2.81 µL, 0.023 mmol, 1.0 equiv) were added under an argon atmosphere. The reaction mixture was cooled to –78 °C using a dry ice–acetone bath, and $BF_3 \cdot OEt_2$ (4.3 µL, 0.034 mmol, 1.5 equiv) was added. The cooling bath was then removed, and the mixture was allowed to warm gradually to room temperature, stirring overnight. Upon completion, as monitored by TLC, the solvent was removed under reduced pressure and the crude residue purified by preparative TLC (SiO_2, 5% EtOAc/hexanes) to afford **6d** as a waxy solid (4 mg, 50%).

R_f = 0.23 (15:85 EtOAc:hexanes)

^1H NMR ($CDCl_3$, 400 MHz) δ: 7.52 – 7.45 (m, 2 H), 7.44 – 7.39 (m, 2 H), 7.38 – 7.30 (m, 3 H), 7.03 (t, J = 7.5 Hz, 1 H), 6.96 (d, J = 8.5 Hz, 1 H), 4.92 (d, J = 4.3 Hz, 1 H), 4.37 (p, J = 6.0 Hz, 1 H), 3.90 (s, 3 H), 1.68 (d, J = 6.4 Hz, 3 H) ppm.

^{13}C NMR (126 MHz, $CDCl_3$) δ 158.2, 140.4, 132.6, 130.5, 129.6, 129.2, 128.4, 126.4, 120.6, 118.7, 111.0, 76.6, 66.2, 55.7, 21.1 ppm.

^{19}F NMR ($CDCl_3$, 377 MHz) δ: 54.2 ppm.

HRMS(+ESI) calc'd for $C_{17}H_{17}FN_2O_3S$ $[M+H]^+$ 349.1017, found 349.1007.

Synthesis and characterization of **6e**

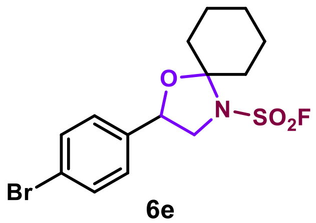

6e

To a flame-dried 1 dram vial equipped with a stir bar was added aziridine **4b** (5 mg, 0.018 mmol, 1 equiv). The vial was evacuated and backfilled with argon three times. Dry dichloromethane (150 µL) was added under an argon atmosphere. The reaction mixture was cooled to –78 °C using a dry ice–acetone bath, and cyclohexanone (2.1 mg, 0.021 mmol, 1.2 equiv) was added followed by BF_3•OEt_2 (3.3 µL, 0.027 mmol, 1.5 equiv). The cooling bath was then removed, and the mixture was allowed to warm gradually to room temperature, stirring overnight. Upon completion, as monitored by TLC, the solvent was removed under reduced pressure and the crude residue purified by preparative TLC (SiO_2, 5% EtOAc/hexanes) to afford **6e** as a waxy solid (5 mg, 72%).

R_f = 0.6 (15:85 EtOAc:hexanes)

^{1}H NMR ($CDCl_3$, 400 MHz) δ: 7.54 (d, J = 8.4 Hz, 2 H), 7.28 (d, J = 8.6 Hz, 2 H), 5.12 (dd, J = 9.8, 5.7 Hz, 1 H), 4.02 (ddd, J = 9.1, 5.7, 1.2 Hz, 1 H), 3.38 (td, J = 9.5, 3.9 Hz, 1 H), 2.24 (td, J = 13.2, 4.8 Hz, 1 H), 2.08 – 1.94 (m, 2 H), 1.81 – 1.53 (m, 6 H), 1.30 – 1.18 (m, 1 H) ppm.

^{13}C NMR ($CDCl_3$, 101 MHz) δ: 135.5, 132.1, 128.1, 123.1, 100.3, 75.6, 54.6, 35.6, 33.4, 24.5, 23.5 ppm.

^{19}F NMR ($CDCl_3$, 377 MHz) δ: 49.9 ppm.

HRMS(–ESI) calc'd for $C_{14}H_{17}BrFNO_3S$ $[M–H]^-$ 376.0013, found 376.0018.

Synthesis and characterization of **6f**

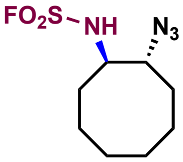

6f

To a flame-dried 1 dram vial equipped with a stir bar was added **4h** (50 mg, 0.24 mmol, 1 equiv), NaN_3 (31.2 mg, 0.48 mmol, 2 equiv) and DMF (300 µL). The mixture was stirred for 14 h at room temperature. Upon completion of the reaction, as monitored by TLC, the mixture was diluted with EtOAc (10 mL), washed with water (3 x 10 mL) and extracted with EtOAc (3 x 15 mL). The combined organic fraction was dried over Na_2SO_4 and evaporated to obtain the crude mixture. Purification of the crude via column chromatography (SiO_2, 5–20% EtOAc/Hexanes) afforded **6f** as a colourless oil (29 mg, 48%).

R_f = 0.70 (3% MeOH:DCM)

^{1}H NMR ($CDCl_3$, 400 MHz) δ: 5.32 (s, 1 H), 3.66 – 3.56 (m, 1 H), 3.54 – 3.44 (m, 1 H), 2.10 – 1.97 (m, 2 H), 1.96 – 1.76 (m, 3 H), 1.75 – 1.55 (m, 5 H), 1.54 – 1.35 (m, 2 H) ppm.

^{13}C NMR ($CDCl_3$, 101 MHz) δ: 65.20, 60.01, 30.19, 29.05, 25.74, 25.55, 24.54, 24.46 ppm.

^{19}F NMR ($CDCl_3$, 377 MHz) δ: 53.6 ppm.

HRMS(–ESI) calc'd for $C_8H_{15}FN_4O_2S$ $[M–H]^-$ 249.0816, found 249.0826.

Synthesis and characterization of **6g**

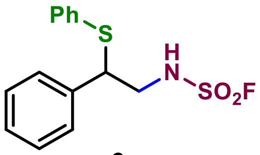
6g

6g was synthesized from aziridine **4a. 4a** was first synthesized from styrene **3a** (45 mg, 0.3 mmol, 1.5 equiv) and **1** (0.2 mmol) following **general procedure A**. Crude NMR analysis (^{1}H and ^{19}F) confirmed product formation. Yield = 0.18 mmol (90%) determined by ^{1}H NMR analysis using 1,2,4,5-tetramethyl benzene as internal standard. The crude was concentrated under rotavap, then dissolved in 1 mL DCM and thiophenol (30 mg, 0.27 mmol, 1.5 equiv) was added. The reaction mixture was stirred at room temperature for 2 h. Upon completion, as monitored by TLC, the reaction mixture was evaporated to obtain the crude ring-opened product. Purification via column chromatography (SiO_2, 5–10% EtOAc/Hexanes) afforded **6g** as a yellow oil (45 mg, 81%).

R_f = 0.37 (20% EtOAc/Hexanes)

^{1}H NMR (400 MHz, $CDCl_3$) δ 7.32 – 7.16 (m, 10 H), 5.15 (s, 1 H), 4.29 (t, J = 7.6 Hz, 1 H), 3.74 – 3.56 (m, 2 H) ppm.

^{13}C NMR (126 MHz, $CDCl_3$) δ 137.6, 133.2, 132.4, 129.4, 129.3, 128.7, 128.5, 128.0, 52.5, 48.4 ppm.

^{19}F NMR (376 MHz, $CDCl_3$) δ: 52.3 ppm

HRMS(-ESI) calc'd for $C_{14}H_{14}FNO_2S_2$ $[M-H]^-$ 310.0366, found 310.0376.

Synthesis and characterization of **7a**

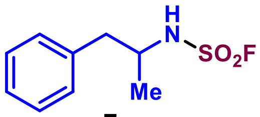
7a

To a flame-dried 1 dram vial equipped with a stir bar was added aziridine *cis*-**4e** (10 mg, 0.046 mmol, 1 equiv), Pd/C (10 wt%, 10 mg) and dry methanol (500 µL). The suspension was then purged with H_2 gas for 15 minutes. The reaction mixture under H_2 atmosphere was then allowed to stir overnight for 16 h. Upon completion, as monitored by TLC, the suspension was filtered through celite®, and the filtrate was evaporated to obtain the crude product. Purification of the crude via column chromatography (SiO_2, 5–10% EtOAc/Hexanes) afforded **7a** as a colourless oil (8 mg, 80%).

R_f = 0.25 (10% EtOAc/Hexanes)

^{1}H NMR ($CDCl_3$, 400 MHz) δ: 7.40 – 7.27 (m, 3H), 7.19 (d, J = 6.8 Hz, 2 H), 4.75 (s, 1 H), 3.96 (dq, J = 13.3, 6.7 Hz, 1 H), 2.89 (qd, J = 13.8, 6.4 Hz, 2 H), 1.29 (d, J = 6.8 Hz, 3 H) ppm.

^{13}C NMR (101 MHz, $CDCl_3$) δ: 136.0, 129.6, 129.0, 127.4, 53.0, 42.7, 20.5 ppm.

^{19}F NMR (376 MHz, $CDCl_3$) δ: 54.2 ppm

HRMS(+ESI) calc'd for $C_9H_{12}FNO_2S$ $[M+H]^+$ 216.0489, found 216.0493.

Synthesis and characterization of **7b**

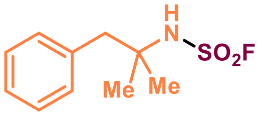

7b

To a flame-dried 1 dram vial equipped with a stir bar was added aziridine **4f** (20 mg, 0.087 mmol, 1 equiv), Pd/C (10 wt%, 20 mg) and dry methanol (500 µL). The suspension was then purged with H_2 gas for 15 minutes. The reaction mixture under H_2 atmosphere was then allowed to stir overnight for 16 h. Upon completion, as monitored by TLC, the suspension was filtered through celite®, and the filtrate was evaporated to obtain the crude product. Purification of the crude via column chromatography (SiO_2, 5–10% EtOAc/Hexanes) afforded **7b** as a colourless oil (15 mg, 77%).

R_f = 0.30 (3% MeOH:DCM)

^{1}H NMR ($CDCl_3$, 400 MHz) δ: 7.38 – 7.29 (m, 3 H), 7.21 (d, J = 6.6 Hz, 2 H), 4.72 (s, 1 H), 2.93 (s, 2 H), 1.42 (s, 6 H) ppm.

^{13}C NMR ($CDCl_3$, 101 MHz) δ: 135.7, 130.7, 128.8, 127.4, 58.8, 47.7, 27.3 ppm.

^{19}F NMR ($CDCl_3$, 377 MHz) δ: 59.7 ppm.

HRMS(–ESI) calc'd for $C_{10}H_{14}FNO_2S$ $[M–H]^-$ 230.0646, found 230.0652.

Synthesis and characterization of **8a**

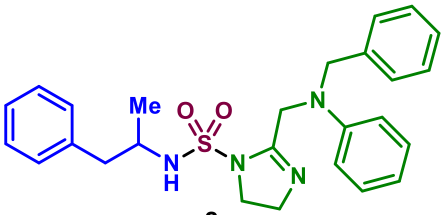

8a

To a flame-dried 1 dram vial equipped with a stir bar was added **7a** (12 mg, 0.055 mmol, 1 equiv), antazoline hydrochloride (21 mg, 0.07 mmol, 1.2 equiv) and MeCN (500 µL). DBU (21 µL, 0.14 mmol, 2 equiv) was added and the reaction was stirred at 50 °C for 4 h. Upon completion, as monitored by TLC, the reaction mixture was evaporated to obtain the crude product. Purification of the crude via column chromatography (SiO_2, 2% MeOH/DCM) afforded **8a** as a yellow oil (20 mg, 72%).

R_f = 0.37 (10% EtOAc/Hexanes)

^{1}H NMR (CD_3CN, 400 MHz) δ: 7.39 – 7.30 (m, 5 H), 7.30 – 7.20 (m, 5 H), 7.13 (dd, J = 8.8, 7.3 Hz, 2H), 6.65 (m, 3 H), 5.78 (d, J = 8.6 Hz, 1 H), 4.59 (s, 2 H), 4.49 – 4.32 (m, 2 H), 3.63 (m, 1 H), 3.57 – 3.40 (m, 3 H), 3.39 – 3.31 (m, 1 H), 2.79–2.77 (m, 2 H), 1.23 (d, J = 4Hz, 3 H) ppm.

^{13}C NMR (CD_3CN, 126 MHz) δ: 156.6, 149.3, 140.1, 139.1, 130.0, 129.4, 129.1, 129.0, 127.4, 127.3, 127.1, 113.1, 55.7, 53.0, 52.9, 49.9, 49.0, 43.4, 21.6 ppm.

HRMS(+ESI) calc'd for $C_{26}H_{30}N_4O_2S$ $[M+H]^+$ 463.2162, found 463.2148.

Synthesis and characterization of **8b**

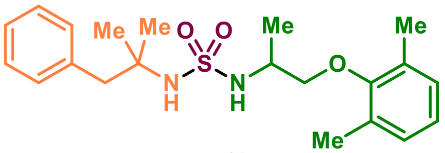

8b

To a flame-dried 1 dram vial equipped with a stir bar was added **7b** (7 mg, 0.03 mmol, 1 equiv), mexilitene hydrochloride (6.5 mg, 0.03 mmol, 1 equiv) and MeCN (500 µL). DBU (9 µL, 0.06 mmol, 2 equiv) was added and the reaction was stirred at 50 °C for 4 h. Upon completion, as monitored by TLC, the reaction mixture was evaporated to obtain the crude product. Purification of the crude via column chromatography (SiO_2, 2% MeOH/DCM) afforded **8b** as a yellow oil (10 mg, 85%).

R_f = 0.75 (3% MeOH:DCM)

^{1}H NMR ($CDCl_3$, 400 MHz) δ: 7.35 – 7.28 (m, 2 H), 7.24 (d, J = 8.1 Hz, 3 H), 4.66 (m, 1 H), 4.08 (s, 1 H), 3.83 – 3.76 (m, 1 H), 3.70 (m, 2 H), 2.97 – 2.84 (m, 2 H), 2.27 (s, 6 H), 1.46 – 1.36 (m, 9 H) ppm.

^{13}C NMR ($CDCl_3$, 101 MHz) δ: 154.9, 136.8, 131.0, 130.9, 129.2, 128.4, 127.0, 124.4, 74.5, 56.6, 50.2, 48.8, 27.3, 18.3, 16.4.

HRMS(+ESI) calc'd for $C_{21}H_{30}N_2O_3S$ $[M+H]^+$ 391.2050, found 391.2039.

Synthesis and characterization of **8c**

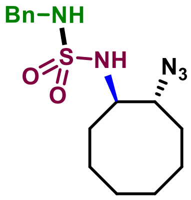

To a flame-dried 1 dram vial equipped with a stir bar was added **6f** (16 mg, 0.064 mmol, 1 equiv), benzylamine (13.7 mg, 0.128 mmol, 1 equiv) and MeCN (500 µL). DBU (14.3 µL, 0.096 mmol, 1.5 equiv) was added and the reaction was stirred at 50 °C for 4 h. Upon completion, as monitored by TLC, the reaction mixture was evaporated to obtain the crude product. Purification of the crude via column chromatography (SiO_2, 2% MeOH/DCM) afforded **8c** as a white solid (18 mg, 84%).

R_f = 0.32 (2% MeOH/DCM)

^{1}H NMR ($CDCl_3$, 400 MHz) δ : 7.42 – 7.29 (m, 5 H), 4.53 (s, 1 H), 4.46 (d, J = 6.6 Hz, 1 H), 4.28 (s, 2 H), 3.49 – 3.34 (m, 2 H), 2.08 – 1.94 (m, 2 H), 1.91 – 1.74 (m, 3 H), 1.71 – 1.52 (m, 5 H), 1.50 – 1.33 (m, 2 H) ppm.

^{13}C NMR ($CDCl_3$, 101 MHz) δ: 136.8, 129.0, 128.3, 128.2, 66.4, 58.2, 47.6, 30.6, 28.9, 25.9, 25.7, 24.8, 24.7 ppm.

HRMS(+ESI) calc'd for $C_{15}H_{23}N_5O_2S$ $[M+H]^+$ 338.1645, found 338.1641.

Synthesis and characterization of **9**

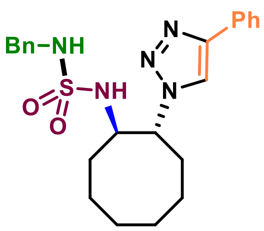

9

To a flame-dried 1 dram vial equipped with a stir bar was added **8c** (22 mg, 0.066 mmol, 1 equiv), phenylacetylene (7 µL, 0.066 mmol, 1 equiv), $CuSO_4 \cdot 5H_2O$ (1 mg, 5 mol%), sodium ascorbate (1.5 mg, 10 mol%), ^{t}BuOH (132 µL), H_2O (132 µL) and DCM (13 µL). The mixture was stirred at room temperature for 8 h. Upon completion, as monitored by TLC, the reaction mixture was evaporated to obtain the crude product. Purification of the crude via column chromatography (SiO_2, 2% MeOH/DCM) afforded **9** as a white solid (25 mg, 86%).

R_f = 0.15 (3% MeOH:DCM)

^{1}H NMR ($CDCl_3$, 400 MHz) δ : 7.91 (s, 1 H), 7.75 (d, J = 8.5 Hz, 2 H), 7.35 (m, 3 H), 7.20 – 7.11 (m, 3 H), 7.10 – 7.00 (m, 2 H), 4.97 (d, J = 9.0 Hz, 1 H), 4.76 – 4.66 (m, 1 H), 4.40 (m, 1 H), 4.13 – 4.01 (m, 1 H), 3.77 – 3.67 (m, 1H), 3.52 (m, 1 H), 2.22–2.10 (m, 4 H), 1.87 – 1.73 (m, 4 H), 1.72 – 1.59 (m, 4 H) ppm.

^{13}C NMR ($CDCl_3$, 126 MHz) δ: 136.4, 130.3, 128.9, 128.5, 128.3, 127.8, 127.7, 125.7, 65.1, 58.2, 46.9, 32.0, 30.6, 26.3, 25.9, 25.2, 23.7 ppm.

HRMS(+ESI) calc'd for $C_{23}H_{29}N_5O_2S$ $[M+H]^+$ 440.2115, found 440.2109.

Table S4: Yield comparison between chiral (*S*)-**Rh-1** and achiral **Rh-3** (Rh_2(tfacam)$_4$)

All reactions carried out according to general procedure **A** by varying the catalyst

Aziridine	Isolated yield with **Rh-3**	Isolated yield with (*S*)-**Rh-1**
**4d**	78	93
trans-**4e**	46	82
cis-**4e**	76	98
**4f**	87	98
**4n**	70	98
**4v**	43	48

Enantioselectivity studies

Synthesis of racemic aziridines

Racemic aziridines were synthesized following **general procedure A** using $Rh_2(tfacam)_4$ (**Rh-3)** as the catalyst.

Enantiomeric excess determination for **4f**

Chiral HPLC was run using an Agilent 1260 Infinity II HPLC and a Daicel Chiralpak® AD-H column that is 4.6 mm I.D. x 250 mm in length and particle size of 5 μm. The compounds were eluted with hexane at a flow rate of 1 mL/min. Detection was done by UV at 220 nm. T_R major (**4f**) = 10.8 min, T_R minor (**4f**) = 12.6 min, ee = 90%.

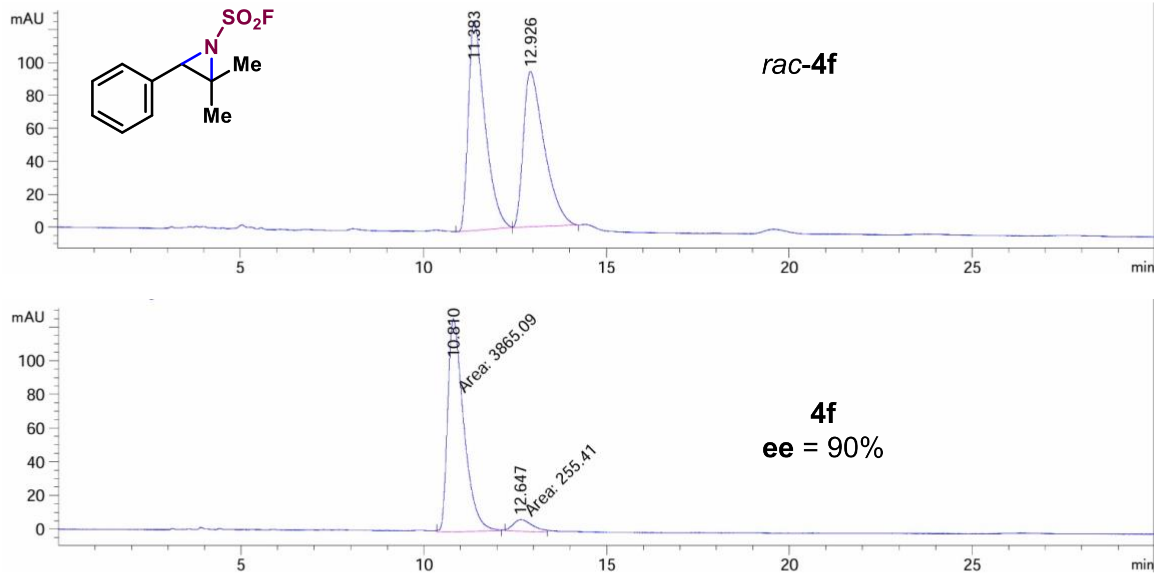

Figure S5. Top HPLC trace for racemic **4f**. Bottom HPLC trace for **4f**.

Enantiomeric excess determination for **4d**

Chiral HPLC was run using an Agilent 1260 Infinity II HPLC and a Daicel Chiralpak® AD-H column that is 4.6 mm I.D. x 250 mm in length and particle size of 5 μm. The compounds were eluted with hexane/isopropanol 95/05 at a flow rate of 0.9 mL/min. Detection was done by UV at 254 nm. T_R major (**4d**) = 9.7 min, T_R minor (**4d**) = 8.6 min, ee = 54%.

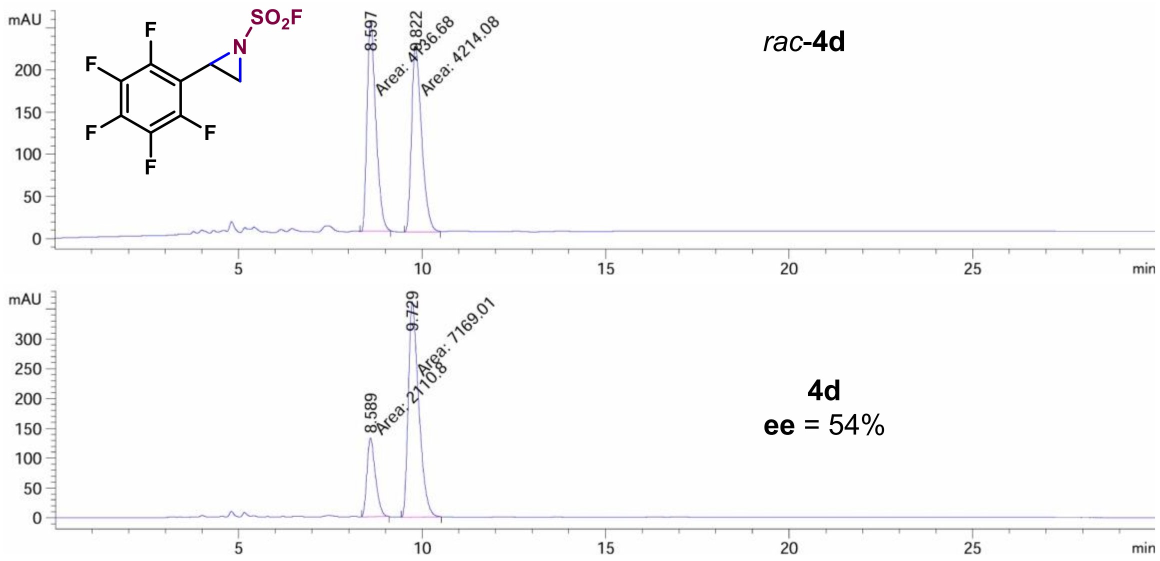

Figure S6. Top HPLC trace for racemic **4f**. Bottom HPLC trace for **4f**.

<u>Enantiomeric excess determination for *cis*-**4e**</u>

Chiral HPLC was run using an Agilent 1260 Infinity II HPLC and a Daicel Chiralpak® AD-H column that is 4.6 mm I.D. x 250 mm in length and particle size of 5 µm. The compounds were eluted with hexane/isopropanol 98/02 at a flow rate of 0.9 mL/min. Detection was done by UV at 220 nm. T_R major (*cis*-**4e**) = 24.1 min, T_R minor (*cis*-**4e**) = 18.2 min., ee = 30%.

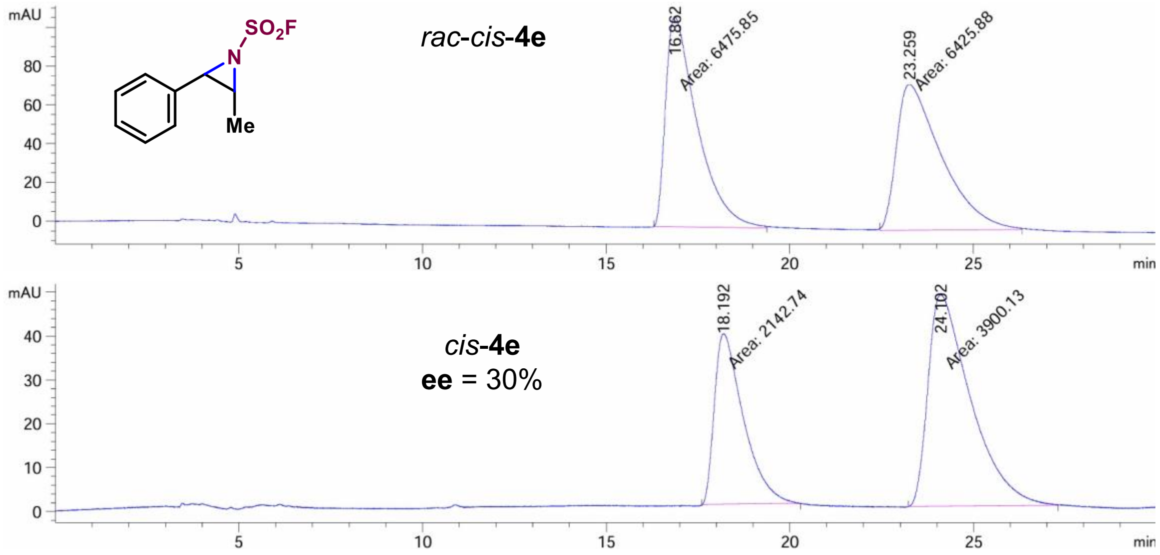

Figure S7. Top HPLC trace for racemic cis-**4e**. Bottom HPLC trace for cis-**4e**.

<u>Enantiomeric excess determination for *trans*-**4e**</u>

Chiral HPLC was run using an Agilent 1260 Infinity II HPLC and a Daicel Chiralpak® AD-H column that is 4.6 mm I.D. x 250 mm in length and particle size of 5 µm. The compounds were eluted with hexane/isopropanol 99.5/0.5 at a flow rate of 0.9 mL/min. Detection was done by UV at 210 nm. T_R major (*trans*-**4e**) = 35.6 min, T_R minor (*trans*-**4e**) = 23.8 min., ee = 52%.

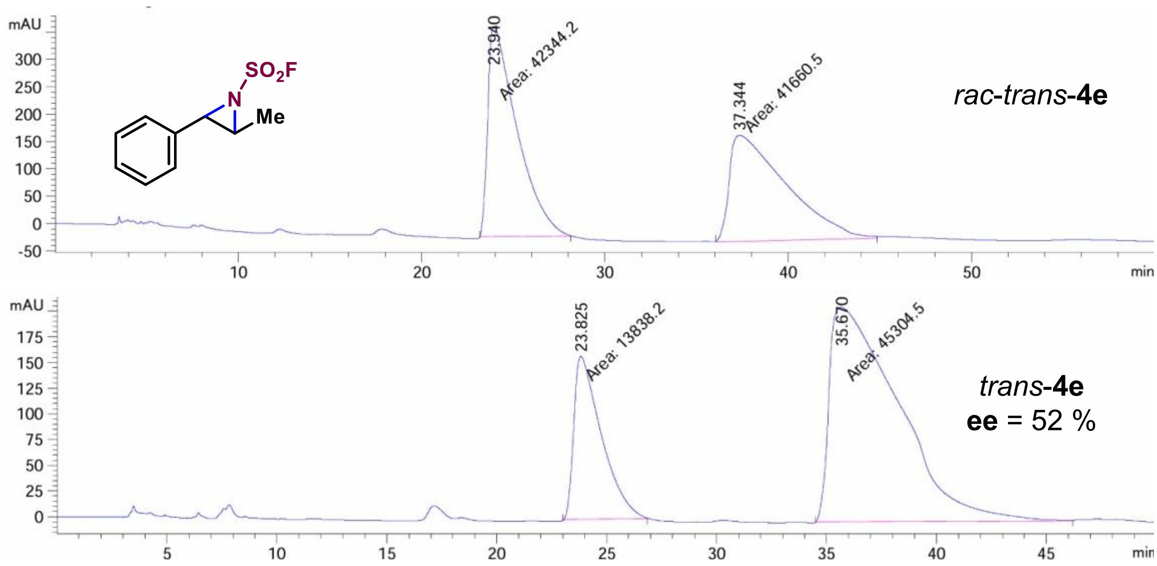

Figure S8. Top HPLC trace for racemic trans-**4e**. Bottom HPLC trace for trans-**4e**.

<u>Enantiomeric excess determination for **4n**</u>

Chiral HPLC was run using an Agilent 1260 Infinity II HPLC and a Daicel Chiralpak® AD-H column that is 4.6 mm I.D. x 250 mm in length and particle size of 5 μm. The compounds were eluted with hexane/isopropanol gradient (1→2% iPrOH over 40 minutes) at a flow rate of 0.9 mL/min. Detection was done by UV at 220 nm. T_R major (**4n**) = 25.2 min, T_R minor (**4n**) = 26.4 min., ee = 20%

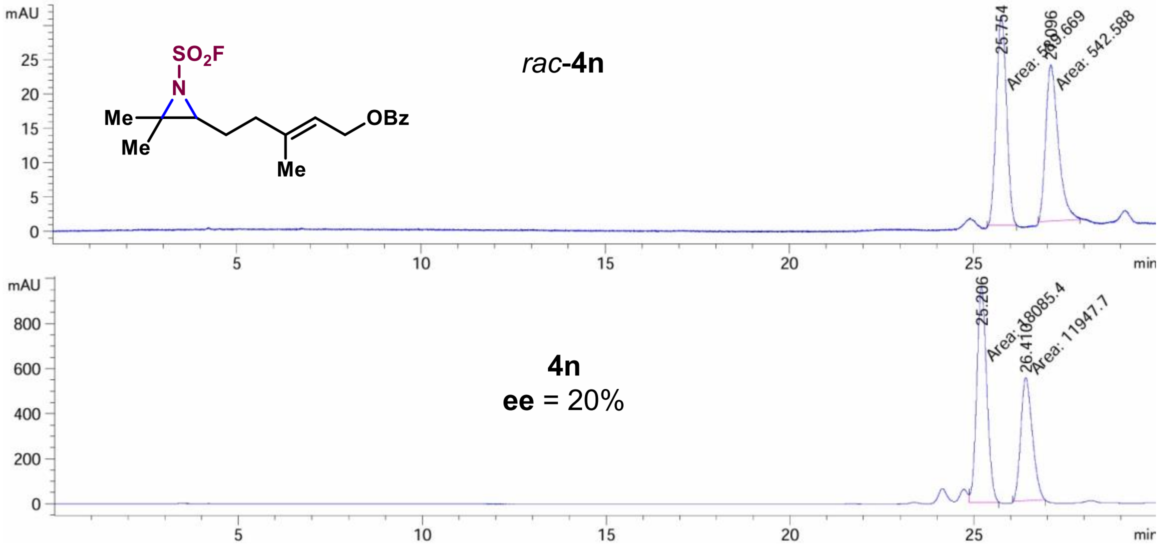

Figure S9. Top HPLC trace for racemic **4n**. Bottom HPLC trace for **4n**.

Table S5. Photodegradation of FSO_2N_3 (**2a**) as a solution in MTBE under various wavelengths

Entry	Wavelength (nm)	Time (h)	Degradation	FSO_2NH_2 (**1**) formed (%)[a]
1	450	48	No degradation	–
2	350	48	No degradation	–
3	254	2	Fully degraded	62

[a] ^{19}F NMR yield determined using TsF as an internal standard.

Table S6. FSO_2N_3 (2a) (solution in MTBE) reduction attempts in various conditions:

Entry	Reducing agent	FSO_2NH_2 (**1**) formed (%)[a]
1	PPh_3	12
2	H_2, Pd/C	24
3	$NaBH_4$ (Reproducibility issues)	0–50
4	$LiAlH_4$	–

[a] ^{19}F NMR yield determined using TsF as an internal standard.

Table S7. Attempts for aziridination of various alkenes using **2a** under direct 254 nm UV irradiation[a]

Entry	Alkene	Alkene equiv	**2a** (equiv)	Aziridine yield (%)
1	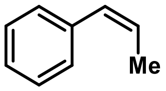	5	1	10
2	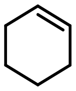	5	1	Trace
3	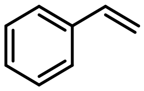	5	1	Trace
4	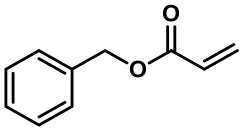	5	1	–
5	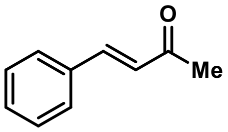	5	1	–

[a]All reactions performed with 0.2 mmol of **2a** as a solution in MTBE. Equal volumes of DCM was used a co-solvent.

Entries 1–3 afforded only trace quantities of the desired aziridines, accompanied by uncharacterizable byproducts. Entry 4 underwent polymerization under 254 nm UV irradiation without detectable aziridine formation. Entry 5 exhibited trans-to-cis isomerization with no evidence of aziridine formation.

Attempts for aziridination of styrene via photosensitized excitation of **2a** under 450 nm blue LEDs

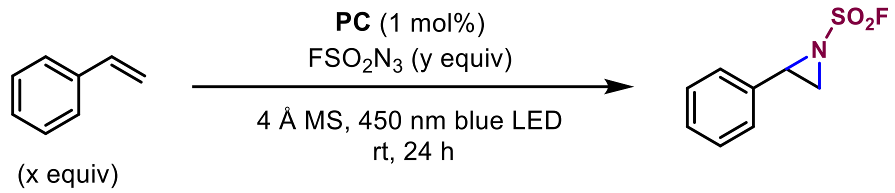

A flame-dried 2-dram vial equipped with a stir bar and 4 Å molecular sieves was charged under an argon atmosphere with the photocatalyst (PC) and alkene. In a separate 2-dram vial, the desired volume of **2a** in MTBE was subjected to five freeze–pump–thaw cycles and transferred to the reaction vial under argon. The reaction vial was then irradiated using blue LEDs (30 W, 450 nm), while the reaction was stirred at 600 rpm. After 24 hours of irradiation, the reaction mixture was diluted with dichloromethane (DCM, 5 mL) and filtered through a fritted funnel containing a Celite® pad (~1–1.5 cm), eluting with additional DCM (20 mL). Solvents were removed under reduced pressure, and the crude residue was analyzed by ^{1}H and ^{19}F NMR spectroscopy.

Table S8. Aziridination attempts with various photocatalysts and co-solvents

Entry	**PC** (1 mol%)	Styrene (equiv)	**2a** (equiv)	NMR yield (%)[a]
1	Eosin Y	1	5	–
2	Rose bengal	1	5	–
3	4-CzIPN	1	5	–
4	$Ir(ppy)_3$	1	5	2
5	$[Ir(dF(CF_3)ppy)_2(dtbbpy)]PF_6$	1	5	13
6	$[Ru(bpy)_3][PF_6]_2$	1	5	–
7	$[Ir(dF(CF_3)ppy)_2(bpy)]PF_6$	5	1	4
8	$[Ir(dF(CF_3)ppy)_2(dOMebpy)]PF_6$	5	1	3
9	$[Ir(dF(CF_3)ppy)_2(dCF_3bpy)]PF_6$	5	1	8
10	$Ir(dFppy)_3$	5	1	–

[a] ^{1}H NMR yield calculated using 1,2,4,5-tetrabromobenzene as internal standard.

X-ray diffraction data

Compound **4j (**CCDC 2485368)

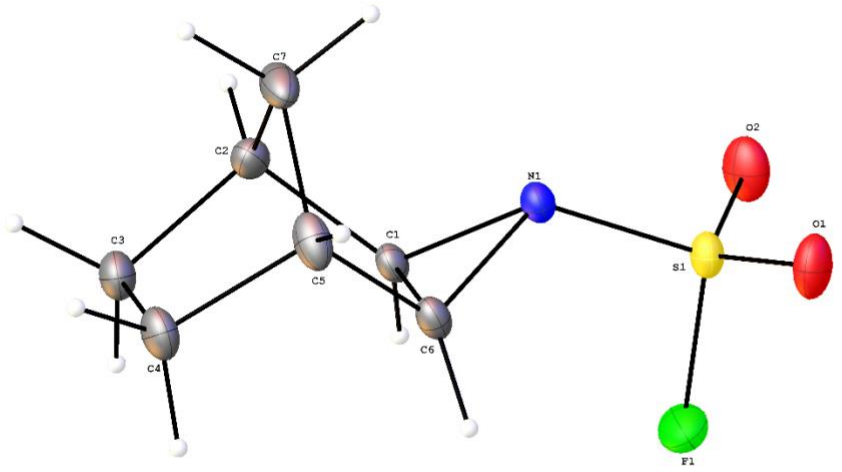

Figure S10. Thermal ellipsoids plot (50 % probability) of **4j**. Hydrogen atoms are shown at an arbitrarily chosen small radius, and not labeled, for clarity.

Table S9. Crystal data and structure refinement for **4j**

**Compound**	**4j**
Formula	$C_7H_{10}NO_2FS$
$D_{calc.}$/ g cm^{-3}	1.573
m/mm^{-1}	3.412
Formula Weight	191.22
Color	colorless
Shape	block-shaped
Size/mm^3	0.52×0.40×0.19
T/K	100.00(10)
Crystal System	orthorhombic
Flack Parameter	0.059(9)
Hooft Parameter	0.066(4)
Space Group	$P2_12_12_1$
a/Å	8.57870(10)
b/Å	8.98840(10)
c/Å	10.46860(10)
a/°	90
b/°	90
g/°	90
V/Å^3	807.221(15)
Z	4
Z'	1
Wavelength/Å	1.54184

Radiation type	Cu K$_a$
Q_{min}/°	6.491
Q_{max}/°	74.446

**Compound**	**4j**
Measured Refl's.	8274
Indep't Refl's	1653
Refl's I≥2 s(I)	1652
R_{int}	0.0253
Parameters	110
Restraints	0
Largest Peak	0.325
Deepest Hole	-0.372
GooF	1.112
wR_2 (all data)	0.0678
wR_2	0.0678
R_1 (all data)	0.0256
R_1	0.0255

Table S10: Fractional Atomic Coordinates (×10^4) and Equivalent Isotropic Displacement Parameters (Å^2×10^3) for **4j**. U_{eq} is defined as 1/3 of the trace of the orthogonalized U_{ij}.

Atom	x	y	z	U_{eq}
S1	7223.5(5)	4930.3(6)	5406.6(4)	13.14(19)
F1	6464.7(15)	4782.5(18)	4043.0(12)	25.8(4)
O1	8022(2)	3573.8(18)	5605.4(17)	21.6(4)
O2	8032(2)	6297.1(18)	5381.6(18)	22.7(4)
N1	5768.1(17)	5045(2)	6361.4(14)	12.0(3)
C1	4296(3)	5817(2)	5942(2)	11.6(4)
C2	3317(3)	6402(2)	7038(2)	15.3(5)
C3	1639(3)	5945(3)	6666(2)	17.1(5)
C4	1645(3)	4215(3)	6841(2)	17.6(5)
C5	3339(3)	3893(3)	7299(2)	17.5(5)
C6	4313(3)	4171(2)	6106(2)	12.9(5)
C7	3662(2)	5285(3)	8109(2)	20.0(5)

Table S11: Anisotropic Displacement Parameters (×10^4) for **QM_ACNB**. The anisotropic displacement factor exponent takes the form: $-2p^2[h^2a^{*2} \times U_{11}+ ... +2hka^* \times b^* \times U_{12}]$

Atom	U_{11}	U_{22}	U_{33}	U_{23}	U_{13}	U_{12}
S1	9.6(3)	13.5(3)	16.3(3)	0.7(2)	2.32(15)	0.4(2)
F1	20.7(6)	42.2(9)	14.5(6)	-3.5(6)	1.6(5)	1.8(7)
O1	16.2(9)	17.2(7)	31.3(9)	1.2(7)	6.6(8)	4.4(6)
O2	16.7(9)	17.9(8)	33.5(9)	3.0(7)	5.5(8)	-3.5(7)
N1	9.1(7)	13.0(8)	13.9(7)	0.0(7)	0.0(5)	0.0(8)
C1	8.0(10)	12.7(9)	14.1(9)	-0.4(8)	-1.6(9)	1.1(7)

Atom	U_{11}	U_{22}	U_{33}	U_{23}	U_{13}	U_{12}
C2	12.6(11)	16.3(10)	17.1(10)	-4.5(9)	-0.3(8)	1.0(8)
C3	11.0(11)	20.8(11)	19.4(11)	-1.0(9)	0.9(9)	1.8(8)
C4	10.3(11)	20.1(11)	22.4(11)	1.7(9)	1.3(10)	-1.5(8)
C5	11.2(11)	20.6(11)	20.7(12)	8.7(9)	1.8(9)	0.0(9)
C6	9.0(10)	12.1(10)	17.5(10)	-0.5(8)	-1.2(9)	-0.4(7)
C7	11.8(9)	36.1(13)	12.0(9)	0.5(9)	0.4(7)	1.1(9)

Table S12: Bond Lengths in Å for **4j**.

Atom	Atom	Length/Å
S1	F1	1.5745(13)
S1	O1	1.4142(17)
S1	O2	1.4112(17)
S1	N1	1.6026(15)
N1	C1	1.506(3)
N1	C6	1.499(3)
C1	C2	1.517(3)
C1	C6	1.490(3)
C2	C3	1.547(3)
C2	C7	1.534(3)
C3	C4	1.565(3)
C4	C5	1.557(4)
C5	C6	1.523(3)
C5	C7	1.537(3)

Table S13: Bond Angles in ° for **4j**.

Atom	Atom	Atom	Angle/°
F1	S1	N1	104.41(7)
O1	S1	F1	105.13(10)
O1	S1	N1	109.95(10)
O2	S1	F1	105.06(10)
O2	S1	O1	121.00(10)
O2	S1	N1	109.81(10)
C1	N1	S1	120.04(13)
C6	N1	S1	120.24(14)
C6	N1	C1	59.44(11)
N1	C1	C2	113.80(18)
C6	C1	N1	60.02(15)
C6	C1	C2	105.2(2)
C1	C2	C3	103.45(17)
C1	C2	C7	102.65(17)
C7	C2	C3	100.93(18)
C2	C3	C4	103.4(2)
C5	C4	C3	103.0(2)
C6	C5	C4	103.24(18)
C6	C5	C7	102.72(17)
C7	C5	C4	100.75(18)
N1	C6	C5	113.34(18)
C1	C6	N1	60.53(15)
C1	C6	C5	104.6(2)
C2	C7	C5	95.45(15)

Table S14: Torsion Angles in ° for **4j**.

Atom	Atom	Atom	Atom	Angle/°
S1	N1	C1	C2	-156.05(15)
S1	N1	C1	C6	109.52(18)
S1	N1	C6	C1	-109.20(17)
S1	N1	C6	C5	156.72(15)
F1	S1	N1	C1	-33.83(17)
F1	S1	N1	C6	36.14(17)
O1	S1	N1	C1	-146.15(16)
O1	S1	N1	C6	-76.19(17)
O2	S1	N1	C1	78.37(17)
O2	S1	N1	C6	148.33(16)
N1	C1	C2	C3	-135.70(18)
N1	C1	C2	C7	-31.0(2)
N1	C1	C6	C5	108.86(18)
C1	N1	C6	C5	-94.1(2)
C1	C2	C3	C4	70.8(2)
C1	C2	C7	C5	-50.26(18)
C2	C1	C6	N1	-109.01(18)
C2	C1	C6	C5	-0.2(3)
C2	C3	C4	C5	0.1(3)
C3	C2	C7	C5	56.37(17)
C3	C4	C5	C6	-71.0(2)
C3	C4	C5	C7	34.9(2)
C4	C5	C6	N1	136.07(19)
C4	C5	C6	C1	72.3(2)
C4	C5	C7	C2	-56.17(17)
C6	N1	C1	C2	94.4(2)
C6	C1	C2	C3	-72.2(2)
C6	C1	C2	C7	32.5(2)
C6	C5	C7	C2	50.21(18)
C7	C2	C3	C4	-35.2(2)
C7	C5	C6	N1	31.6(2)
C7	C5	C6	C1	-32.2(2)

Table S15: Hydrogen Fractional Atomic Coordinates ($\times 10^4$) and Equivalent Isotropic Displacement Parameters ($Å^2 \times 10^3$) for **4j**. U_{eq} is defined as 1/3 of the trace of the orthogonalized U_{ij}.

Atom	x	y	z	U_{eq}
H1	4217.4	6270.6	5071.87	14
H2	3469.77	7474.82	7259.39	18
H3A	1406.15	6219.82	5770.15	20
H3B	862.37	6419.06	7234.62	20
H4A	872.26	3900.78	7489.02	21
H4B	1419.87	3701.62	6024.93	21
H5	3507.31	2915.23	7733.42	21
H6	4239.38	3493.05	5350.39	15
H7A	2936.39	5382.31	8839.61	24
H7B	4754.31	5336.74	8410.98	24

Experimental. Single colorless block-shaped crystals of **4j** were used as supplied. A suitable crystal with dimensions 0.52 × 0.40 × 0.19 mm^3 was selected and mounted on a MITIGEN holder on a XtaLAB Synergy, Dualflex, HyPix diffractometer. The crystal was kept at a steady T = 100.00(10) K during data collection. The structure was solved with the ShelXT 2018/2 (Sheldrick, 2018) solution program using dual methods and by using Olex2 1.5 (Dolomanov et al., 2009) as the graphical interface. The model was refined with ShelXL 2019/1 (Sheldrick, 2015) using full matrix least squares minimization on $\boldsymbol{F^2}$.

Crystal Data. $C_7H_{10}NO_2FS$, M_r = 191.22, orthorhombic, $P2_12_12_1$ (No. 19), a = 8.57870(10) Å, b = 8.98840(10) Å, c = 10.46860(10) Å, $a = b = g = 90^\circ$, V = 807.221(15) Å^3, T = 100.00(10) K, Z = 4, Z' = 1, m(Cu K$_a$) = 3.412, 8274 reflections measured, 1653 unique (R_{int} = 0.0253) which were used in all calculations. The final wR_2 was 0.0678 (all data) and R_1 was 0.0255 (I≥2 s(I)).

Compound **5f** (CCDC 2485372)

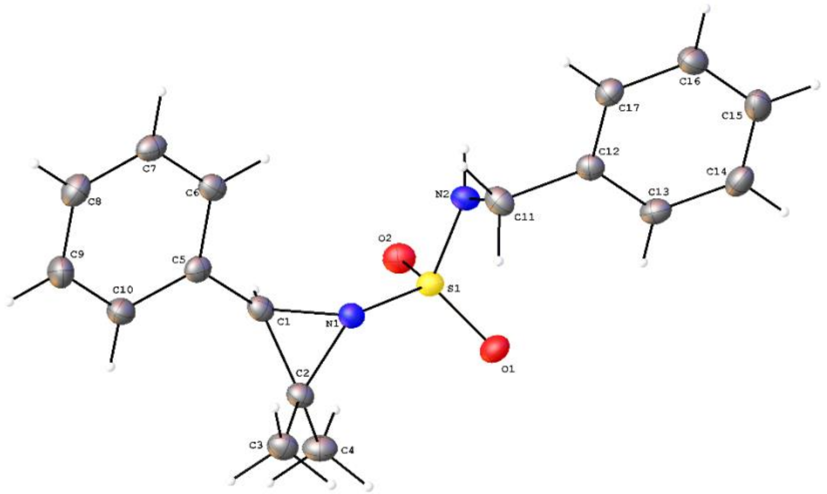

Figure S11 Thermal ellipsoids plot (50 % probability) **5f**. Hydrogen atoms are shown at an arbitrarily chosen small radius, and not labeled, for clarity.

Table S16. Crystal data and structure refinement for **5f**

**Compound**	**5f**
Formula	$C_{17}H_{20}N_2O_2S$
$D_{calc.}$/ g cm^{-3}	1.318
m/mm^{-1}	1.873
Formula Weight	316.41
Color	colorless
Shape	plate-shaped
Size/mm^3	0.19×0.04×0.02
T/K	100.00(10)

Crystal System	orthorhombic
Flack Parameter	-0.009(4)
Hooft Parameter	-0.005(4)
Space Group	$P2_12_12_1$
a/Å	6.29394(5)
b/Å	13.60879(11)
c/Å	18.61169(15)
a/°	90
b/°	90
g/°	90
V/Å^3	1594.15(2)
Z	4
Z'	1
Wavelength/Å	1.54184
Radiation type	Cu K_a
Q_{min}/°	4.024
Q_{max}/°	80.167
Measured Refl's.	33687
Indep't Refl's	3430
Refl's I≥2 s(I)	3354
R_{int}	0.0346
Parameters	201
Restraints	0
Largest Peak	0.376
Deepest Hole	-0.356
GooF	1.073
wR_2 (all data)	0.0700
wR_2	0.0696
R_1 (all data)	0.0269
R_1	0.0263

Table S17: Fractional Atomic Coordinates (×10^4) and Equivalent Isotropic Displacement Parameters (Å^2×10^3) for **5f**. U_{eq} is defined as 1/3 of the trace of the orthogonalized U_{ij}.

Atom	x	y	z	U_{eq}
S1	9229.4(7)	7156.8(3)	3817.9(2)	16.92(11)
O1	9953(2)	6425.6(10)	3325.0(7)	22.5(3)
O2	10726(2)	7569.6(10)	4324.0(7)	22.8(3)
N1	8058(2)	8012.3(11)	3323.0(8)	17.4(3)
N2	7338(3)	6712.8(12)	4292.2(8)	19.5(3)
C1	8484(3)	9069.6(13)	3472.1(10)	17.6(3)
C2	9286(3)	8620.6(13)	2791.1(9)	18.4(4)
C3	8083(3)	8768.9(14)	2099.5(10)	22.2(4)
C4	11648(3)	8462.3(16)	2703.2(11)	23.8(4)
C5	6620(3)	9737.6(13)	3525.1(10)	17.9(4)
C6	4983(3)	9536.1(13)	4007.6(9)	18.9(4)
C7	3313(3)	10191.1(14)	4096.1(10)	21.3(4)
C8	3234(3)	11045.3(15)	3689.8(11)	25.0(4)
C9	4850(4)	11251.4(14)	3202.0(11)	27.0(4)
C10	6550(3)	10606.6(14)	3124.3(11)	23.0(4)
C11	5485(3)	6256.1(13)	3935.9(10)	20.9(4)
C12	5175(3)	5213.8(13)	4190.6(9)	18.6(4)

Atom	x	y	z	U_{eq}
C13	6702(3)	4504.3(15)	4036.3(10)	21.9(4)
C14	6419(3)	3539.5(15)	4263.2(11)	24.8(4)
C15	4630(4)	3286.1(14)	4660.1(10)	26.1(4)
C16	3129(3)	3992.8(15)	4828.2(11)	24.5(4)
C17	3392(3)	4951.9(14)	4585.7(10)	20.9(4)

Table S18: Anisotropic Displacement Parameters (×10[4]) for **5f**. The anisotropic displacement factor exponent takes the form: $-2p^2[h^2a^{*2} \times U_{11} + ... + 2hka^* \times b^* \times U_{12}]$

Atom	U_{11}	U_{22}	U_{33}	U_{23}	U_{13}	U_{12}
S1	15.34(19)	19.89(19)	15.53(19)	0.17(16)	-0.39(15)	1.67(16)
O1	22.6(6)	23.4(6)	21.5(6)	-2.1(5)	2.1(5)	5.1(5)
O2	19.6(6)	28.2(7)	20.5(6)	0.0(5)	-4.2(5)	0.6(6)
N1	17.6(7)	18.2(7)	16.6(7)	0.2(6)	0.4(6)	-1.0(6)
N2	20.9(8)	22.3(7)	15.2(7)	1.1(6)	1.5(6)	-0.7(6)
C1	17.4(8)	18.4(8)	17.1(8)	0.9(7)	-0.8(7)	-3.9(7)
C2	18.0(9)	19.6(8)	17.7(8)	1.0(7)	0.8(7)	-2.3(8)
C3	24.1(10)	25.5(10)	17.1(8)	0.9(7)	-0.8(7)	-1.1(8)
C4	18.7(9)	31.5(10)	21.2(9)	0.1(8)	2.7(7)	-1.0(8)
C5	17.0(8)	18.7(8)	18.0(8)	-3.6(7)	-0.9(7)	-3.4(7)
C6	20.0(8)	20.1(8)	16.6(8)	-0.8(6)	-1.9(7)	-4.2(7)
C7	18.7(9)	24.6(9)	20.8(8)	-5.8(7)	2.3(7)	-4.2(8)
C8	23.5(9)	23.2(9)	28.4(10)	-5.0(8)	-0.6(8)	2.8(8)
C9	34.2(11)	19.1(9)	27.8(10)	1.7(7)	3.0(9)	2.7(8)
C10	25.7(10)	21.2(9)	22.1(9)	-0.3(7)	5.0(8)	-2.4(8)
C11	17.6(9)	23.2(9)	22.0(9)	5.0(7)	-1.8(7)	-1.2(7)
C12	18.8(9)	21.4(8)	15.5(8)	0.0(6)	-1.9(7)	0.1(7)
C13	20.9(9)	26.7(9)	18.2(8)	-1.3(7)	2.2(7)	2.1(8)
C14	29.5(10)	22.4(9)	22.6(9)	-4.5(8)	-0.9(8)	5.9(8)
C15	36.6(12)	18.8(8)	22.8(9)	-0.6(7)	-1.5(8)	-1.4(8)
C16	26.2(10)	24.4(9)	23.1(9)	-0.2(7)	3.6(8)	-4.2(8)
C17	20.1(9)	21.7(9)	21.0(8)	-1.5(7)	0.1(7)	2.1(7)

Table S19: Bond Lengths in Å for **5f**.

Atom	Atom	Length/Å
S1	O1	1.4281(13)
S1	O2	1.4456(14)
S1	N1	1.6576(16)
S1	N2	1.6003(16)
N1	C1	1.490(2)
N1	C2	1.504(2)
N2	C11	1.479(2)
C1	C2	1.495(2)
C1	C5	1.488(3)
C2	C3	1.507(3)
C2	C4	1.511(3)
C5	C6	1.394(3)

Atom	Atom	Length/Å
C5	C10	1.399(3)
C6	C7	1.388(3)
C7	C8	1.388(3)
C8	C9	1.392(3)
C9	C10	1.392(3)
C11	C12	1.508(2)
C12	C13	1.392(3)
C12	C17	1.388(3)
C13	C14	1.391(3)
C14	C15	1.390(3)
C15	C16	1.384(3)
C16	C17	1.391(3)

Table S20: Bond Angles in ° for **5f**.

Atom	Atom	Atom	Angle/°
O1	S1	O2	118.79(9)
O1	S1	N1	105.92(8)
O1	S1	N2	109.18(8)
O2	S1	N1	112.27(8)
O2	S1	N2	105.78(8)
N2	S1	N1	103.93(8)
C1	N1	S1	119.65(12)
C1	N1	C2	59.89(11)
C2	N1	S1	121.57(12)
C11	N2	S1	119.87(12)
N1	C1	C2	60.54(11)
C5	C1	N1	117.41(15)
C5	C1	C2	124.91(16)
N1	C2	C3	112.18(15)
N1	C2	C4	119.89(16)
C1	C2	N1	59.57(11)
C1	C2	C3	119.99(16)
C1	C2	C4	118.82(16)
C3	C2	C4	114.90(16)
C6	C5	C1	120.36(17)
C6	C5	C10	119.13(18)
C10	C5	C1	120.39(17)
C7	C6	C5	120.65(17)
C8	C7	C6	120.02(18)
C7	C8	C9	119.89(19)
C8	C9	C10	120.18(18)
C9	C10	C5	120.11(18)
N2	C11	C12	110.90(15)
C13	C12	C11	119.86(17)
C17	C12	C11	120.85(17)
C17	C12	C13	119.29(17)
C14	C13	C12	120.24(19)
C15	C14	C13	119.96(18)
C16	C15	C14	120.04(18)

Atom	Atom	Atom	Angle/°
C15	C16	C17	119.83(19)
C12	C17	C16	120.61(18)

Table S21: Torsion Angles in ° for **5f**.

Atom	Atom	Atom	Atom	Angle/°
S1	N1	C1	C2	111.49(15)
S1	N1	C1	C5	-131.93(15)
S1	N1	C2	C1	-108.35(15)
S1	N1	C2	C3	138.91(14)
S1	N1	C2	C4	-0.5(2)
S1	N2	C11	C12	123.50(15)
O1	S1	N1	C1	-135.82(14)
O1	S1	N1	C2	-64.94(14)
O1	S1	N2	C11	-52.99(16)
O2	S1	N1	C1	-4.68(16)
O2	S1	N1	C2	66.20(15)
O2	S1	N2	C11	178.10(14)
N1	S1	N2	C11	59.69(15)
N1	C1	C2	C3	99.59(18)
N1	C1	C2	C4	-109.66(19)
N1	C1	C5	C6	55.3(2)
N1	C1	C5	C10	-128.69(18)
N2	S1	N1	C1	109.17(14)
N2	S1	N1	C2	-179.96(13)
N2	C11	C12	C13	-65.3(2)
N2	C11	C12	C17	113.90(19)
C1	N1	C2	C3	-112.74(18)
C1	N1	C2	C4	107.89(19)
C1	C5	C6	C7	175.45(17)
C1	C5	C10	C9	-176.92(18)
C2	N1	C1	C5	116.58(18)
C2	C1	C5	C6	127.01(19)
C2	C1	C5	C10	-57.0(3)
C5	C1	C2	N1	-104.50(19)
C5	C1	C2	C3	-4.9(3)
C5	C1	C2	C4	145.83(19)
C5	C6	C7	C8	1.5(3)
C6	C5	C10	C9	-0.9(3)
C6	C7	C8	C9	-1.0(3)
C7	C8	C9	C10	-0.5(3)
C8	C9	C10	C5	1.4(3)
C10	C5	C6	C7	-0.6(3)
C11	C12	C13	C14	-179.40(17)
C11	C12	C17	C16	-179.05(18)
C12	C13	C14	C15	-1.5(3)
C13	C12	C17	C16	0.2(3)
C13	C14	C15	C16	0.1(3)
C14	C15	C16	C17	1.4(3)

Atom	Atom	Atom	Atom	Angle/°
C15	C16	C17	C12	-1.6(3)
C17	C12	C13	C14	1.4(3)

Table S22: Hydrogen Fractional Atomic Coordinates (×10^4) and Equivalent Isotropic Displacement Parameters (Å^2×10^3) for **5f**. U_{eq} is defined as 1/3 of the trace of the orthogonalized U_{ij}.

Atom	x	y	z	U_{eq}
H2	7400.87	6740.52	4764.1	23
H1	9606.57	9188.77	3842.34	21
H3A	8336.34	8209.36	1779.4	33
H3B	8569.33	9374.27	1866.65	33
H3C	6559.8	8819.59	2202.86	33
H4A	12284.25	8322.2	3172.71	36
H4B	12295.33	9055.68	2500.63	36
H4C	11895.69	7906.27	2379.25	36
H6	5010.55	8944.61	4278.59	23
H7	2222.91	10054.56	4434.86	26
H8	2079.77	11488.73	3744.64	30
H9	4791.11	11833.76	2921.29	32
H10	7665.86	10756.9	2798.45	28
H11A	5703.13	6259.14	3409.11	25
H11B	4193.7	6644.85	4042.45	25
H13	7941.6	4679.94	3775.1	26
H14	7447.45	3053.99	4147.11	30
H15	4437.54	2627.32	4815.98	31
H16	1921.98	3823.35	5108.59	29
H17	2342.26	5432.08	4691.53	25

Experimental. Single colorless plate-shaped crystals of **5f** were used as supplied. A suitable crystal with dimensions 0.19 × 0.04 × 0.02 mm^3 was selected and mounted on a MITIGEN holder on a XtaLAB Synergy, Dualflex, HyPix diffractometer. The crystal was kept at a steady T = 100.00(10) K during data collection. The structure was solved with the ShelXT 2018/2 (Sheldrick, 2018) solution program using dual methods and by using Olex2 1.5 (Dolomanov et al., 2009) as the graphical interface. The model was refined with ShelXL 2019/1 (Sheldrick, 2015) using full matrix least squares minimization on $\boldsymbol{F}^2$.

Crystal Data. $C_{17}H_{20}N_2O_2S$, M_r = 316.41, orthorhombic, $P2_12_12_1$ (No. 19), a = 6.29394(5) Å, b = 13.60879(11) Å, c = 18.61169(15) Å, $a = b = g$ = 90°, V = 1594.15(2) Å^3, T = 100.00(10) K, Z = 4, Z' = 1, m(Cu K_a) = 1.873, 33687 reflections measured, 3430 unique (R_{int} = 0.0346) which were used in all calculations. The final wR_2 was 0.0700 (all data) and R_1 was 0.0263 (I≥2 s(I)).

Compound **5e** (CCDC 2491898)

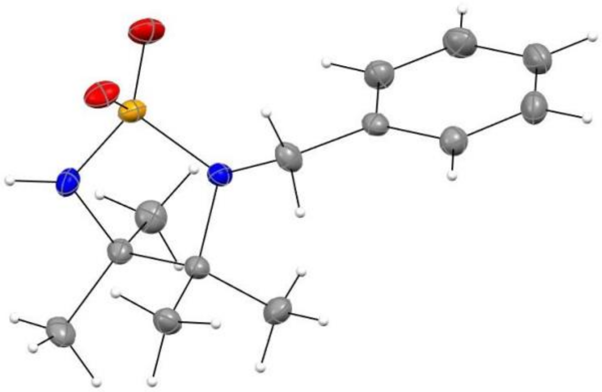

Figure S12 Thermal ellipsoids plot (50 % probability) **5e**. Hydrogen atoms are shown at an arbitrarily chosen small radius, and not labeled, for clarity.

Table S23. Crystal data and structure refinement for **5e**

**Compound**	**5e**
Formula	$C_{13}H_{20}N_2O_2S$
$D_{calc.}$/ g cm^{-3}	1.304
m/mm^{-1}	2.079
Formula Weight	268.37
Colour	colourless
Shape	block-shaped
Size/mm^3	0.10×0.10×0.10
T/K	100.00(10)
Crystal System	monoclinic
Space Group	$P2_1/c$
a/Å	12.8757(2)
b/Å	8.27280(10)
c/Å	25.8394(2)
a/°	90
b/°	96.6790(10)
g/°	90
V/Å^3	2733.68(6)
Z	8
Z'	2
Wavelength/Å	1.54184
Radiation type	Cu K_a
Q_{min}/°	3.444
Q_{max}/°	80.209
Measured Refl's.	29464
Indep't Refl's	5861
Refl's I≥2*s*(I)	5599
R_{int}	0.0426
Parameters	334

Restraints	0
Largest Peak/eÅ^3	0.839
Deepest Hole/eÅ^3	-0.780
GooF	1.063
wR_2 (all data)	0.1201
wR_2	0.1190
R_1 (all data)	0.0445
R_1	0.0433

Table S24: Fractional Atomic Coordinates (×10^4) and Equivalent Isotropic Displacement Parameters (Å^2×10^3) for **5e**. U_{eq} is defined as 1/3 of the trace of the orthogonalized U_{ij}.

Atom	*x*	*y*	*z*	U(eq)
S1A	4070.4(3)	8985.6(5)	2771.1(2)	15.77(12)
S1B	6564.5(3)	3612.4(5)	4936.8(2)	16.72(12)
O2A	4985.9(10)	7991.2(15)	2910.6(4)	21.8(3)
O2B	5465.2(10)	3202.7(15)	4878.8(5)	24.2(3)
O1A	3548.0(10)	8637.8(16)	2260.9(4)	24.4(3)
O1B	7213.7(10)	2545.9(15)	5272.6(5)	24.2(3)
N1A	3248.9(11)	8912.5(16)	3206.9(5)	15.1(3)
N1B	7069.9(11)	3797.5(16)	4391.3(5)	15.9(3)
N2B	6699.5(11)	5512.8(17)	5111.1(5)	18.4(3)
N2A	4359.0(11)	10869.4(17)	2868.2(5)	17.8(3)
C1A	3313.0(12)	10391.0(19)	3540.0(6)	15.5(3)
C3A	2268.6(13)	10654(2)	3747.9(7)	21.1(3)
C8A	1967.1(13)	6681.1(19)	3297.0(6)	16.5(3)
C8B	7932.9(13)	1544(2)	3945.3(6)	17.8(3)
C7A	3066.9(13)	7322(2)	3430.4(6)	18.8(3)
C9A	1570.0(13)	5609(2)	3640.1(7)	20.0(3)
C2B	7353.4(13)	6390(2)	4756.3(6)	17.8(3)
C2A	3558.0(12)	11706.9(19)	3141.4(6)	16.8(3)
C7B	6938.0(13)	2448(2)	4021.5(6)	18.2(3)
C13A	1360.8(14)	7077(2)	2832.6(6)	21.9(3)
C10A	584.6(14)	4925(2)	3517.0(7)	24.1(4)
C6A	4053.9(14)	13223(2)	3396.3(7)	22.4(3)
C1B	7094.1(13)	5514(2)	4218.5(6)	17.6(3)
C4B	6043.6(14)	6039(2)	3931.5(7)	22.5(4)
C10B	8811.9(15)	-290(2)	3413.4(7)	26.1(4)
C12B	9630.8(14)	459(2)	4260.0(8)	26.6(4)
C4A	4189.9(13)	10227(2)	3993.4(6)	19.6(3)
C11B	9642.4(14)	-423(2)	3803.3(8)	26.4(4)
C13B	8776.3(14)	1440(2)	4333.2(7)	21.7(3)
C12A	370.0(14)	6411(2)	2715.1(7)	26.2(4)
C5A	2612.0(14)	12177(2)	2755.9(7)	22.3(3)
C3B	7943.1(15)	5692(2)	3859.5(7)	24.8(4)
C9B	7963.8(14)	694(2)	3483.2(7)	22.6(4)
C11A	-17.5(14)	5333(2)	3055.3(7)	25.7(4)
C5B	8500.5(14)	6227(2)	4977.0(7)	24.1(4)
C6B	7037.9(15)	8166(2)	4740.0(7)	23.8(4)

Table S25 Anisotropic Displacement Parameters (×10^4) for **5e**. The anisotropic displacement factor exponent takes the form: $-2p^2[h^2a^{*2} \times U_{11} + \ldots + 2hka^* \times b^* \times U_{12}]$

Atom	U_{11}	U_{22}	U_{33}	U_{23}	U_{13}	U_{12}
S1A	22.4(2)	13.1(2)	12.32(19)	0.08(13)	4.16(14)	2.04(14)
S1B	21.5(2)	15.2(2)	14.34(19)	0.77(13)	5.52(14)	-2.13(14)
O2A	28.0(6)	19.0(6)	19.1(6)	0.1(5)	5.4(5)	8.3(5)
O2B	24.1(6)	19.9(6)	30.5(7)	-4.0(5)	11.3(5)	-6.0(5)
O1A	34.3(7)	26.1(6)	12.4(5)	-1.0(5)	1.3(5)	0.9(5)
O1B	34.5(7)	21.2(6)	16.9(5)	5.9(5)	3.4(5)	2.2(5)
N1A	20.7(7)	10.7(6)	14.3(6)	-0.5(5)	3.9(5)	-1.5(5)
N1B	20.7(6)	14.0(6)	13.5(6)	0.3(5)	4.5(5)	-1.2(5)
N2B	23.8(7)	17.4(7)	15.0(6)	-1.5(5)	6.5(5)	-3.5(5)
N2A	19.8(7)	14.6(6)	20.5(7)	2.0(5)	8.9(5)	-0.4(5)
C1A	17.9(7)	13.0(7)	16.1(7)	-2.3(6)	3.7(6)	-1.3(6)
C3A	21.6(8)	22.6(8)	20.5(8)	-1.7(7)	7.9(6)	0.5(6)
C8A	19.8(8)	12.6(7)	17.3(7)	-3.0(6)	3.2(6)	-0.3(6)
C8B	21.4(8)	14.3(7)	18.5(7)	2.0(6)	5.3(6)	-1.9(6)
C7A	22.1(8)	13.6(7)	20.1(7)	3.1(6)	0.0(6)	-2.6(6)
C9A	22.9(8)	17.1(8)	20.2(8)	1.4(6)	4.1(6)	-0.3(6)
C2B	21.5(8)	16.6(8)	15.6(7)	0.1(6)	3.9(6)	-4.6(6)
C2A	18.6(7)	12.2(7)	20.3(7)	-1.1(6)	5.7(6)	0.2(6)
C7B	19.9(8)	18.5(8)	16.0(7)	-2.5(6)	1.5(6)	-0.7(6)
C13A	24.4(8)	23.0(8)	18.2(8)	0.6(6)	1.9(6)	-2.1(7)
C10A	21.9(8)	22.6(8)	28.7(9)	0.6(7)	7.8(7)	-3.6(7)
C6A	24.6(8)	12.2(7)	31.0(9)	-2.1(7)	5.6(7)	-1.6(6)
C1B	22.5(8)	15.4(7)	15.1(7)	1.4(6)	3.3(6)	-3.3(6)
C4B	29.2(9)	18.0(8)	19.0(8)	1.0(6)	-2.7(7)	0.1(7)
C10B	32.5(9)	20.1(8)	27.7(9)	-3.5(7)	11.2(7)	-0.7(7)
C12B	19.8(8)	24.8(9)	34.9(10)	-0.2(8)	2.0(7)	-1.3(7)
C4A	22.6(8)	19.1(8)	16.7(7)	-1.4(6)	0.4(6)	-3.4(6)
C11B	22.3(8)	18.7(8)	40.2(10)	-0.8(8)	12.4(7)	-0.8(7)
C13B	22.1(8)	21.6(8)	21.6(8)	-2.3(7)	2.8(6)	-1.2(6)
C12A	24.3(9)	32.2(10)	20.9(8)	-2.5(7)	-2.1(7)	-1.5(7)
C5A	24.5(8)	17.8(8)	24.4(8)	4.3(7)	2.3(7)	3.5(6)
C3B	32.2(9)	25.3(9)	18.8(8)	1.4(7)	10.7(7)	-7.3(7)
C9B	28.6(9)	19.9(8)	19.7(8)	-0.5(7)	4.0(7)	-0.4(7)
C11A	20.2(8)	27.5(9)	29.7(9)	-7.0(8)	4.1(7)	-5.0(7)
C5B	23.6(9)	27.0(9)	21.1(8)	-1.5(7)	0.2(6)	-6.7(7)
C6B	33.2(9)	16.6(8)	21.8(8)	-1.4(7)	3.8(7)	-4.7(7)

Table S26: Bond Lengths in Å for **5e**.

Atom	Atom	Length/Å	Atom	Atom	Length/Å
S1A	O1A	1.4375(12)	C8B	C7B	1.516(2)
S1A	N1A	1.6334(14)	C8B	C13B	1.392(2)
S1A	N2A	1.6150(14)	C8B	C9B	1.390(2)
S1B	O2B	1.4463(13)	C9A	C10A	1.392(2)
S1B	O1B	1.4357(13)	C2B	C1B	1.568(2)
S1B	N1B	1.6268(13)	C2B	C5B	1.526(2)
S1B	N2B	1.6391(15)	C2B	C6B	1.524(2)
N1A	C1A	1.4922(19)	C2A	C6A	1.522(2)
N1A	C7A	1.466(2)	C2A	C5A	1.531(2)
N1B	C7B	1.467(2)	C13A	C12A	1.390(2)
N1B	C1B	1.490(2)	C10A	C11A	1.387(3)
N2B	C2B	1.502(2)	C1B	C4B	1.528(2)

N2A	C2A	1.487(2)	C1B	C3B	1.521(2)
C1A	C3A	1.521(2)	C10B	C11B	1.386(3)
C1A	C2A	1.556(2)	C10B	C9B	1.390(3)
C1A	C4A	1.536(2)	C12B	C11B	1.389(3)
C8A	C7A	1.514(2)	C12B	C13B	1.397(3)
C8A	C9A	1.393(2)	C12A	C11A	1.386(3)

Table S27: Bond Angles in ° for **5e**.

Atom	Atom	Atom	Angle/°	Atom	Atom	Atom	Angle/°
O2A	S1A	N1A	112.40(7)	C9B	C8B	C13B	119.29(16)
O2A	S1A	N2A	110.09(8)	N1A	C7A	C8A	114.38(13)
O1A	S1A	O2A	113.27(7)	C10A	C9A	C8A	120.39(16)
O1A	S1A	N1A	110.70(8)	N2B	C2B	C1B	103.96(12)
O1A	S1A	N2A	114.22(8)	N2B	C2B	C5B	108.43(13)
N2A	S1A	N1A	94.84(7)	N2B	C2B	C6B	108.42(13)
O2B	S1B	N1B	114.68(7)	C5B	C2B	C1B	112.80(14)
O2B	S1B	N2B	108.93(7)	C6B	C2B	C1B	113.10(14)
O1B	S1B	O2B	114.37(8)	C6B	C2B	C5B	109.79(14)
O1B	S1B	N1B	108.54(7)	N2A	C2A	C1A	101.15(12)
O1B	S1B	N2B	112.81(8)	N2A	C2A	C6A	108.07(13)
N1B	S1B	N2B	96.17(7)	N2A	C2A	C5A	110.64(13)
C1A	N1A	S1A	111.74(10)	C6A	C2A	C1A	113.41(13)
C7A	N1A	S1A	116.78(11)	C6A	C2A	C5A	109.51(14)
C7A	N1A	C1A	120.59(13)	C5A	C2A	C1A	113.64(13)
C7B	N1B	S1B	117.72(11)	N1B	C7B	C8B	115.14(13)
C7B	N1B	C1B	122.35(13)	C12A	C13A	C8A	120.23(16)
C1B	N1B	S1B	111.95(10)	C11A	C10A	C9A	120.09(16)
C2B	N2B	S1B	110.16(10)	N1B	C1B	C2B	100.63(12)
C2A	N2A	S1A	111.32(10)	N1B	C1B	C4B	111.61(13)
N1A	C1A	C3A	109.21(13)	N1B	C1B	C3B	108.46(14)
N1A	C1A	C2A	101.17(12)	C4B	C1B	C2B	112.57(14)
N1A	C1A	C4A	110.73(13)	C3B	C1B	C2B	113.69(14)
C3A	C1A	C2A	112.94(13)	C3B	C1B	C4B	109.56(14)
C3A	C1A	C4A	110.17(13)	C11B	C10B	C9B	120.21(17)
C4A	C1A	C2A	112.29(13)	C11B	C12B	C13B	120.41(17)
C9A	C8A	C7A	118.76(14)	C10B	C11B	C12B	119.53(17)
C13A	C8A	C7A	121.99(15)	C8B	C13B	C12B	119.93(16)
C13A	C8A	C9A	119.19(15)	C11A	C12A	C13A	120.36(17)
C13B	C8B	C7B	122.49(15)	C8B	C9B	C10B	120.59(17)
C9B	C8B	C7B	118.05(15)	C12A	C11A	C10A	119.73(16)

Table S28: Torsion Angles in ° for **5e**

A	B	C	D	Angle/°	A	B	C	D	Angle/°
S1A	N1A	C1A	C3A	153.75(11)	C3A	C1A	C2A	N2A	-160.09(13)
S1A	N1A	C1A	C2A	34.45(14)	C3A	C1A	C2A	C6A	84.44(17)
S1A	N1A	C1A	C4A	-84.77(14)	C3A	C1A	C2A	C5A	-41.50(18)
S1A	N1A	C7A	C8A	-114.21(14)	C8A	C9A	C10A	C11A	-1.2(3)
S1A	N2A	C2A	C1A	39.68(14)	C8A	C13A	C12A	C11A	-0.9(3)
S1A	N2A	C2A	C6A	159.04(11)	C7A	N1A	C1A	C3A	-63.17(18)
S1A	N2A	C2A	C5A	-81.06(14)	C7A	N1A	C1A	C2A	177.52(13)
S1B	N1B	C7B	C8B	-111.80(14)	C7A	N1A	C1A	C4A	58.31(18)

S1B	N1B	C1B	C2B	37.03(14)	C7A	C8A	C9A	C10A	-176.46(15)
S1B	N1B	C1B	C4B	-82.60(14)	C7A	C8A	C13A	C12A	177.41(16)
S1B	N1B	C1B	C3B	156.61(11)	C9A	C8A	C7A	N1A	-152.89(15)
S1B	N2B	C2B	C1B	31.77(15)	C9A	C8A	C13A	C12A	0.2(3)
S1B	N2B	C2B	C5B	-88.48(14)	C9A	C10A	C11A	C12A	0.6(3)
S1B	N2B	C2B	C6B	152.37(12)	C7B	N1B	C1B	C2B	-174.87(13)
O2A	S1A	N1A	C1A	102.92(11)	C7B	N1B	C1B	C4B	65.50(19)
O2A	S1A	N1A	C7A	-41.67(14)	C7B	N1B	C1B	C3B	-55.29(19)
O2A	S1A	N2A	C2A	-134.29(11)	C7B	C8B	C13B	C12B	173.69(16)
O2B	S1B	N1B	C7B	-53.54(14)	C7B	C8B	C9B	C10B	-173.41(16)
O2B	S1B	N1B	C1B	96.17(12)	C13A	C8A	C7A	N1A	29.9(2)
O2B	S1B	N2B	C2B	-128.24(11)	C13A	C8A	C9A	C10A	0.8(3)
O1A	S1A	N1A	C1A	-129.31(11)	C13A	C12A	C11A	C10A	0.5(3)
O1A	S1A	N1A	C7A	86.09(13)	C1B	N1B	C7B	C8B	101.82(17)
O1A	S1A	N2A	C2A	96.97(12)	C4A	C1A	C2A	N2A	74.59(15)
O1B	S1B	N1B	C7B	75.73(13)	C4A	C1A	C2A	C6A	-40.88(18)
O1B	S1B	N1B	C1B	-134.55(11)	C4A	C1A	C2A	C5A	-166.82(13)
O1B	S1B	N2B	C2B	103.63(12)	C11B	C10B	C9B	C8B	-0.6(3)
N1A	S1A	N2A	C2A	-18.31(12)	C11B	C12B	C13B	C8B	-0.4(3)
N1A	C1A	C2A	N2A	-43.50(14)	C13B	C8B	C7B	N1B	28.4(2)
N1A	C1A	C2A	C6A	-158.96(13)	C13B	C8B	C9B	C10B	2.0(3)
N1A	C1A	C2A	C5A	75.09(16)	C13B	C12B	C11B	C10B	1.7(3)
N1B	S1B	N2B	C2B	-9.48(12)	C9B	C8B	C7B	N1B	-156.41(15)
N2B	S1B	N1B	C7B	-167.68(12)	C9B	C8B	C13B	C12B	-1.5(3)
N2B	S1B	N1B	C1B	-17.96(12)	C9B	C10B	C11B	C12B	-1.3(3)
N2B	C2B	C1B	N1B	-40.93(15)	C5B	C2B	C1B	N1B	76.32(16)
N2B	C2B	C1B	C4B	78.00(16)	C5B	C2B	C1B	C4B	-164.75(14)
N2B	C2B	C1B	C3B	-156.67(14)	C5B	C2B	C1B	C3B	-39.41(19)
N2A	S1A	N1A	C1A	-11.14(12)	C6B	C2B	C1B	N1B	-158.32(13)
N2A	S1A	N1A	C7A	-155.74(12)	C6B	C2B	C1B	C4B	-39.39(19)
C1A	N1A	C7A	C8A	104.48(16)	C6B	C2B	C1B	C3B	85.95(18)

Experimental. Single colourless block-shaped crystals of QM_2025_9_11 were used as supplied. A suitable crystal with dimensions 0.10 × 0.10 × 0.10 mm^3 was selected and mounted on a XtaLAB Synergy, Dualflex, HyPix diffractometer. The crystal was kept at a steady T = 100.00(10) K during data collection. The structure was solved with the ShelXT 2018/2 (Sheldrick, 2018) solution program using dual methods and by using Olex2 1.5 (Dolomanov et al., 2009) as the graphical interface. The model was refined with XL (Sheldrick, 2008) using full matrix least squares minimisation on $\boldsymbol{F}^2$.

Crystal Data. $C_{13}H_{20}N_2O_2S$, M_r = 268.37, monoclinic, $P2_1/c$ (No. 14), a = 12.8757(2) Å, b = 8.27280(10) Å, c = 25.8394(2) Å, b = 96.6790(10)°, a = g = 90°, V = 2733.68(6) $Å^3$, T = 100.00(10) K, Z = 8, Z' = 2, m(Cu K_a) = 2.079, 29464 reflections measured, 5861 unique (R_{int} = 0.0426) which were used in all calculations. The final wR_2 was 0.1201 (all data) and R_1 was 0.0433 (I≥2s(I)).

NMR spectra

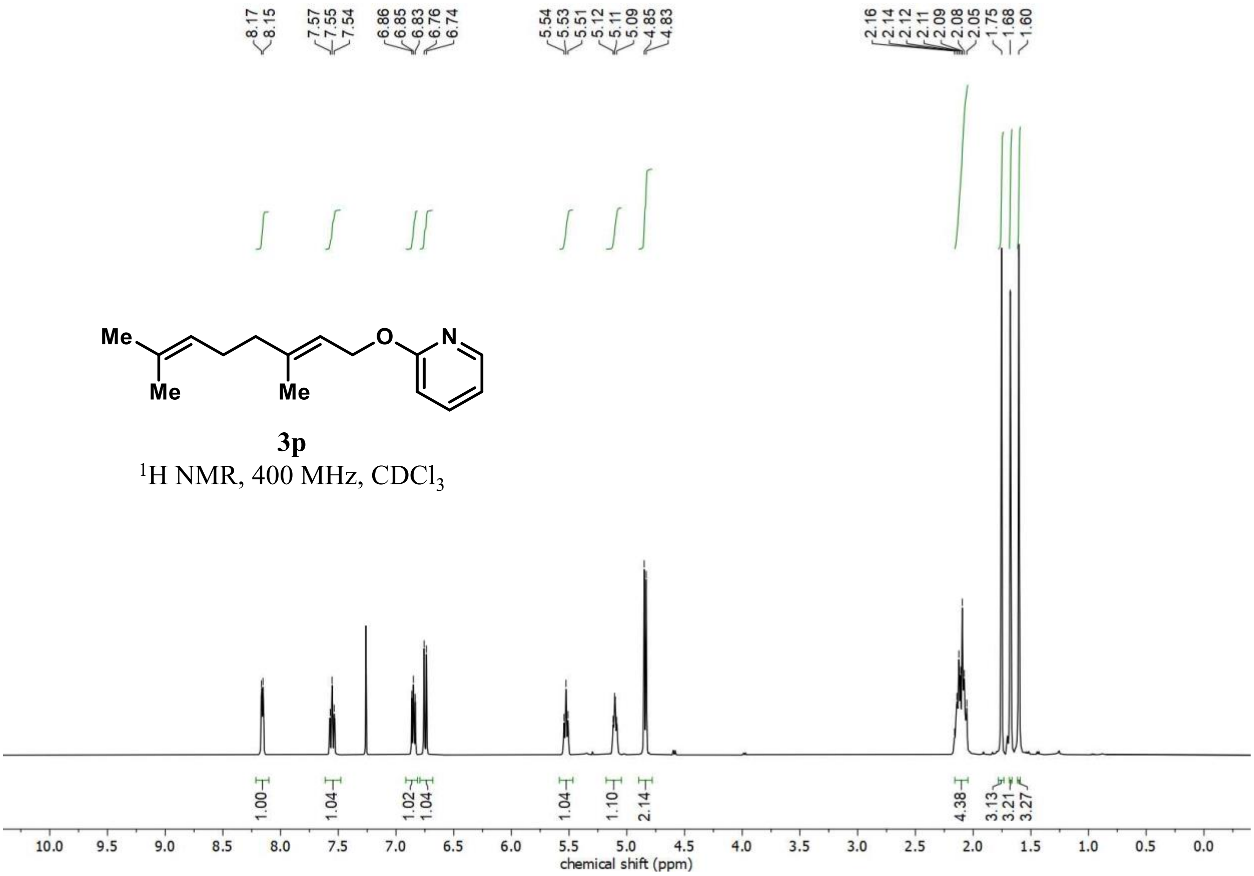
8.17
8.15
7.57
7.55
7.54
6.86
6.85
6.83
6.76
6.74
5.54
5.53
5.51
5.12
5.11
5.09
4.85
4.83
2.16
2.14
2.12
2.11
2.09
2.08
2.05
1.75
1.68
1.60
Me
Me
Me
O
N
3p
^{1}H NMR, 400 MHz, $CDCl_3$
1.00
1.04
1.02
1.04
1.04
1.10
2.14
4.38
3.13
3.21
3.27
10.0
9.5
9.0
8.5
8.0
7.5
7.0
6.5
6.0
5.5
5.0
4.5
4.0
3.5
3.0
2.5
2.0
1.5
1.0
0.5
0.0
chemical shift (ppm)

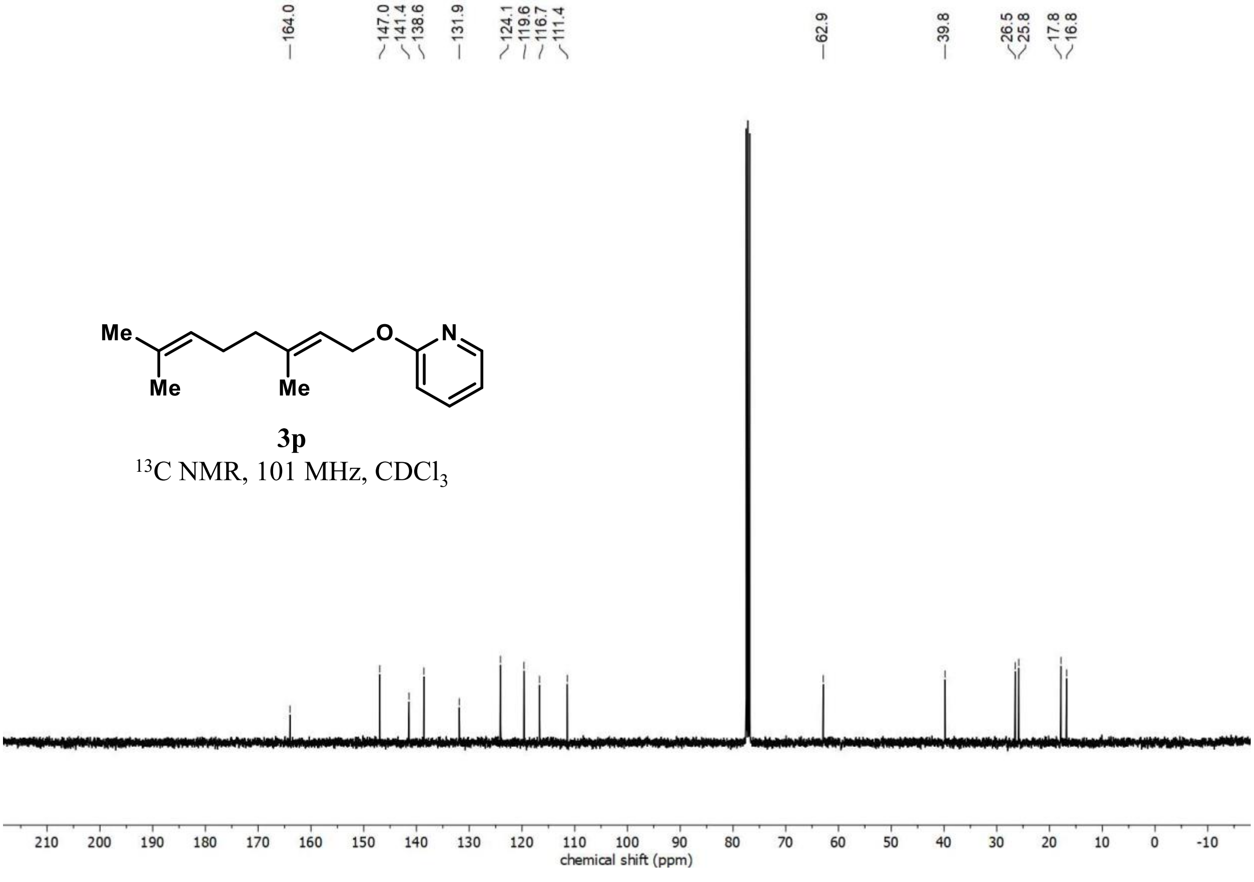
164.0
147.0
141.4
138.6
131.9
124.1
119.6
116.7
111.4
62.9
39.8
26.5
25.8
17.8
16.8
Me
Me
Me
O
N
3p
^{13}C NMR, 101 MHz, $CDCl_3$
210
200
190
180
170
160
150
140
130
120
110
100
90
80
70
60
50
40
30
20
10
0
-10
chemical shift (ppm)

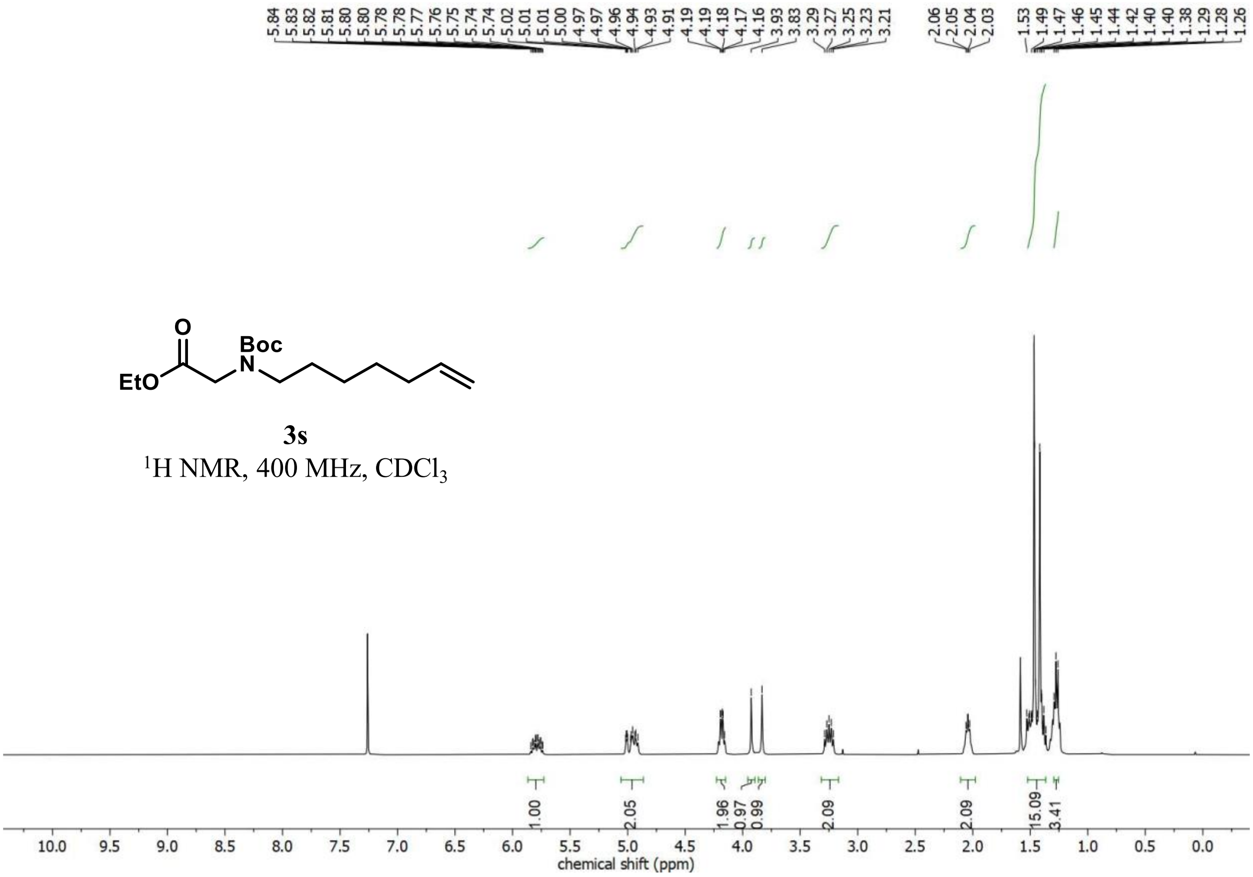

3s
^{1}H NMR, 400 MHz, $CDCl_3$

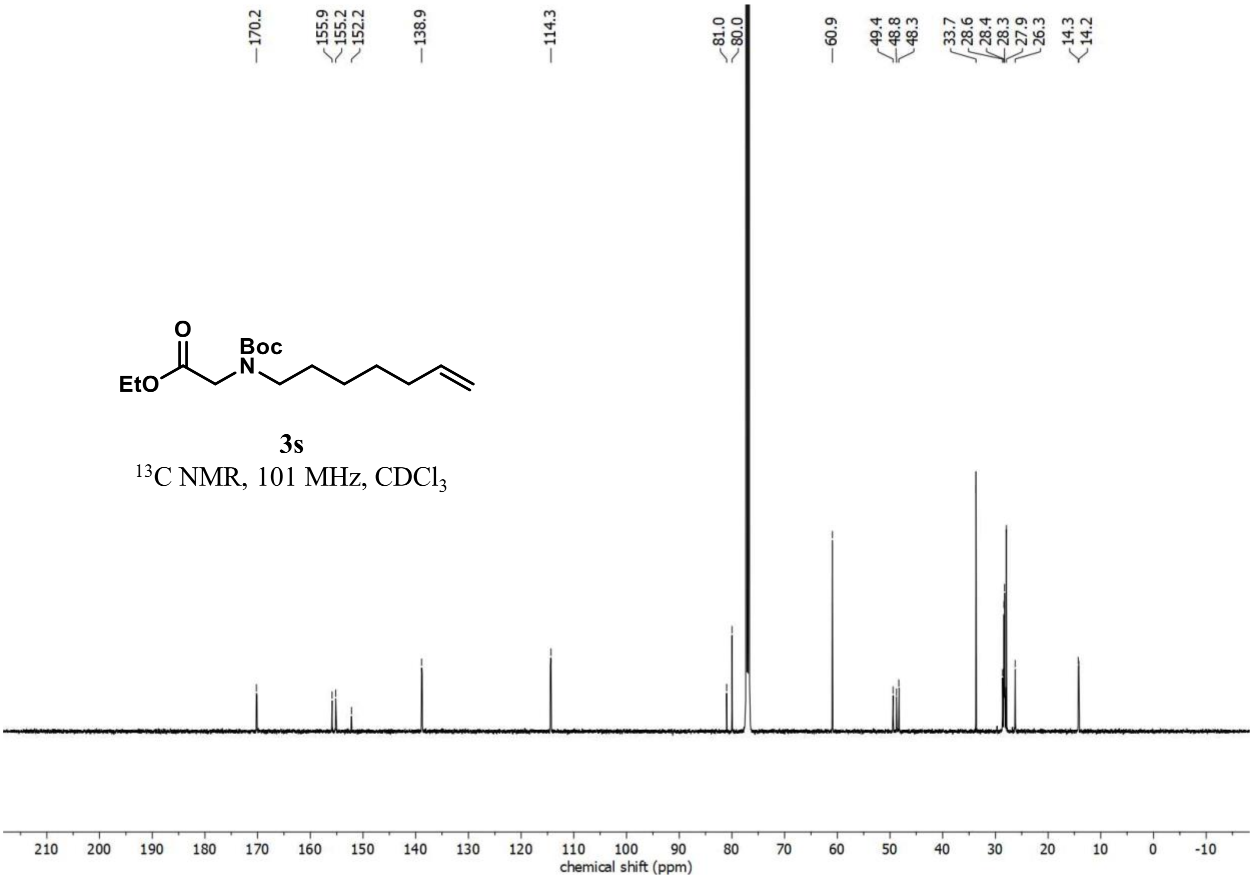

3s
^{13}C NMR, 101 MHz, $CDCl_3$

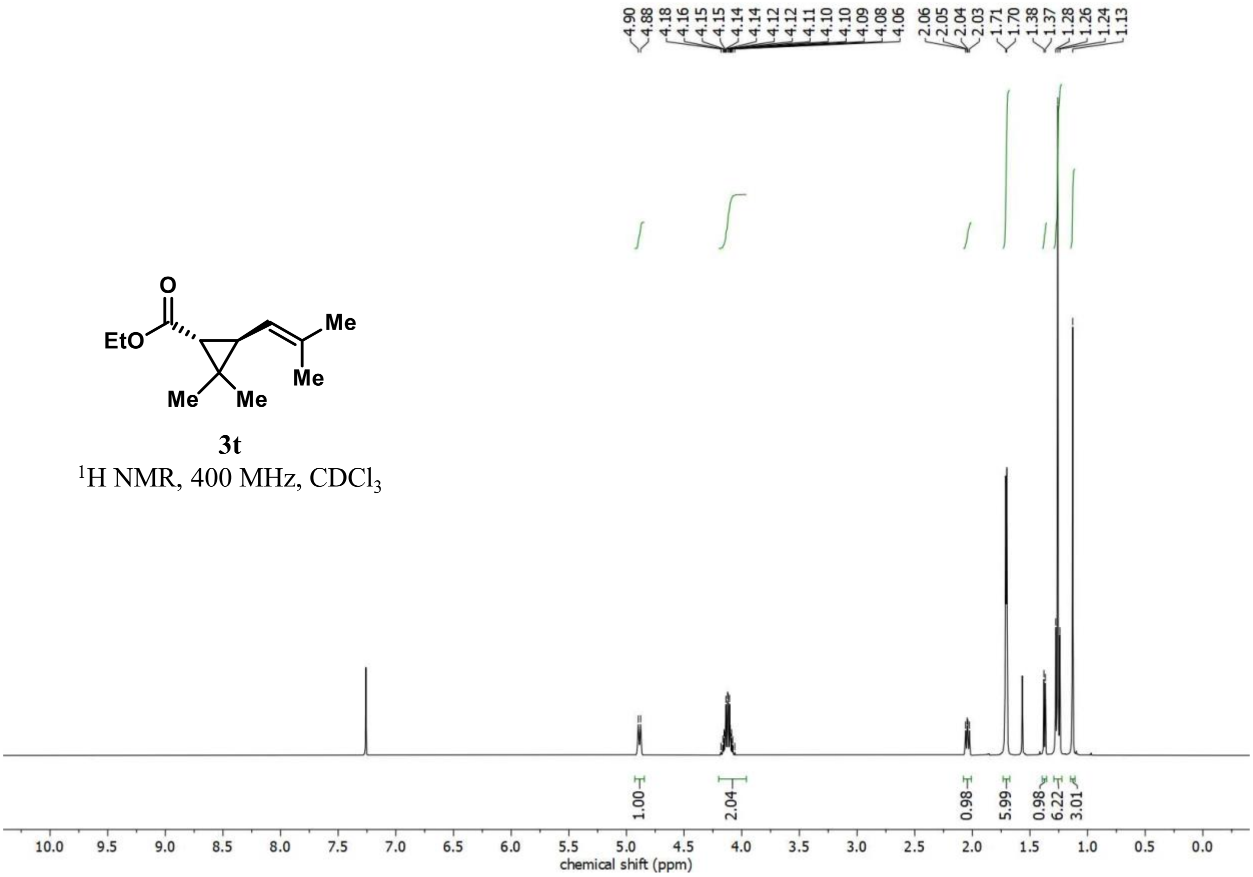

3t
^{1}H NMR, 400 MHz, $CDCl_3$

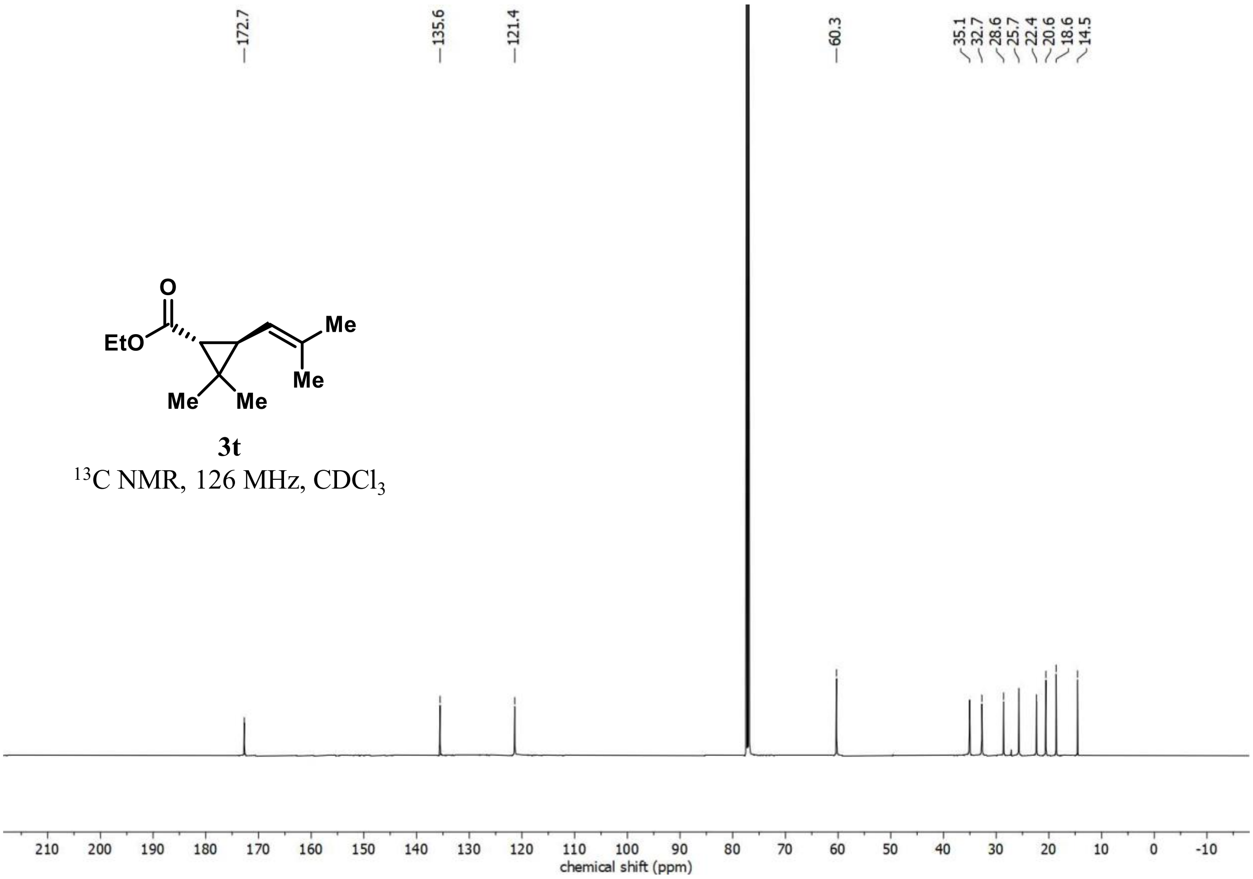

3t
^{13}C NMR, 126 MHz, $CDCl_3$

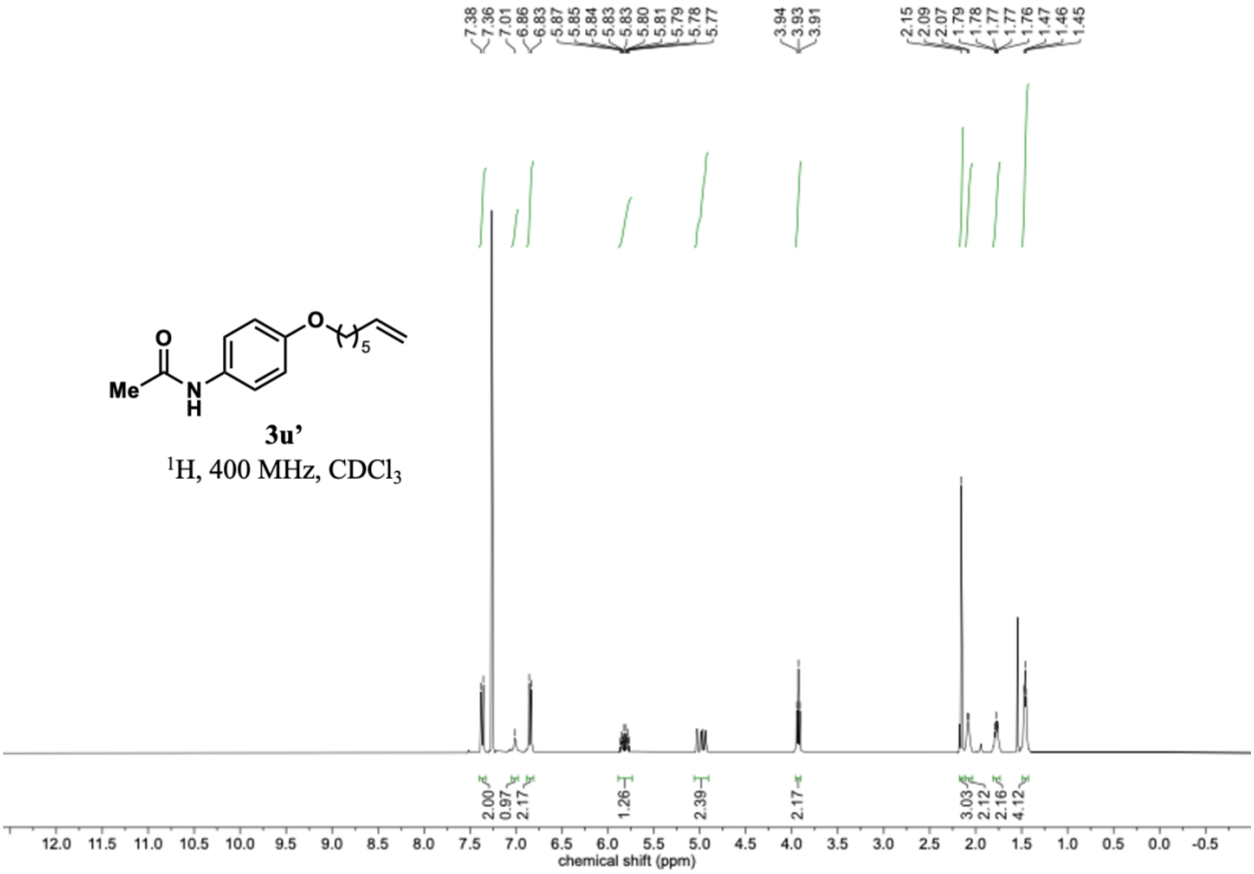

3u'

^{1}H, 400 MHz, $CDCl_3$

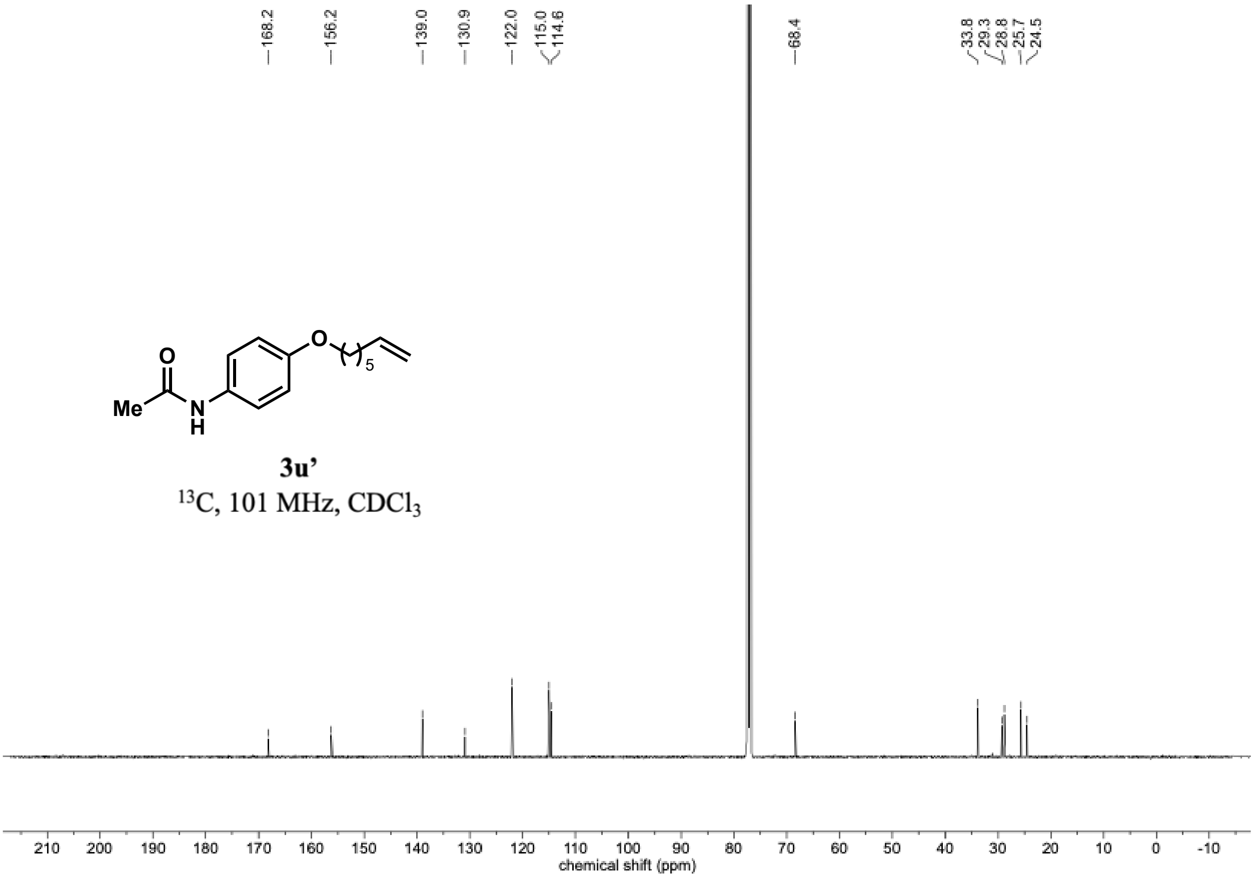

3u'

^{13}C, 101 MHz, $CDCl_3$

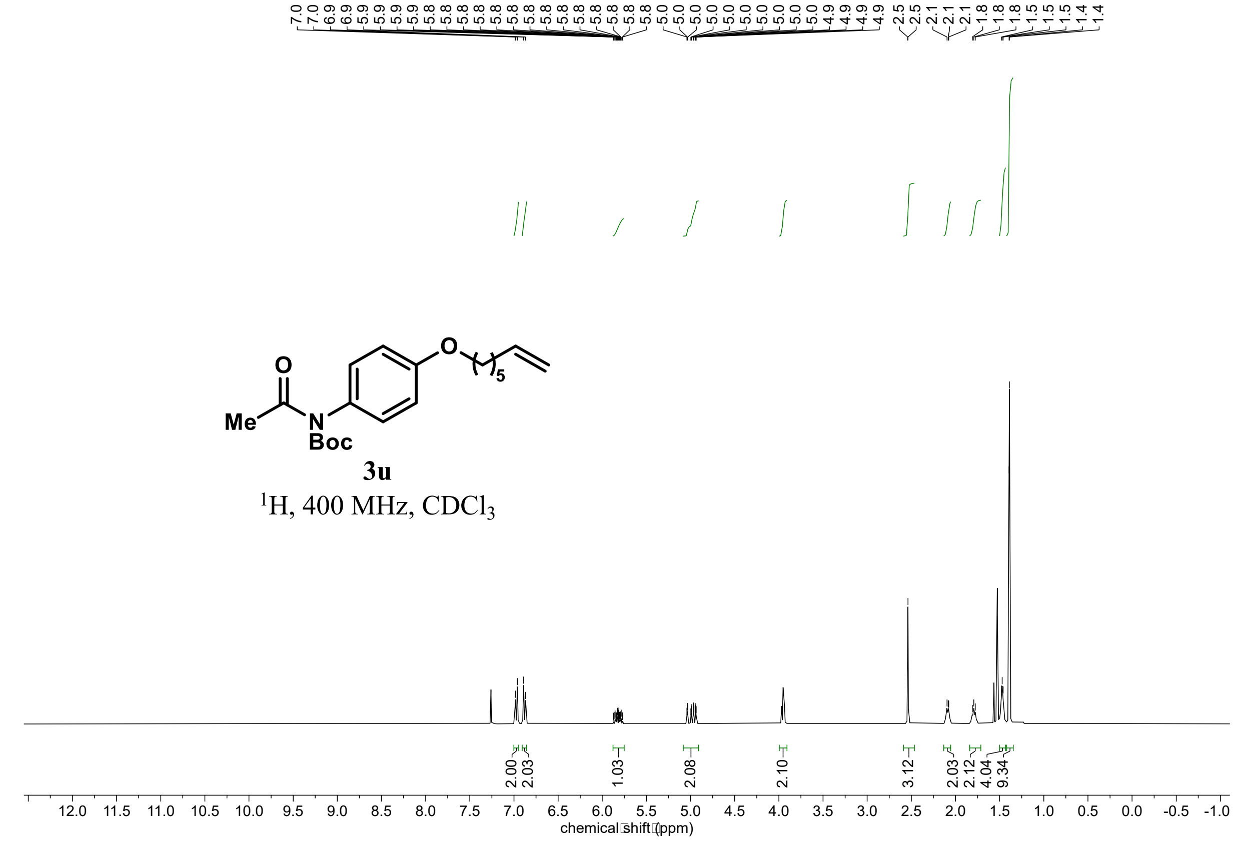

3u

^{1}H, 400 MHz, $CDCl_3$

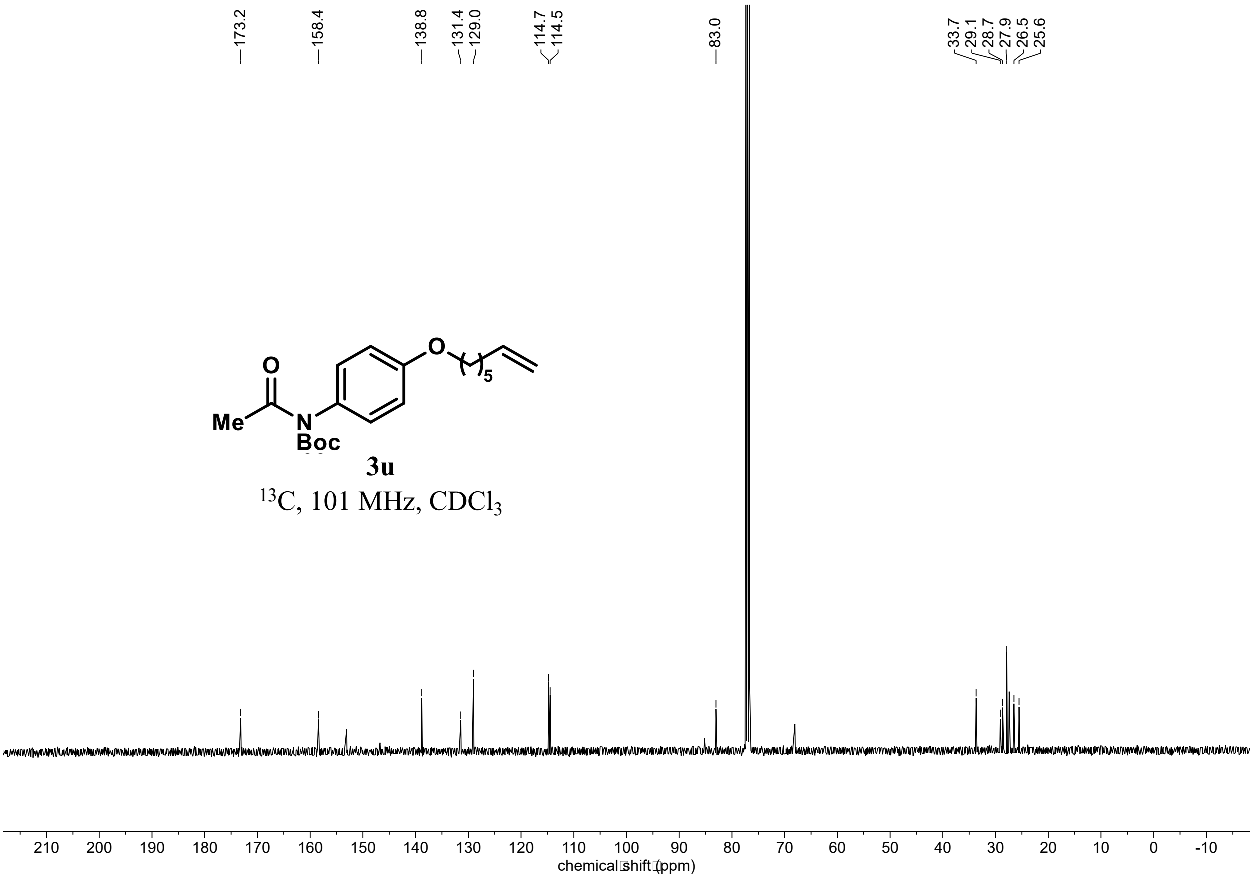

3u

^{13}C, 101 MHz, $CDCl_3$

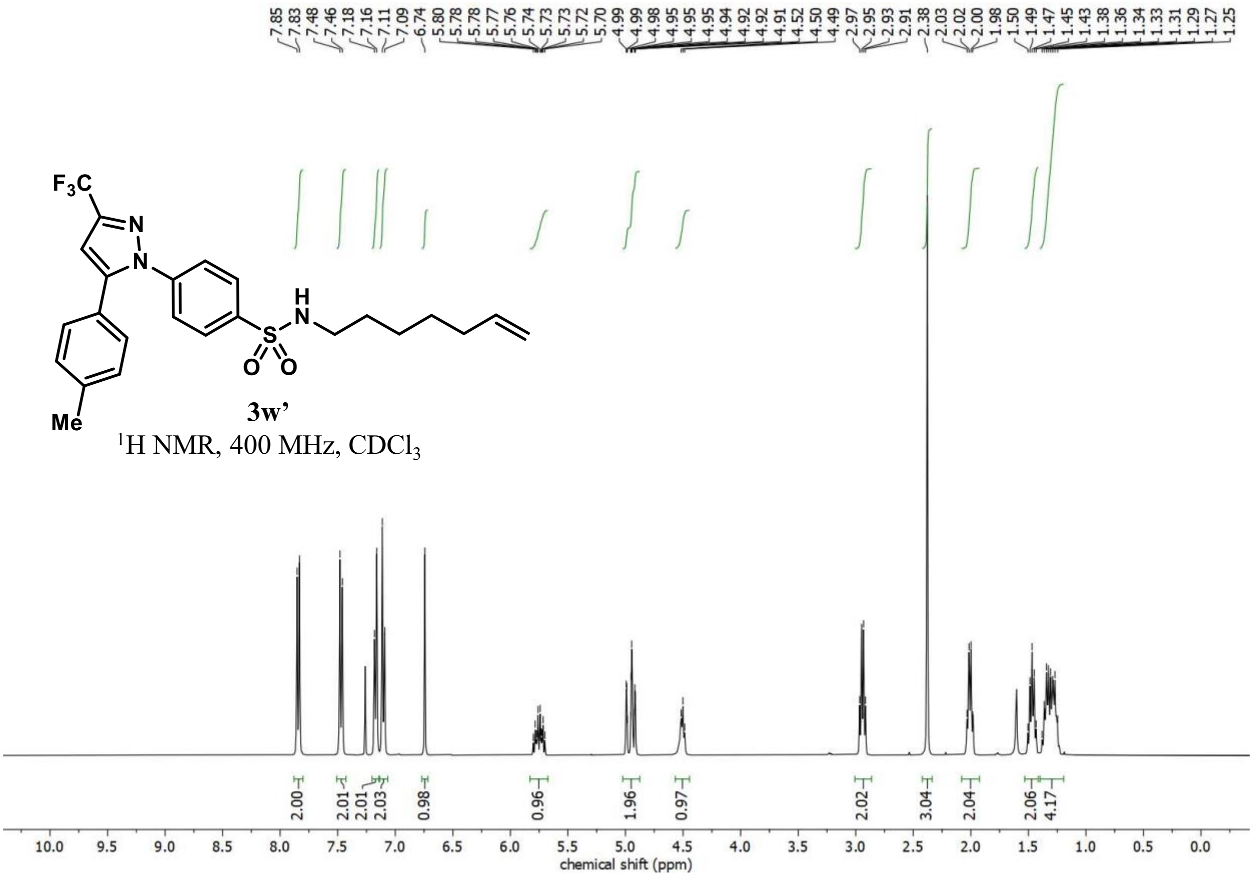

3w'

^{1}H NMR, 400 MHz, CDCl$_3$

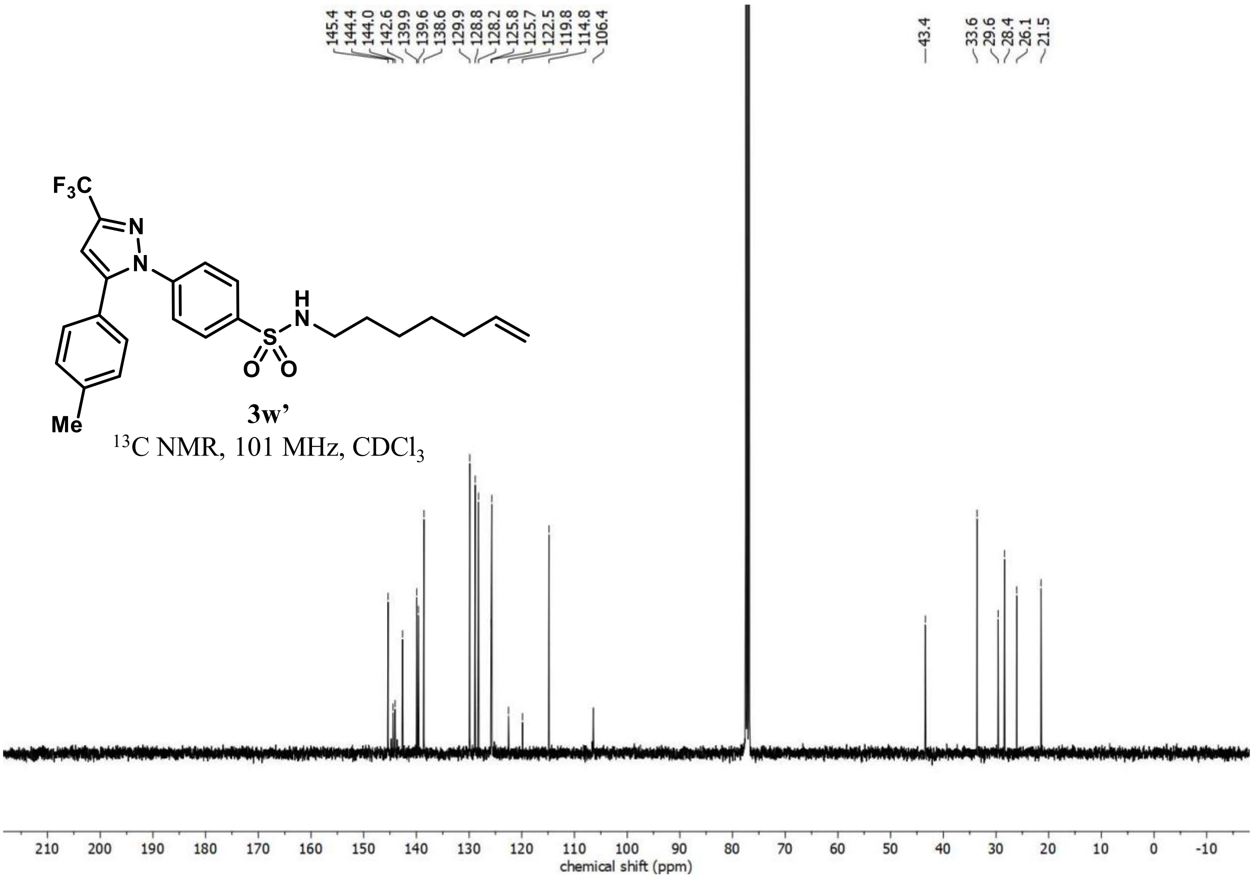

3w'

^{13}C NMR, 101 MHz, CDCl$_3$

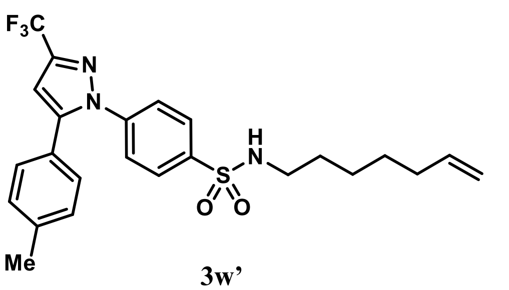

3w'
^{19}F NMR, 376 MHz, $CDCl_3$

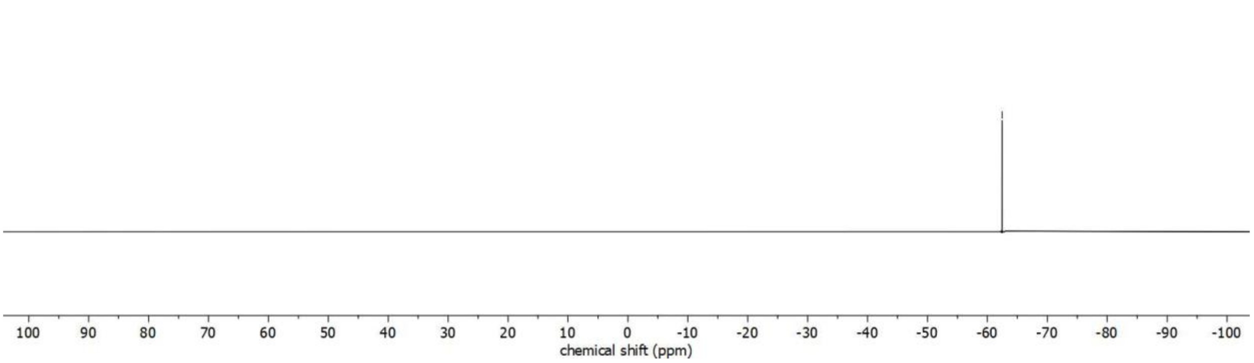

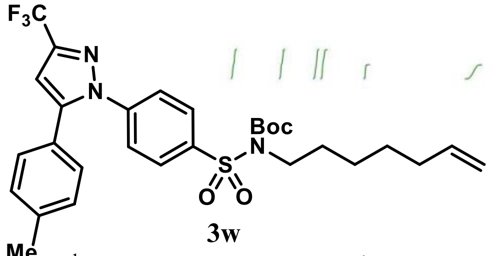

3w
^{1}H NMR, 400 MHz, $CDCl_3$

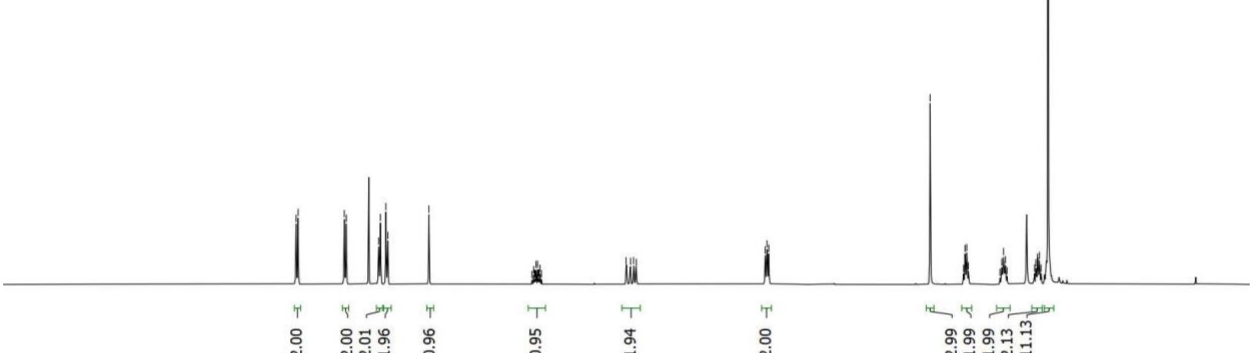

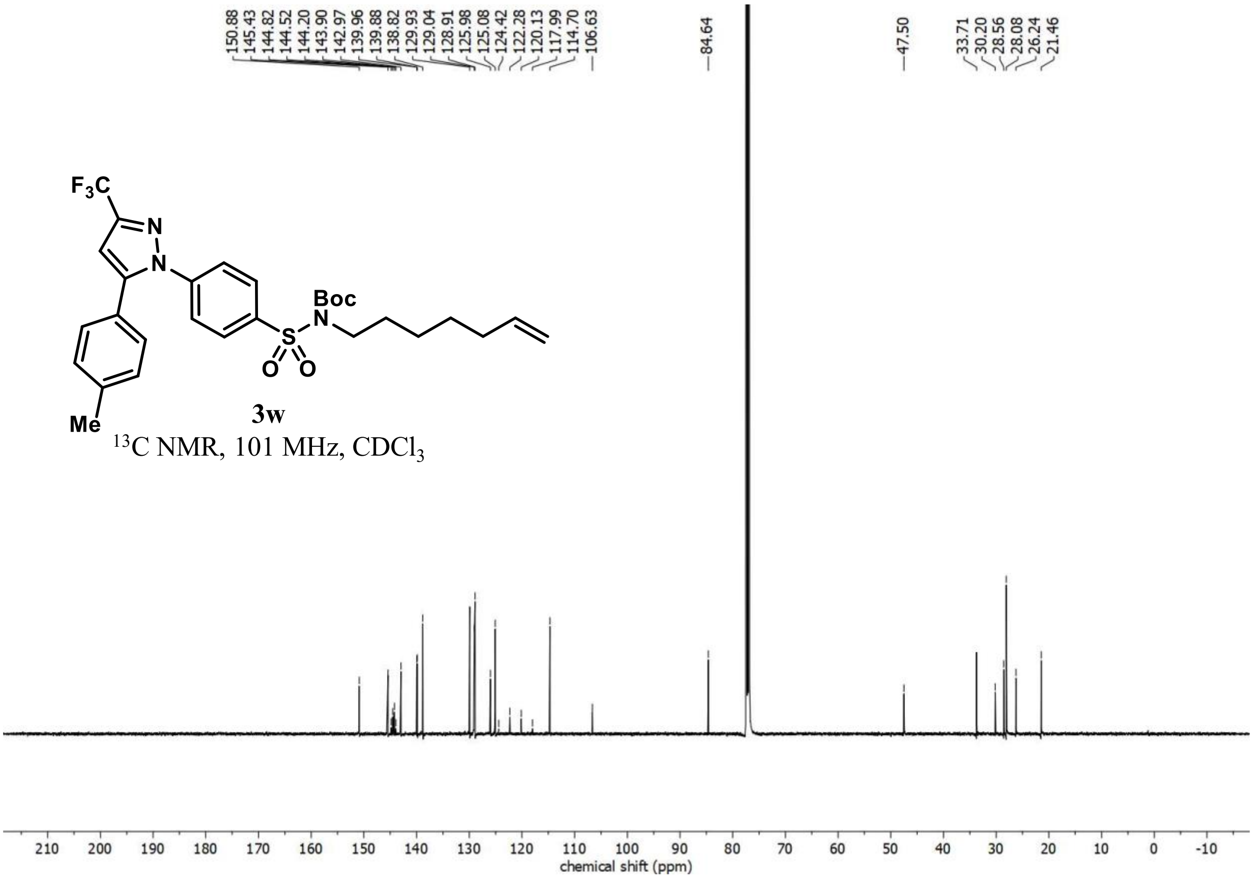
150.88
145.43
144.82
144.52
144.20
143.90
142.97
139.96
139.88
138.82
129.93
129.04
128.91
125.98
125.08
124.42
122.28
120.13
117.99
114.70
106.63
84.64
47.50
33.71
30.20
28.56
28.08
26.24
21.46
F_3C
N
N
Boc
N
S
O
O
Me
3w
^{13}C NMR, 101 MHz, $CDCl_3$
210 200 190 180 170 160 150 140 130 120 110 100 90 80 70 60 50 40 30 20 10 0 -10
chemical shift (ppm)

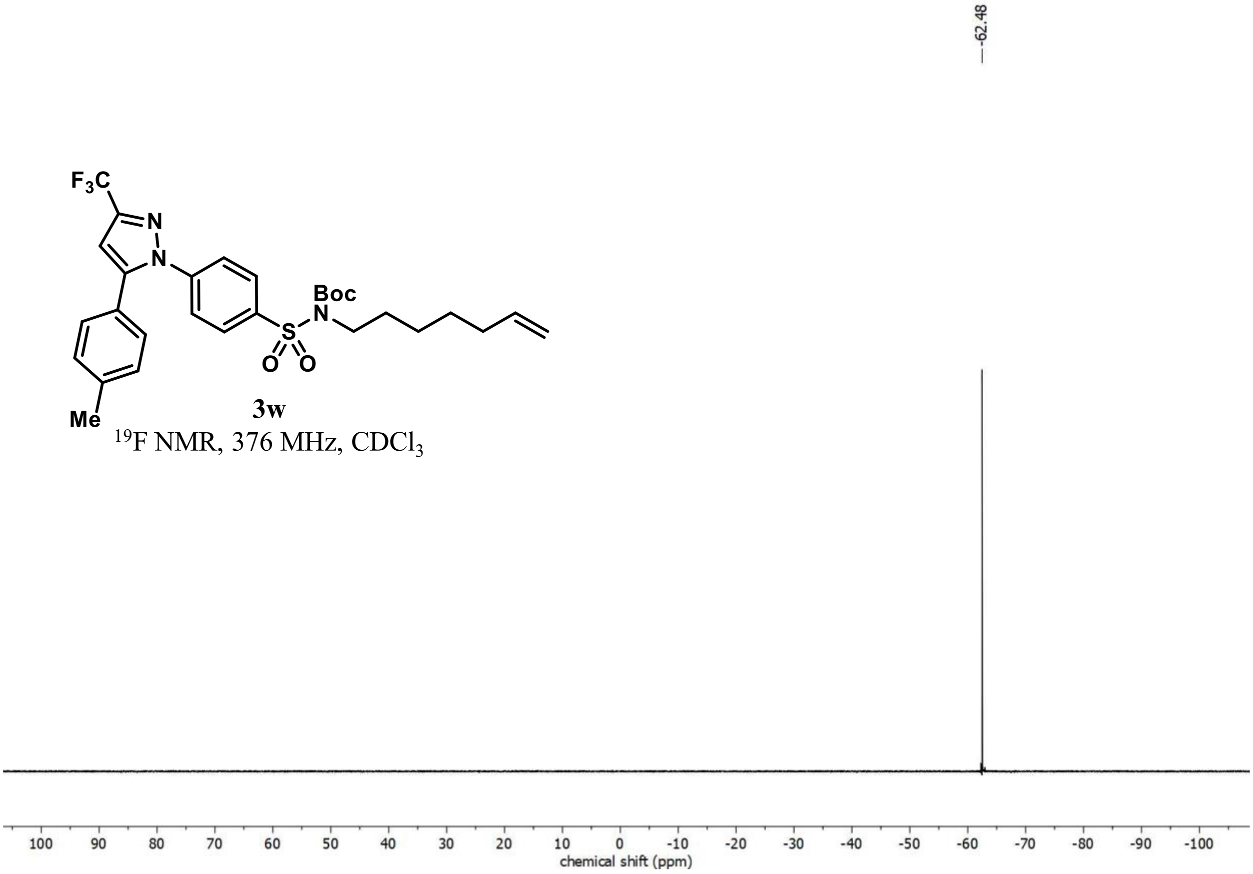
-62.48
F_3C
N
N
Boc
N
S
O
O
Me
3w
^{19}F NMR, 376 MHz, $CDCl_3$
100 90 80 70 60 50 40 30 20 10 0 -10 -20 -30 -40 -50 -60 -70 -80 -90 -100
chemical shift (ppm)

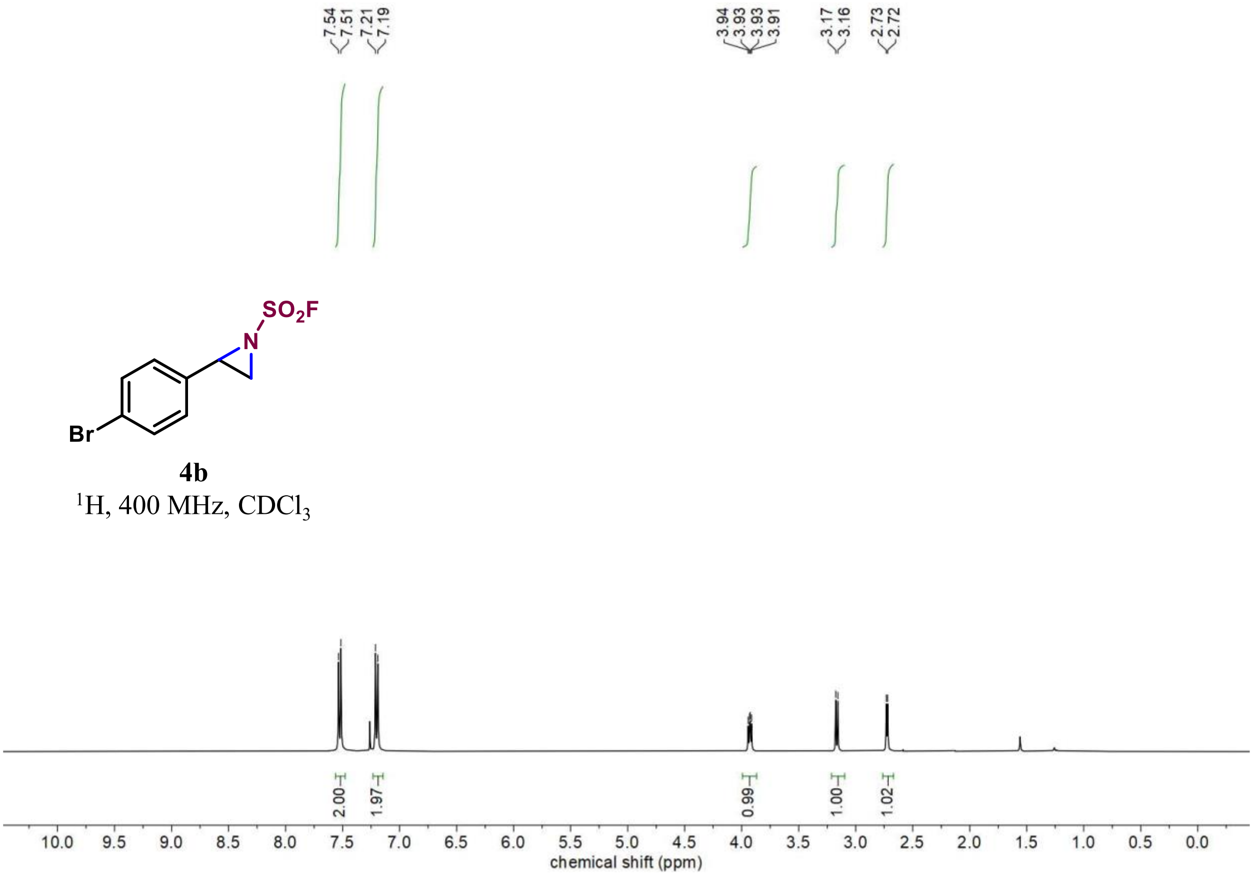

4b

^1H, 400 MHz, $CDCl_3$

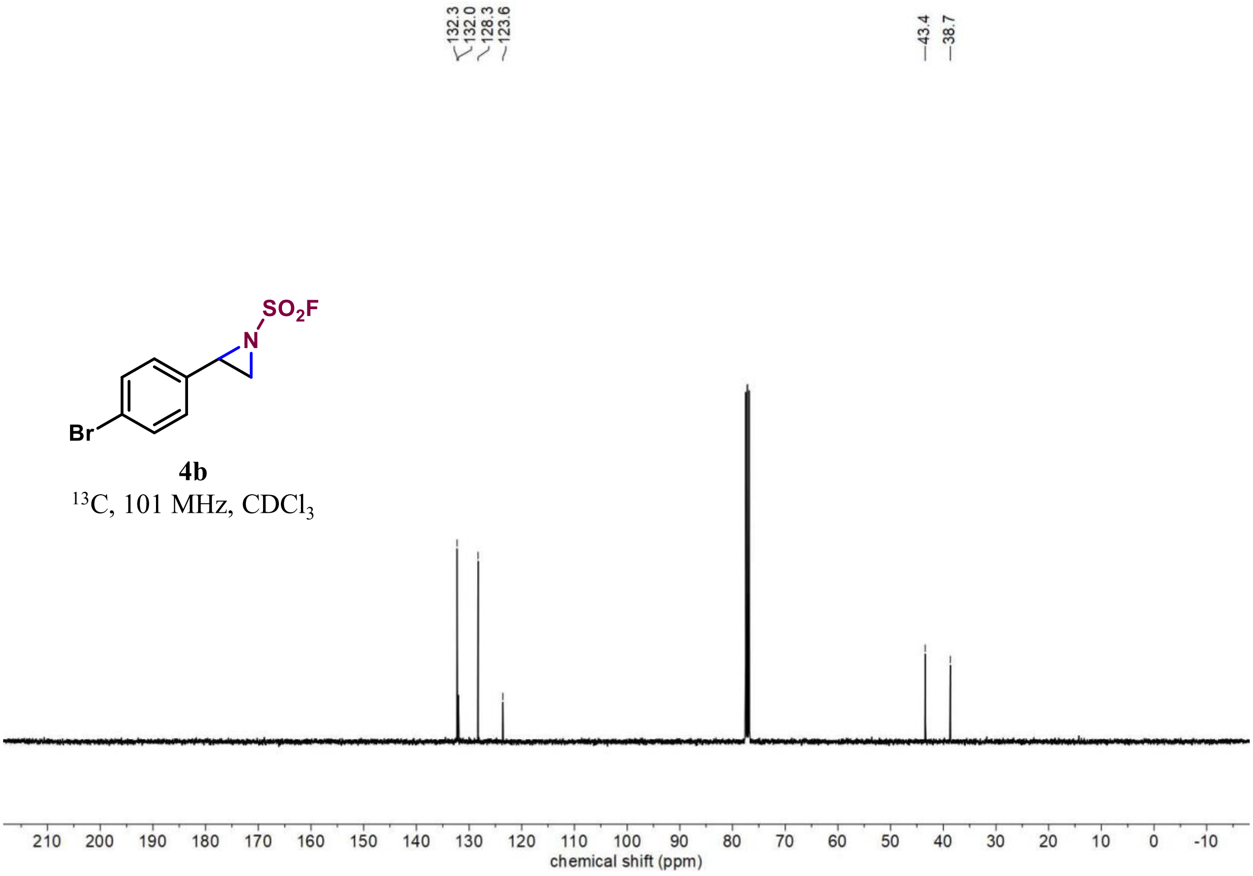

4b

^{13}C, 101 MHz, $CDCl_3$

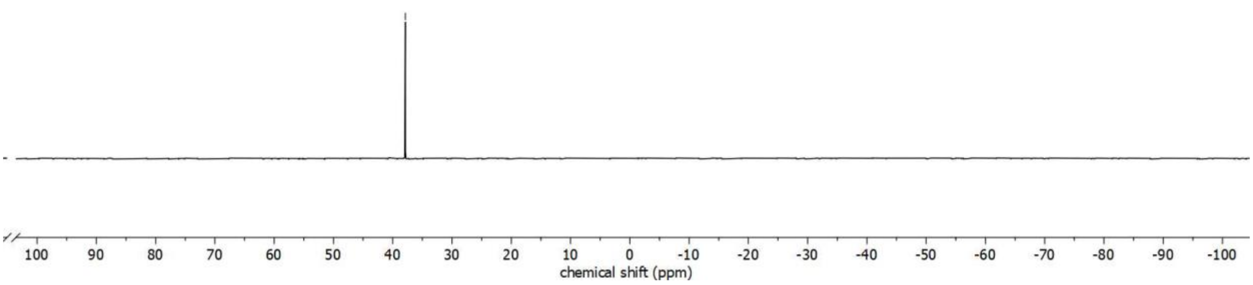

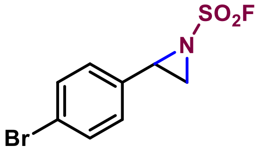

4b

^{19}F, MHz, $CDCl_3$

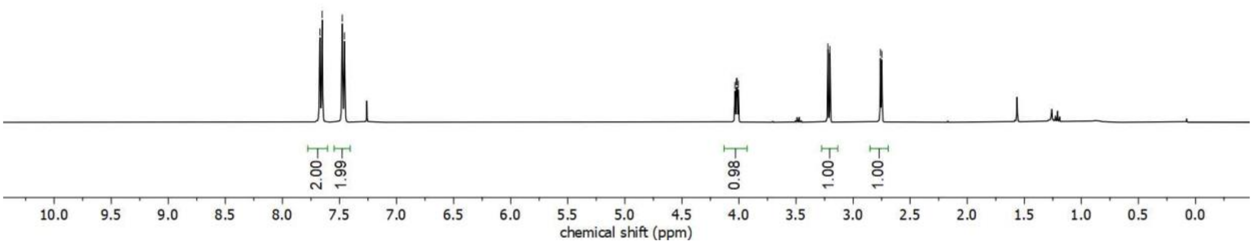

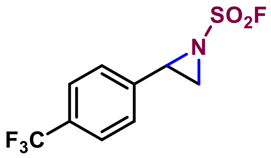

4c

^{1}H, 400 MHz, $CDCl_3$

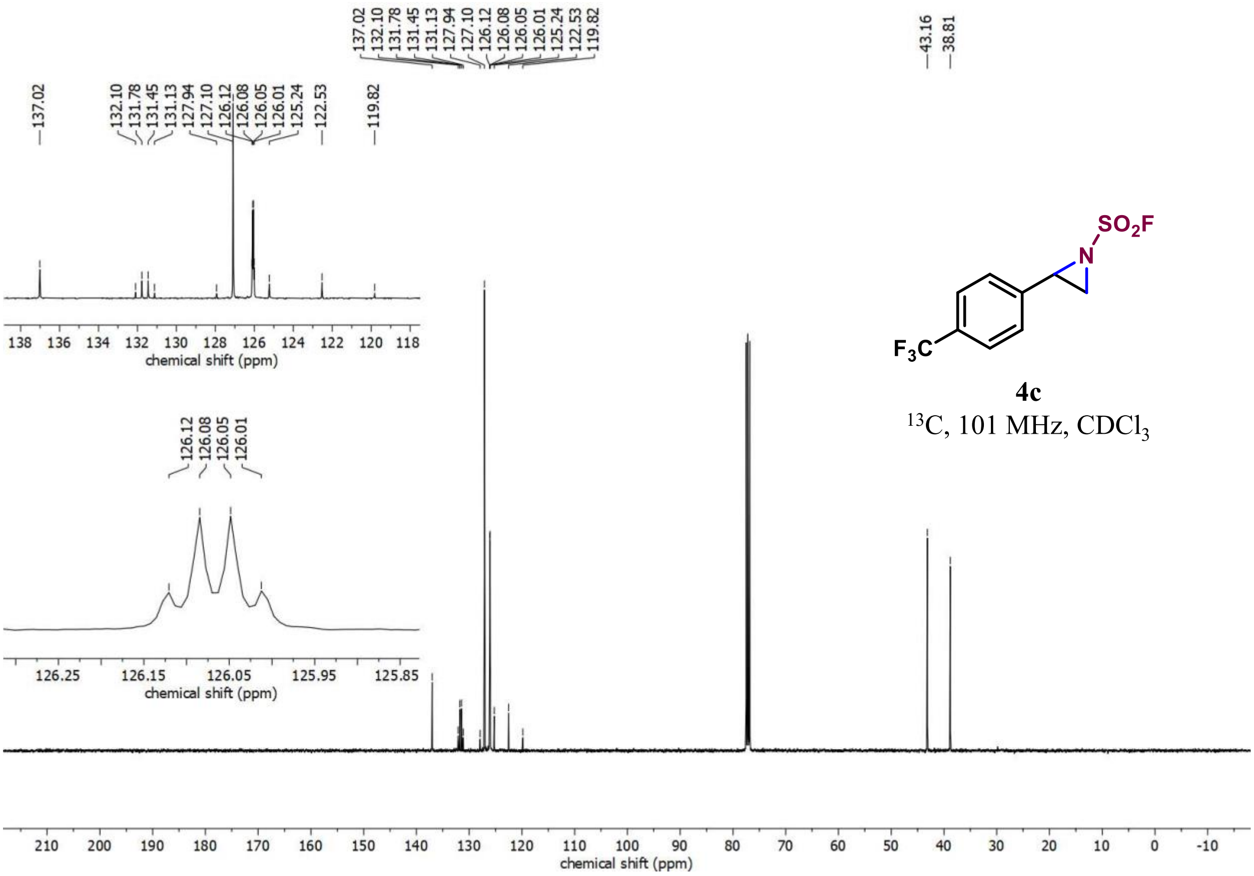

4c

^{13}C, 101 MHz, $CDCl_3$

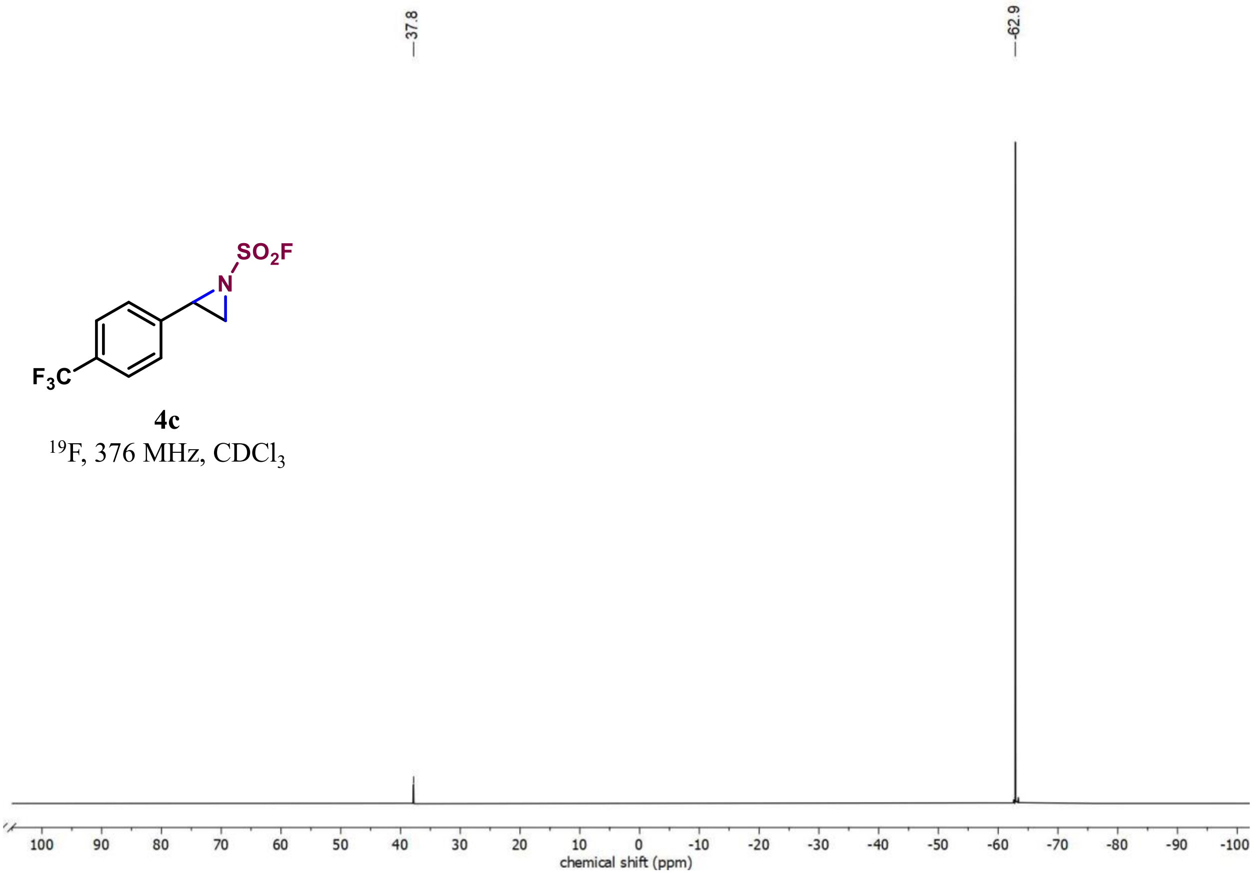

4c

^{19}F, 376 MHz, $CDCl_3$

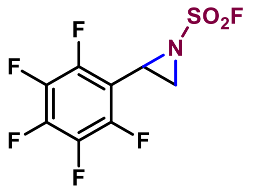

4d

^{1}H, 400 MHz, $CDCl_3$

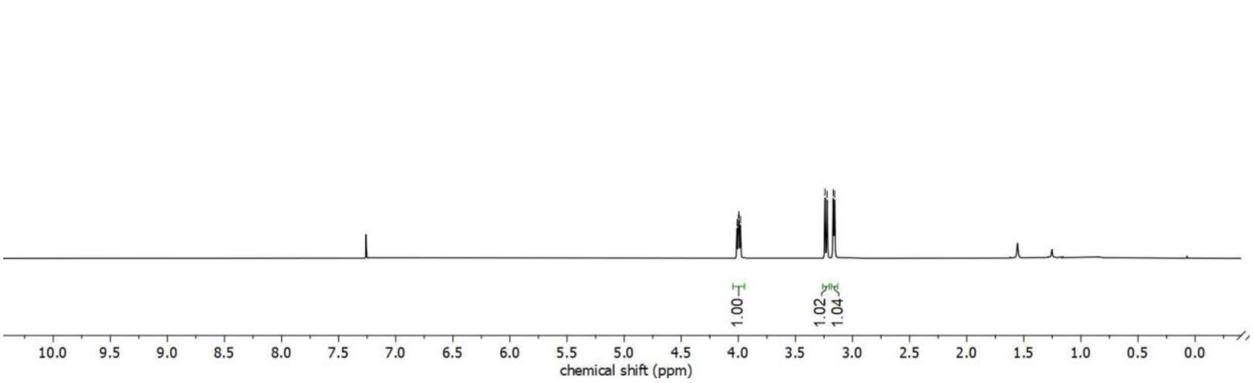

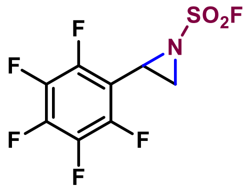

4d

^{13}C, 101 MHz, $CDCl_3$

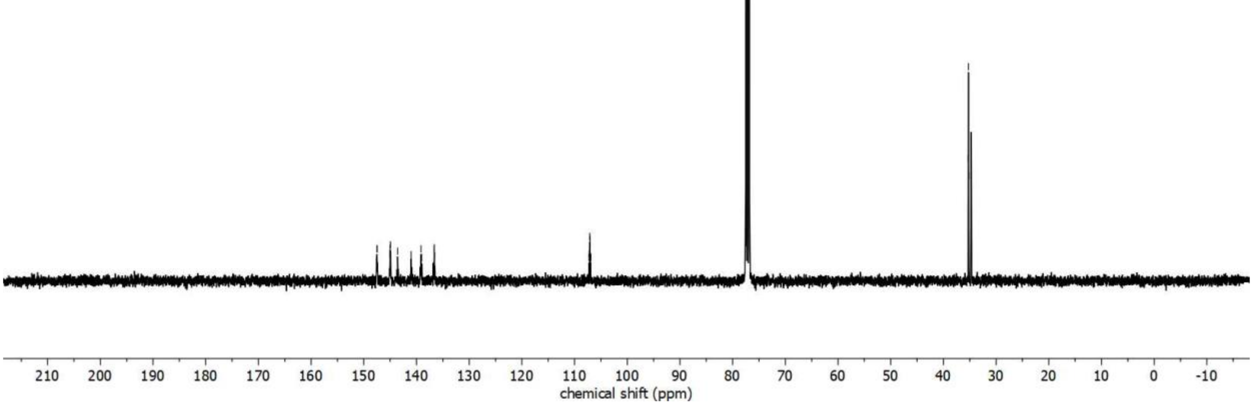

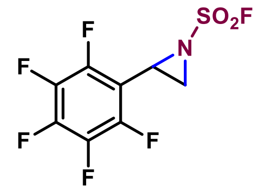

4d
^{19}F, 376 MHz, $CDCl_3$

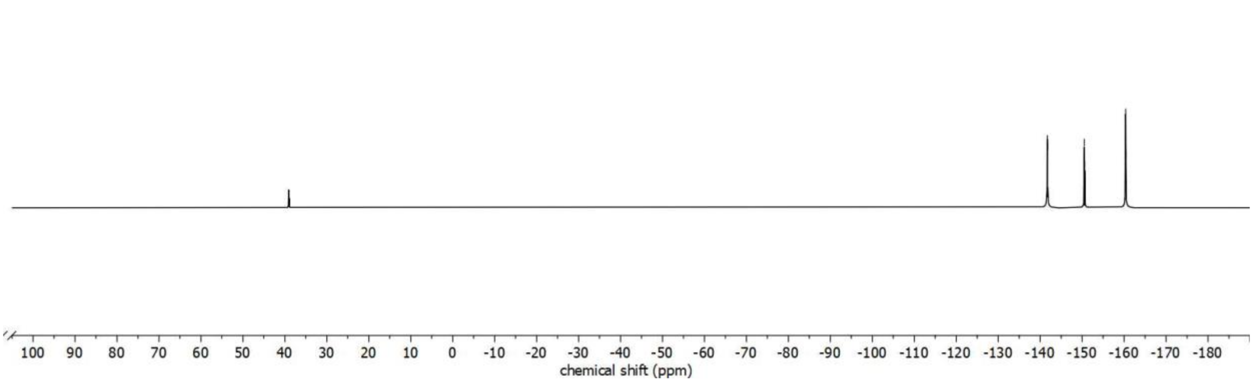

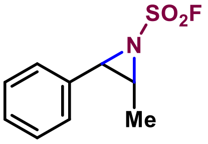

cis-**4e**
^{1}H, 400 MHz, $CDCl_3$

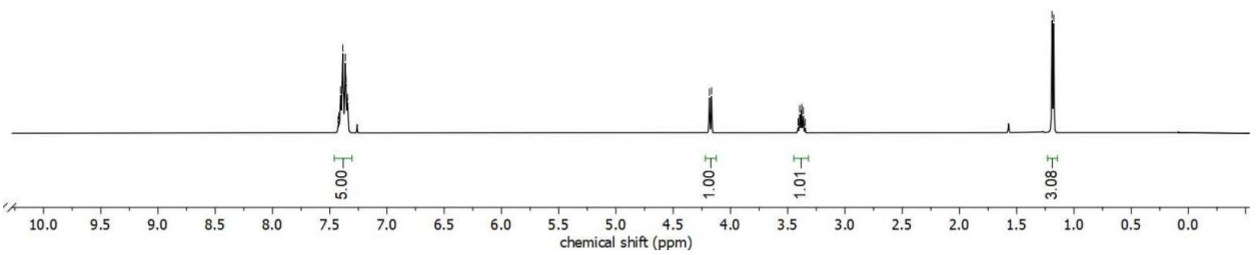

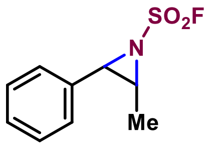

cis-**4e**
^{13}C, 101 MHz, $CDCl_3$

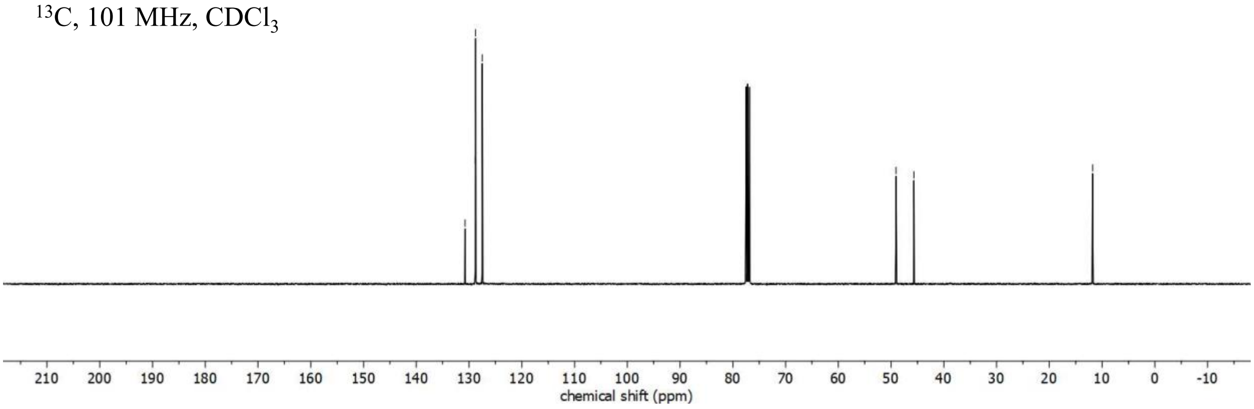

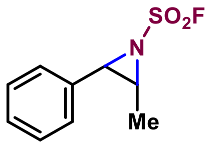

cis-**4e**
^{19}F, 376 MHz, $CDCl_3$

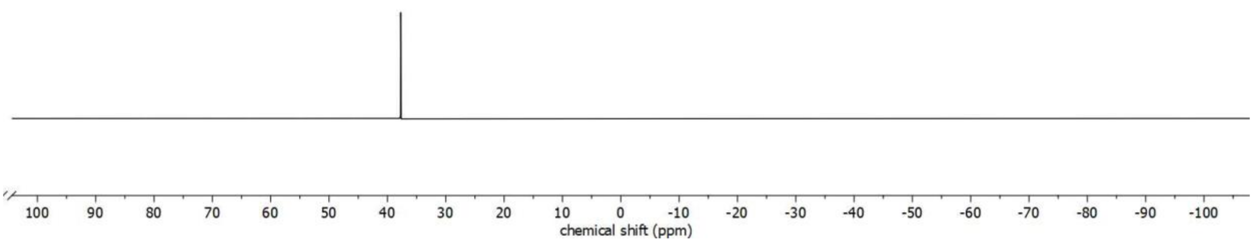

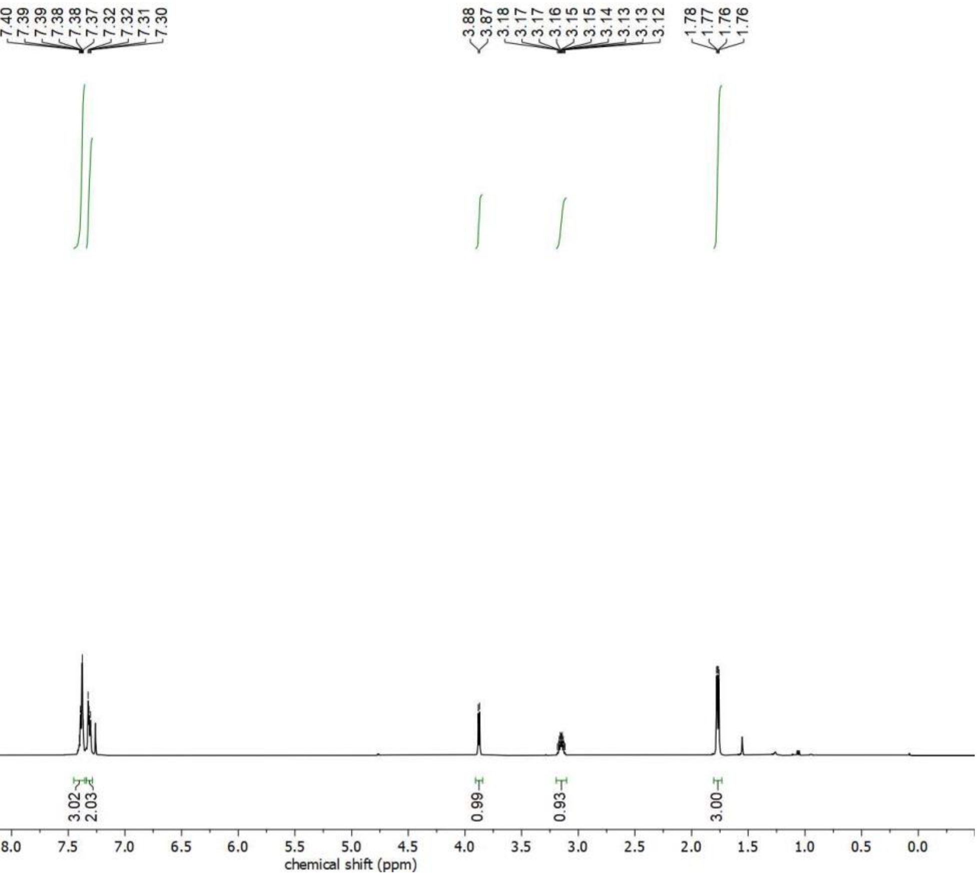

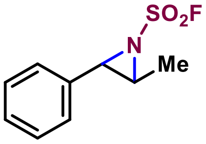

trans-**4e**

^{1}H, 400 MHz, $CDCl_3$

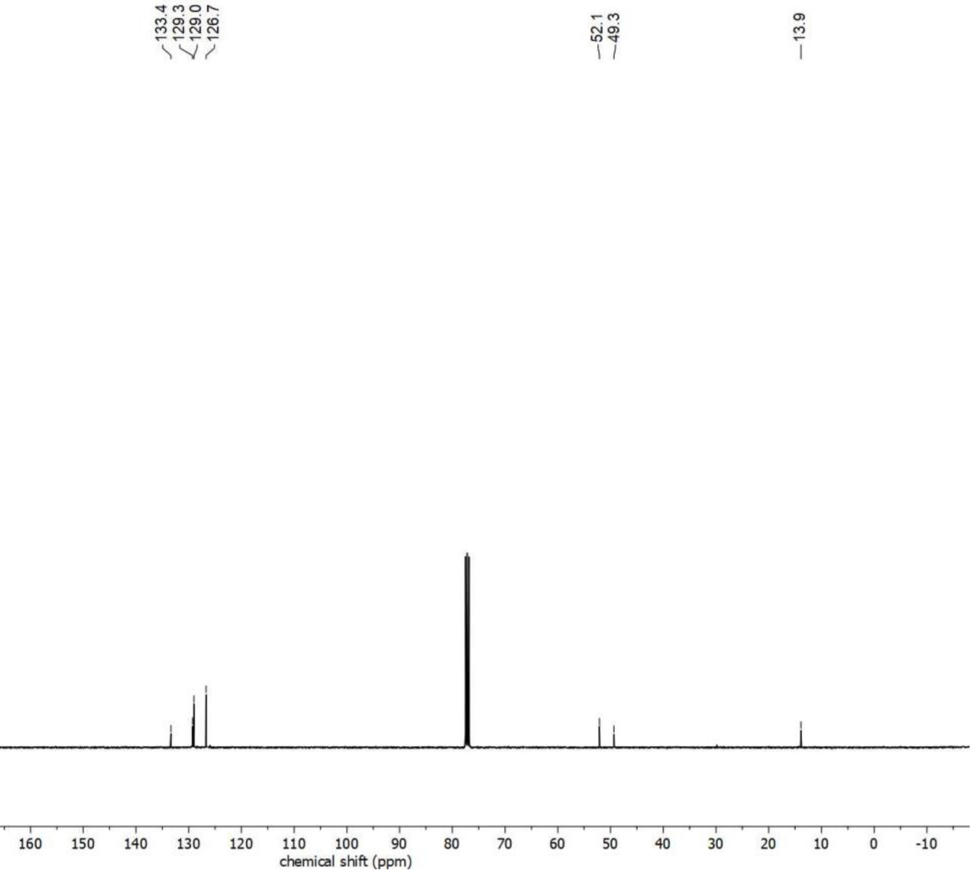

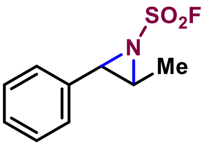

trans-**4e**

^{13}C, 101 MHz, $CDCl_3$

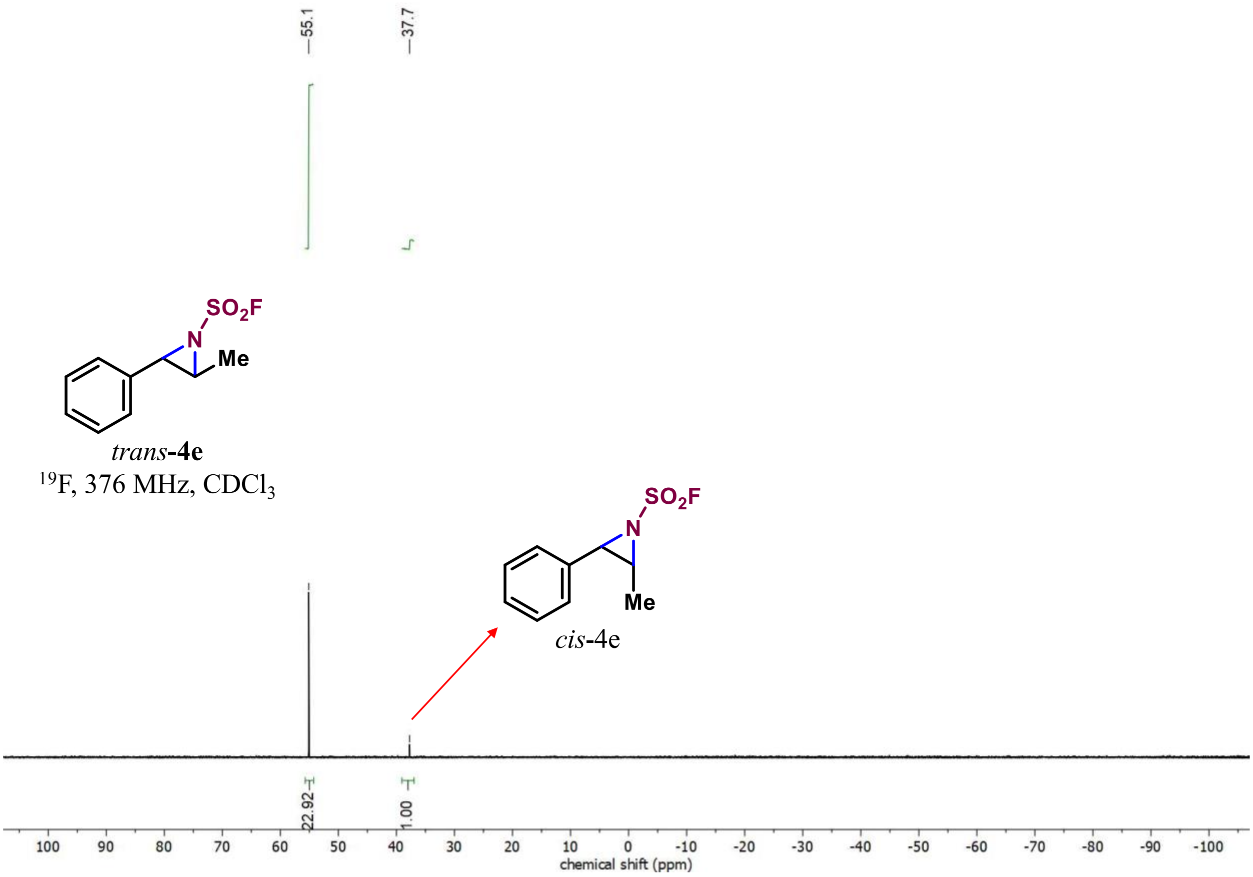
-55.1
-37.7
SO2F
N
Me
trans-4e
19F, 376 MHz, CDCl3
SO2F
N
Me
cis-4e
22.92
1.00
100 90 80 70 60 50 40 30 20 10 0 -10 -20 -30 -40 -50 -60 -70 -80 -90 -100
chemical shift (ppm)

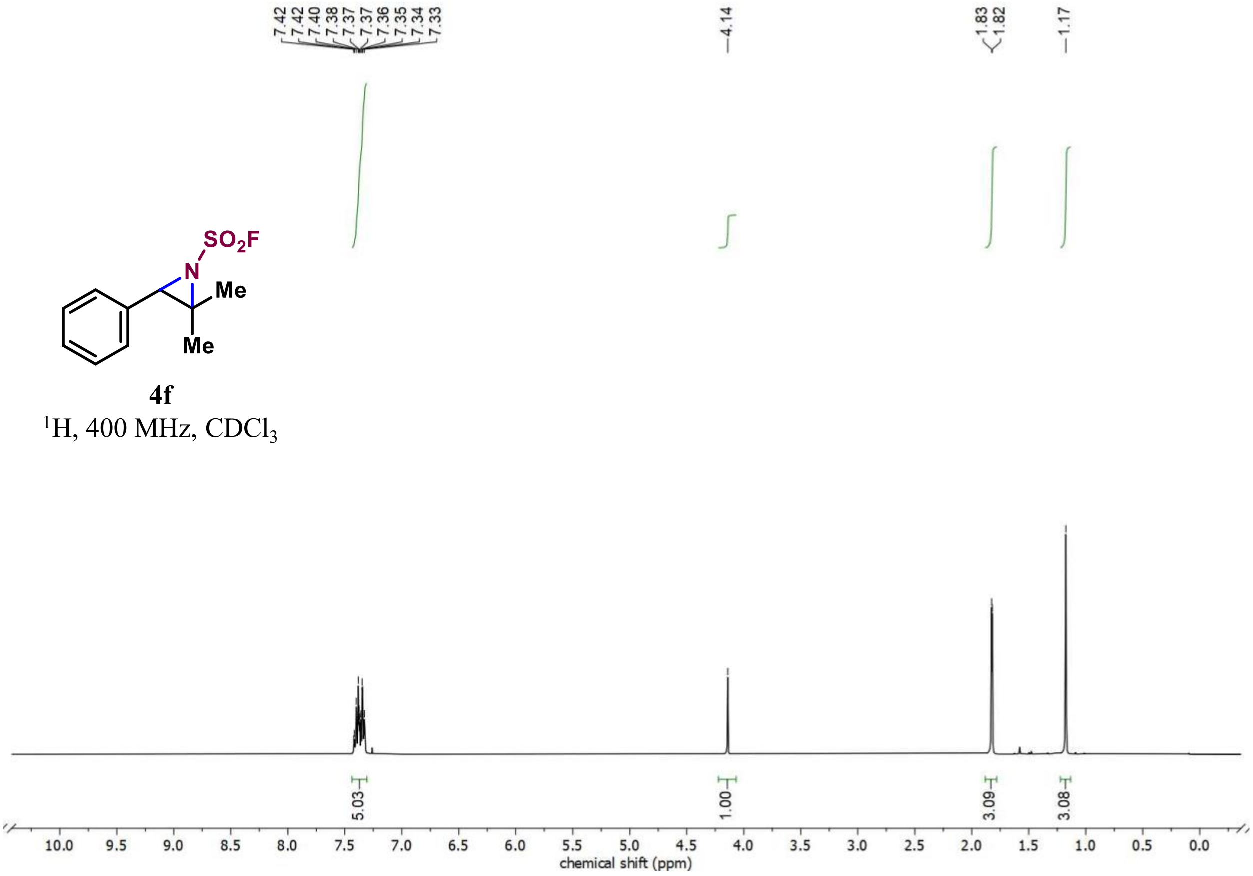
7.42
7.42
7.40
7.38
7.37
7.37
7.36
7.35
7.34
7.33
4.14
1.83
1.82
1.17
SO2F
N
Me
Me
4f
1H, 400 MHz, CDCl3
5.03
1.00
3.09
3.08
10.0 9.5 9.0 8.5 8.0 7.5 7.0 6.5 6.0 5.5 5.0 4.5 4.0 3.5 3.0 2.5 2.0 1.5 1.0 0.5 0.0
chemical shift (ppm)

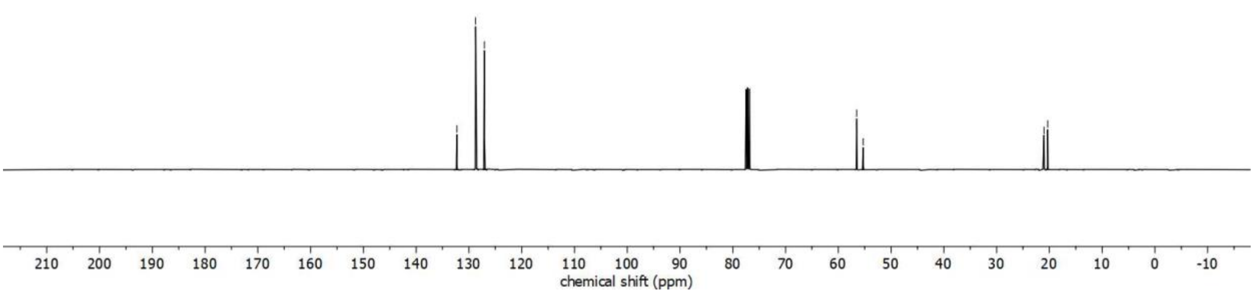

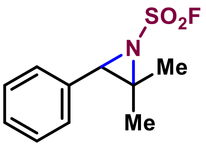

4f

^{13}C, 101 MHz, $CDCl_3$

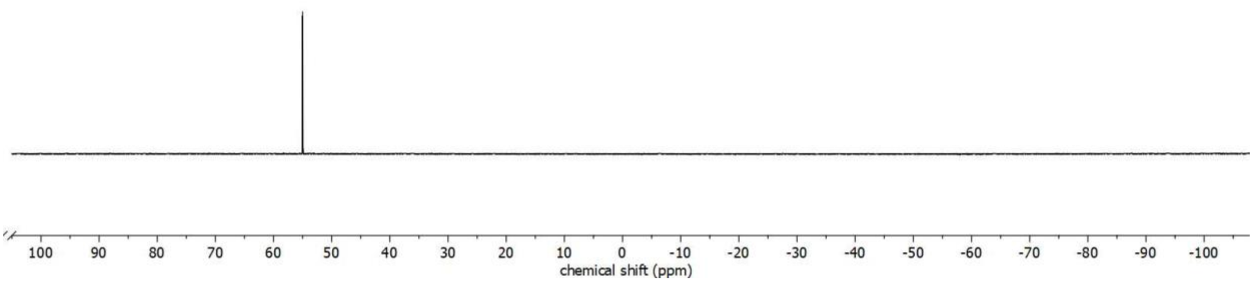

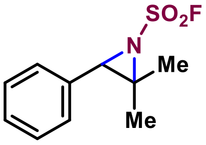

4f

^{19}F, 376 MHz, $CDCl_3$

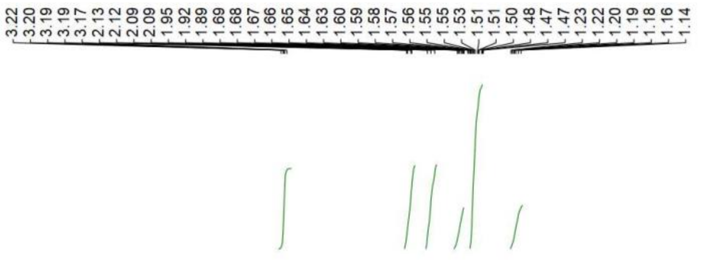

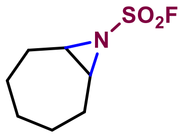

4g

^{1}H, 400 MHz, $CDCl_3$

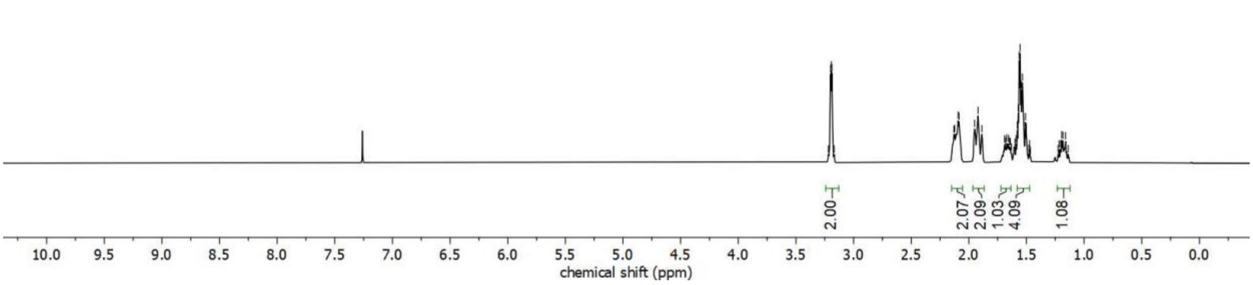

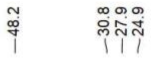

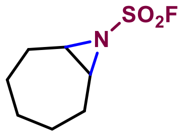

4g

^{13}C, 101 MHz, $CDCl_3$

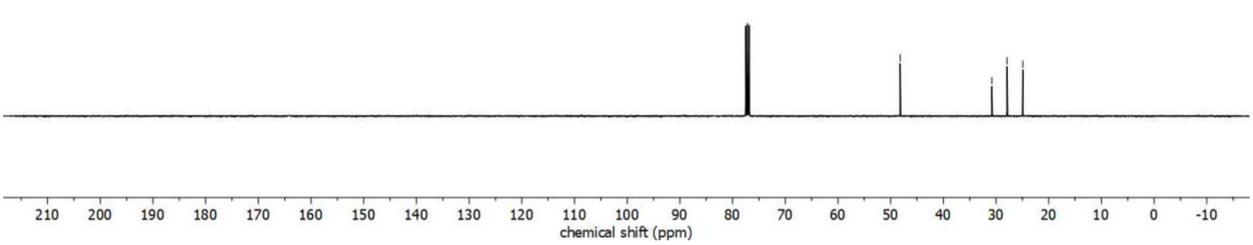

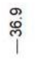

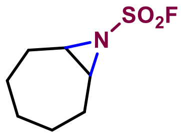

4g

^{19}F, 376 MHz, $CDCl_3$

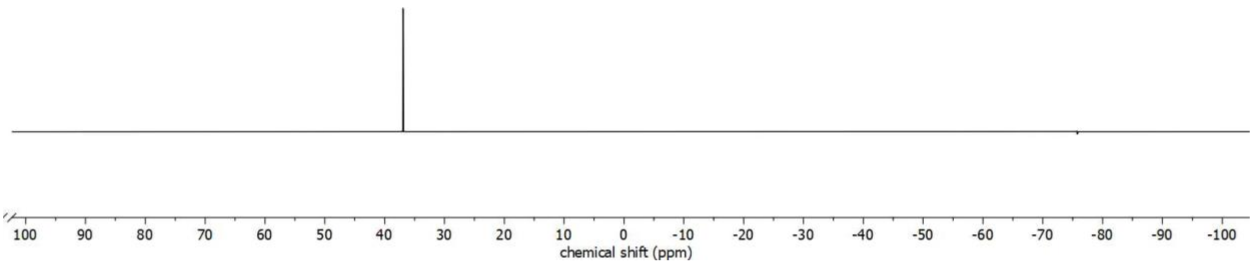

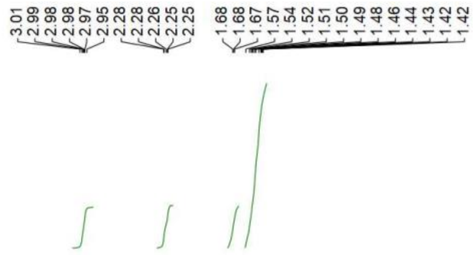

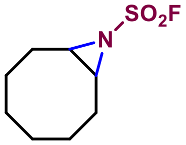

4h

^{1}H, 400 MHz, $CDCl_3$

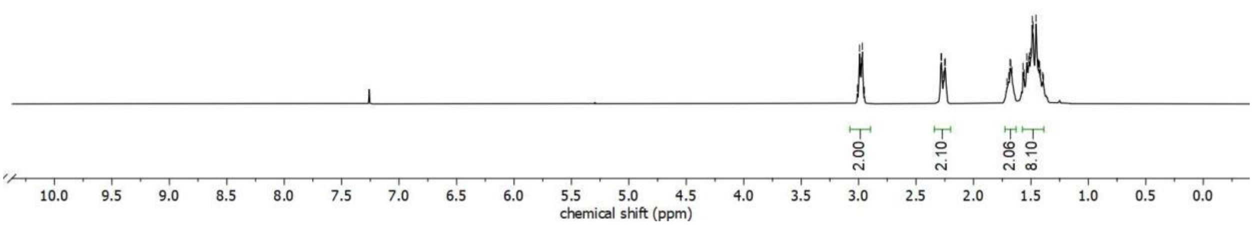

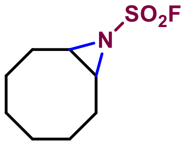

4h
^{13}C, 101 MHz, $CDCl_3$

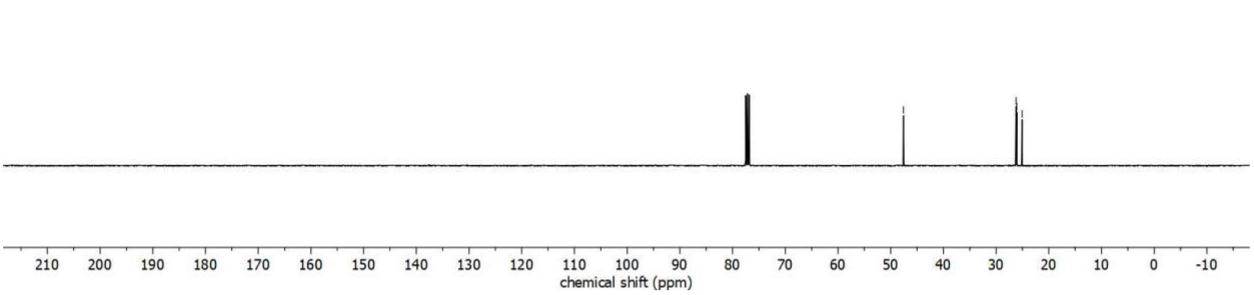

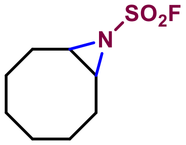

4h
^{19}F, 376 MHz, $CDCl_3$

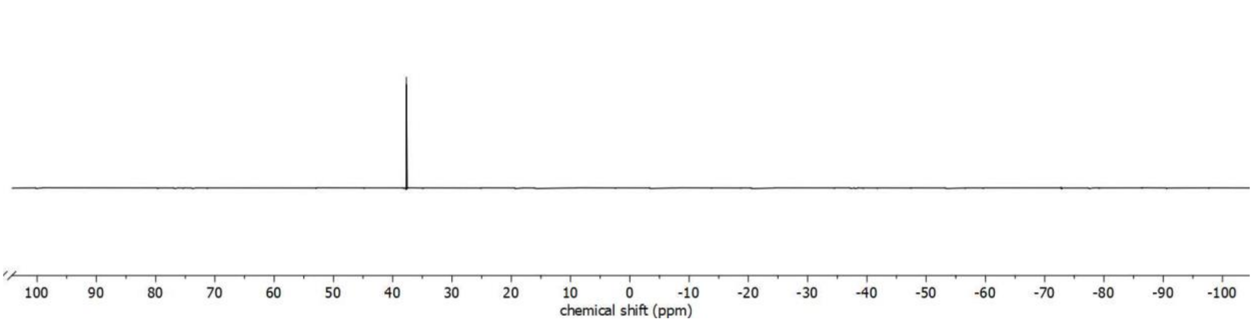

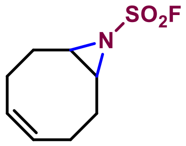

4i

^{1}H, 400 MHz, $CDCl_3$

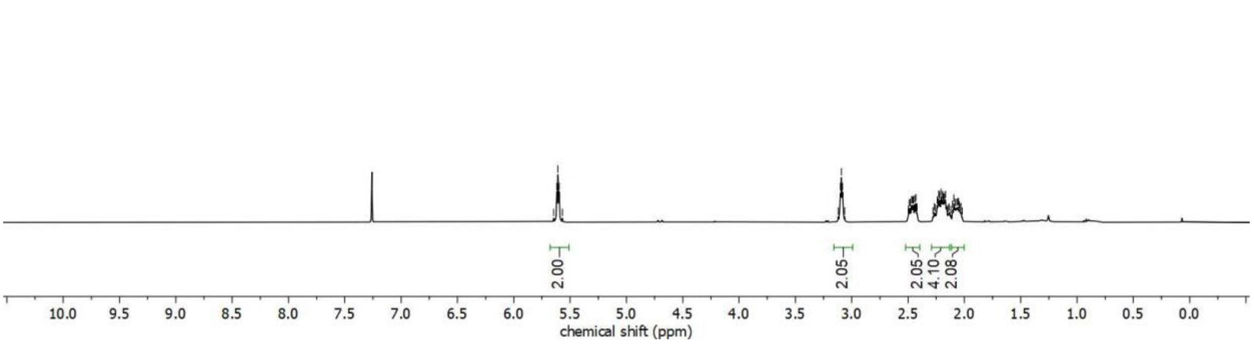

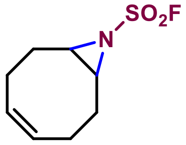

4i

^{13}C, 101 MHz, $CDCl_3$

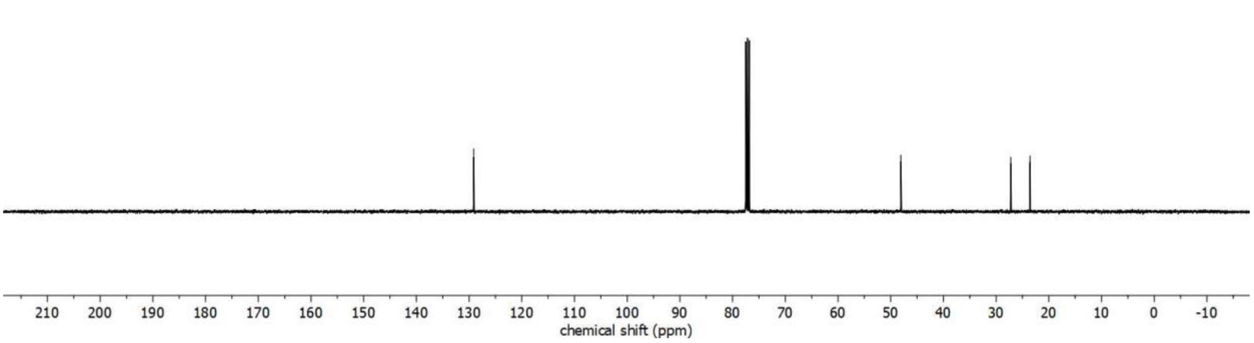

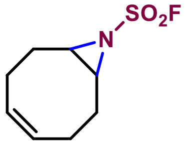

4i

^{19}F, 376 MHz, $CDCl_3$

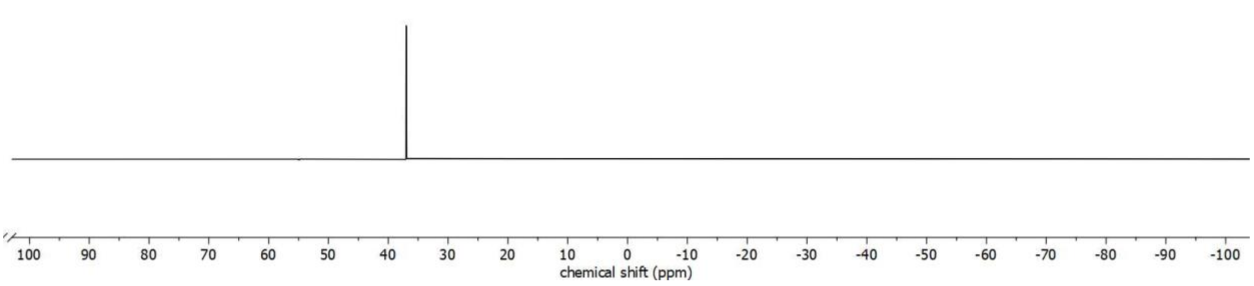

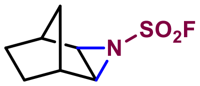

4j

^{1}H, 400 MHz, $CDCl_3$

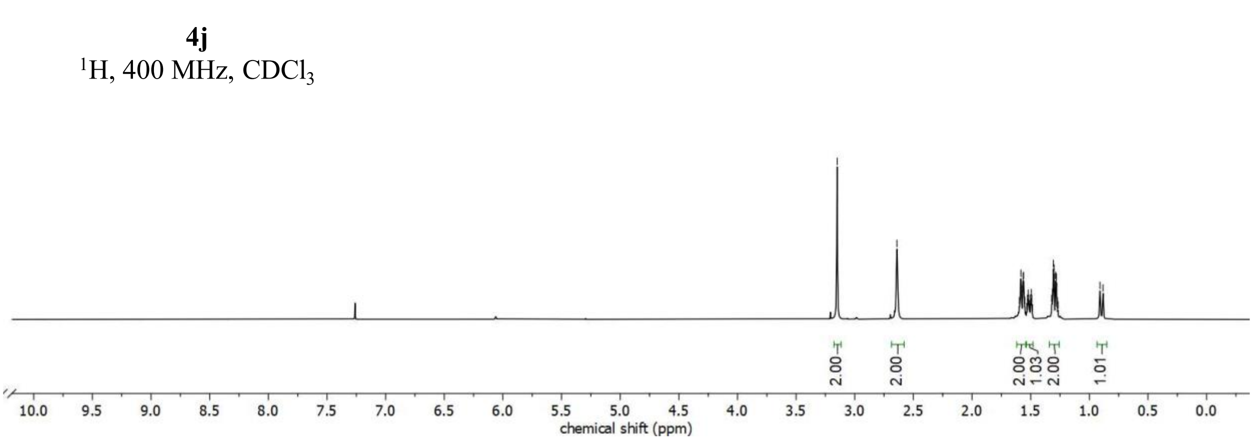

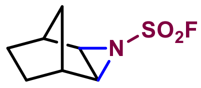

4j

^{13}C, 101 MHz, $CDCl_3$

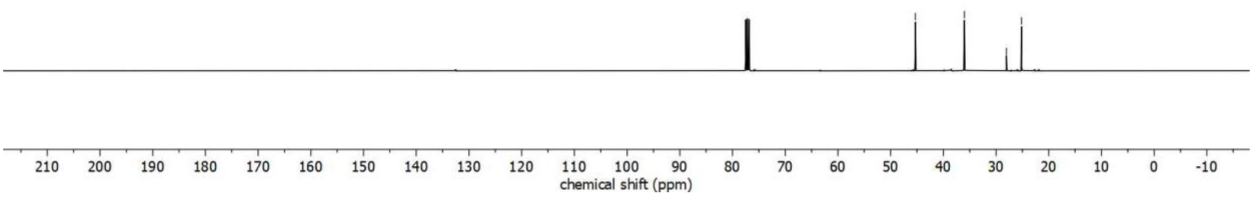

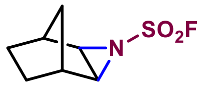

4j

^{19}F, 376 MHz, $CDCl_3$

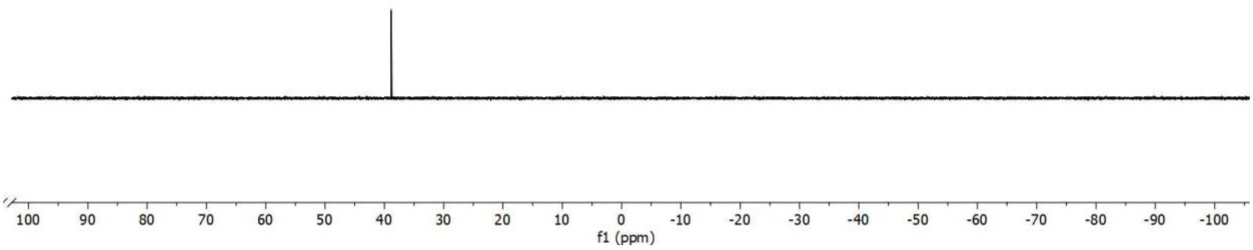

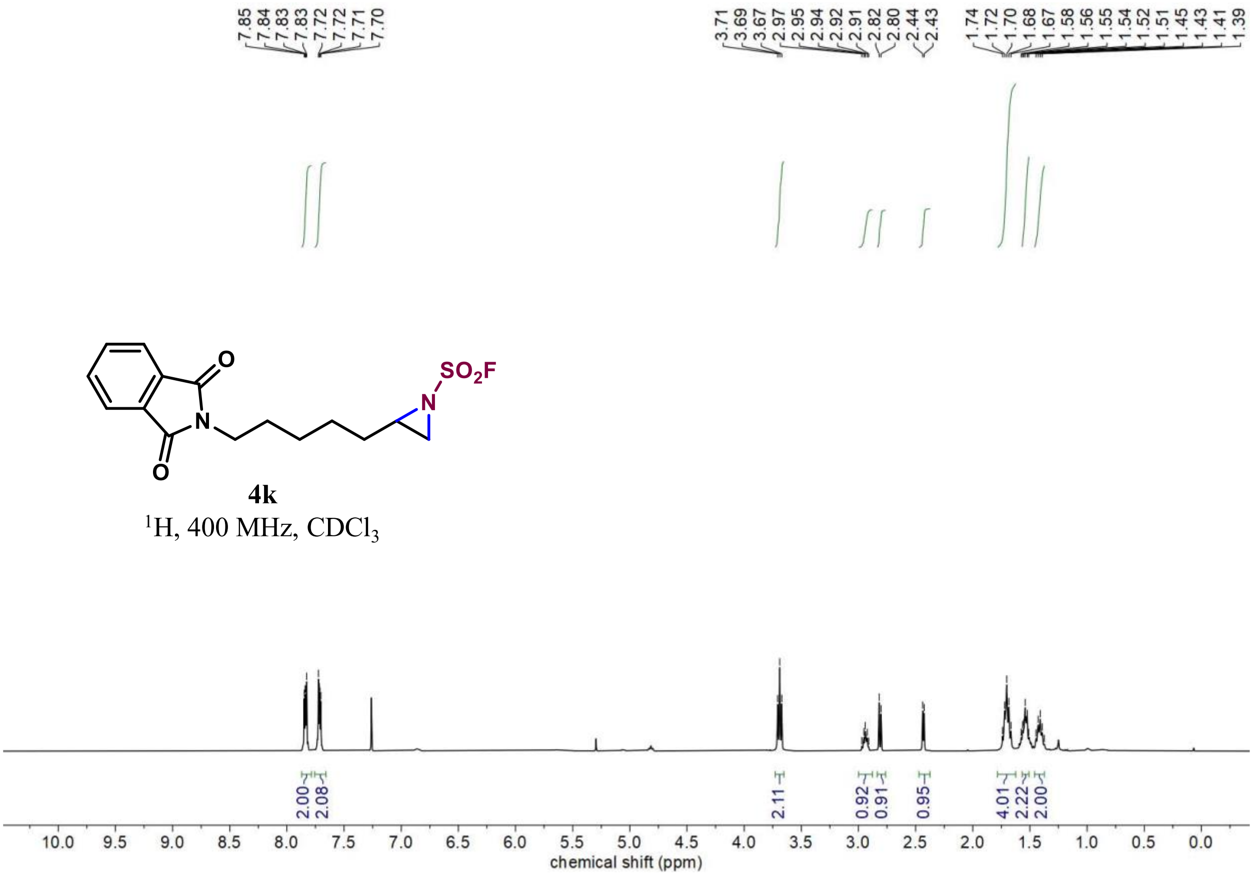

4k

^{1}H, 400 MHz, $CDCl_3$

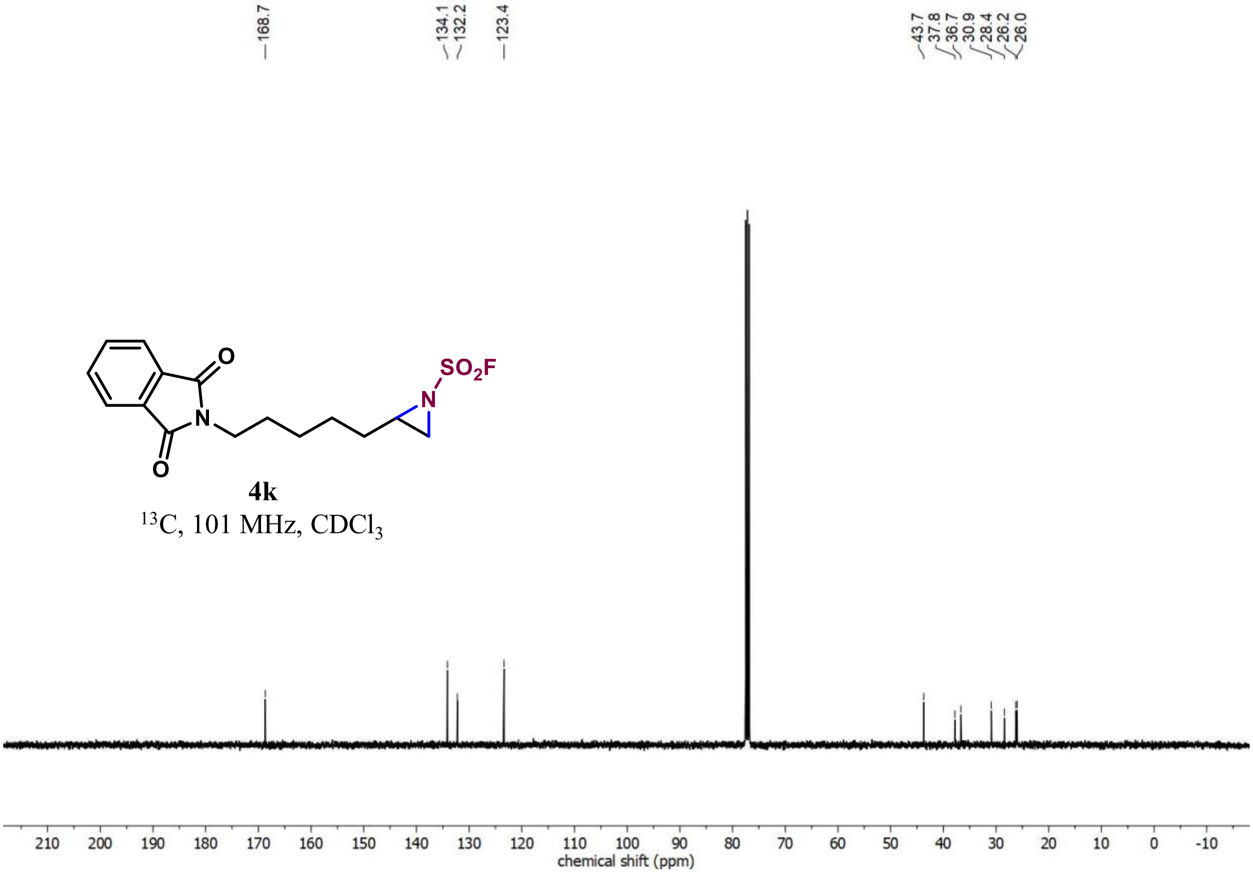

4k

^{13}C, 101 MHz, $CDCl_3$

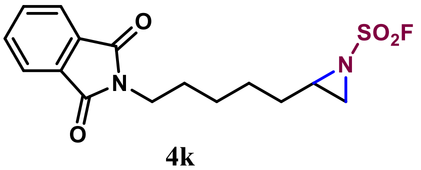

4k

^{19}F, 376 MHz, $CDCl_3$

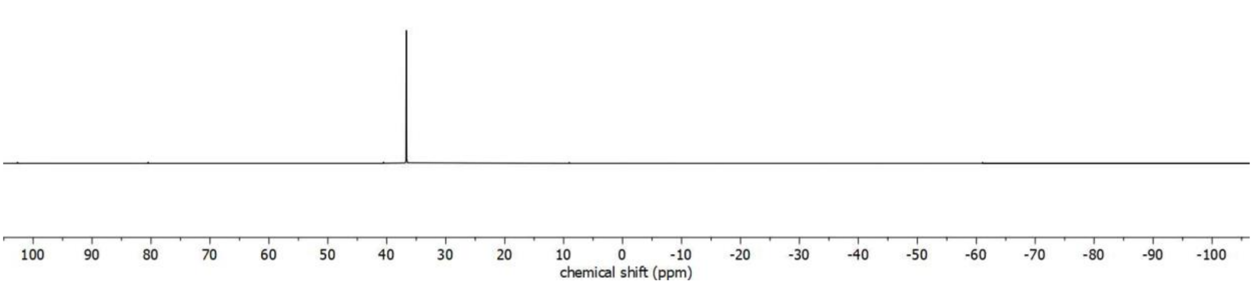

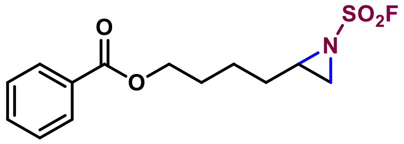

4l

^{1}H, 400 MHz, $CDCl_3$

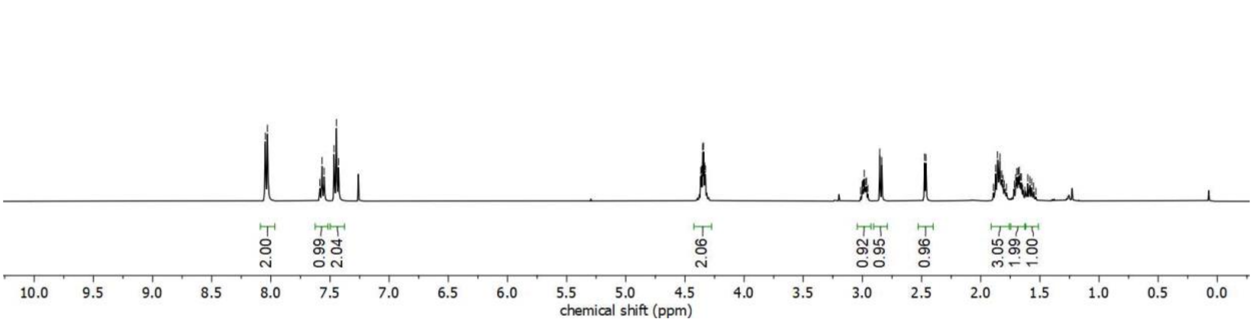

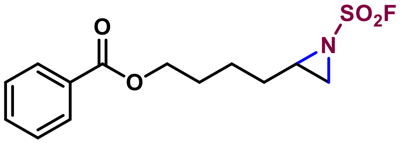

4l

^{13}C, 101 MHz, $CDCl_3$

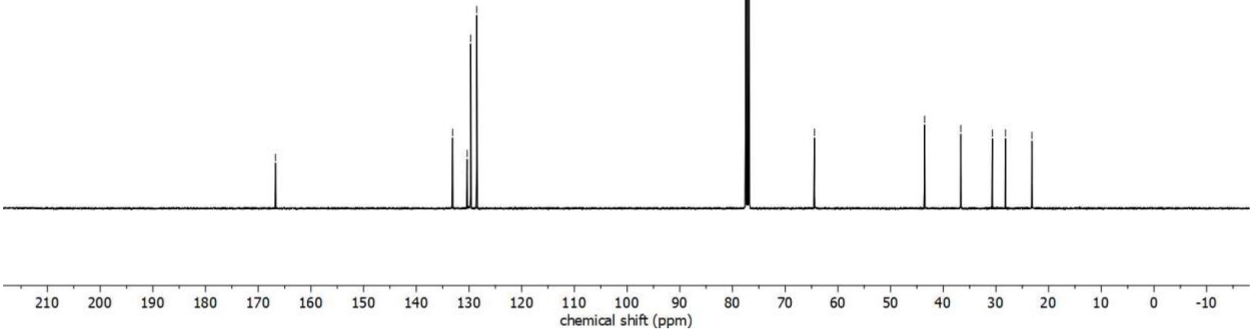

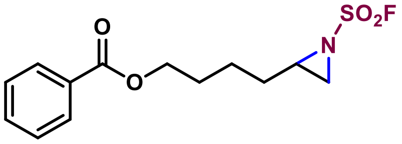

4l

^{19}F, 376 MHz, $CDCl_3$

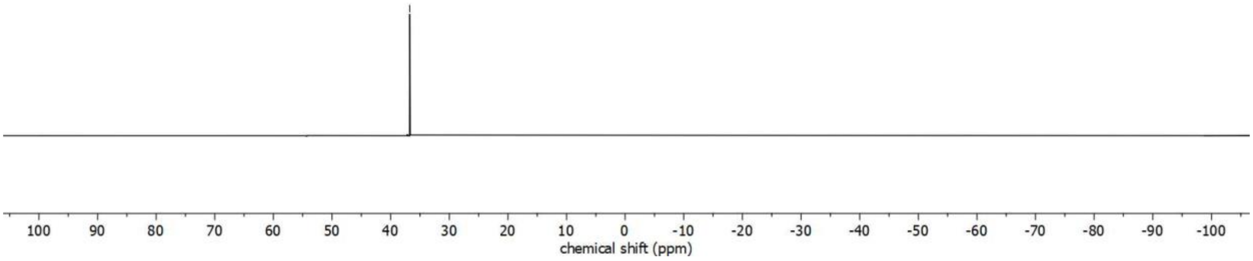

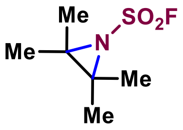

4m

^{1}H, 400 MHz, $CDCl_3$

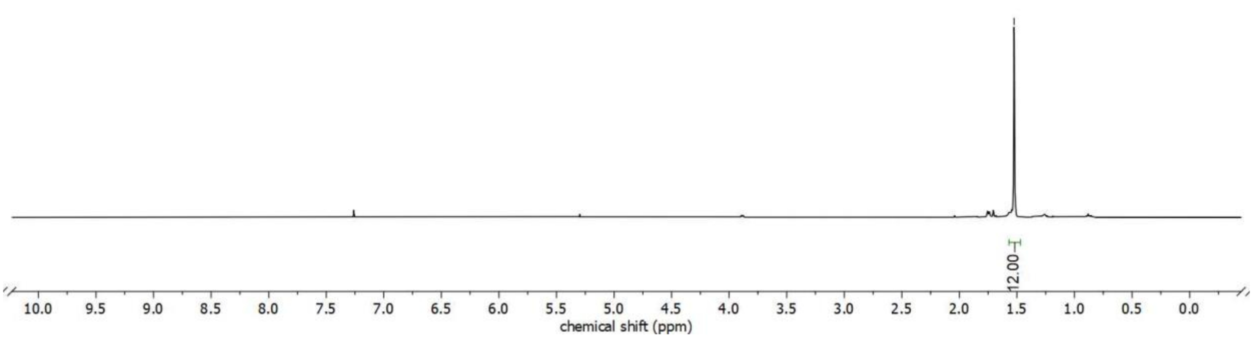

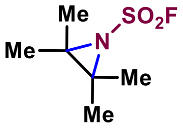

4m

^{13}C, 101 MHz, $CDCl_3$

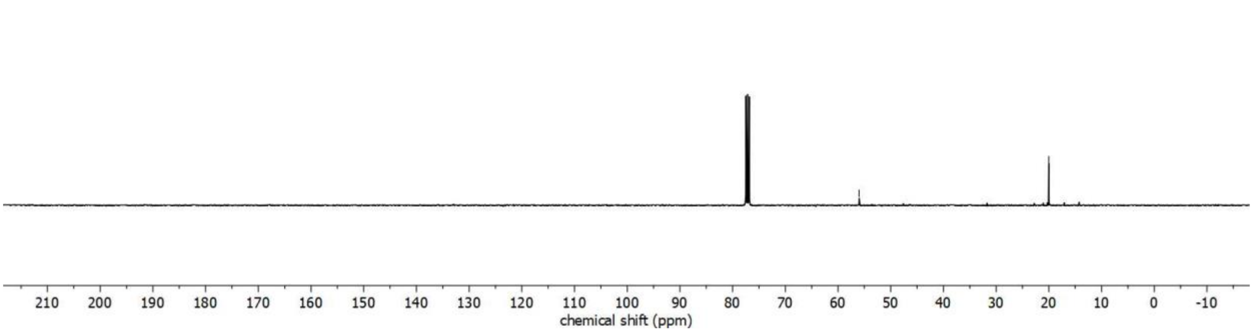

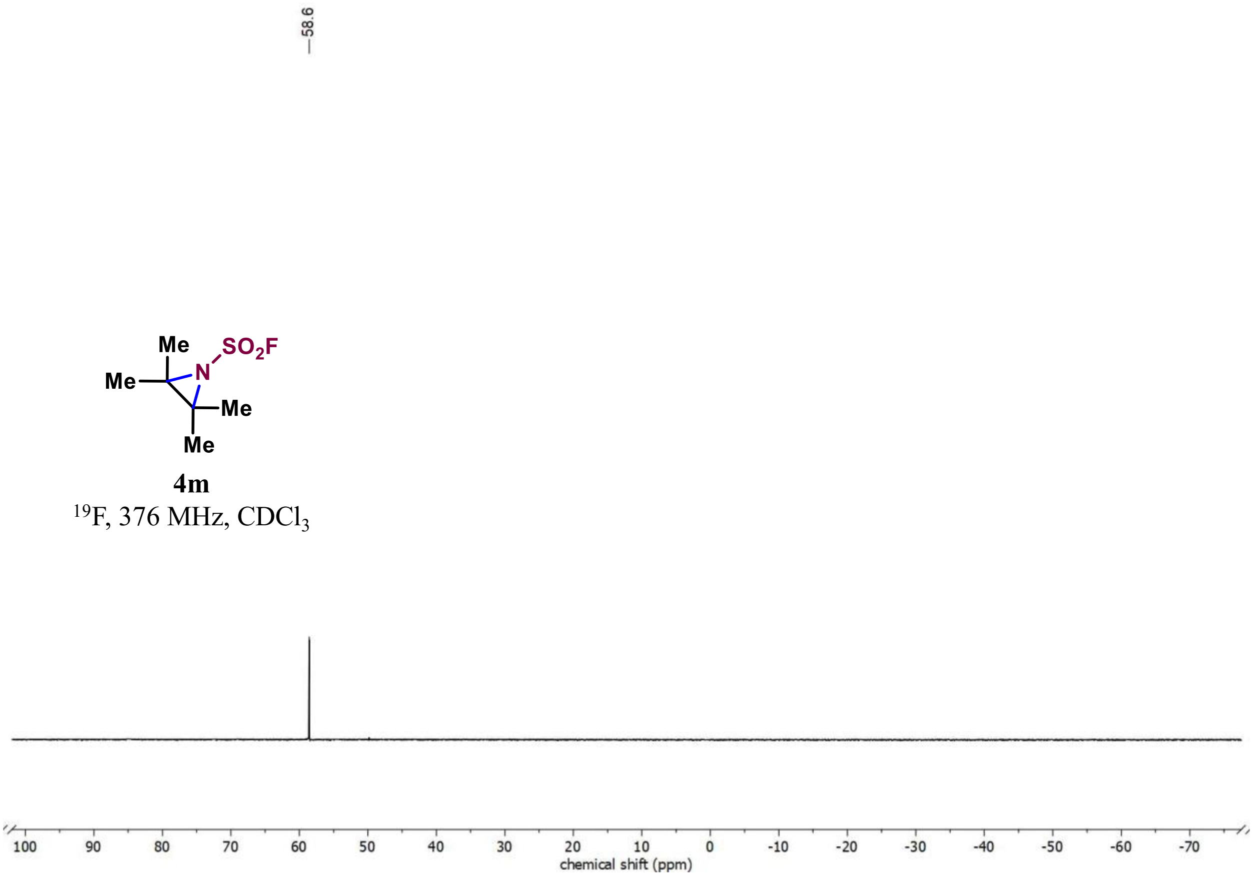

4m

^{19}F, 376 MHz, $CDCl_3$

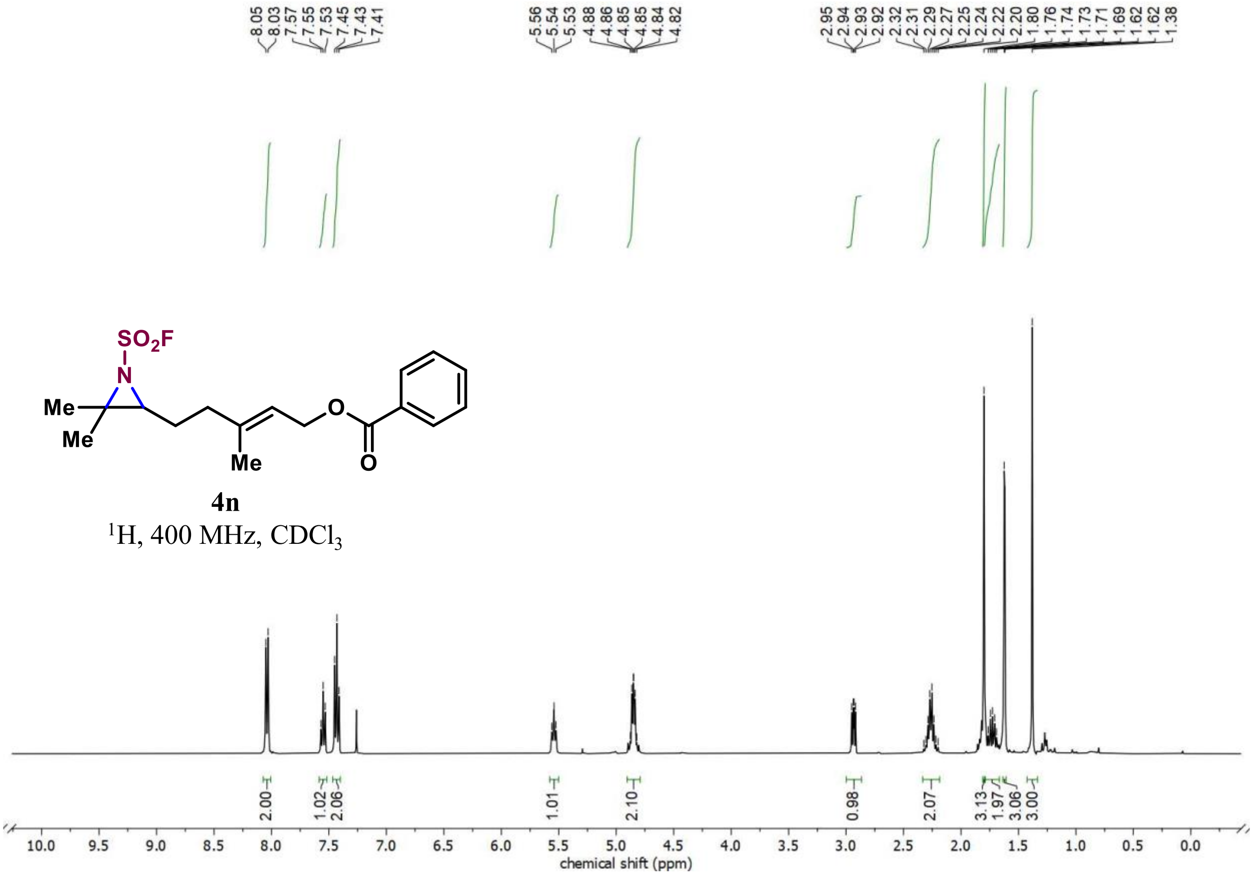

4n

^{1}H, 400 MHz, $CDCl_3$

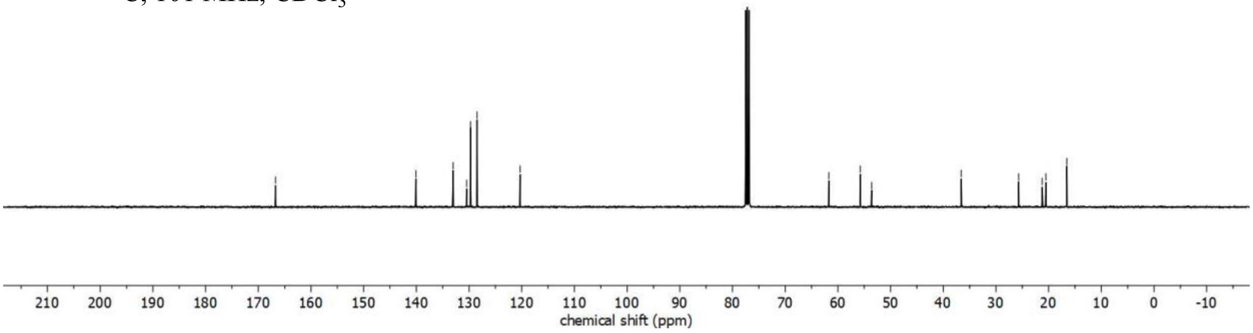

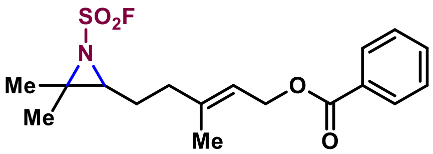

4n

^{13}C, 101 MHz, $CDCl_3$

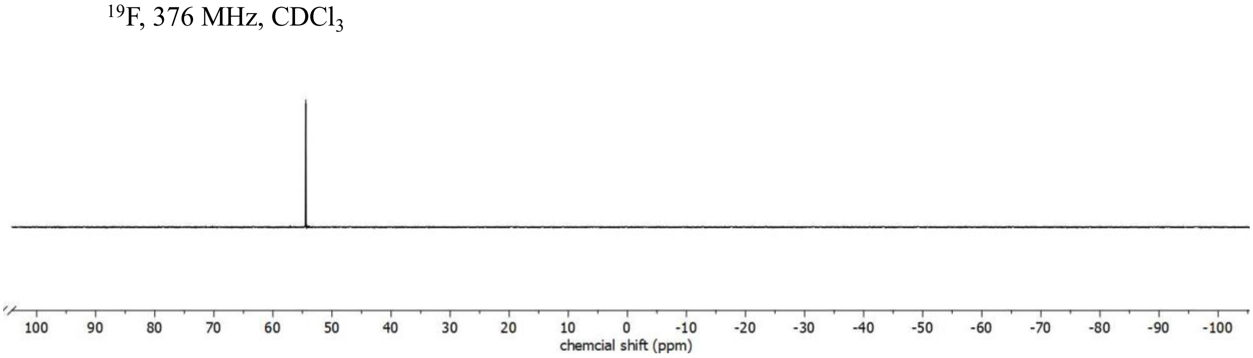

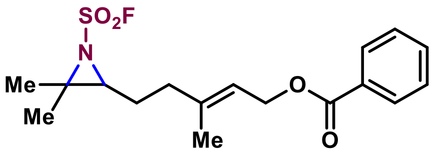

4n

^{19}F, 376 MHz, $CDCl_3$

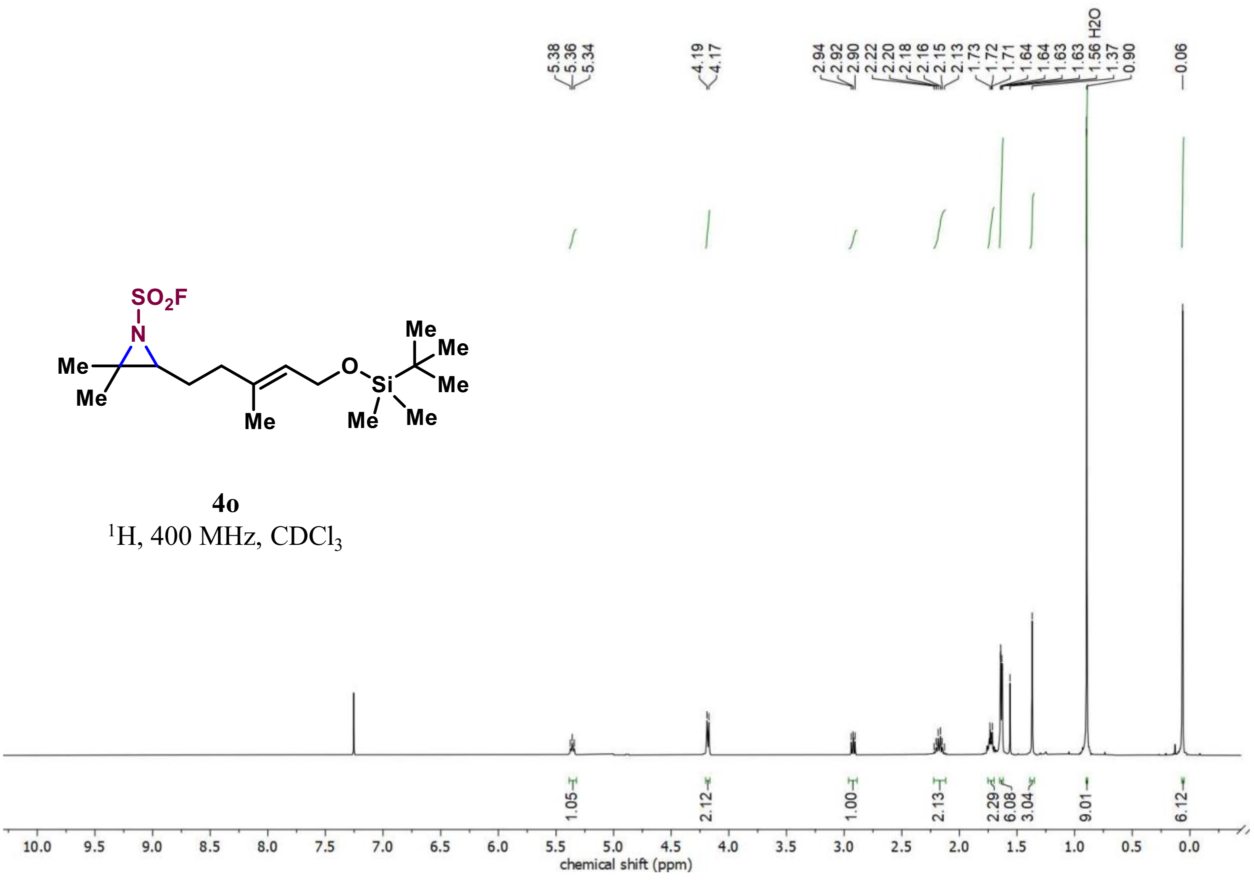

4o

^{1}H, 400 MHz, $CDCl_3$

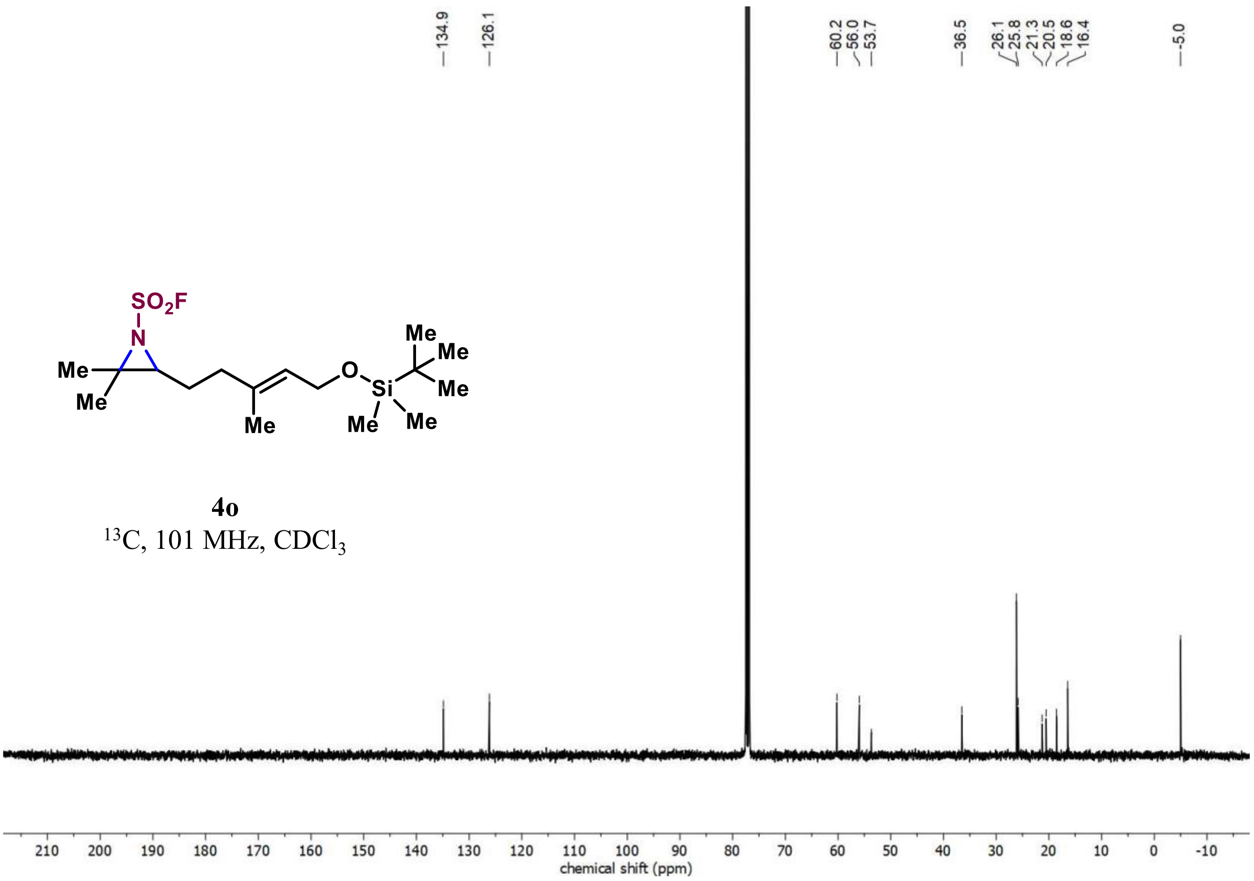

4o

^{13}C, 101 MHz, $CDCl_3$

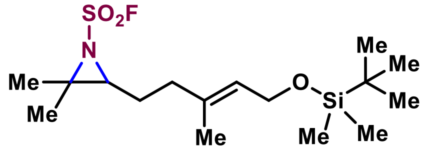

4o

^{19}F, 377 MHz, $CDCl_3$

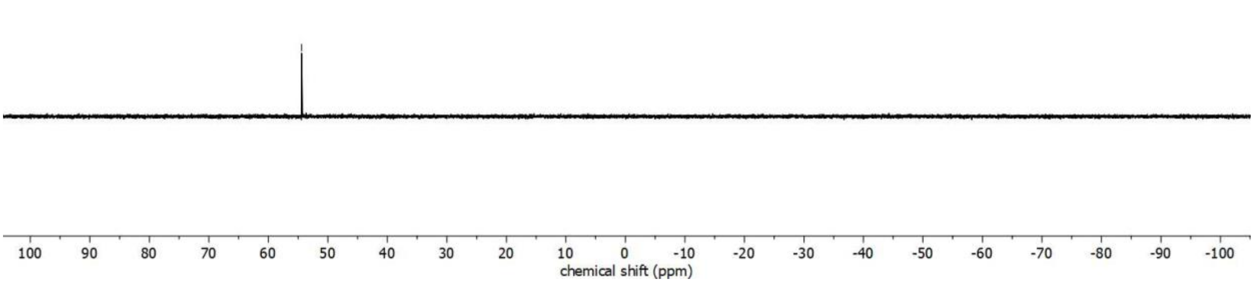

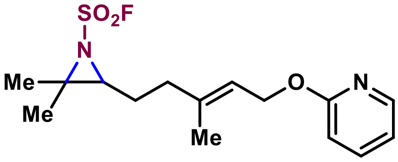

4p

^{1}H, 400 MHz, $CDCl_3$

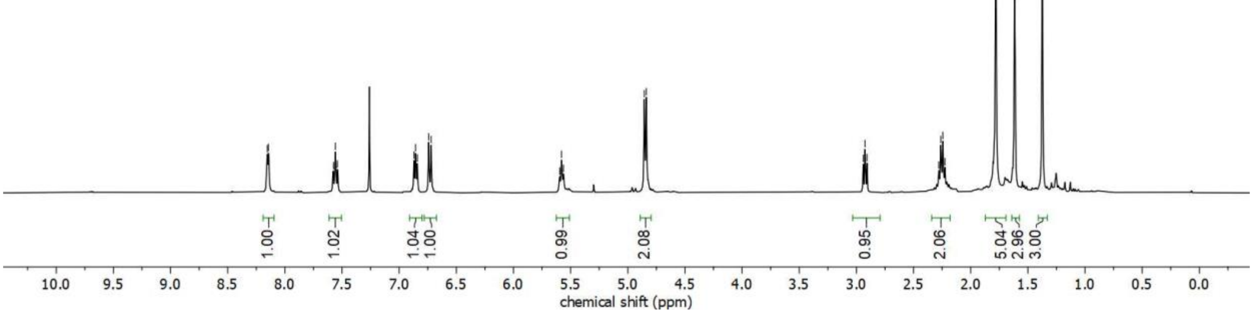

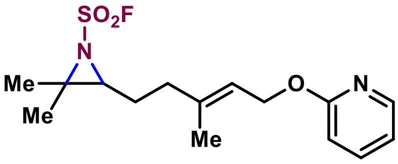

4p

^{13}C, 101 MHz, $CDCl_3$

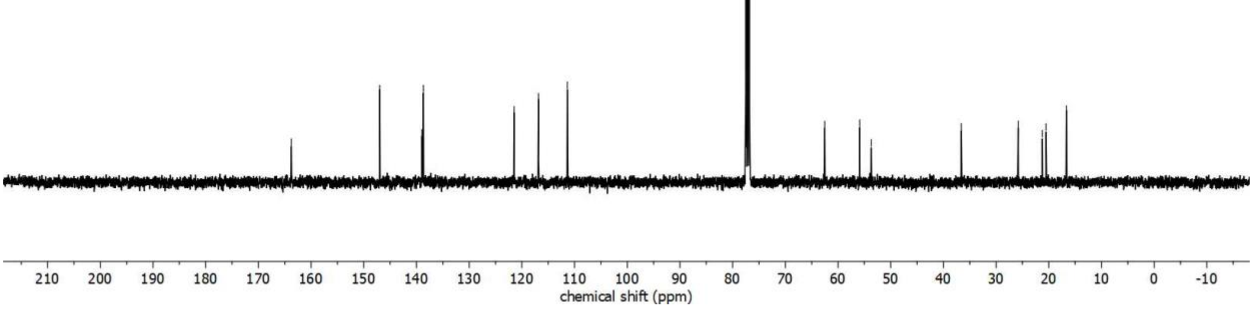

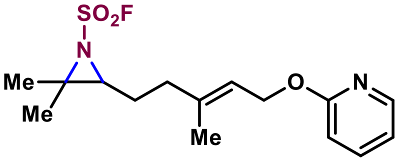

4p

^{19}F, 376 MHz, $CDCl_3$

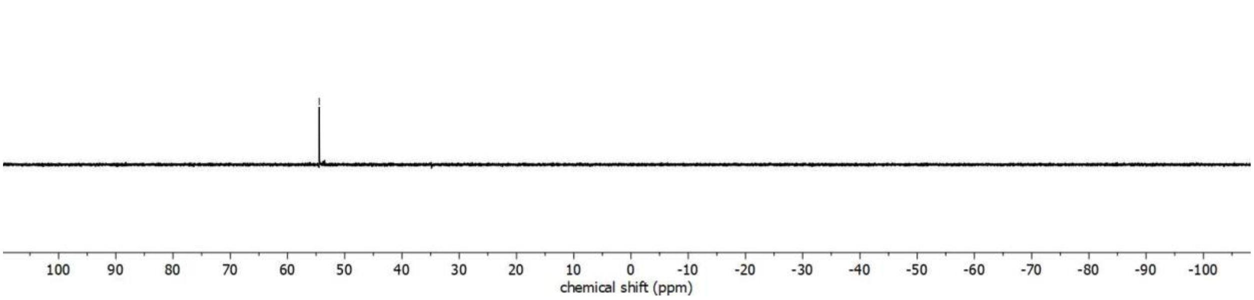

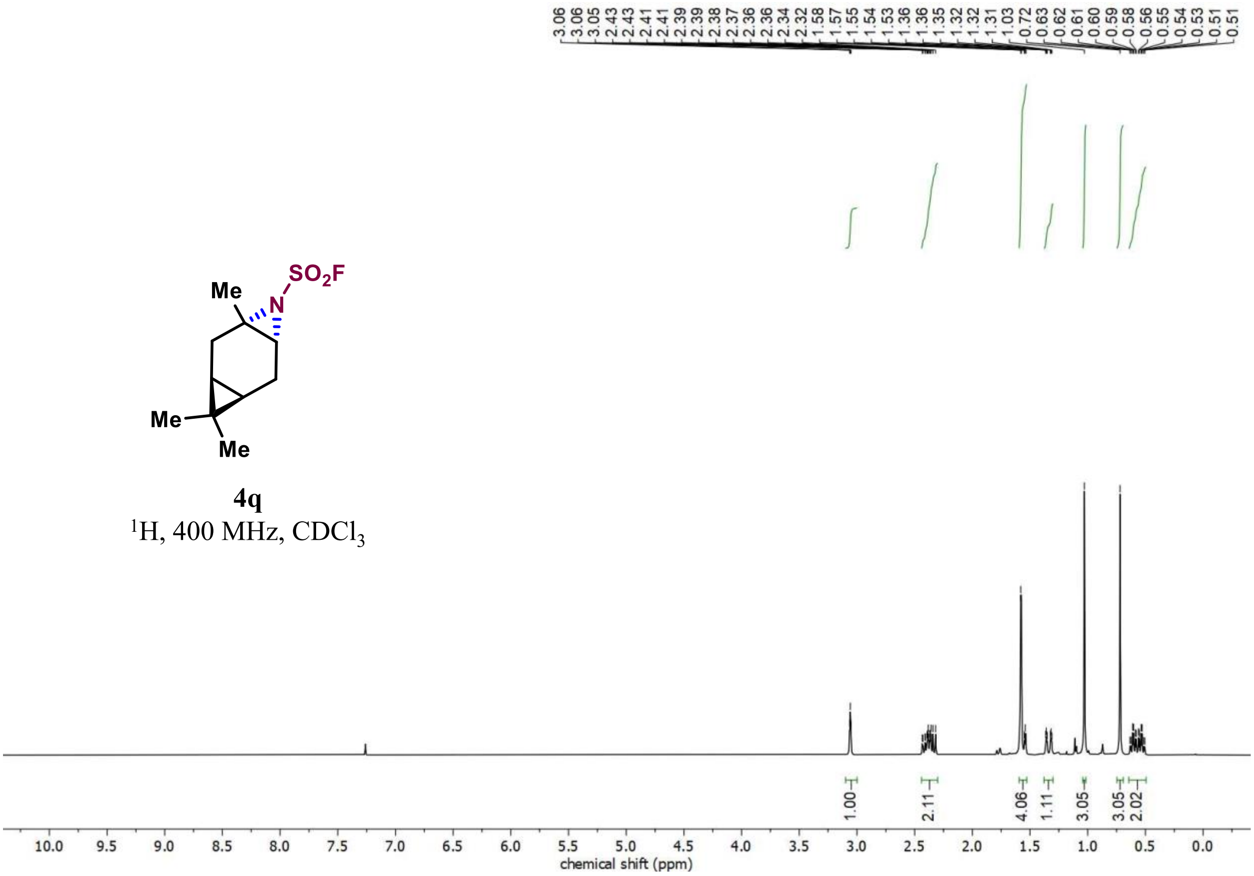

4q

^{1}H, 400 MHz, $CDCl_3$

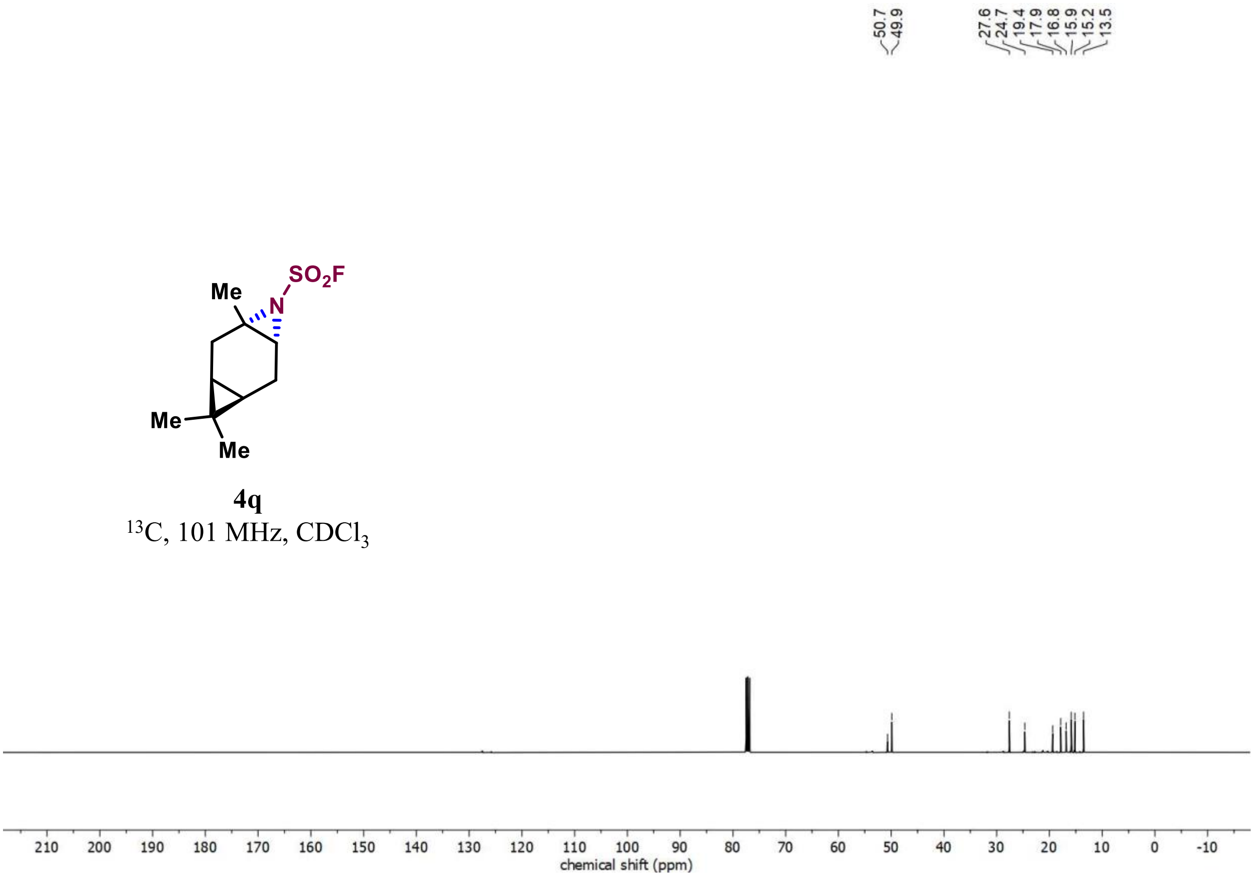

4q

^{13}C, 101 MHz, $CDCl_3$

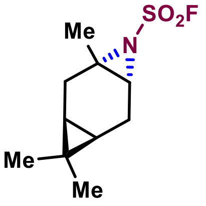

4q
^{19}F, 376 MHz, $CDCl_3$

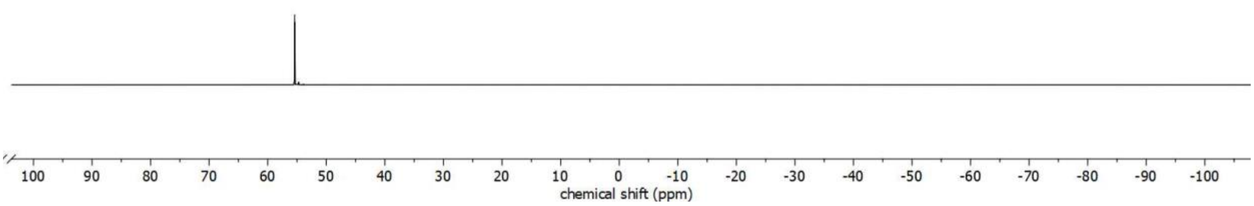

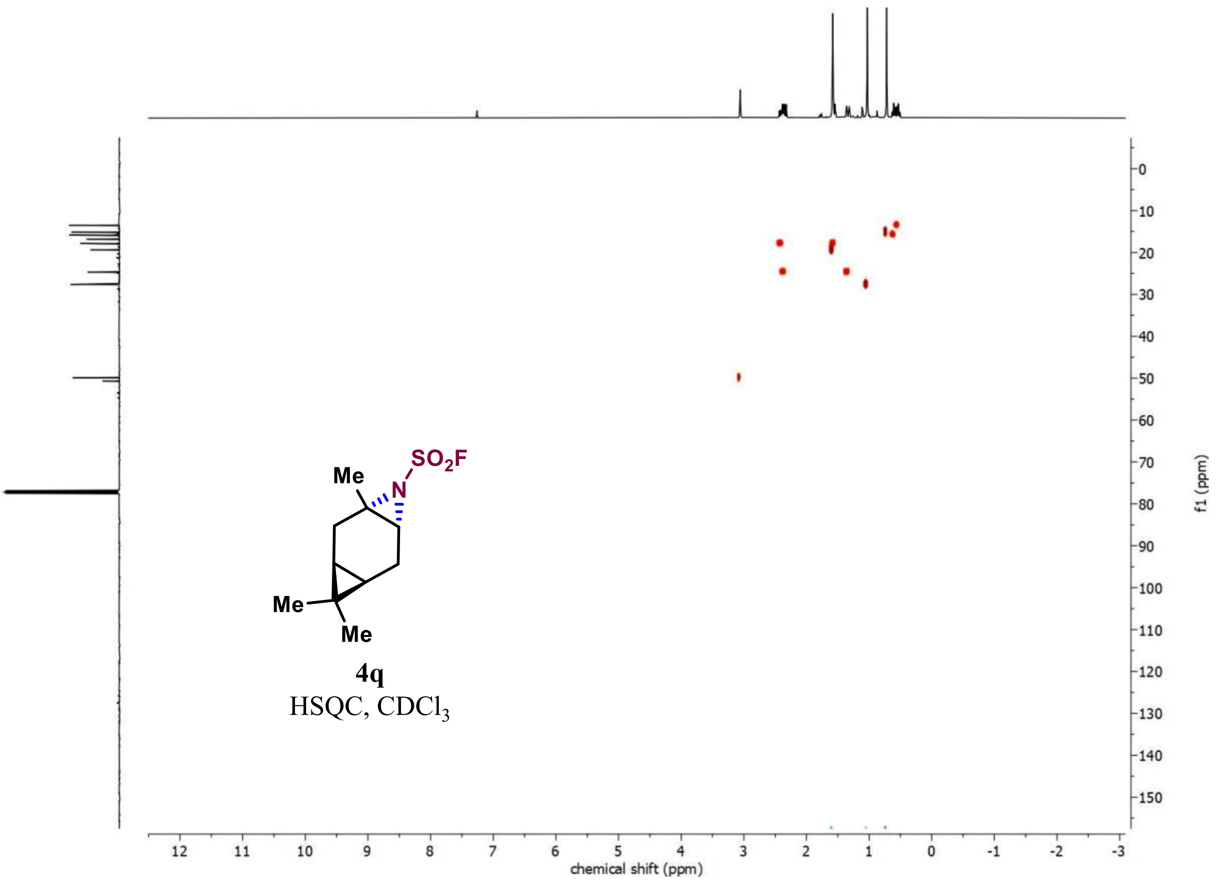

4q
HSQC, $CDCl_3$

4q
NOESY, $CDCl_3$

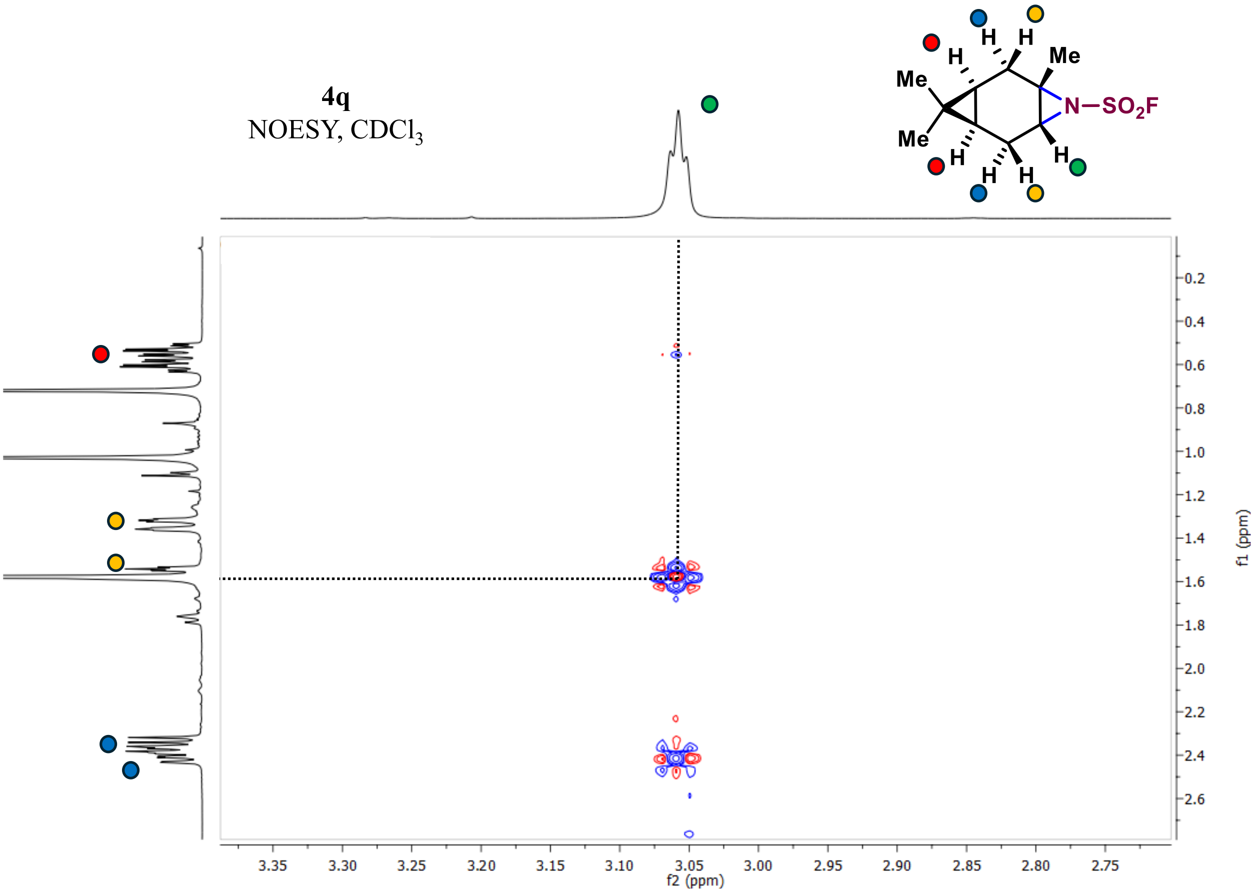

4q
NOESY, $CDCl_3$

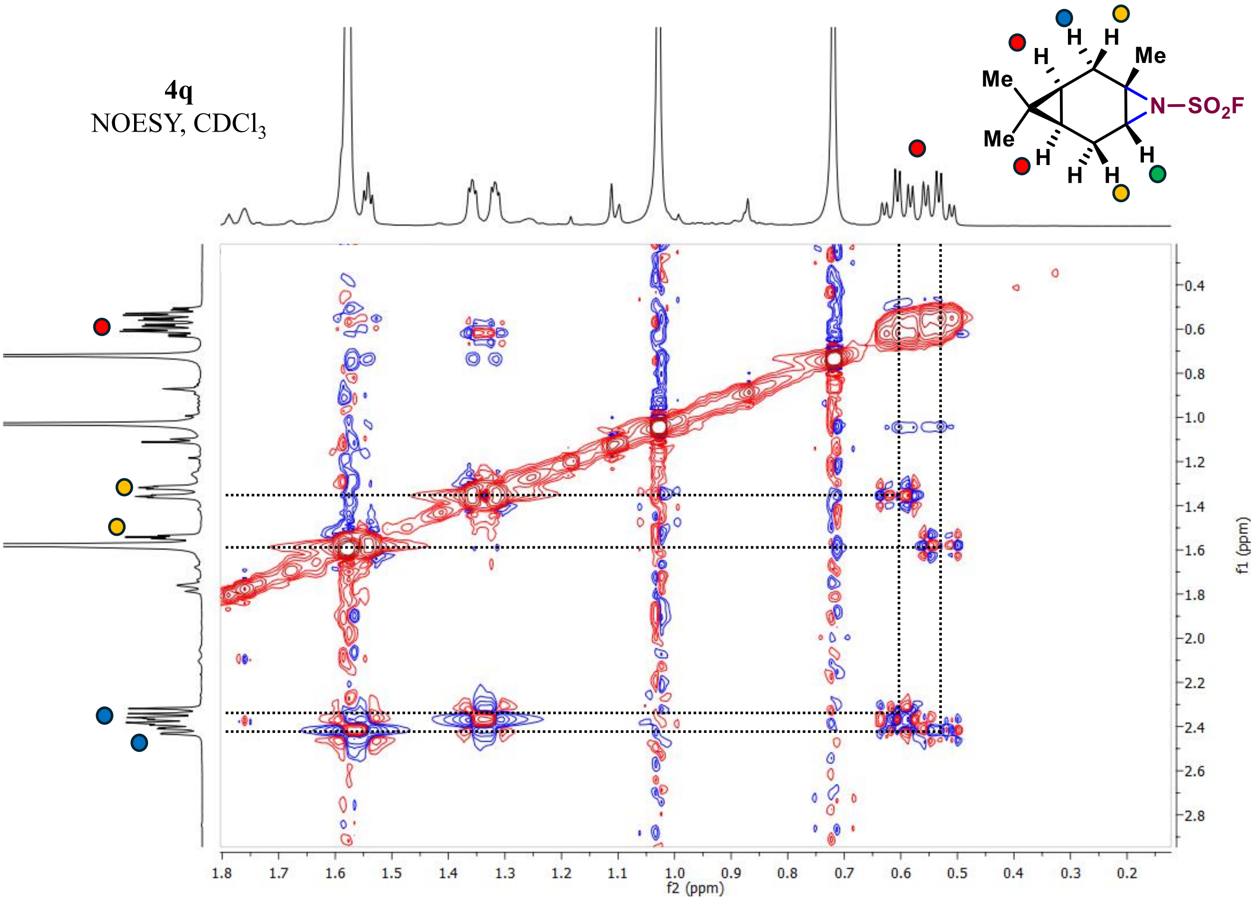

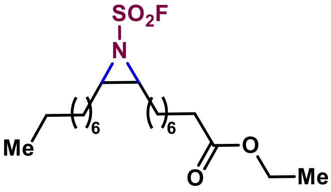

cis-**4r**
^{1}H, 400 MHz, $CDCl_3$

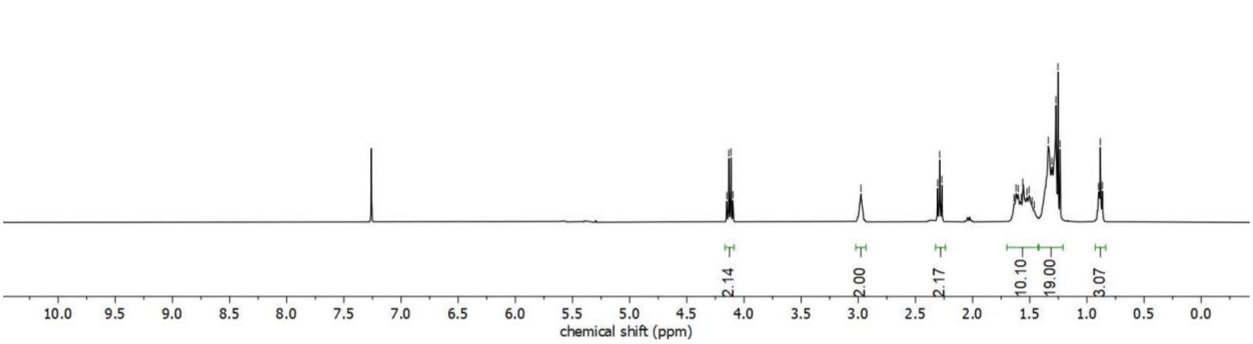

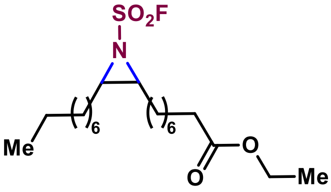

cis-**4r**
^{13}C, 101 MHz, $CDCl_3$

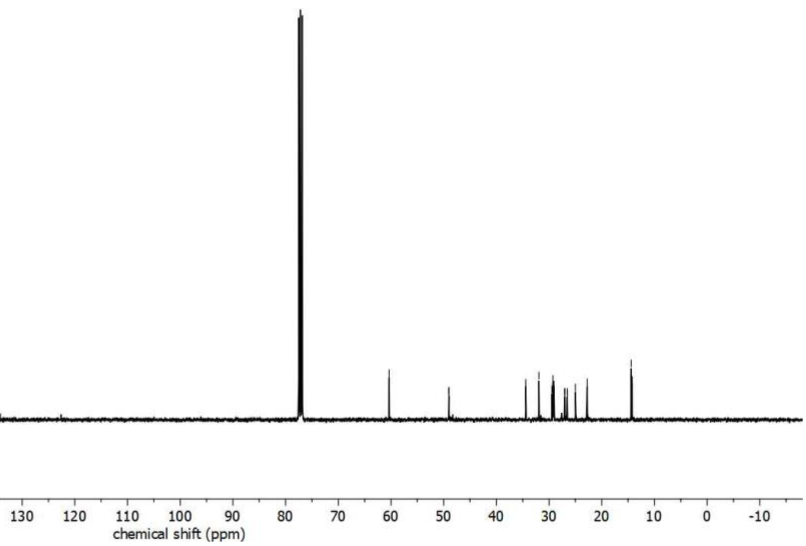

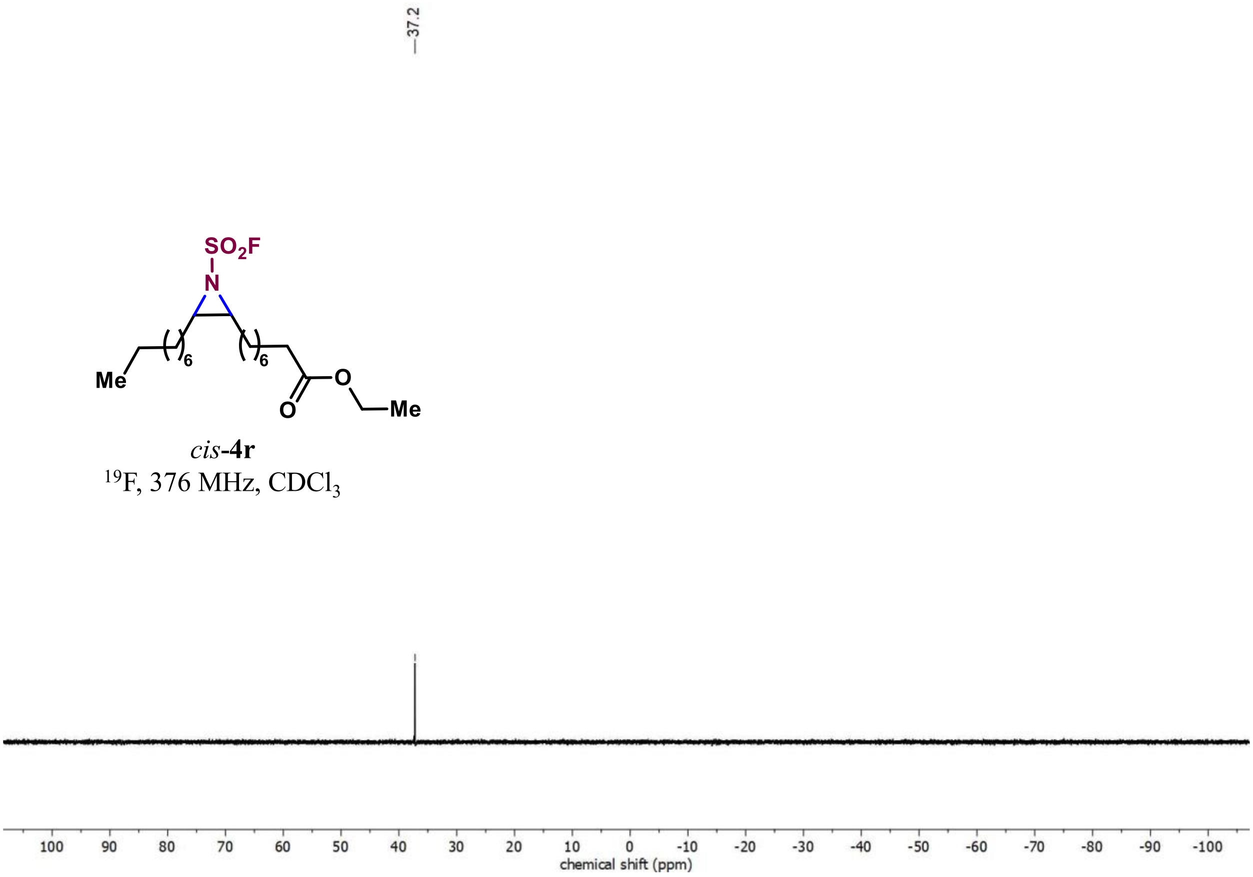

cis-**4r**
^{19}F, 376 MHz, $CDCl_3$

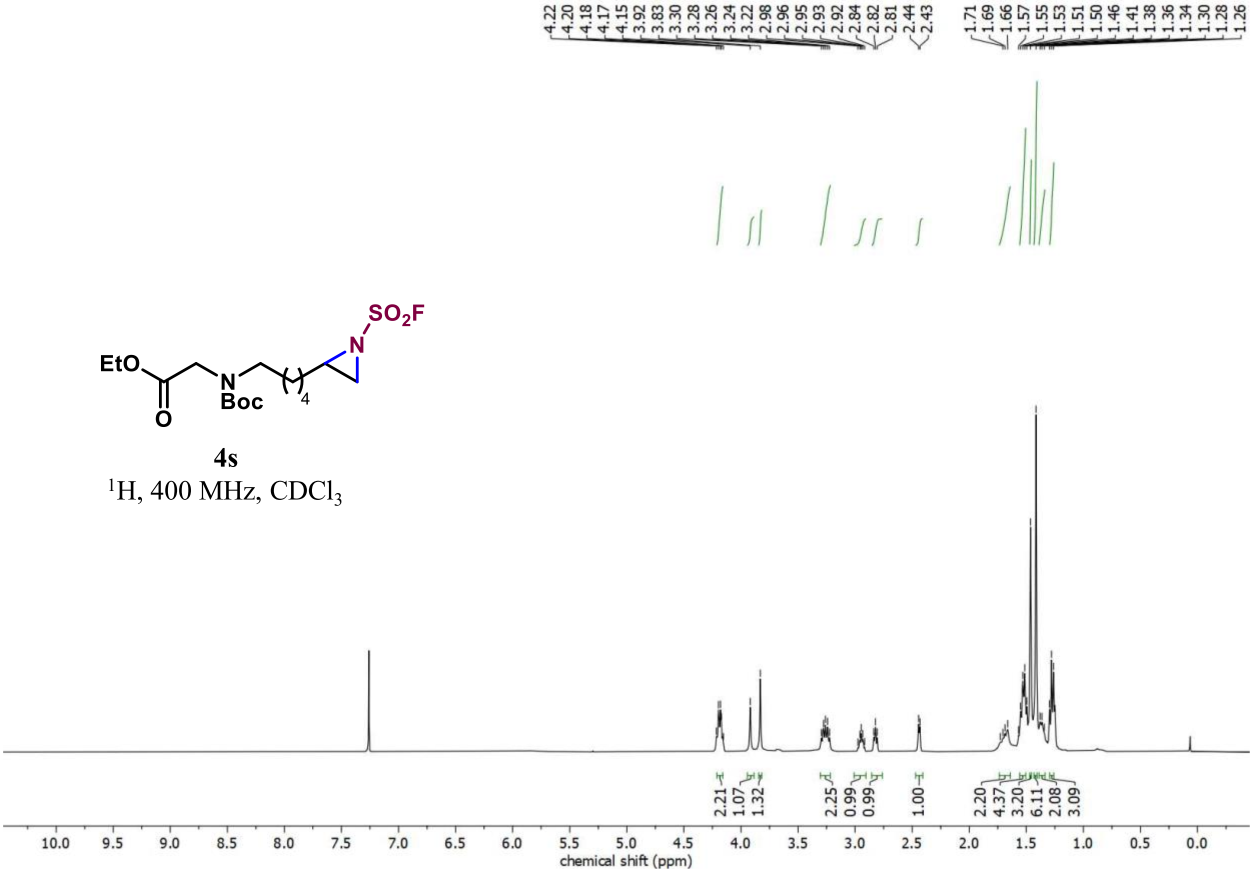

4s
^{1}H, 400 MHz, $CDCl_3$

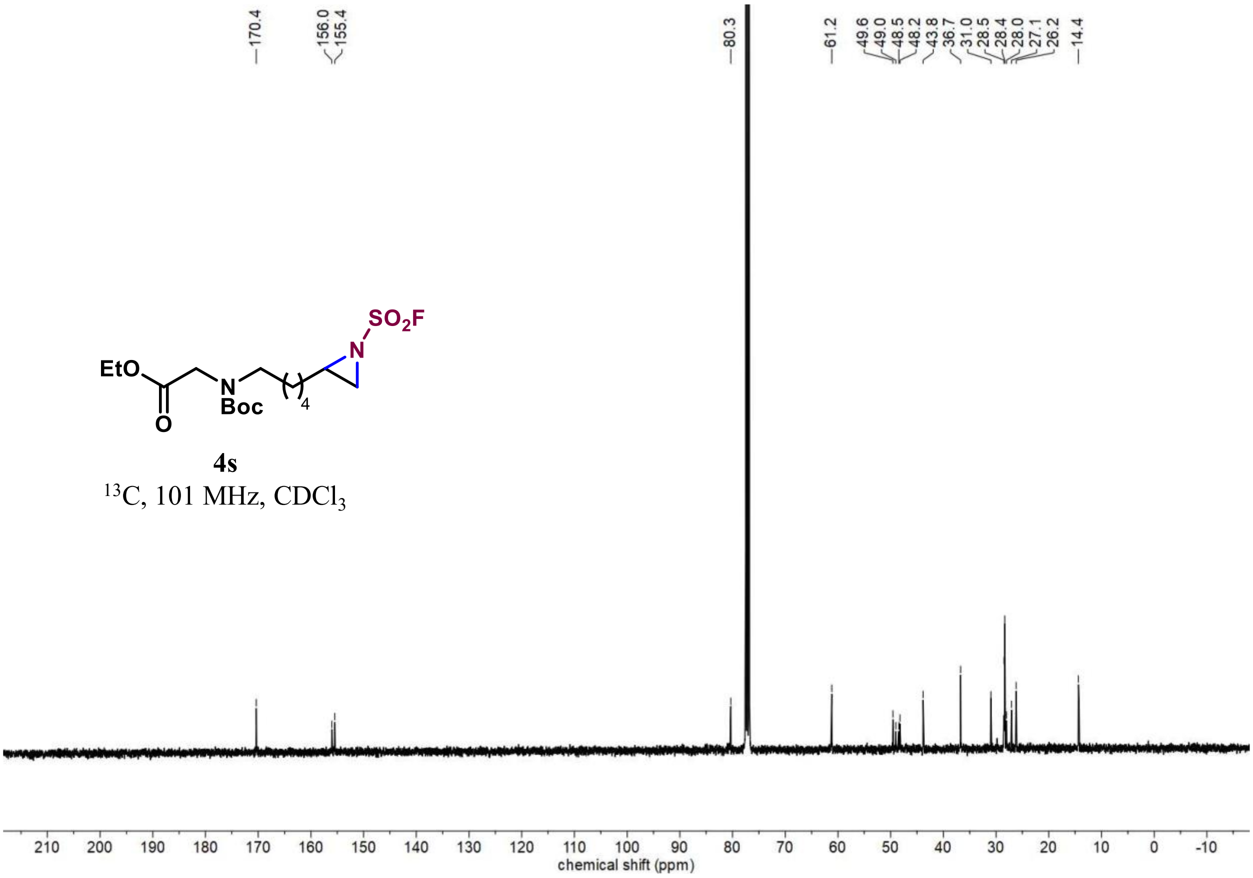

4s

^{13}C, 101 MHz, $CDCl_3$

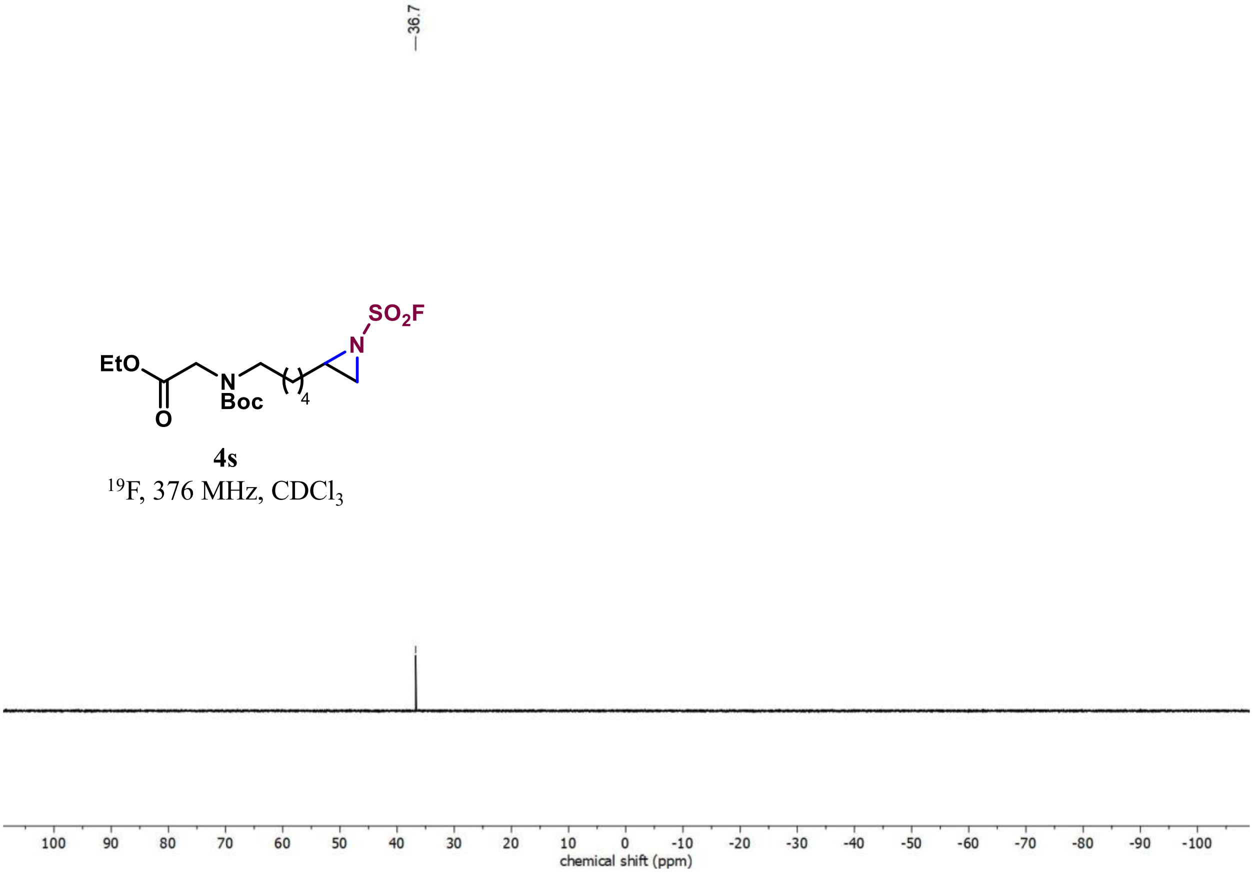

4s

^{19}F, 376 MHz, $CDCl_3$

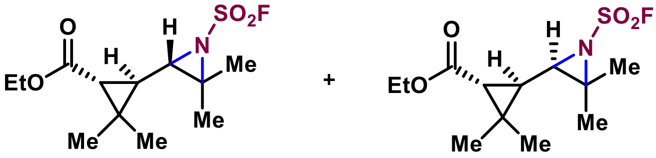

trans : cis = 3 : 1

4t

^{1}H, 400 MHz, $CDCl_3$

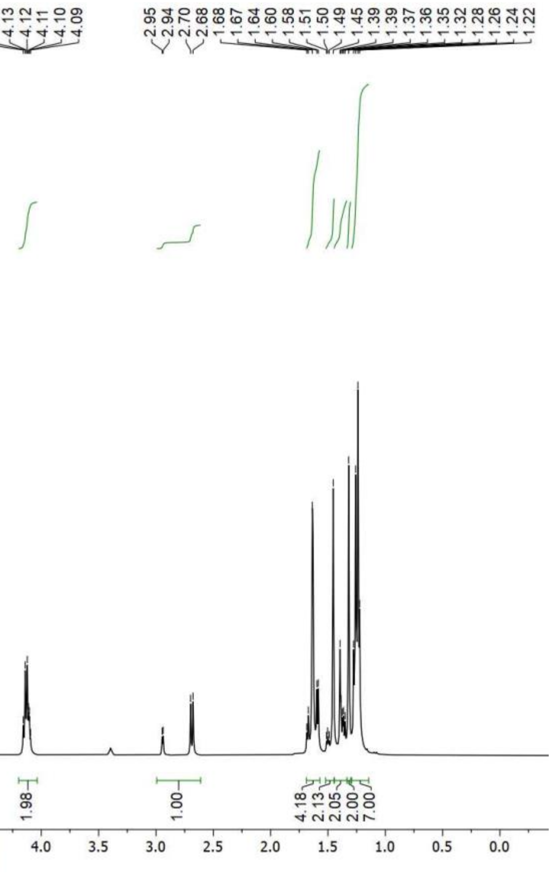

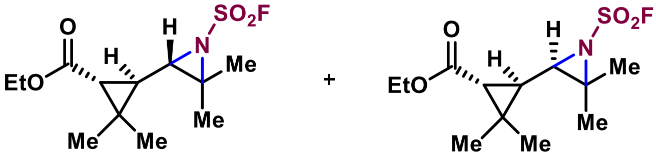

trans : cis = 3 : 1

4t

^{13}C, 101 MHz, $CDCl_3$

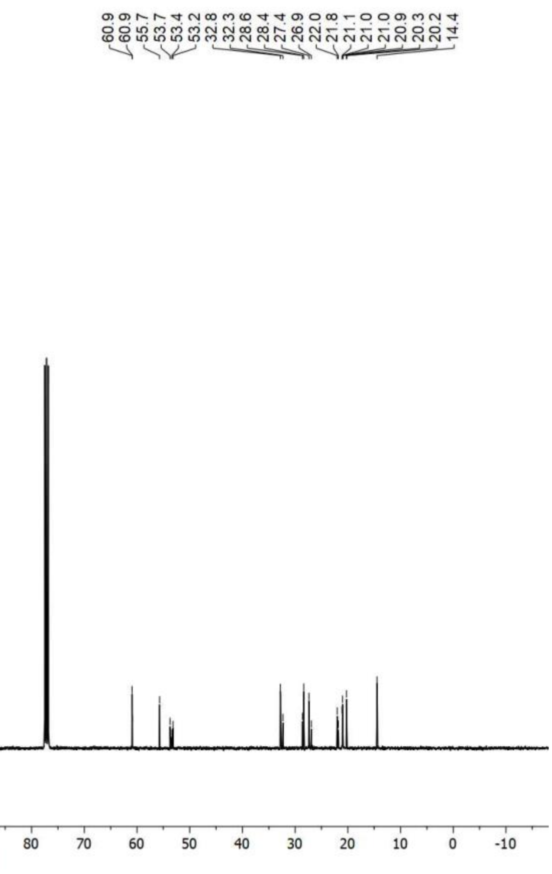

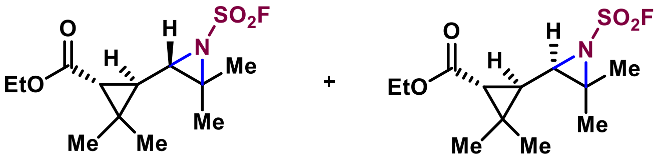

trans : cis = 3 : 1

4t

^{19}F, 376 MHz, $CDCl_3$

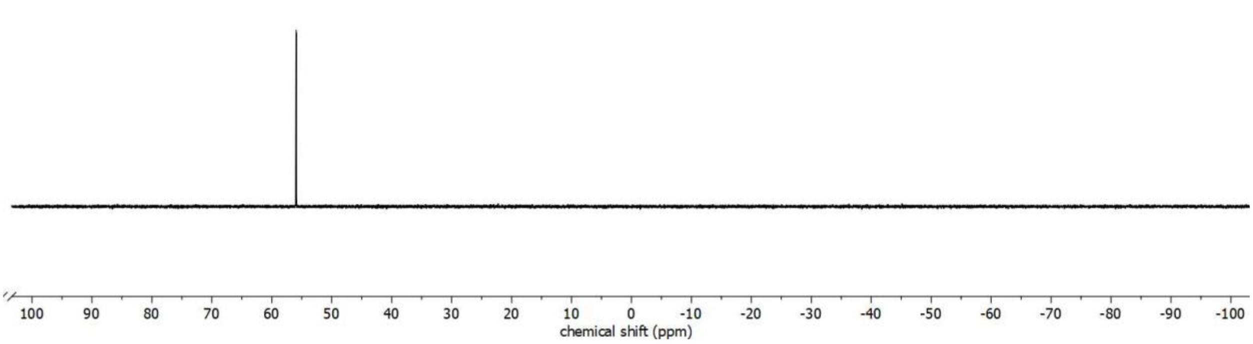

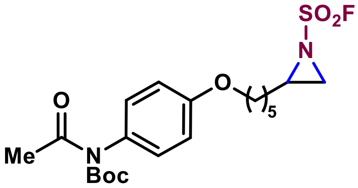

4u

^{1}H, 400 MHz, $CDCl_3$

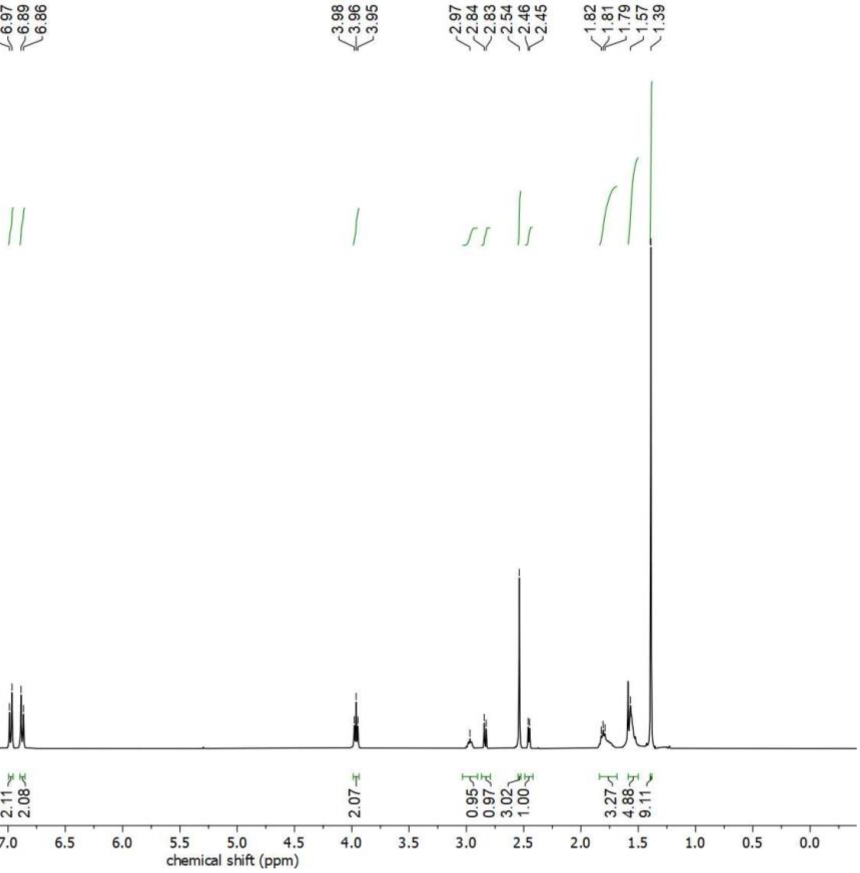

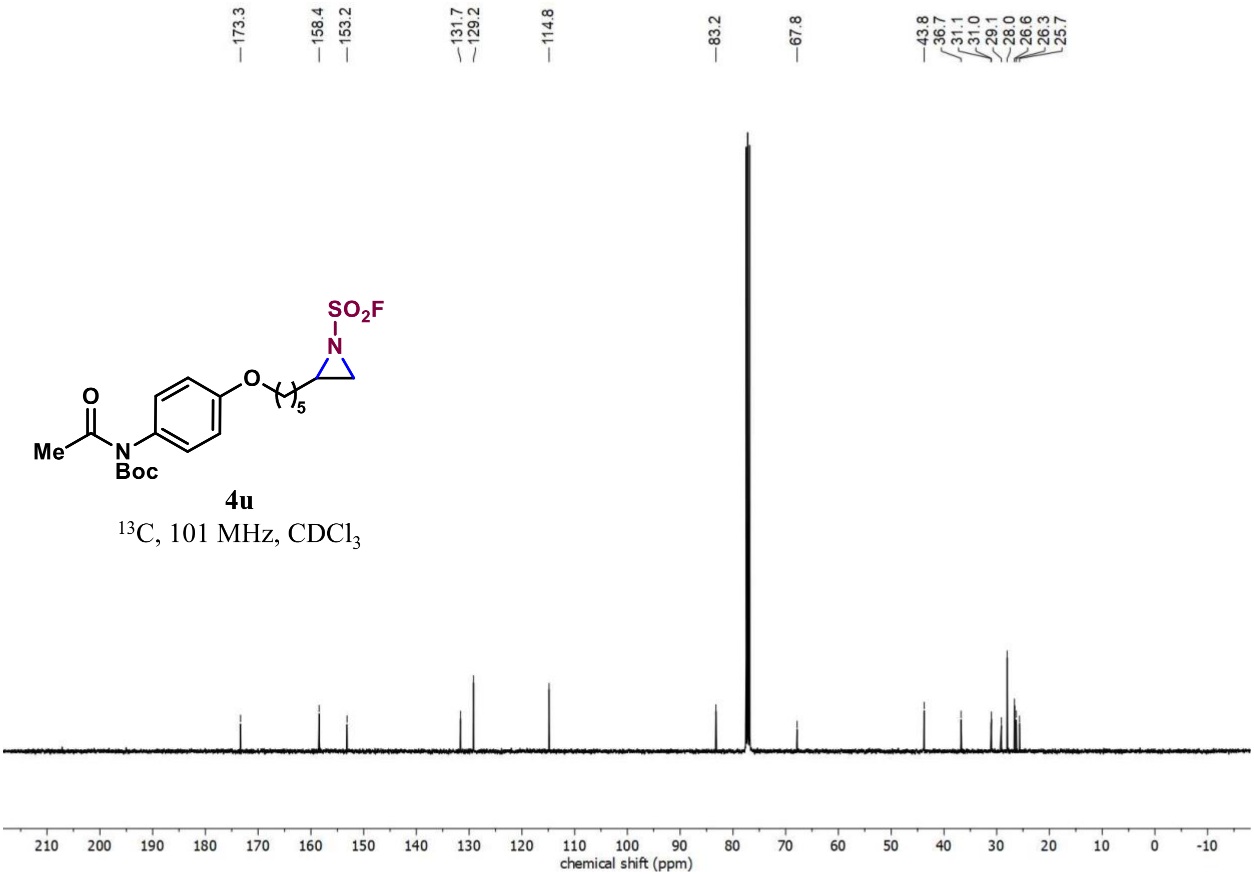
173.3
158.4
153.2
131.7
129.2
114.8
83.2
67.8
43.8
36.7
31.1
31.0
29.1
28.0
26.6
26.3
25.7
SO2F
N
O
O
5
Me
N
Boc
4u
13C, 101 MHz, CDCl3
210 200 190 180 170 160 150 140 130 120 110 100 90 80 70 60 50 40 30 20 10 0 -10
chemical shift (ppm)

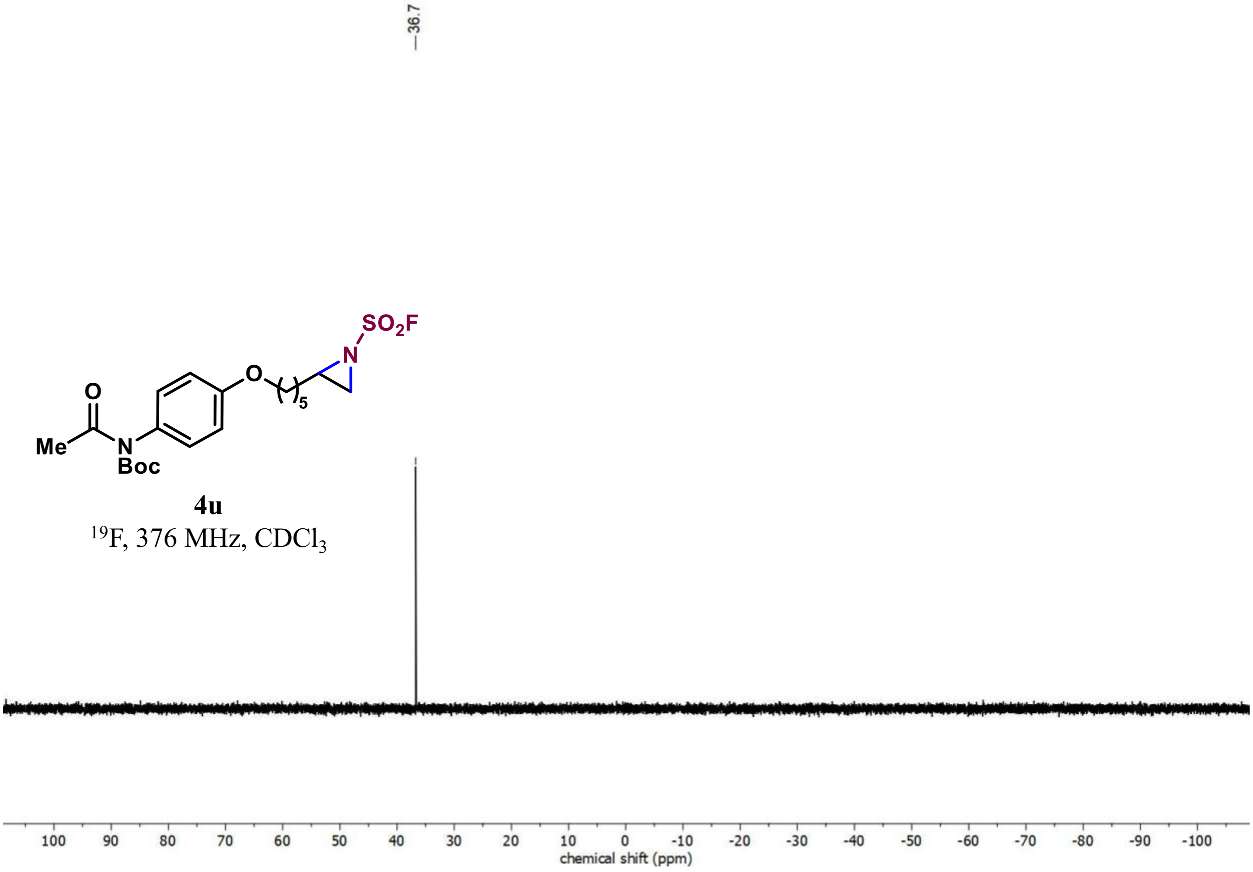
36.7
SO2F
N
O
O
5
Me
N
Boc
4u
19F, 376 MHz, CDCl3
100 90 80 70 60 50 40 30 20 10 0 -10 -20 -30 -40 -50 -60 -70 -80 -90 -100
chemical shift (ppm)

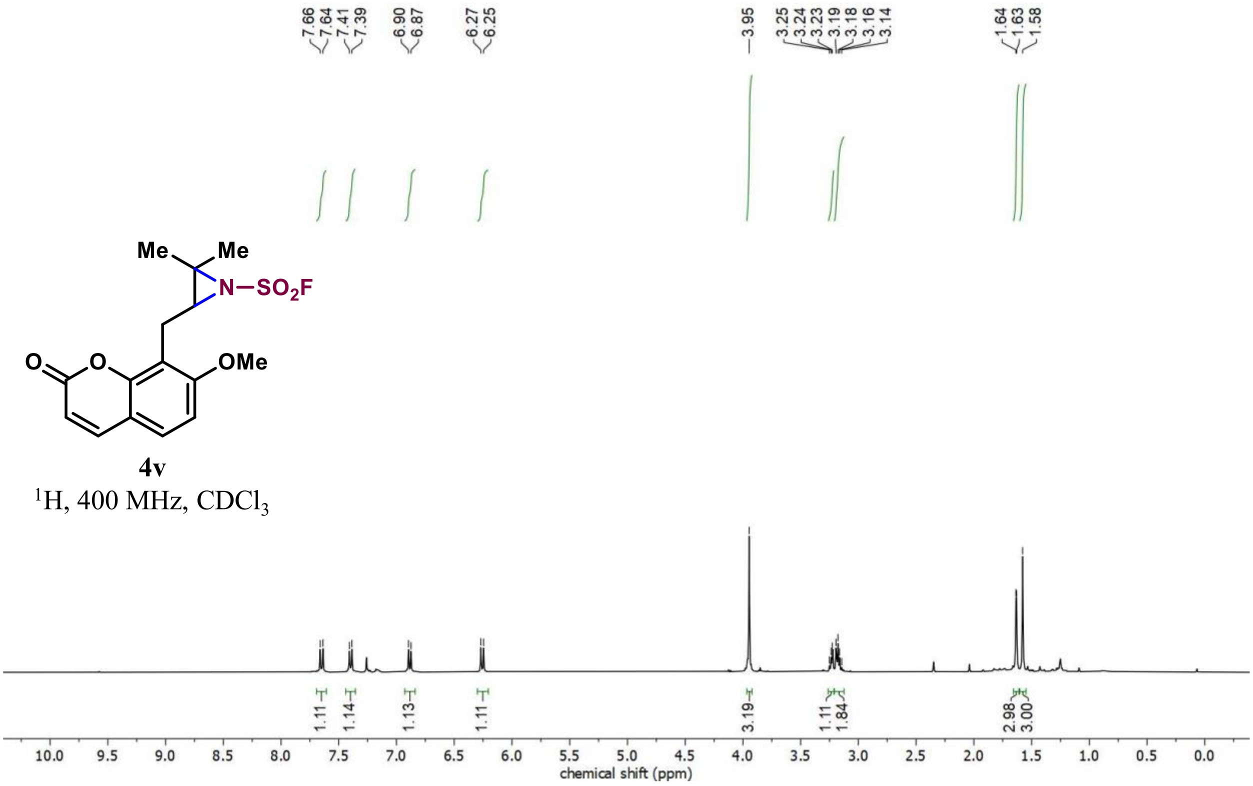

4v

^{1}H, 400 MHz, $CDCl_3$

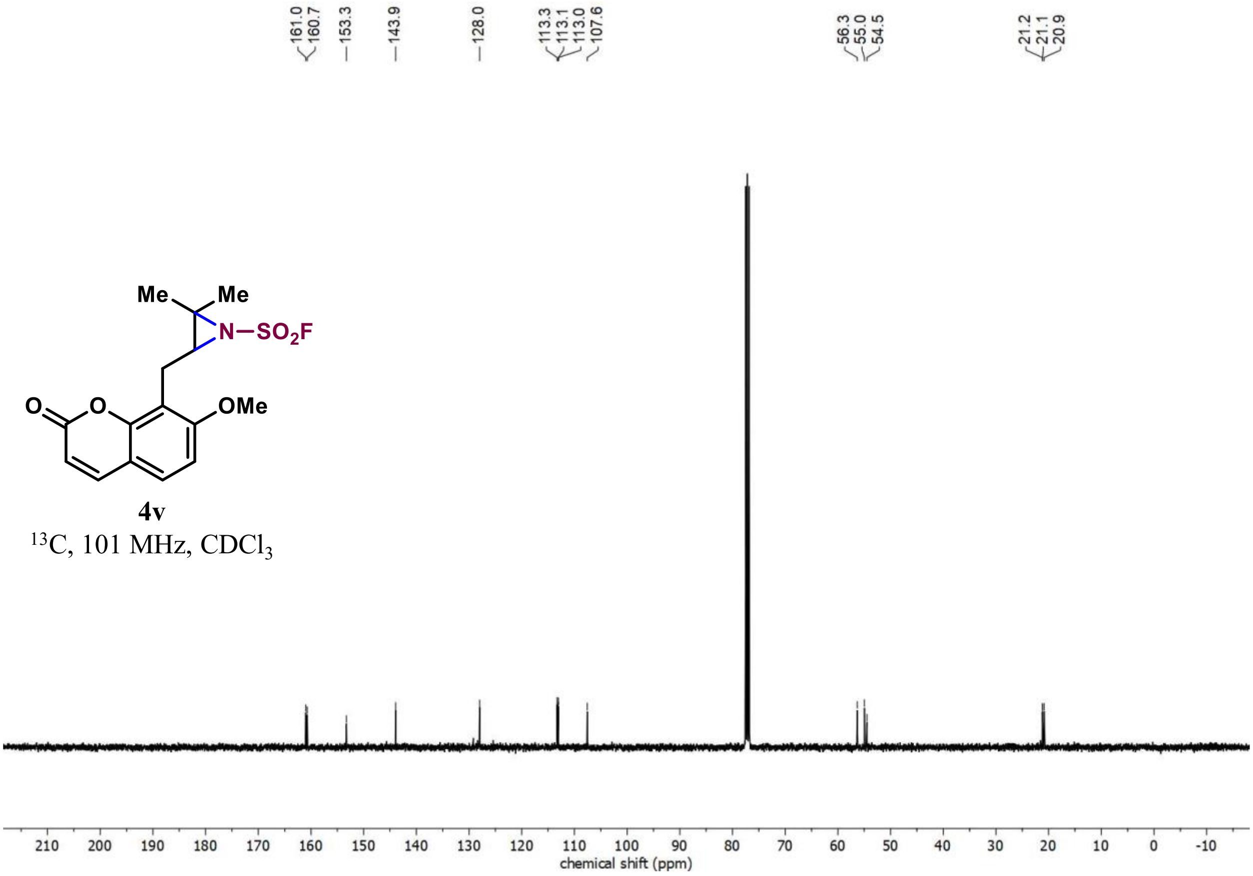

4v

^{13}C, 101 MHz, $CDCl_3$

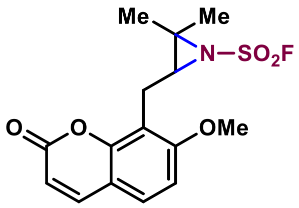

4v

^{19}F, 376 MHz, $CDCl_3$

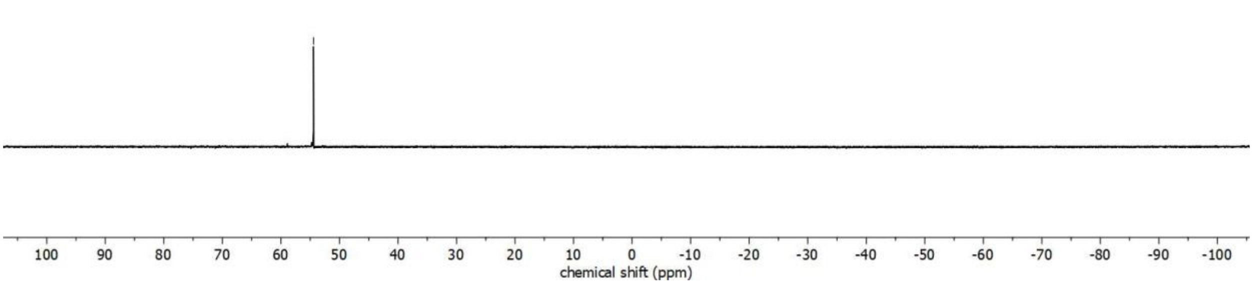

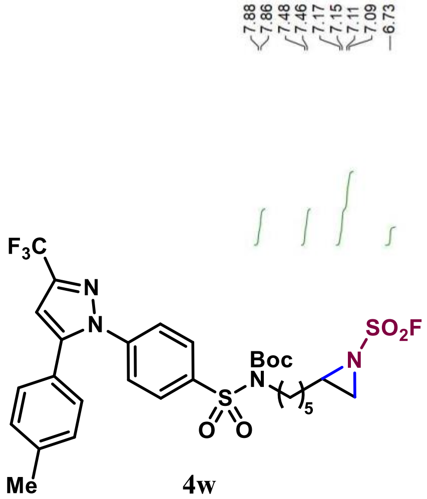

4w

^{1}H, 400 MHz, $CDCl_3$

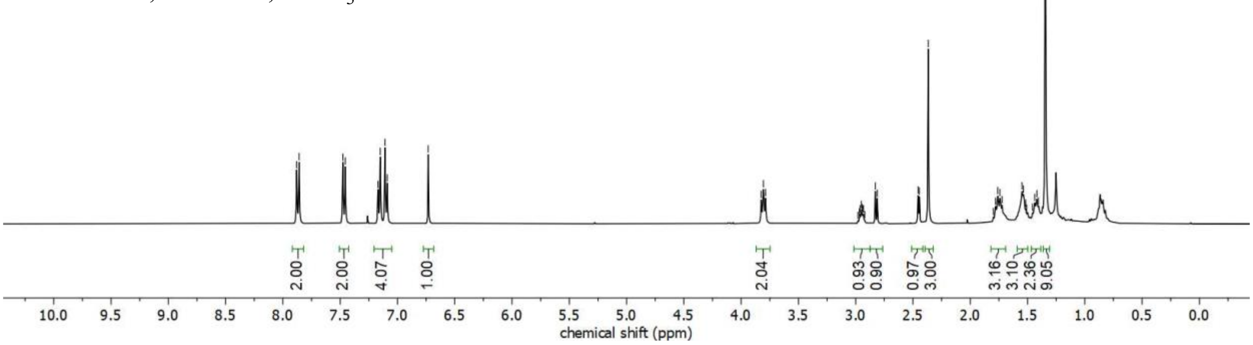

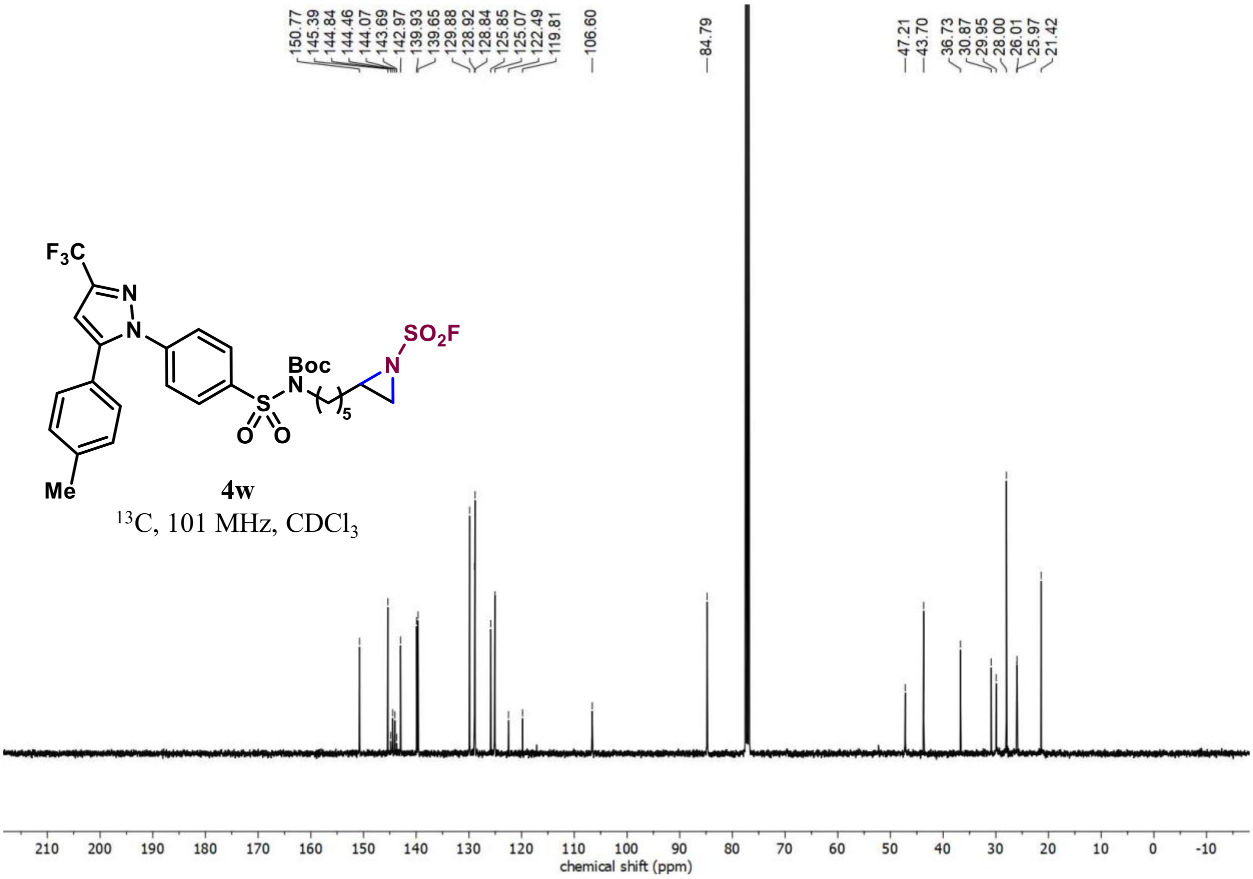

4w

^{13}C, 101 MHz, $CDCl_3$

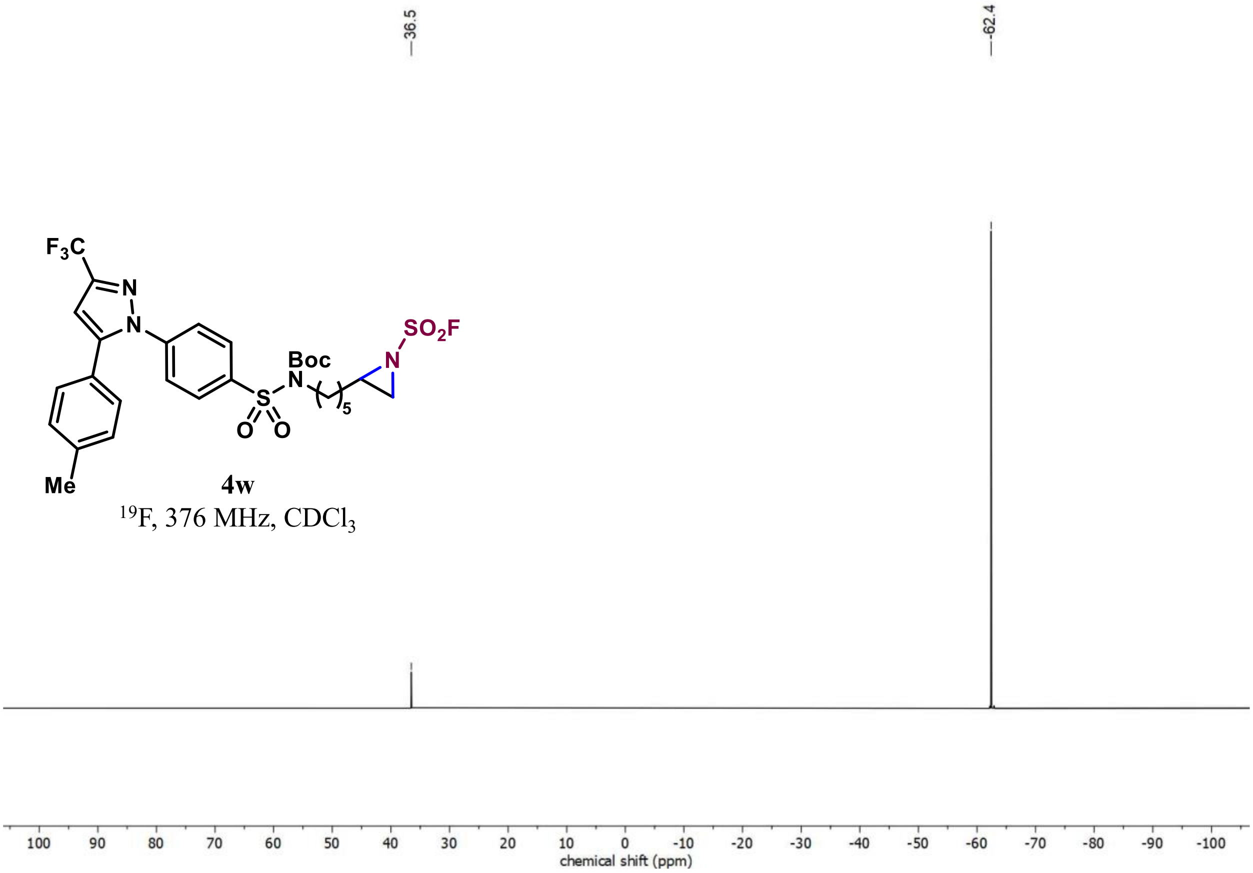

4w

^{19}F, 376 MHz, $CDCl_3$

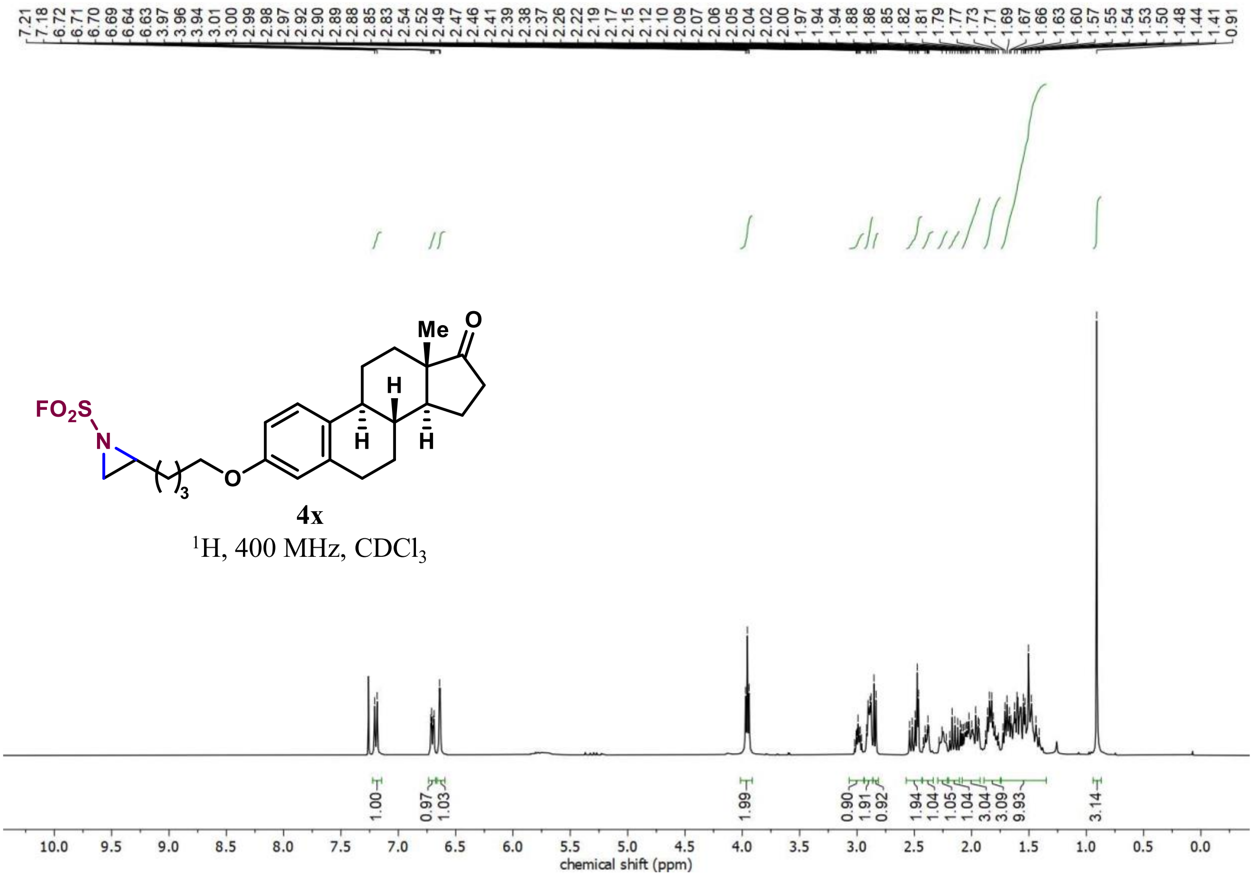

4x
^{1}H, 400 MHz, $CDCl_3$

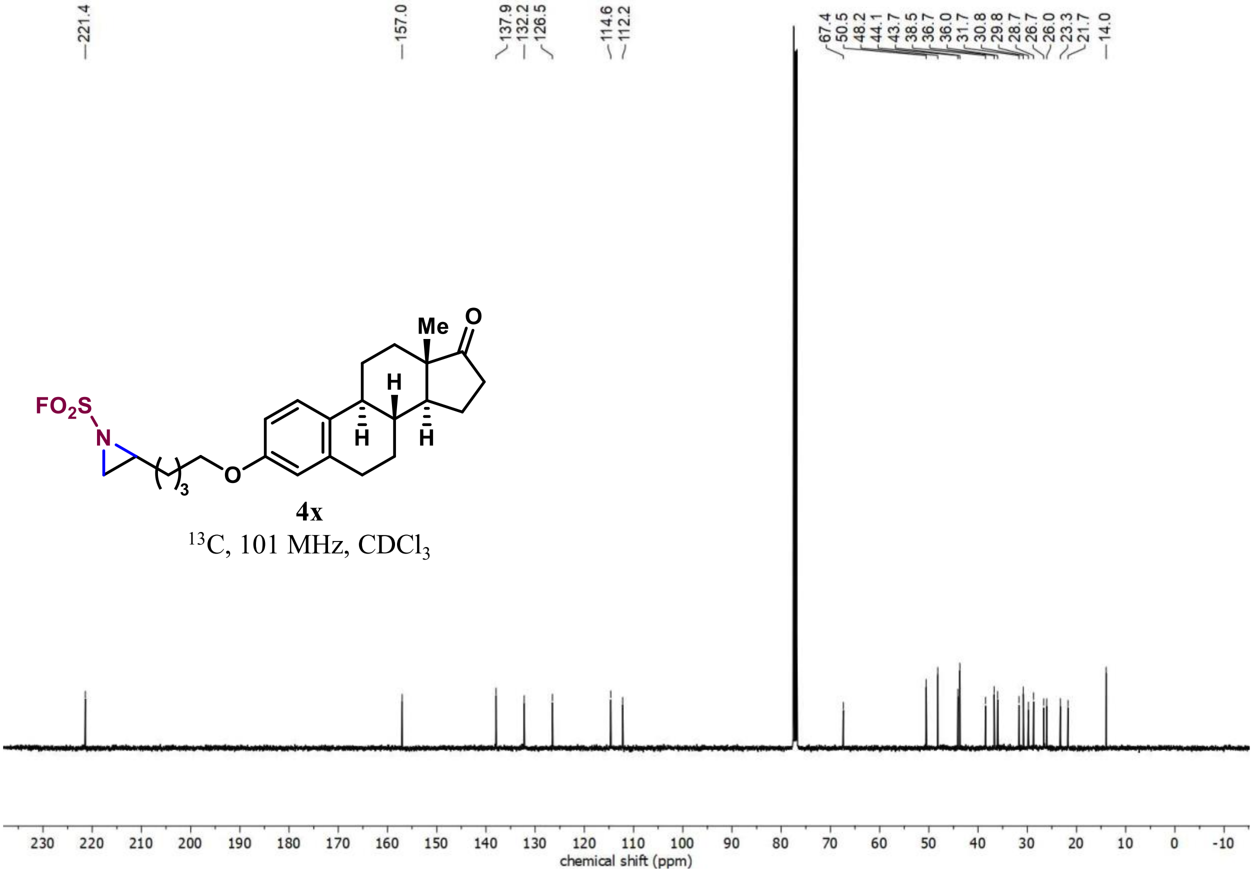

4x
^{13}C, 101 MHz, $CDCl_3$

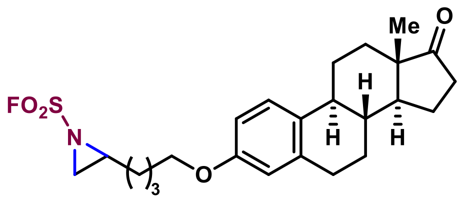

4x

^{19}F, 376 MHz, $CDCl_3$

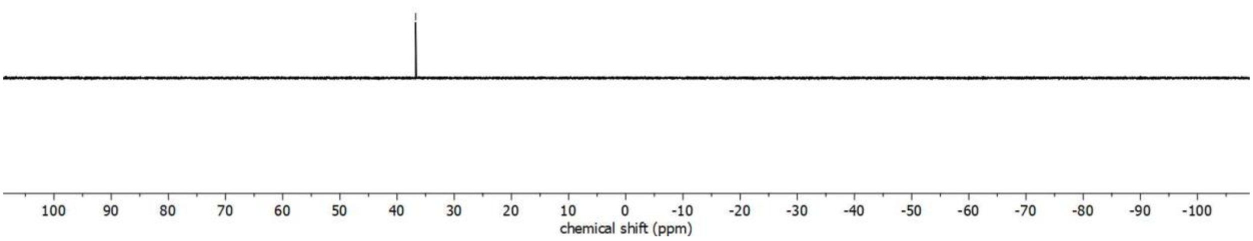

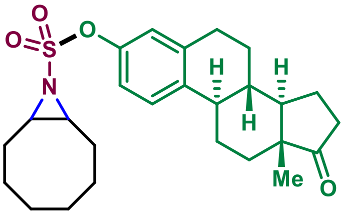

5a

^{1}H, 400 MHz, $CDCl_3$

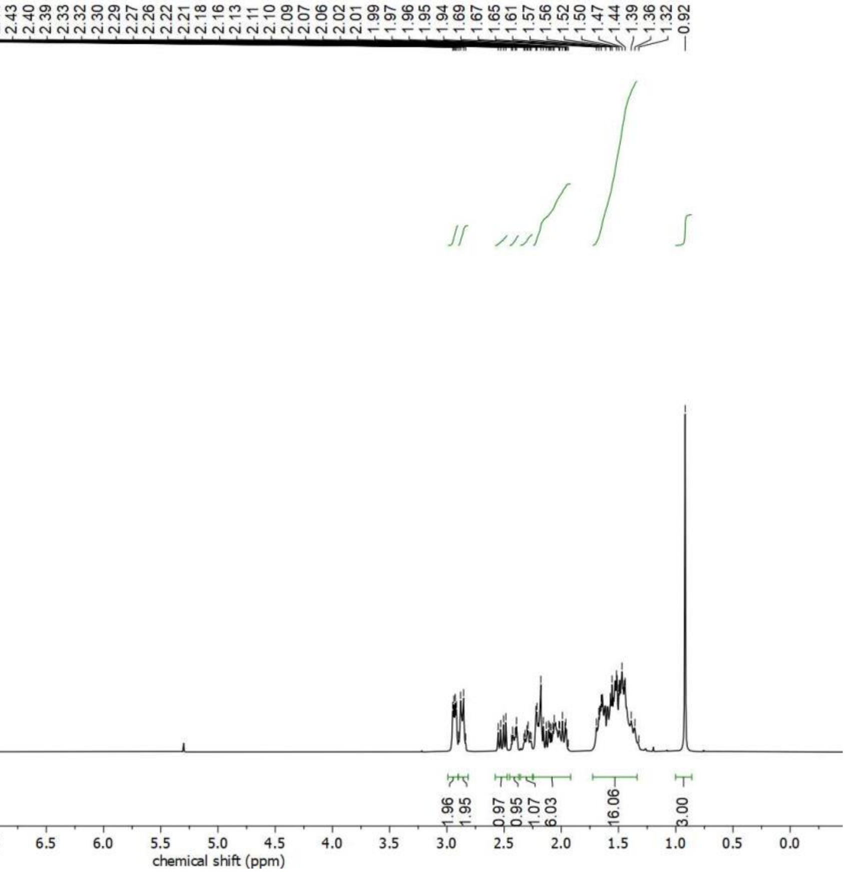

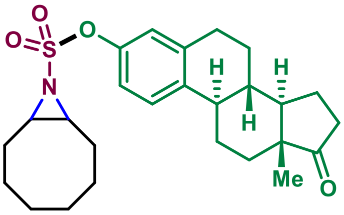

5a

^{13}C, 101 MHz, $CDCl_3$

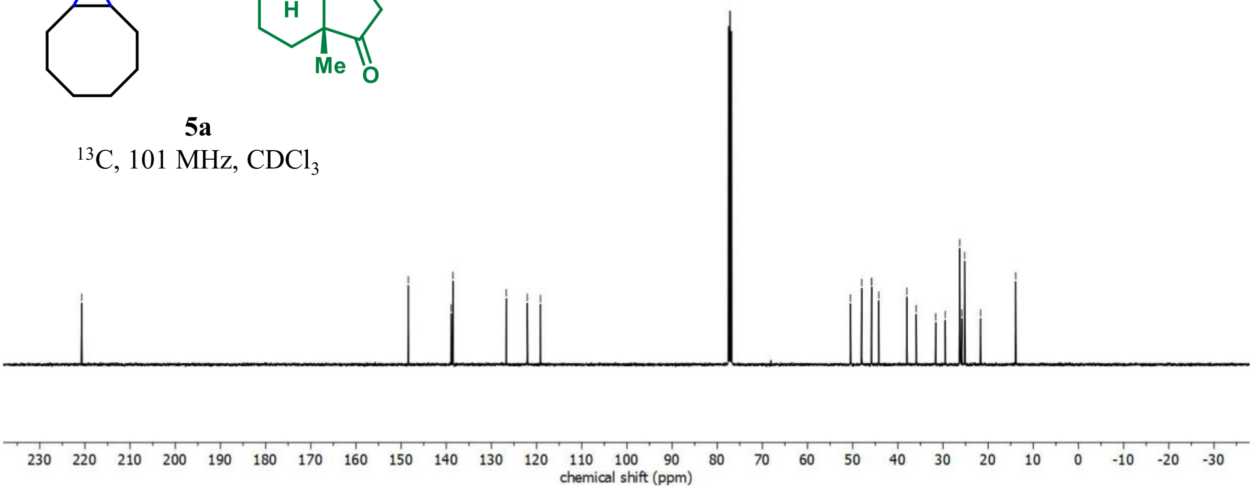

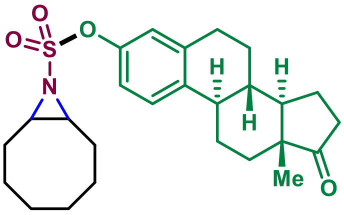

5a

^{19}F, 377 MHz, $CDCl_3$

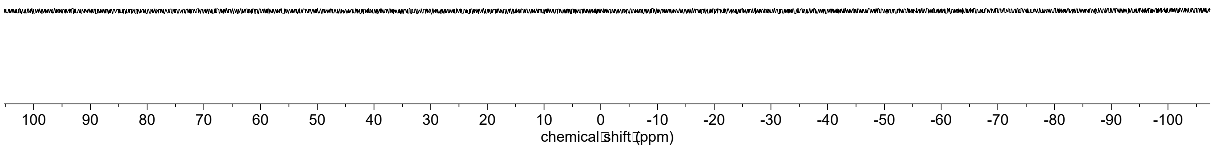

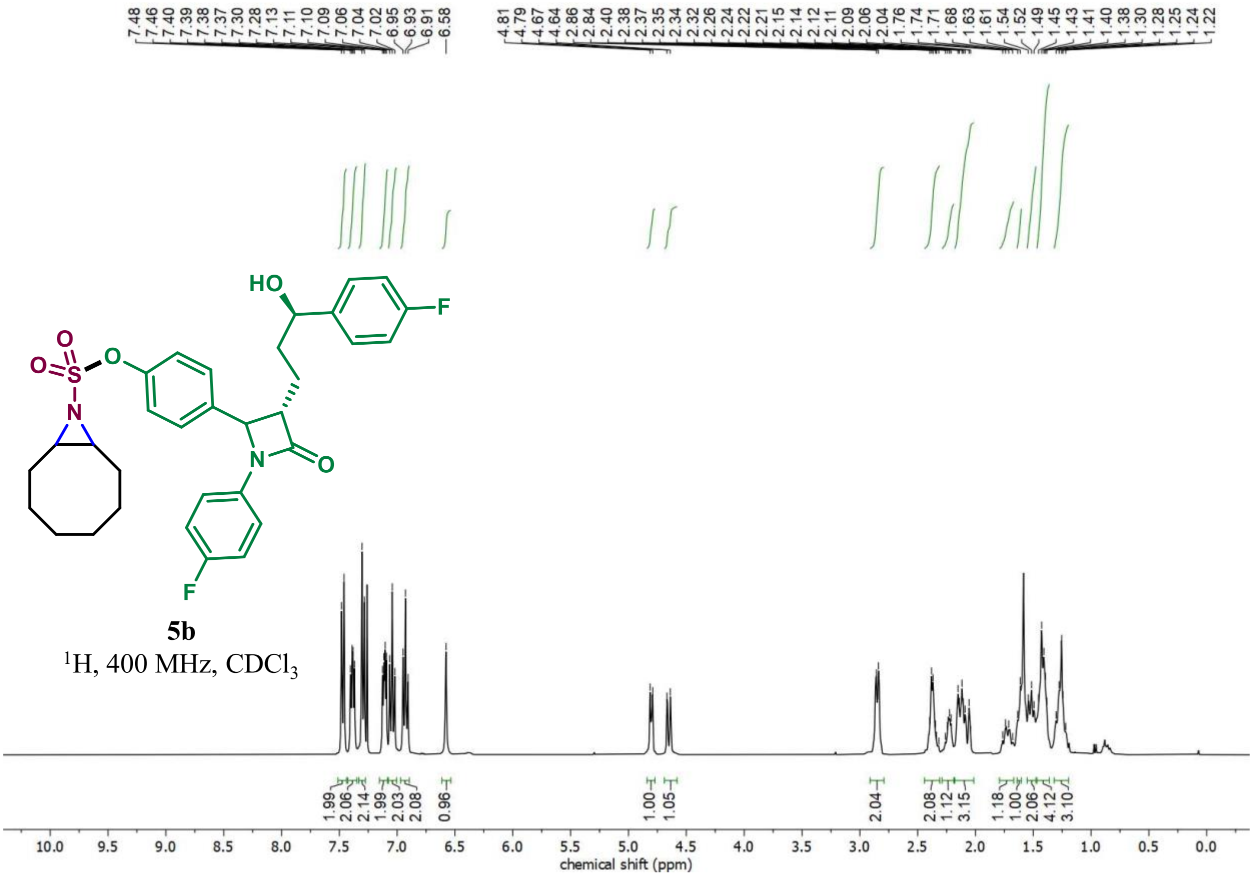

5b

^{1}H, 400 MHz, $CDCl_3$

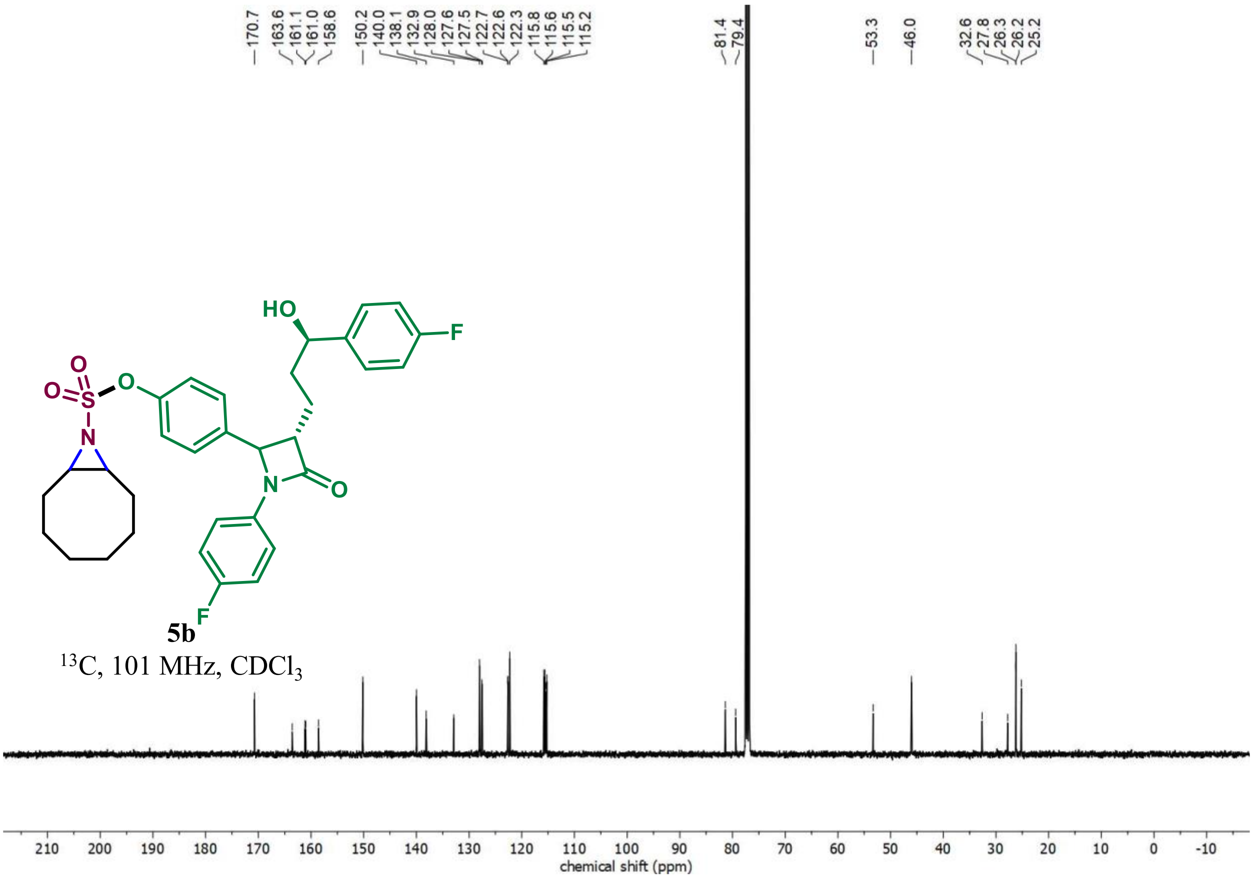

5b

^{13}C, 101 MHz, $CDCl_3$

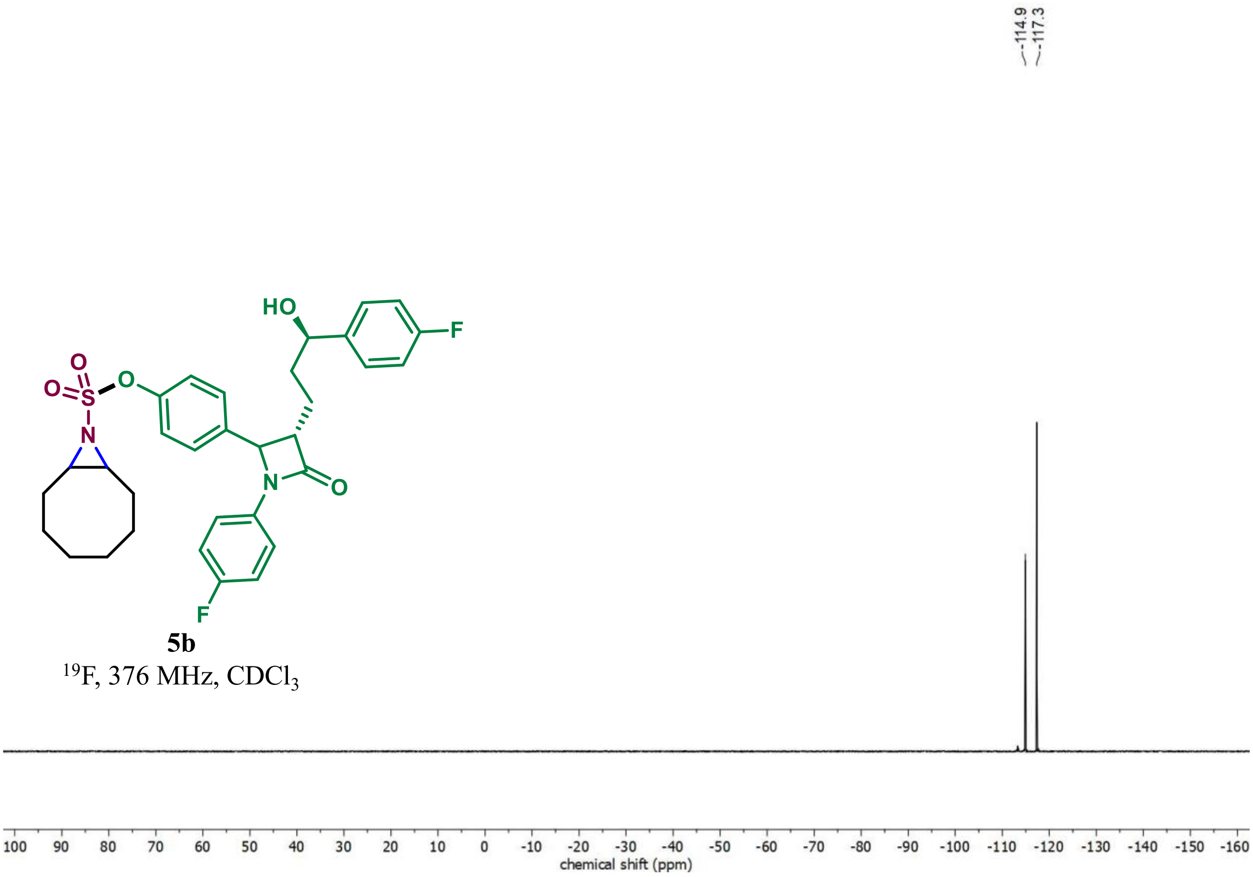
-114.9
-117.3
HO
F
O
O
S
O
N
N
O
F
5b
^{19}F, 376 MHz, CDCl$_3$
chemical shift (ppm)

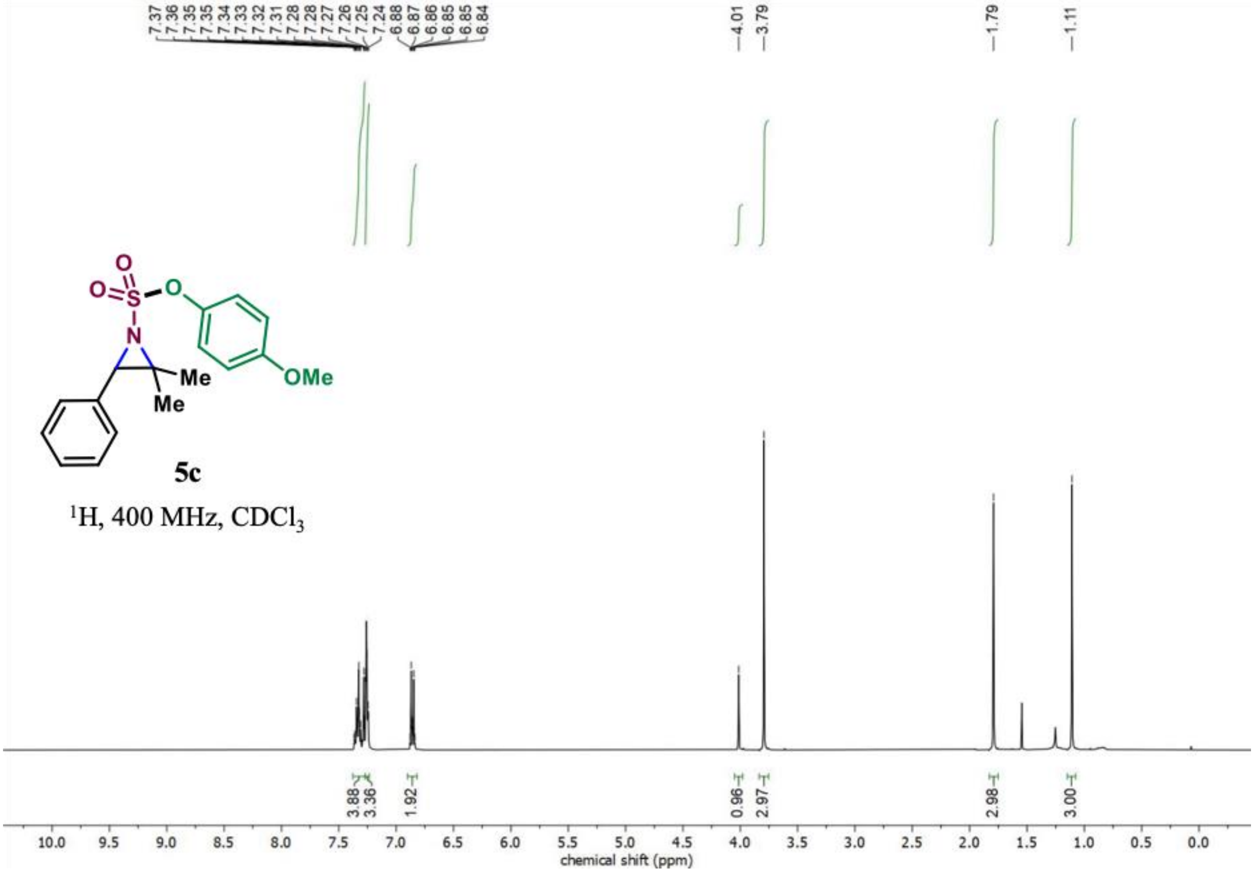
7.37
7.36
7.35
7.35
7.34
7.33
7.32
7.31
7.28
7.28
7.27
7.26
7.25
7.24
6.88
6.87
6.86
6.85
6.85
6.84
4.01
3.79
1.79
1.11
O
O
S
O
N
Me
Me
OMe
5c
^{1}H, 400 MHz, CDCl$_3$
3.88
3.36
1.92
0.96
2.97
2.98
3.00
chemical shift (ppm)

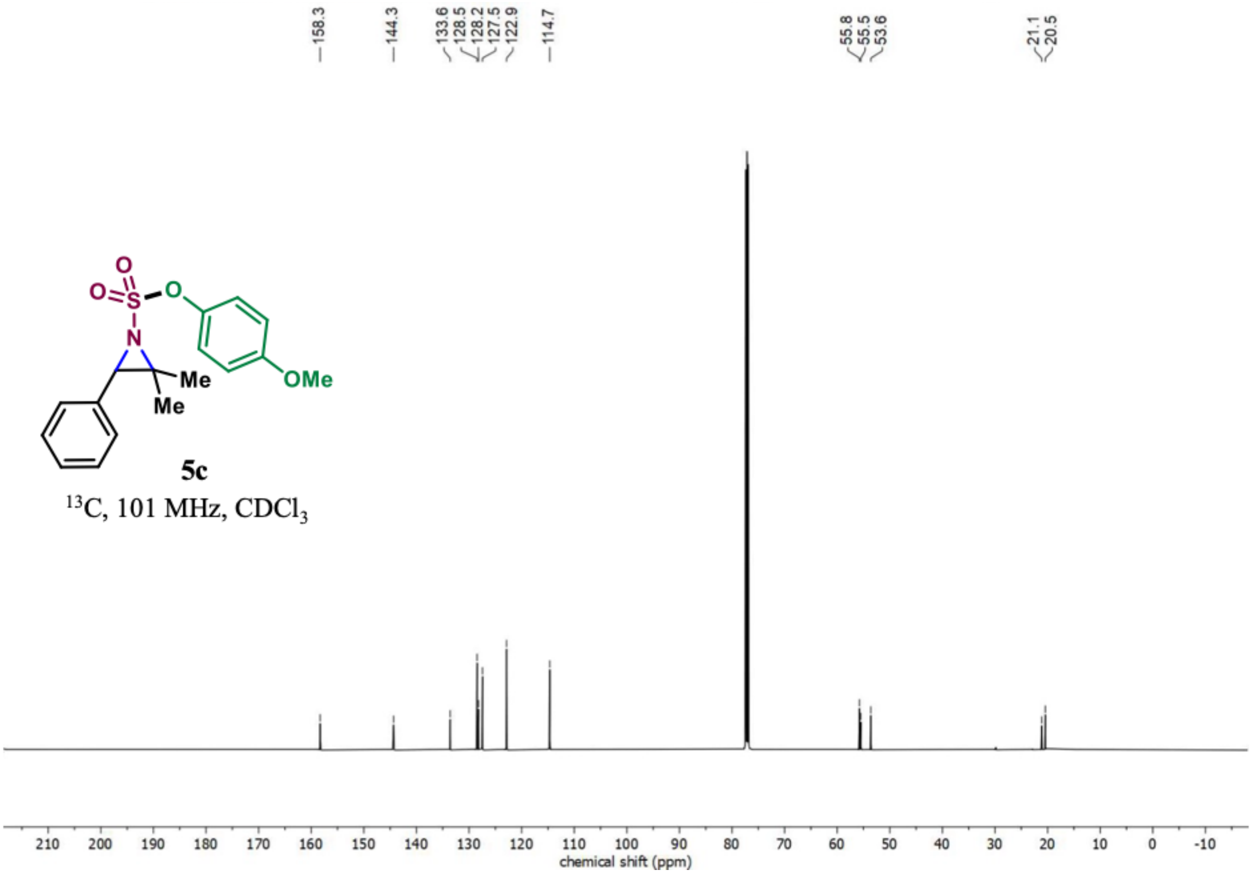

5c

^{13}C, 101 MHz, $CDCl_3$

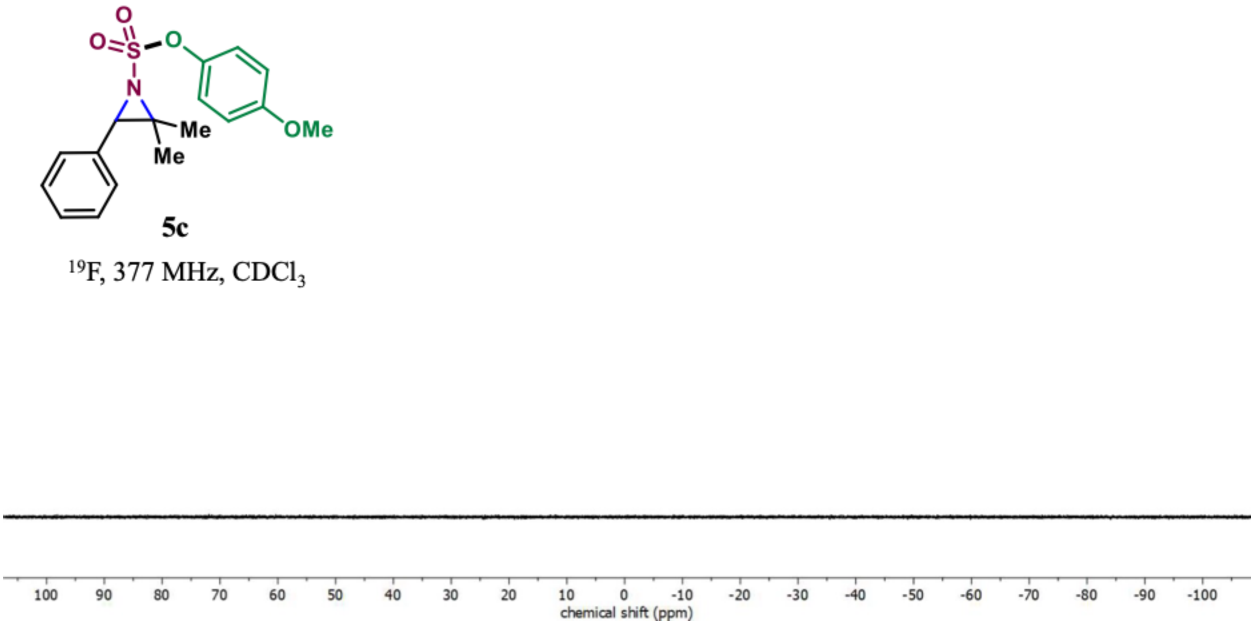

5c

^{19}F, 377 MHz, $CDCl_3$

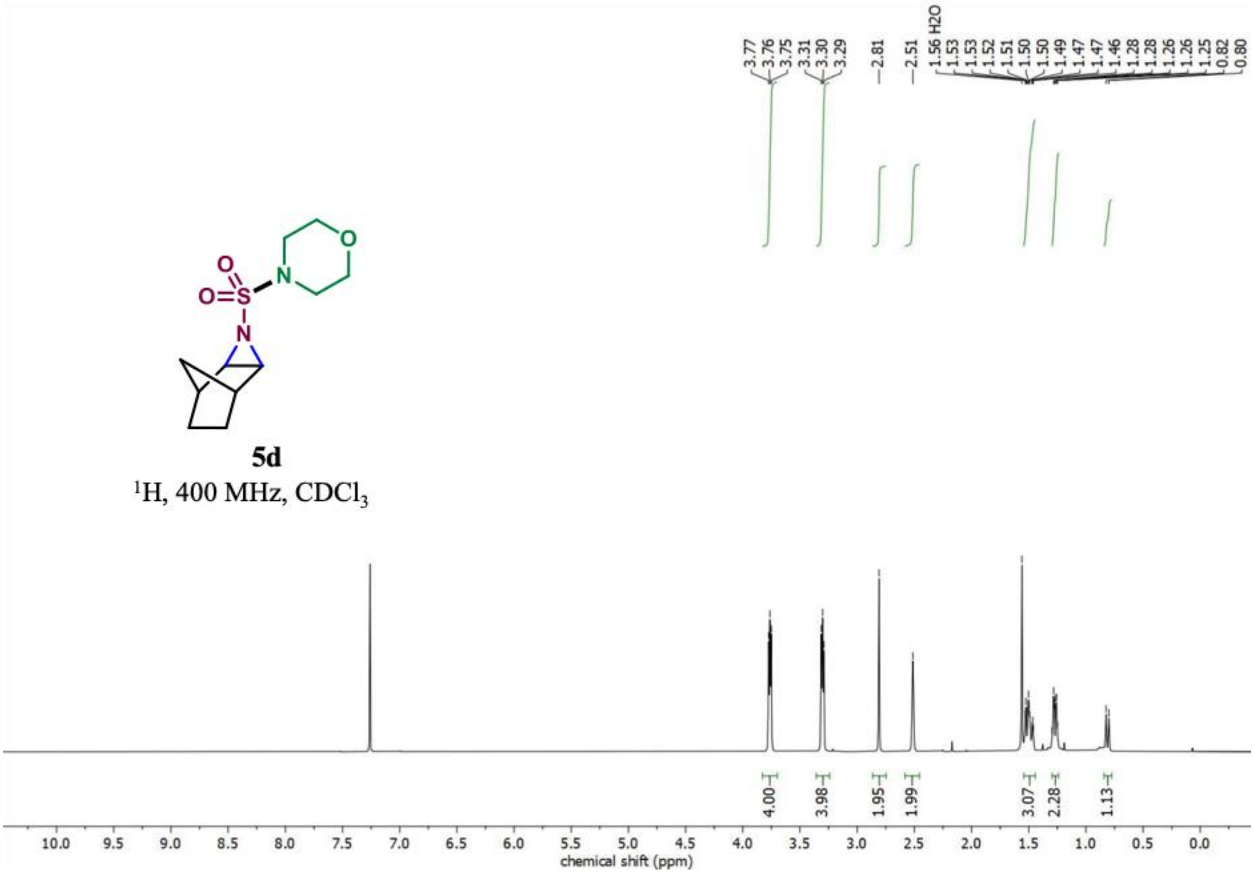

3.77
3.76
3.75
3.31
3.30
3.29
2.81
2.51
1.56 H2O
1.53
1.53
1.52
1.51
1.50
1.50
1.49
1.47
1.47
1.46
1.28
1.28
1.26
1.26
1.25
0.82
0.80
O
N
O
S
O
N
5d
^{1}H, 400 MHz, $CDCl_3$
4.00
3.98
1.95
1.99
3.07
2.28
1.13
chemical shift (ppm)

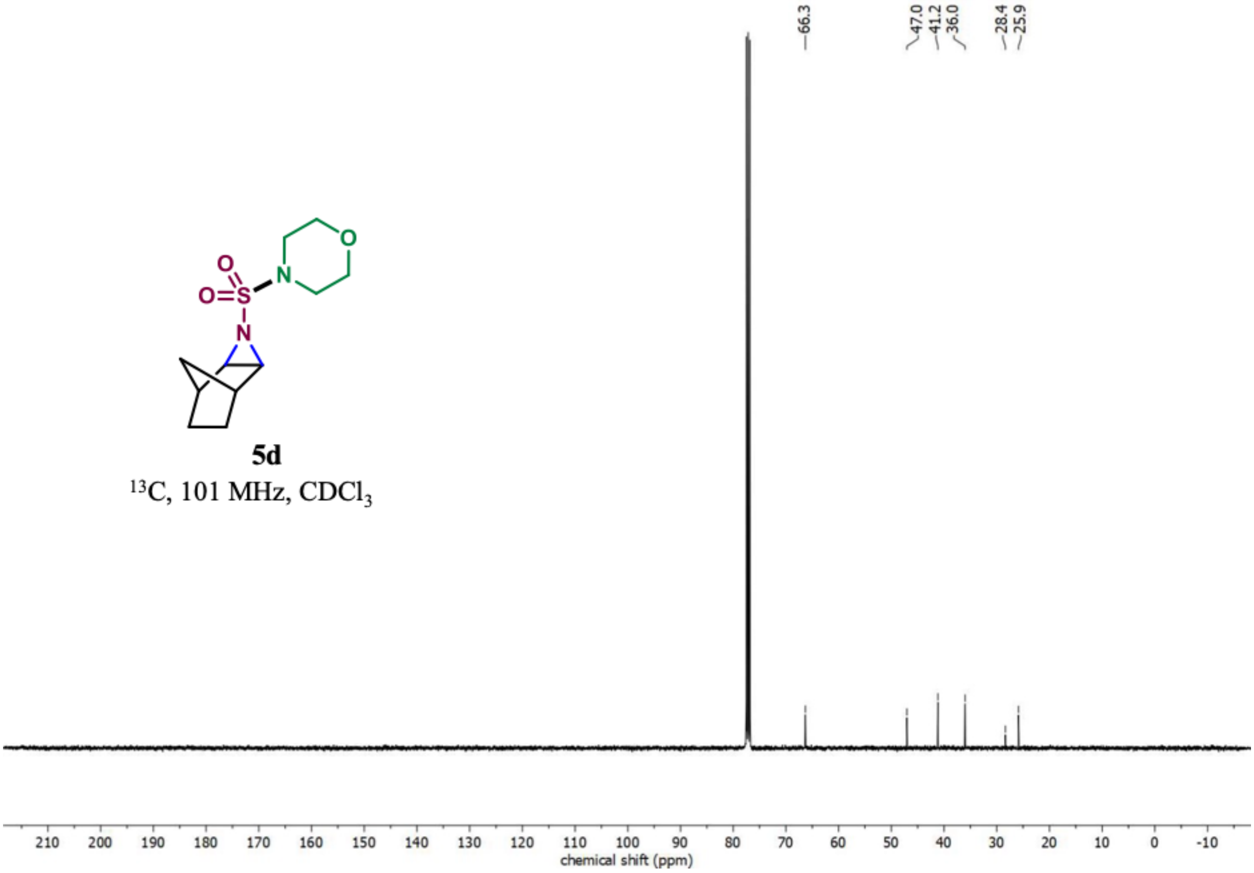

66.3
47.0
41.2
36.0
28.4
25.9
O
N
O
S
O
N
5d
^{13}C, 101 MHz, $CDCl_3$
chemical shift (ppm)

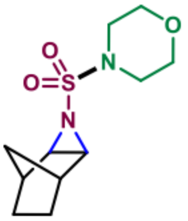

5d

^{19}F, 377 MHz, $CDCl_3$

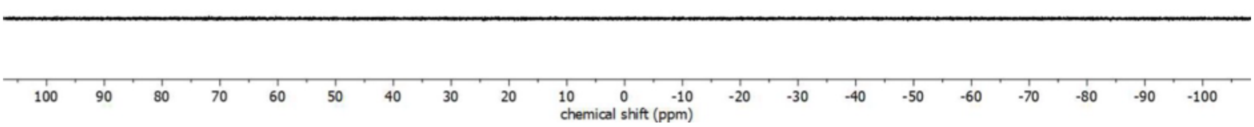

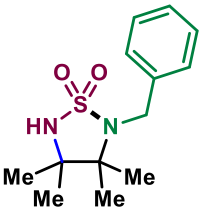

5e

^{1}H, 400 MHz, $CDCl_3$

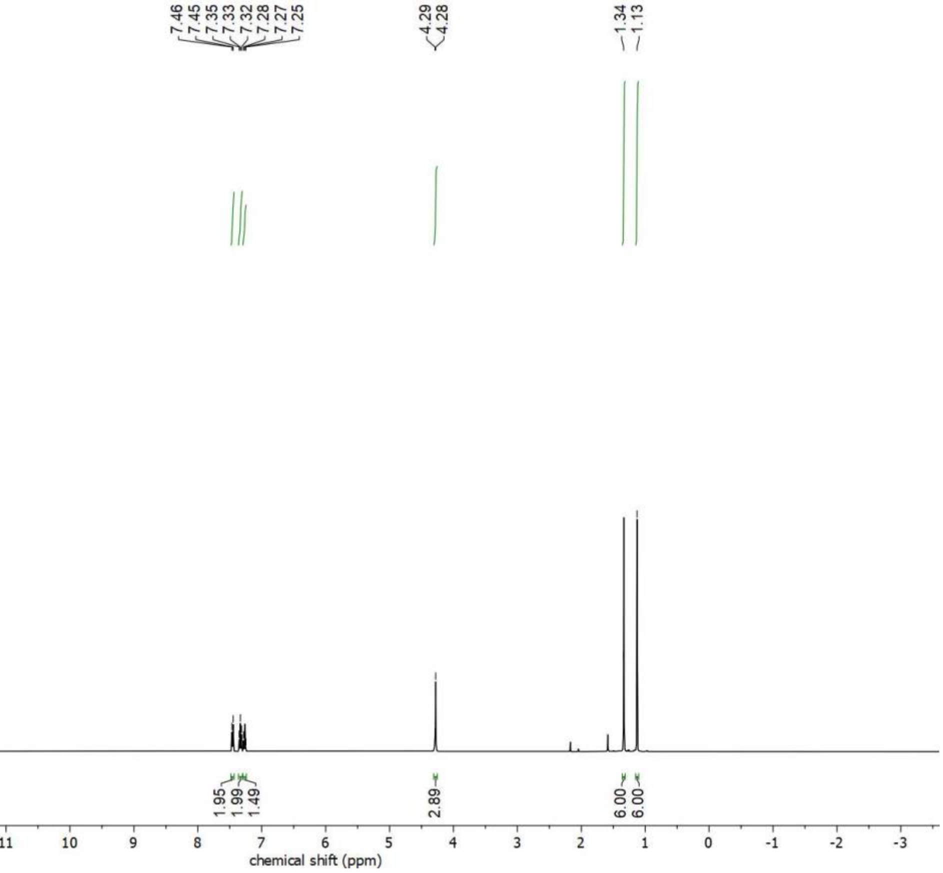

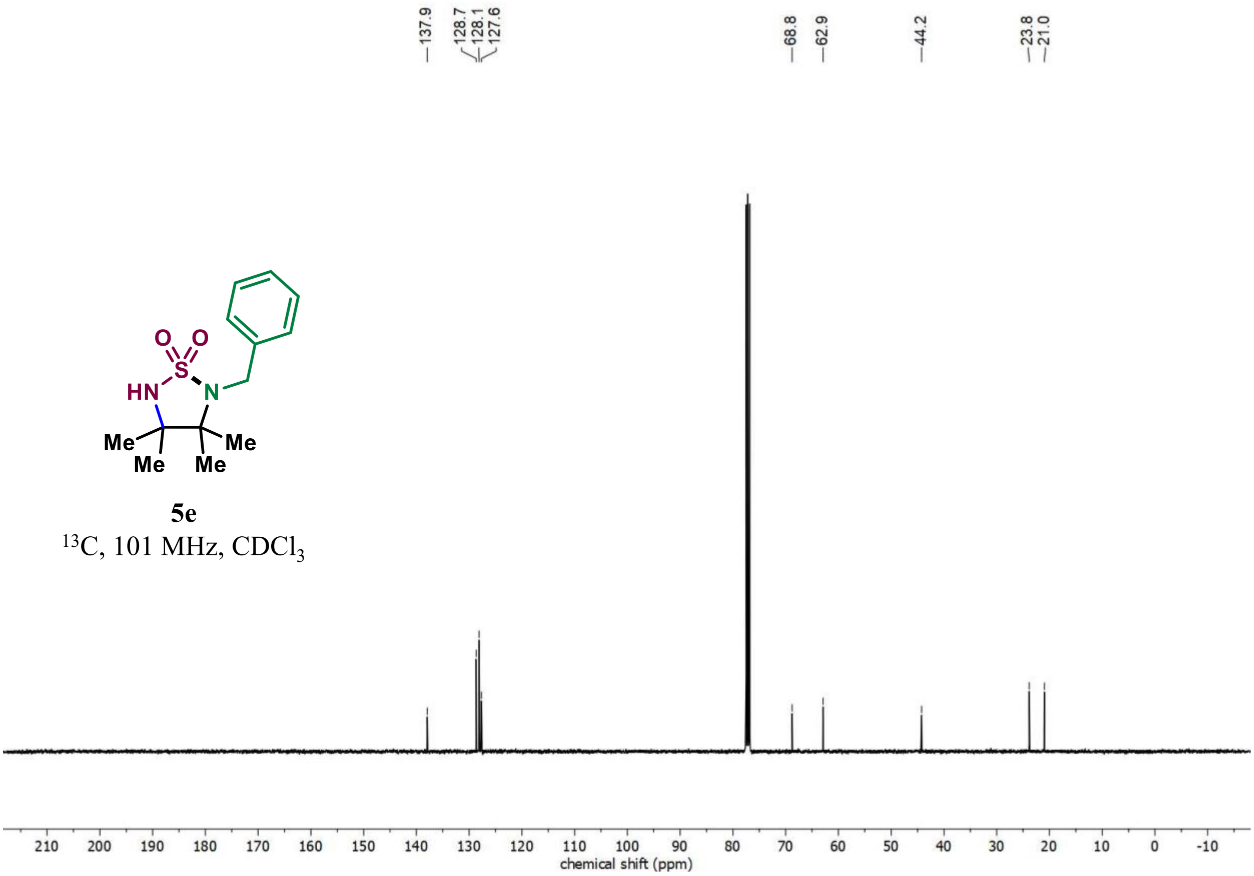

5e
^{13}C, 101 MHz, $CDCl_3$

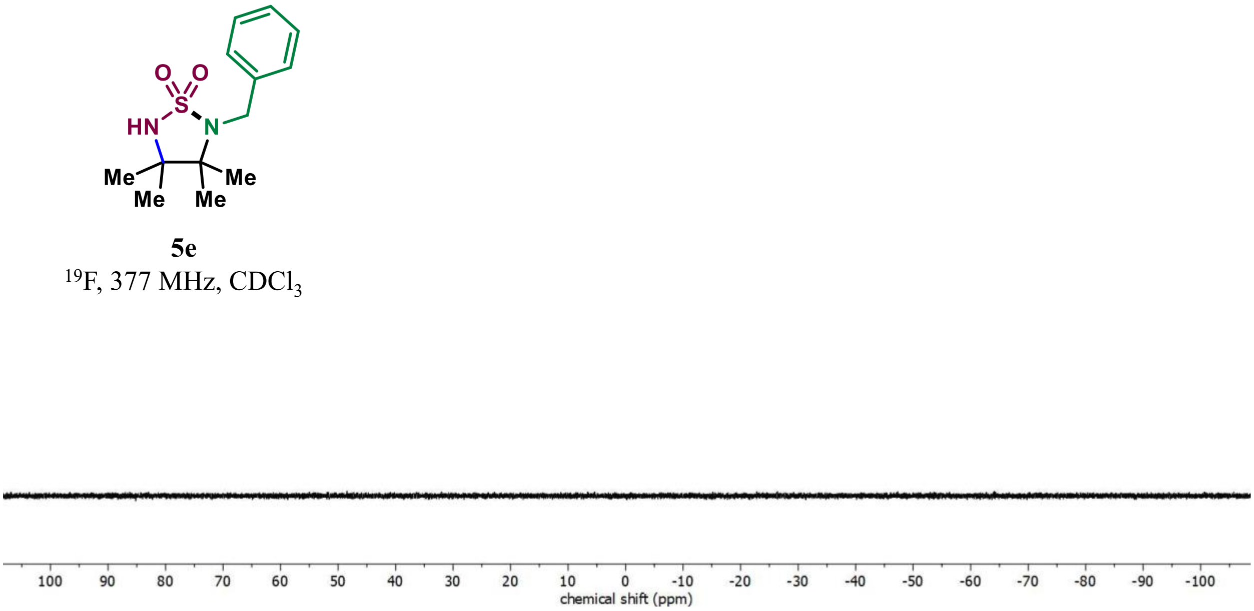

5e
^{19}F, 377 MHz, $CDCl_3$

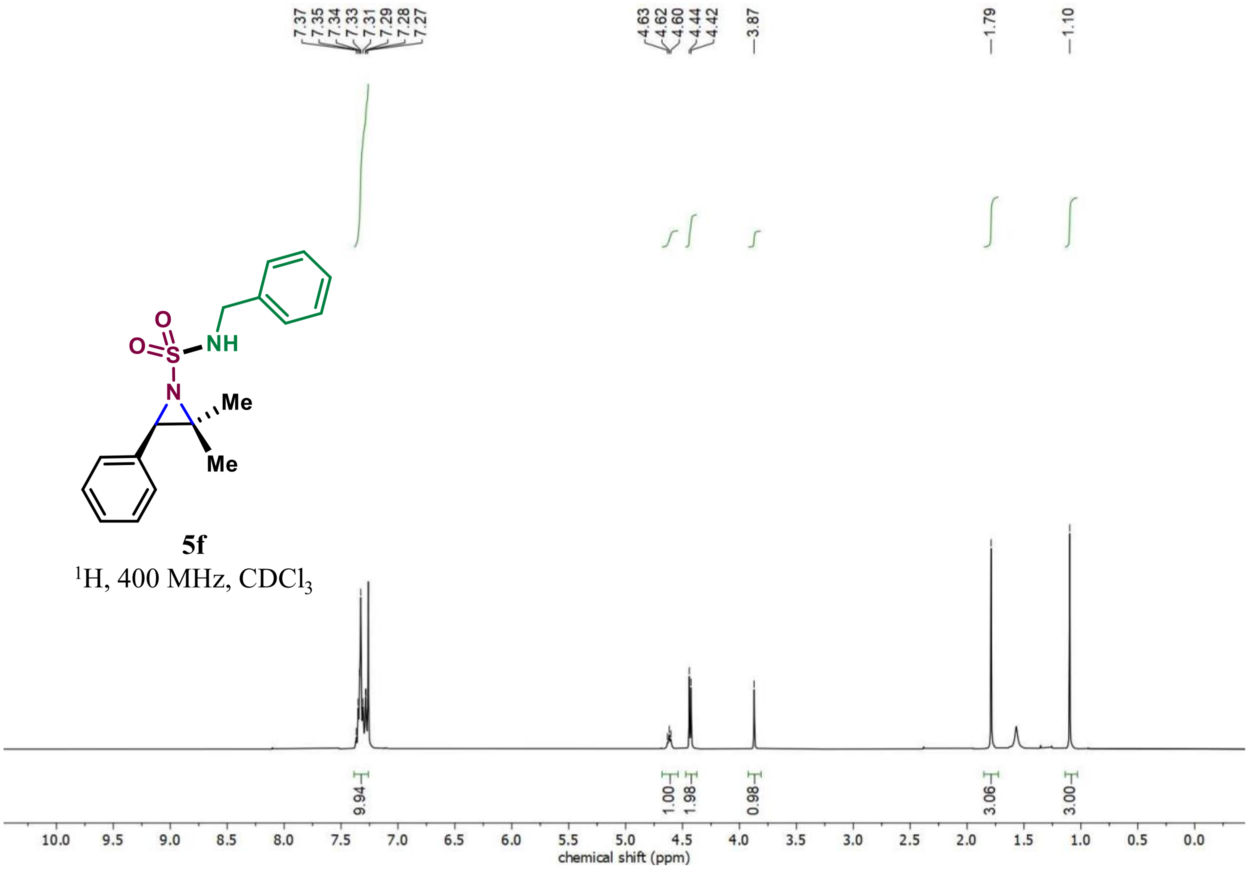

5f

^{1}H, 400 MHz, $CDCl_3$

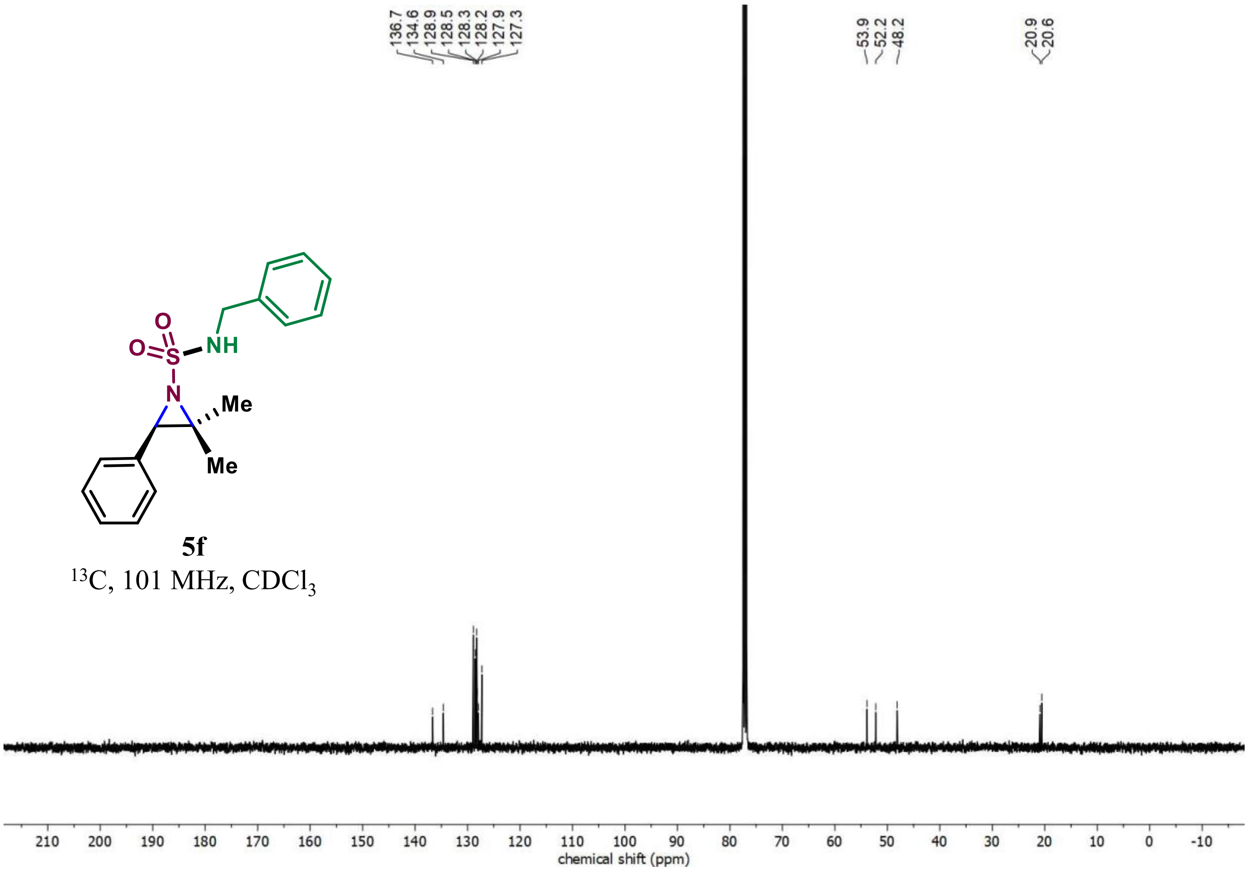

5f

^{13}C, 101 MHz, $CDCl_3$

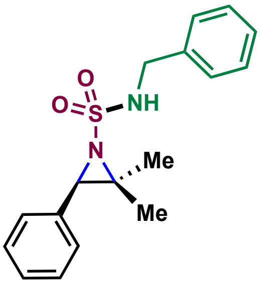

5f

^{19}F, 377 MHz, $CDCl_3$

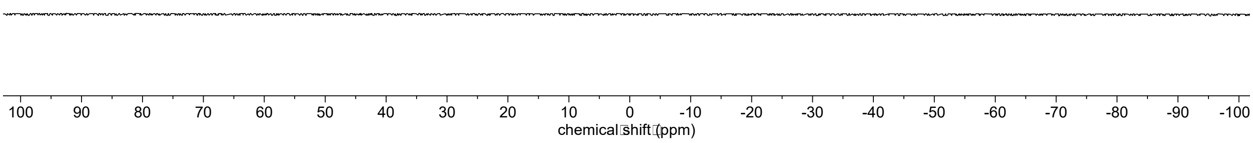

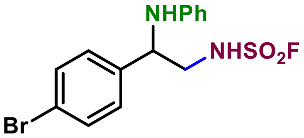

6a

^{1}H, 400 MHz, $CDCl_3$

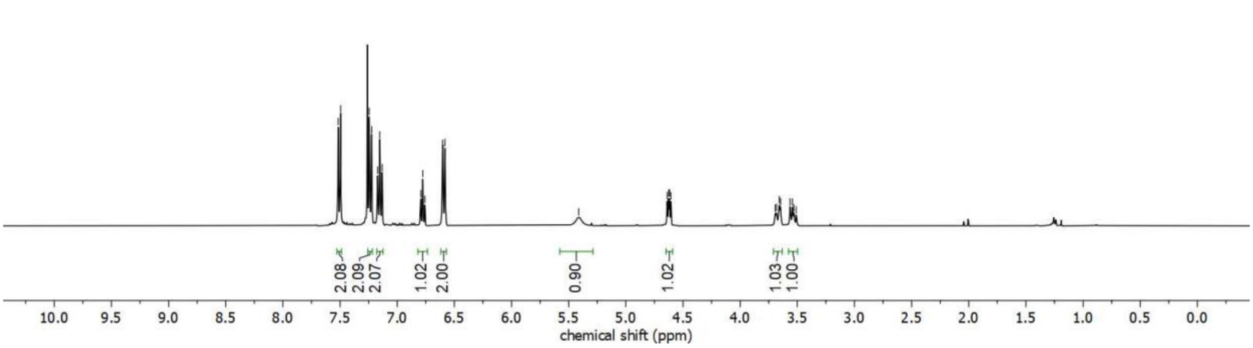

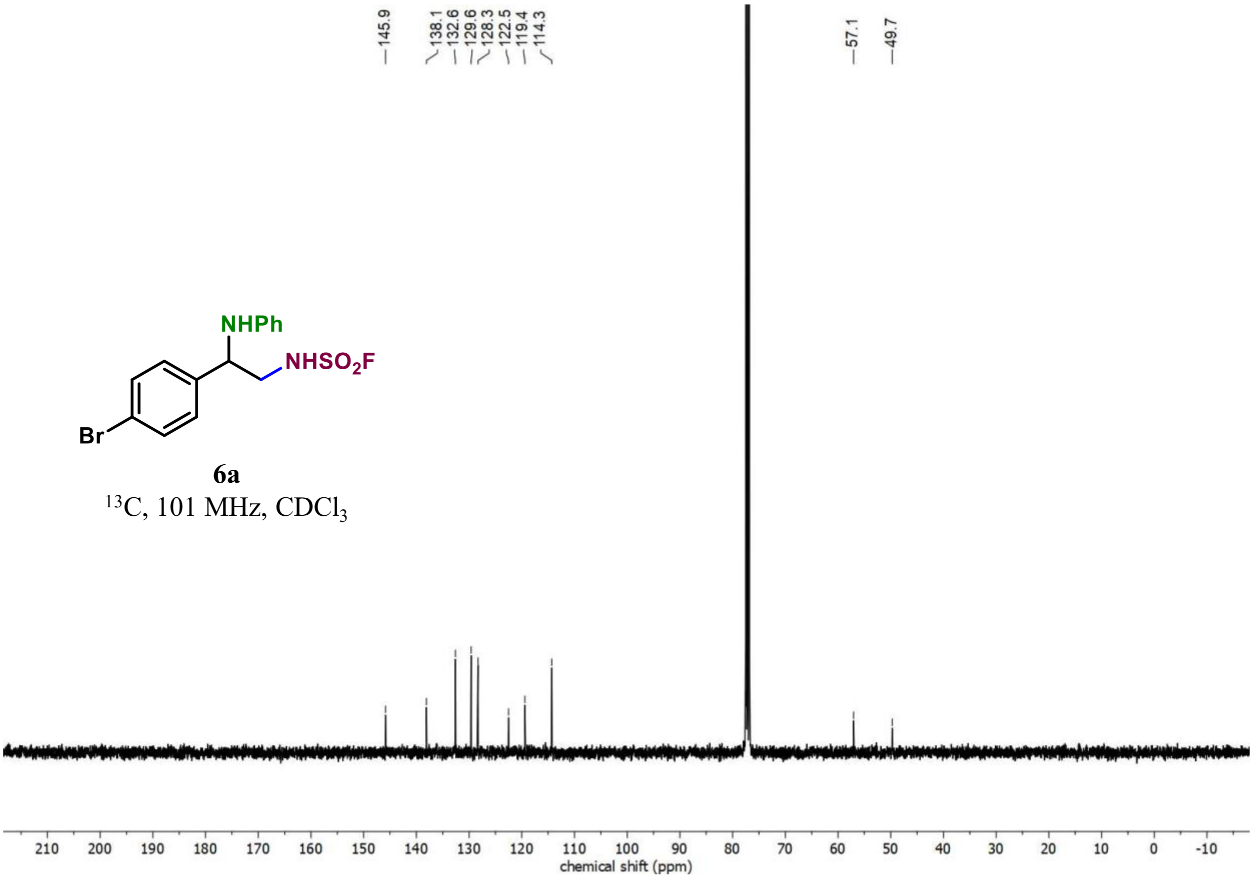

6a

^{13}C, 101 MHz, $CDCl_3$

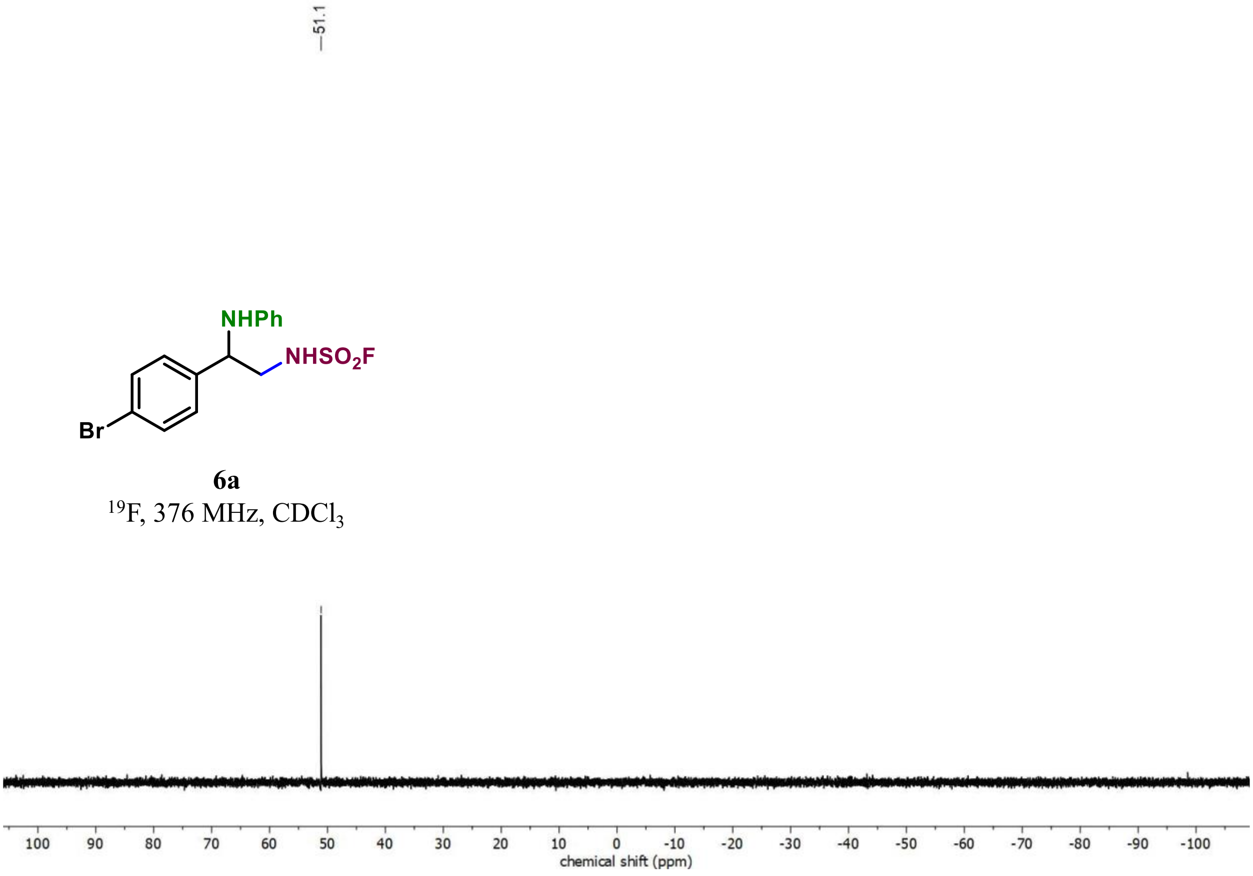

6a

^{19}F, 376 MHz, $CDCl_3$

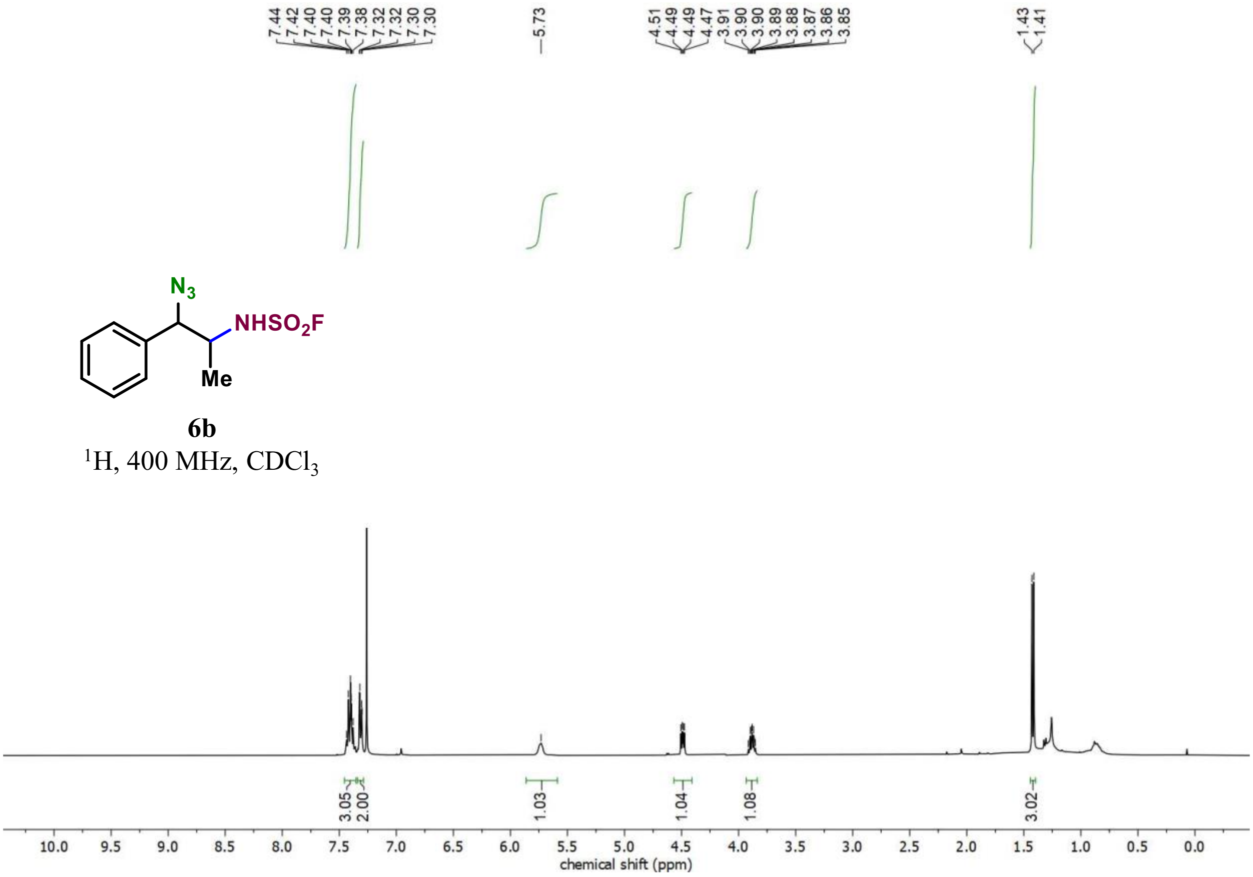

6b

^{1}H, 400 MHz, $CDCl_3$

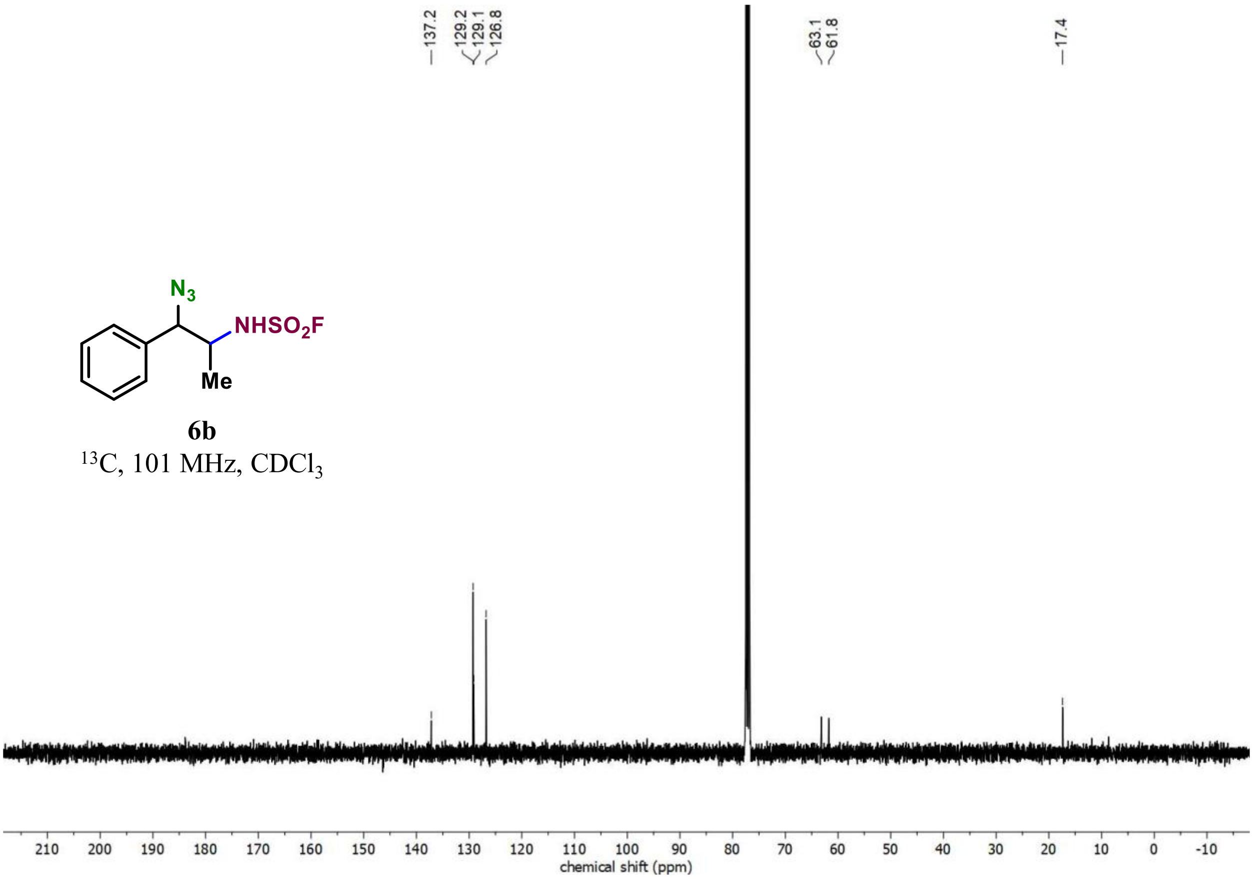

6b

^{13}C, 101 MHz, $CDCl_3$

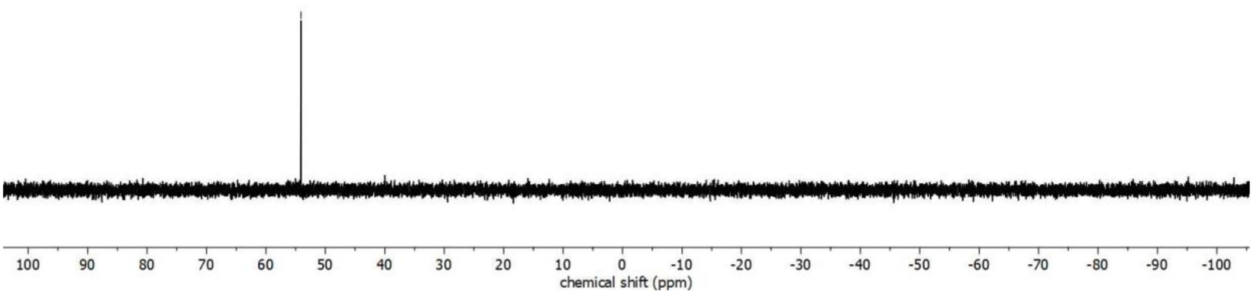

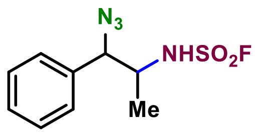

6b

^{19}F, 376 MHz, $CDCl_3$

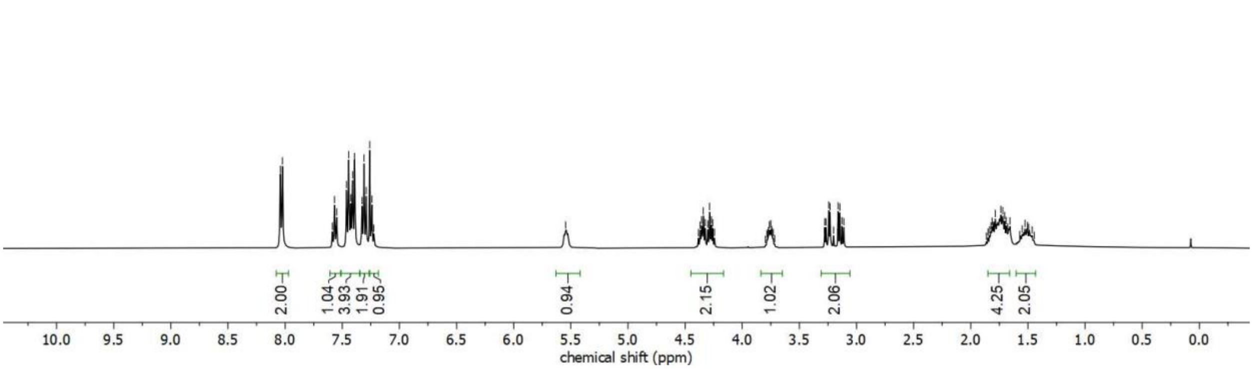

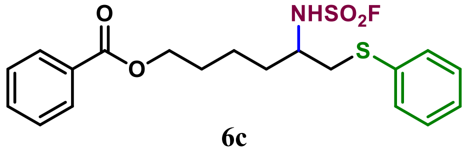

6c

^{1}H, 400 MHz, $CDCl_3$

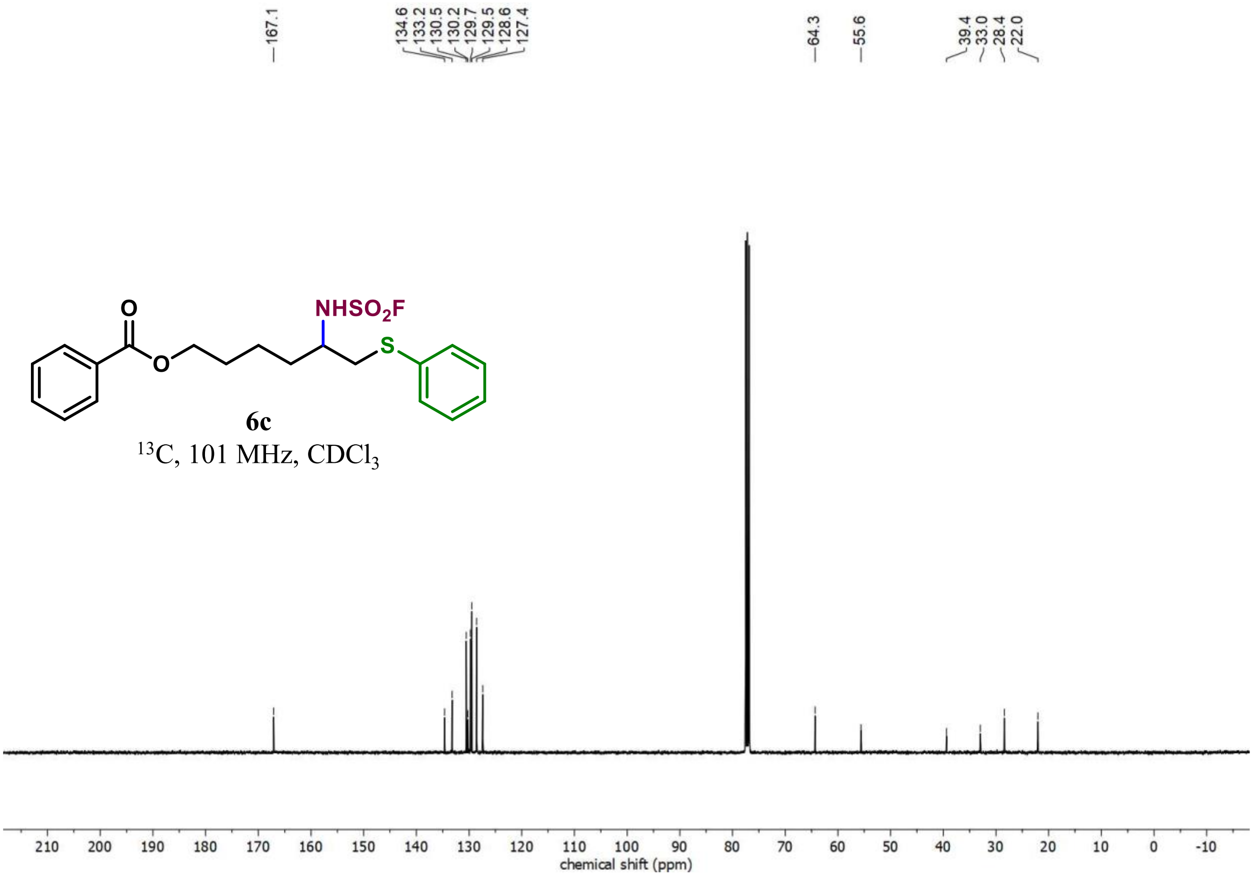

6c

^{13}C, 101 MHz, $CDCl_3$

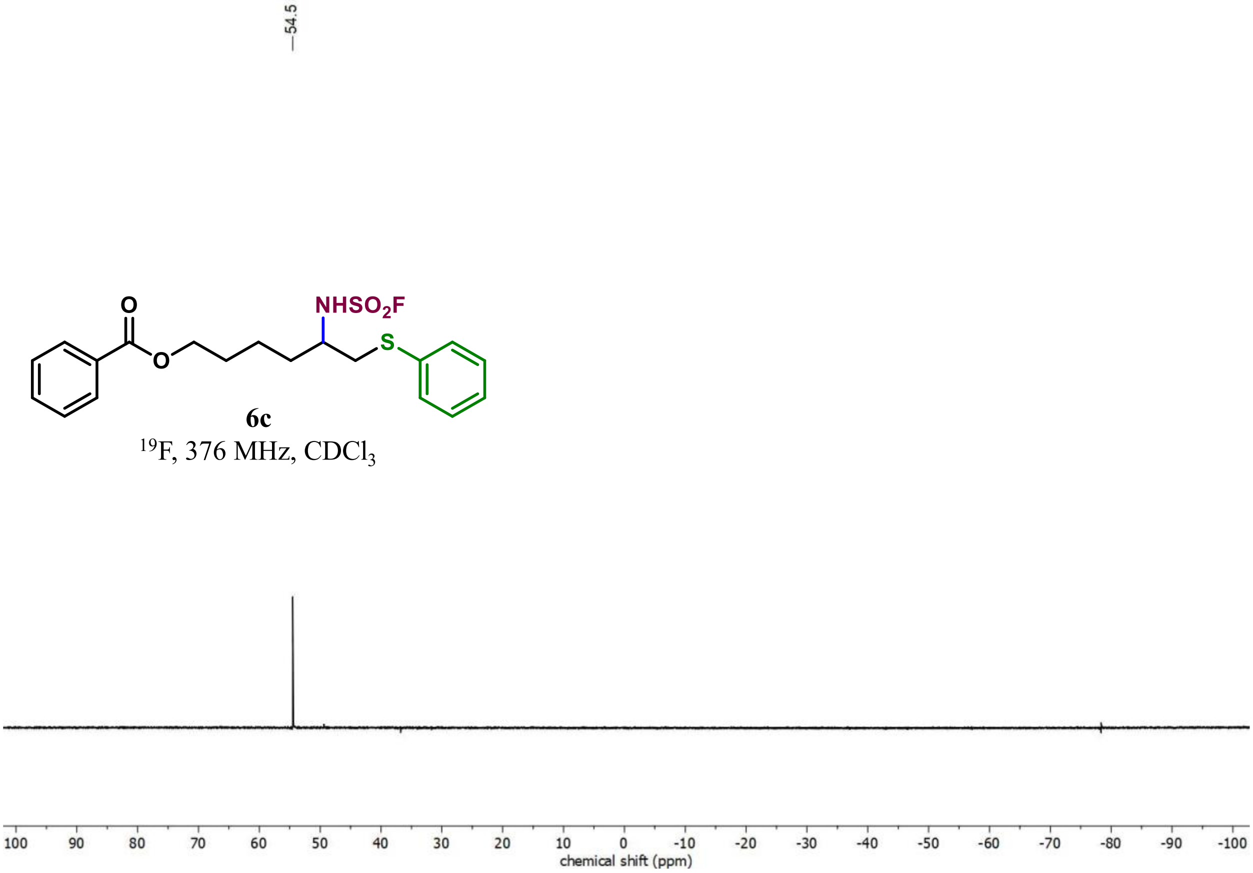

6c

^{19}F, 376 MHz, $CDCl_3$

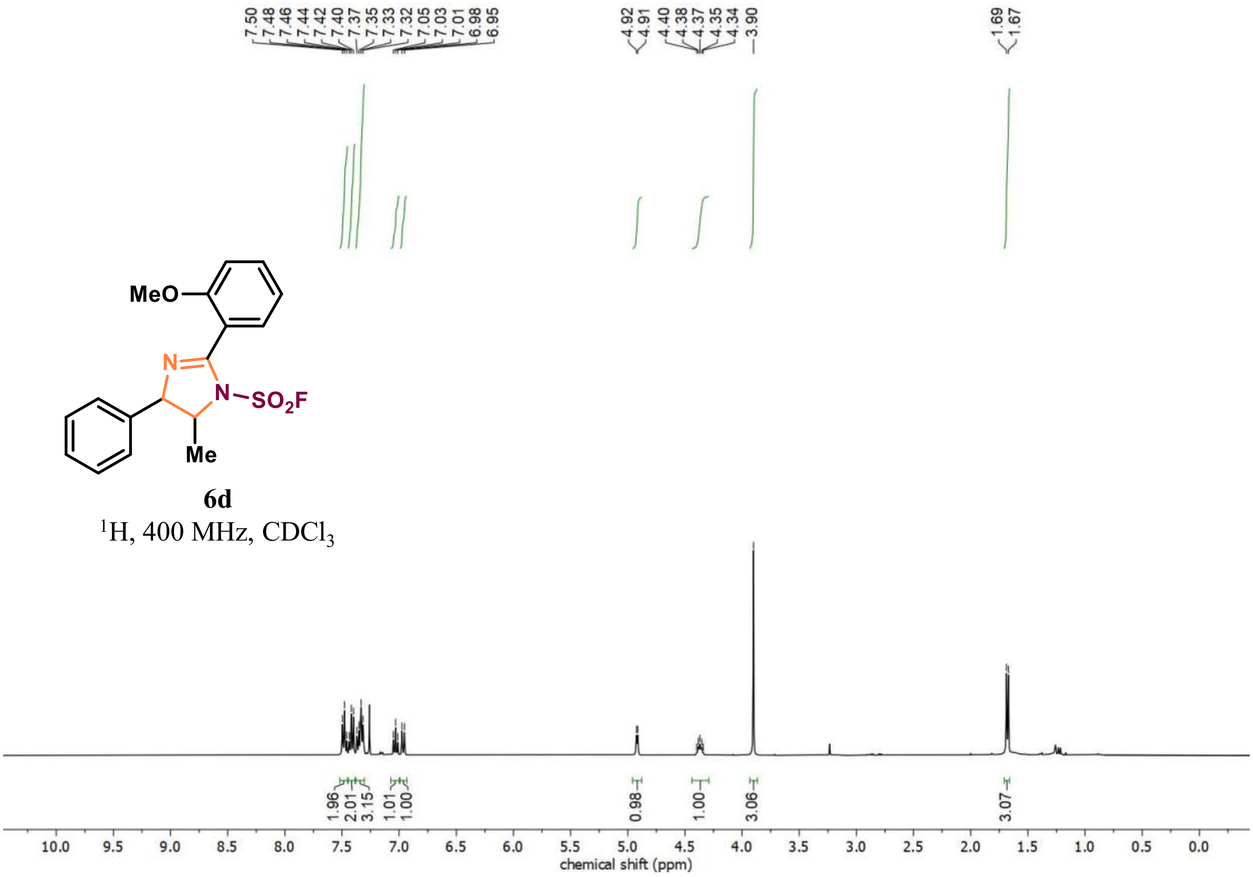

6d

^{1}H, 400 MHz, $CDCl_3$

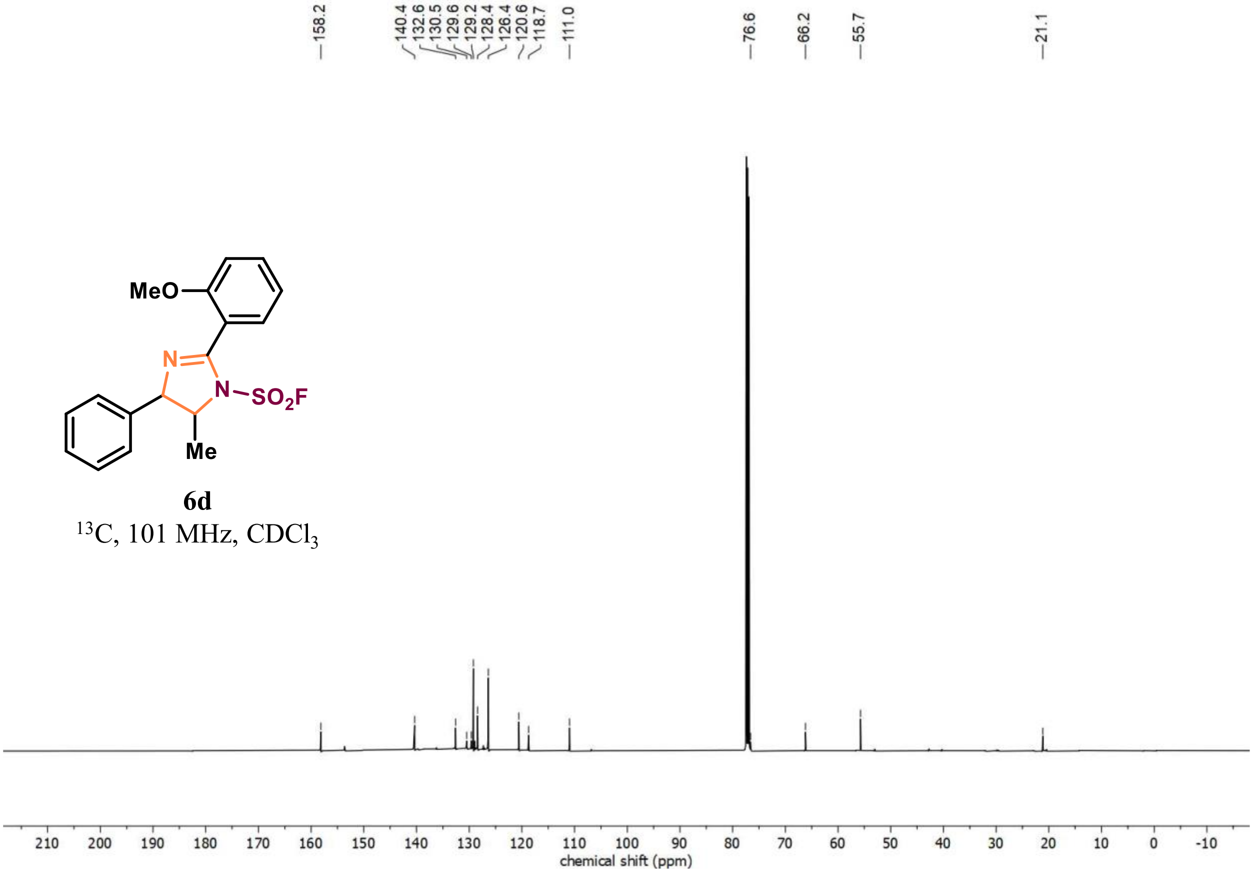

6d

^{13}C, 101 MHz, $CDCl_3$

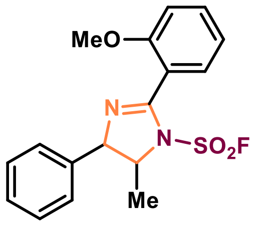

6d

^{19}F, 376 MHz, $CDCl_3$

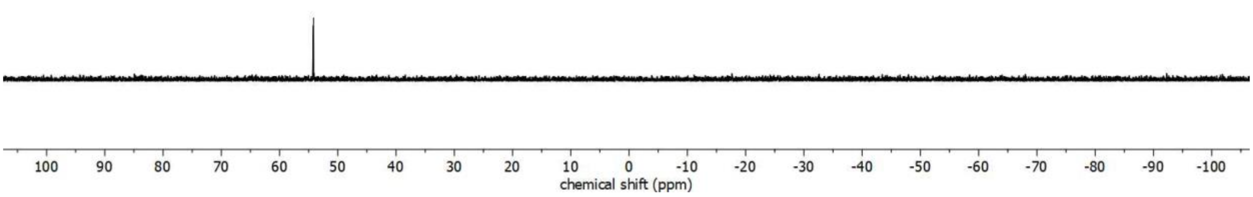

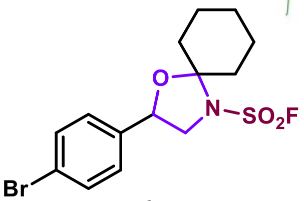

6e

^{1}H, 400 MHz, $CDCl_3$

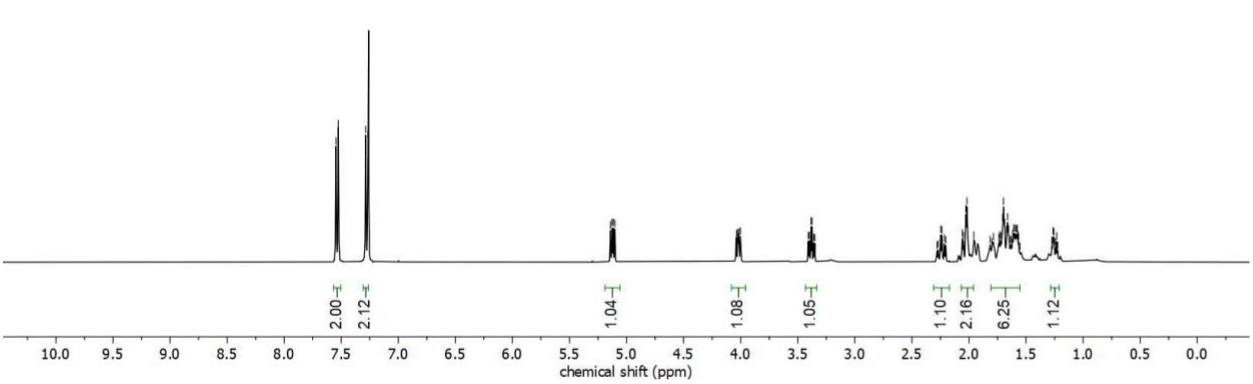

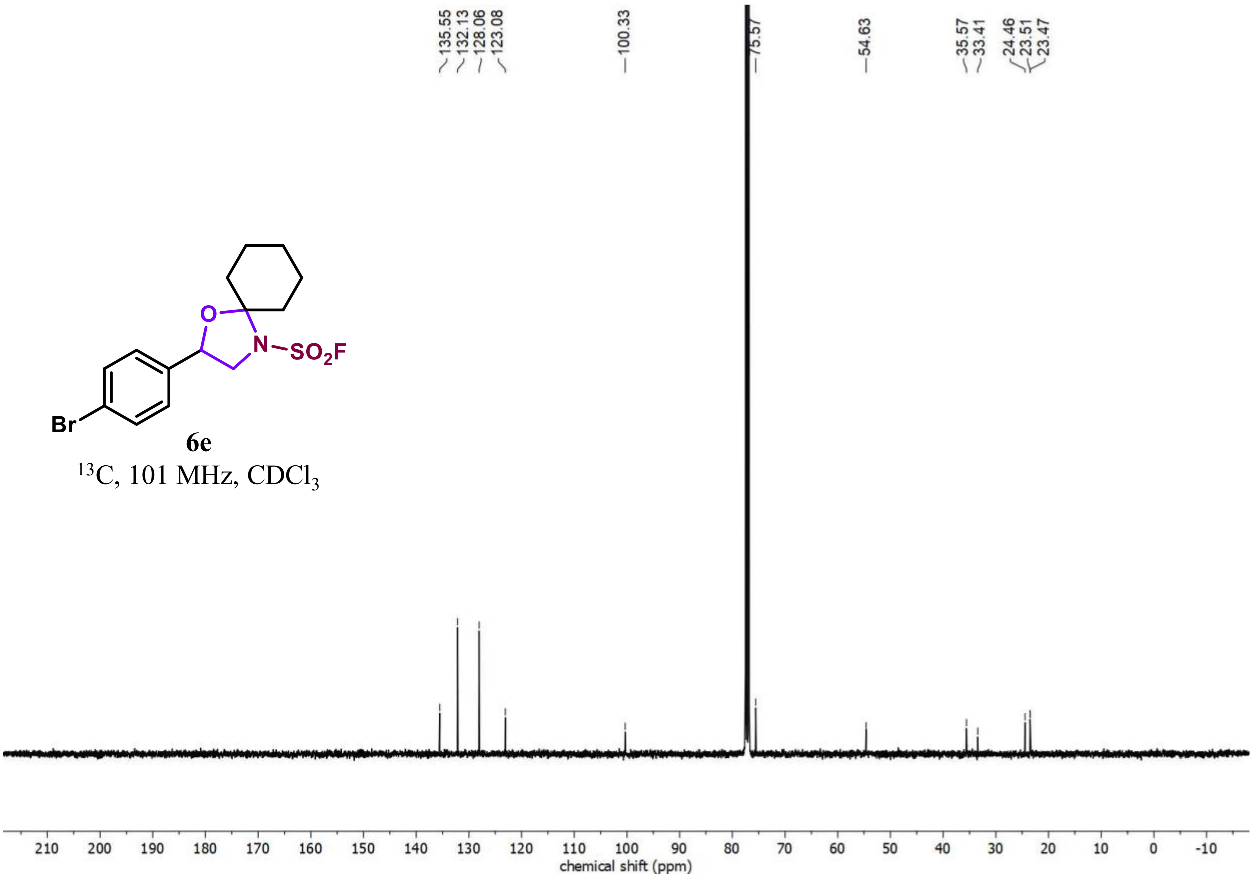

6e

^{13}C, 101 MHz, $CDCl_3$

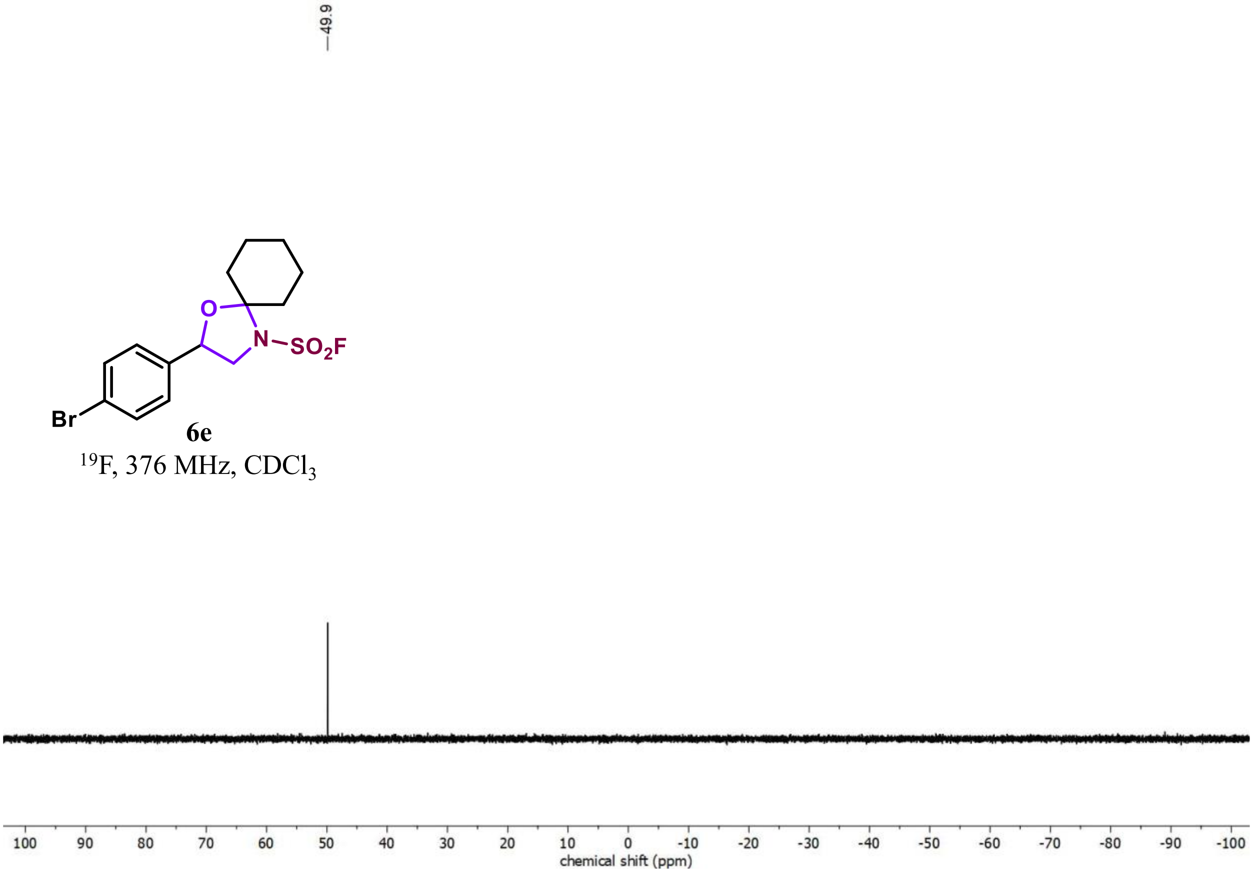

6e

^{19}F, 376 MHz, $CDCl_3$

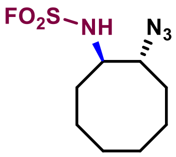

6f

^{1}H, 400 MHz, $CDCl_3$

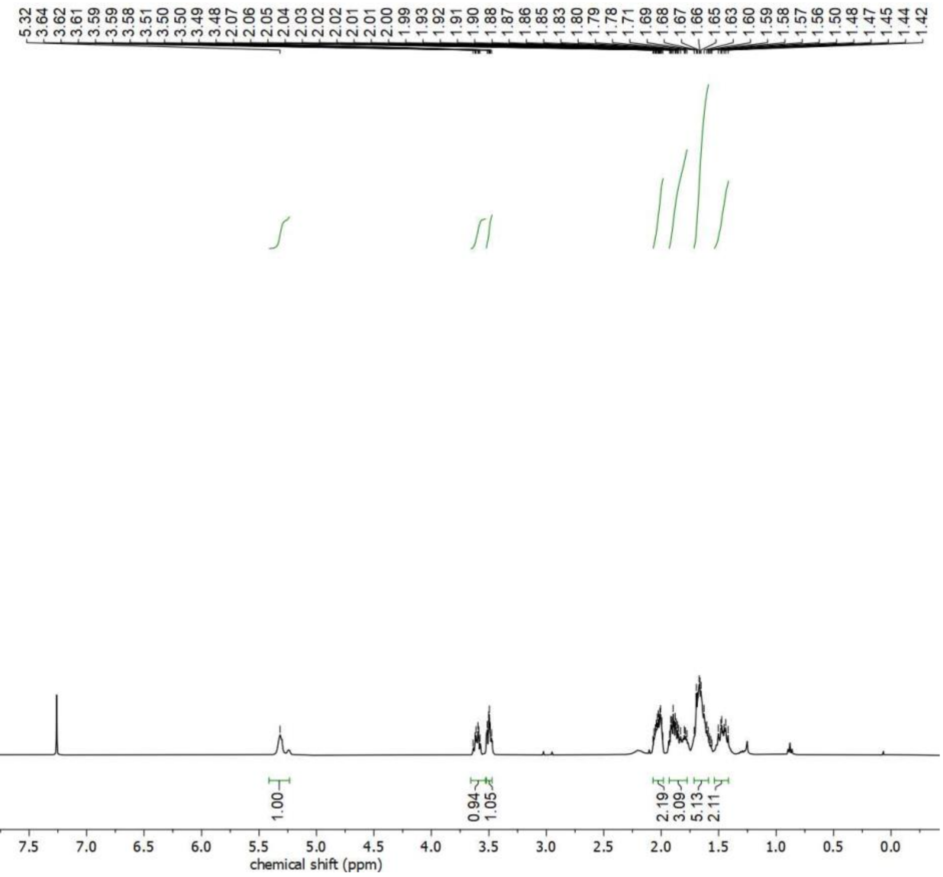

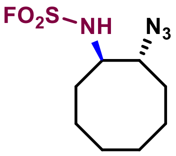

6f

^{13}C, 126 MHz, $CDCl_3$

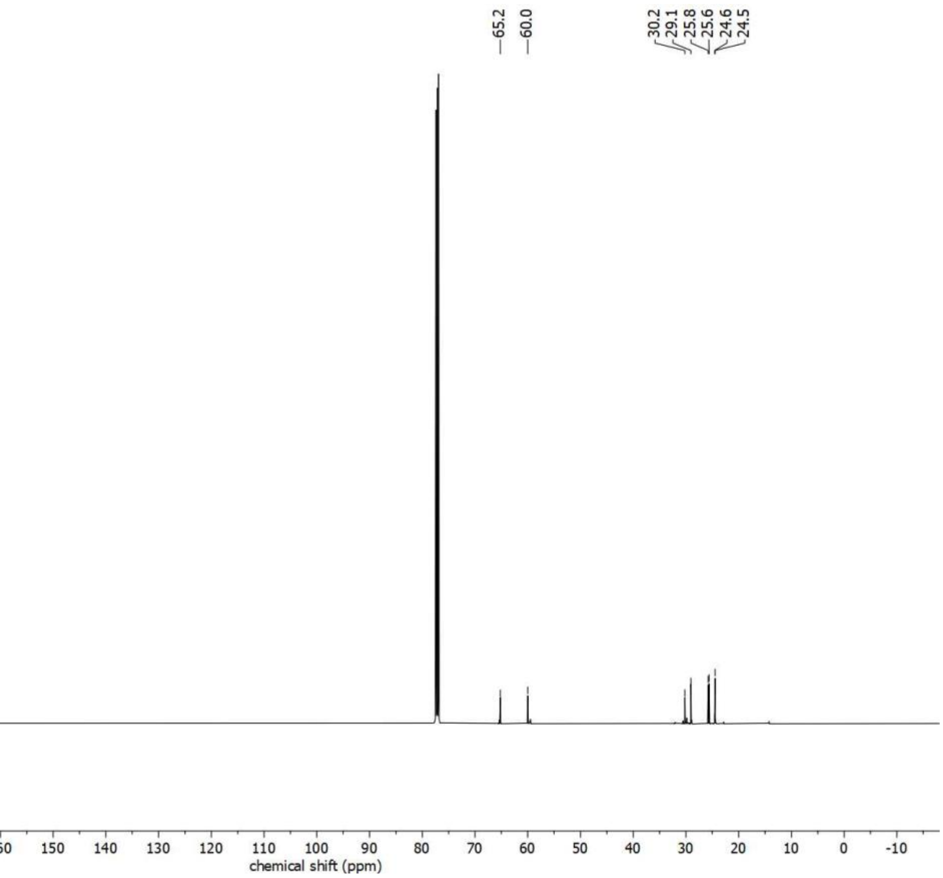

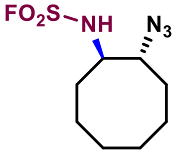

6f

^{19}F, 376 MHz, $CDCl_3$

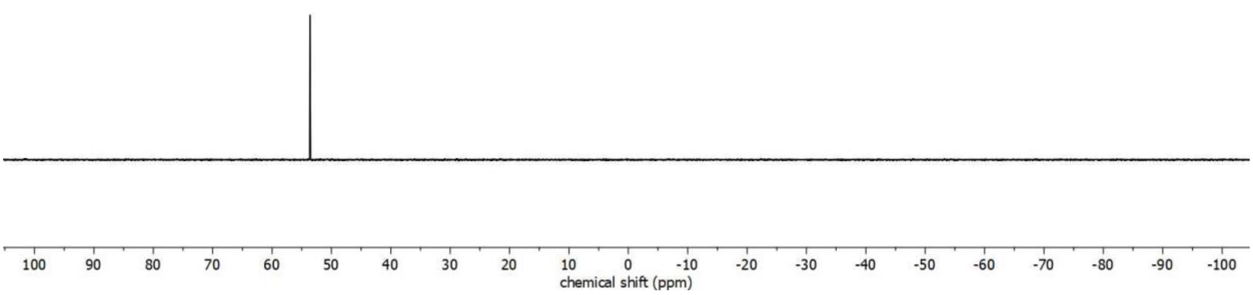

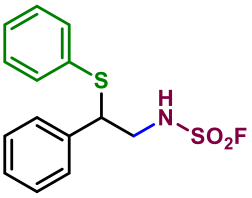

6g

^{1}H, 400 MHz, $CDCl_3$

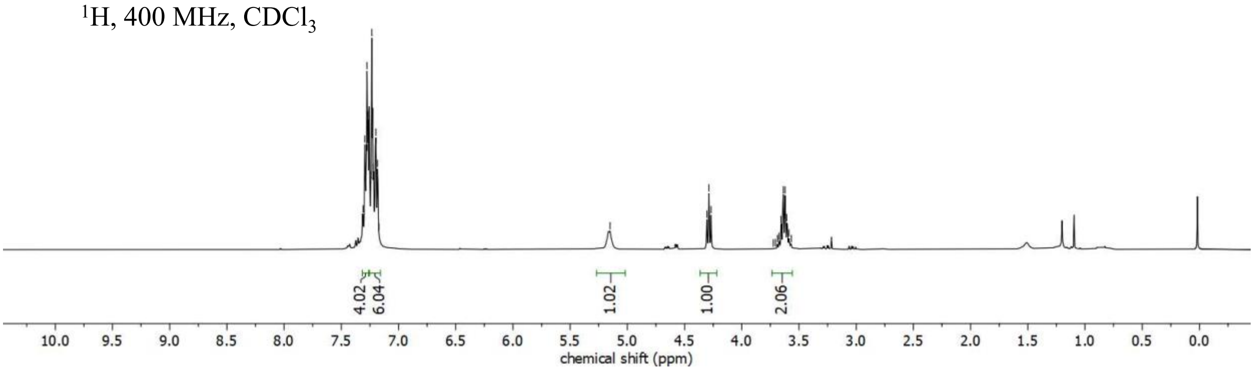

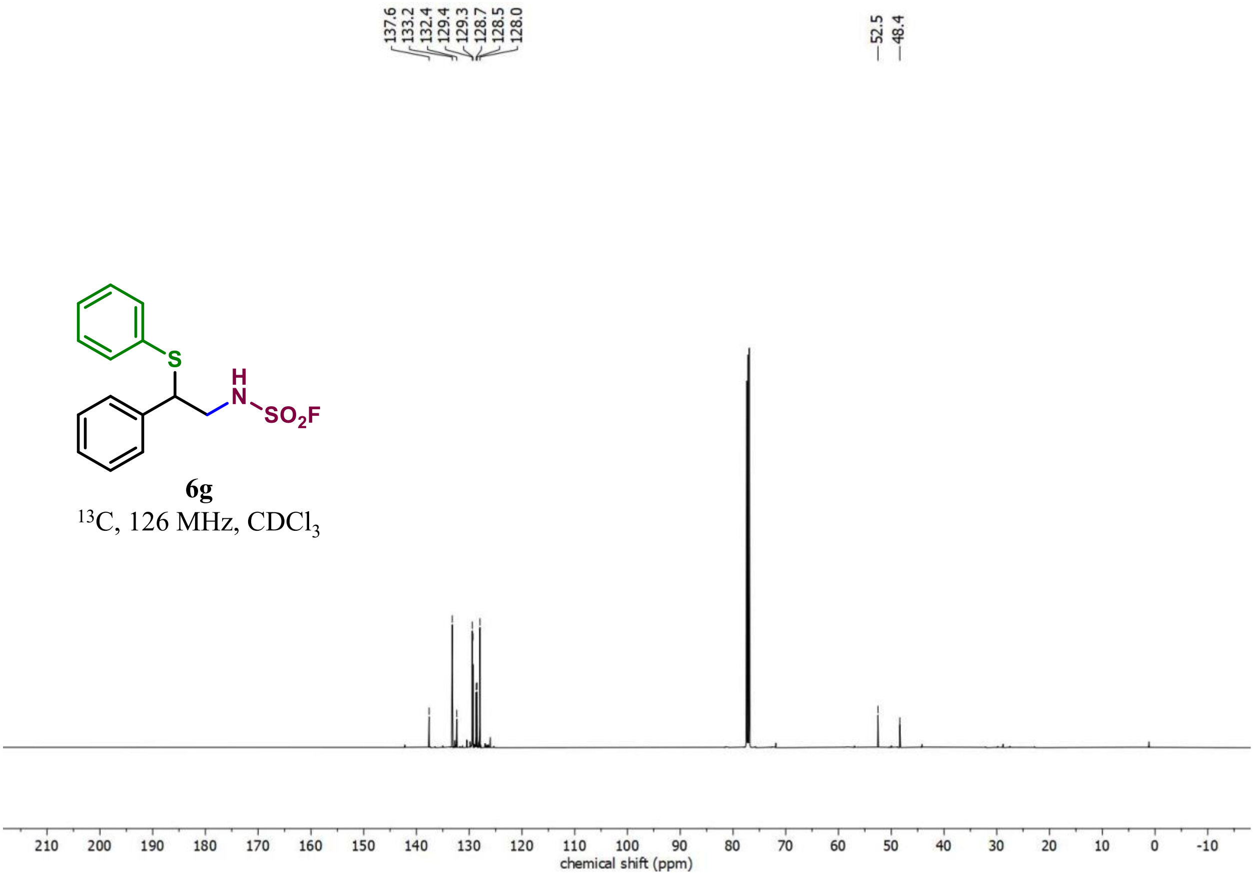

6g

^{13}C, 126 MHz, $CDCl_3$

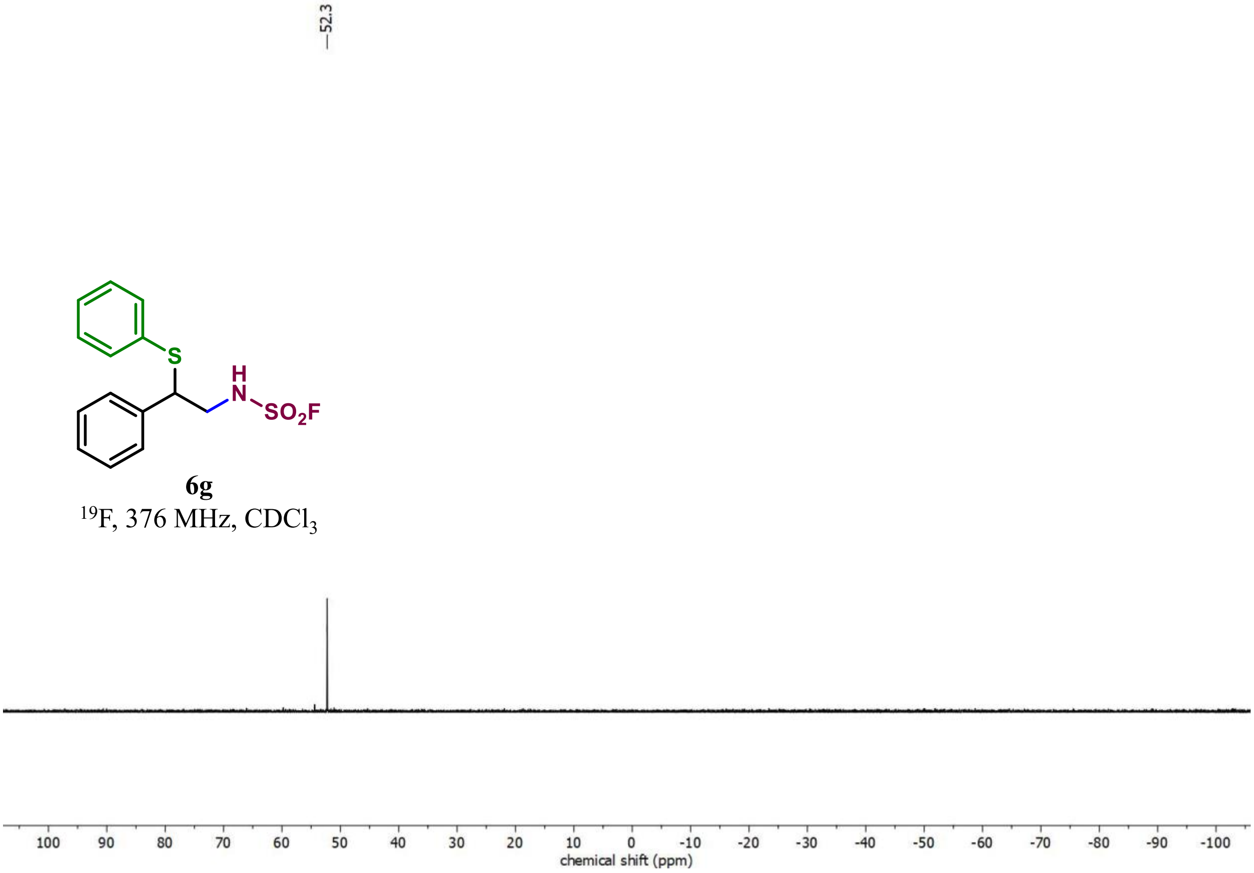

6g

^{19}F, 376 MHz, $CDCl_3$

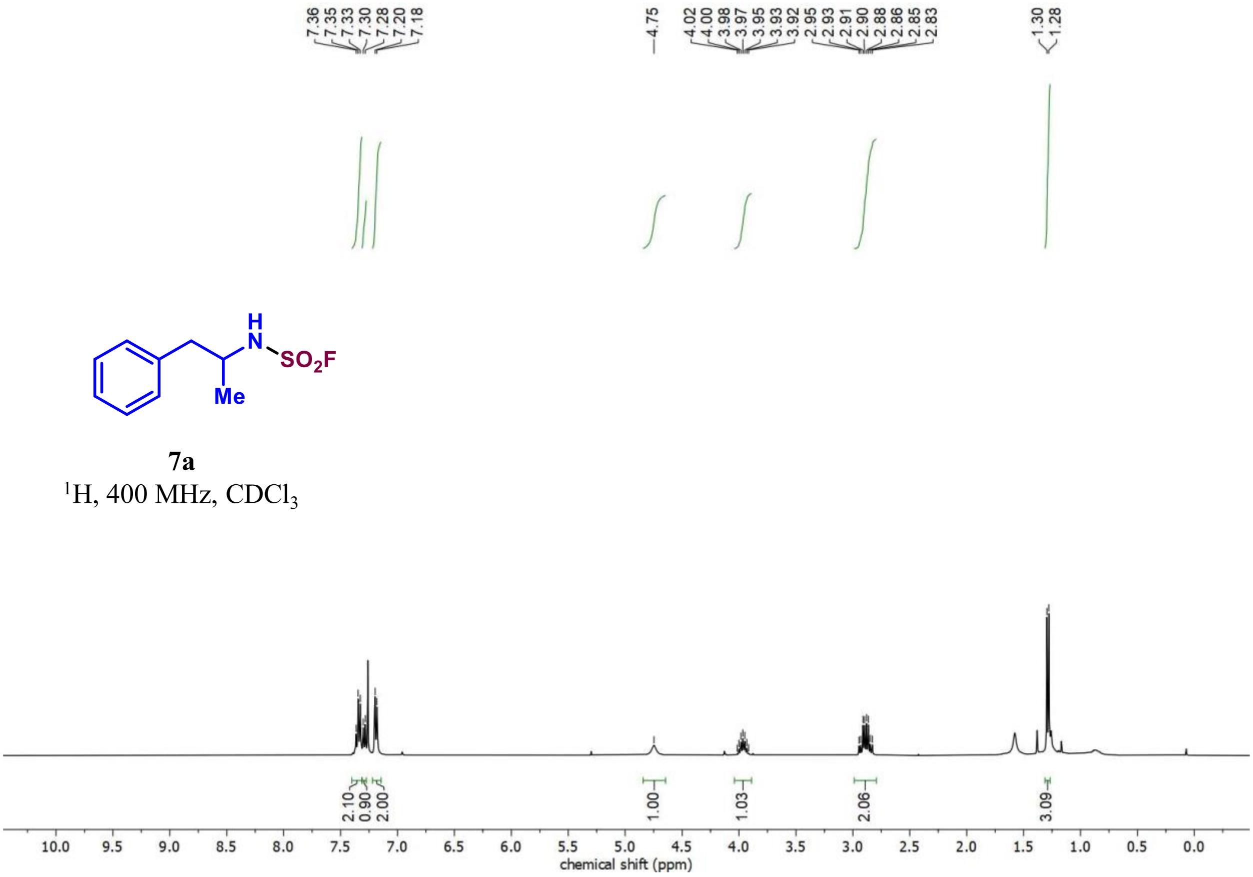

7a
^{1}H, 400 MHz, $CDCl_3$

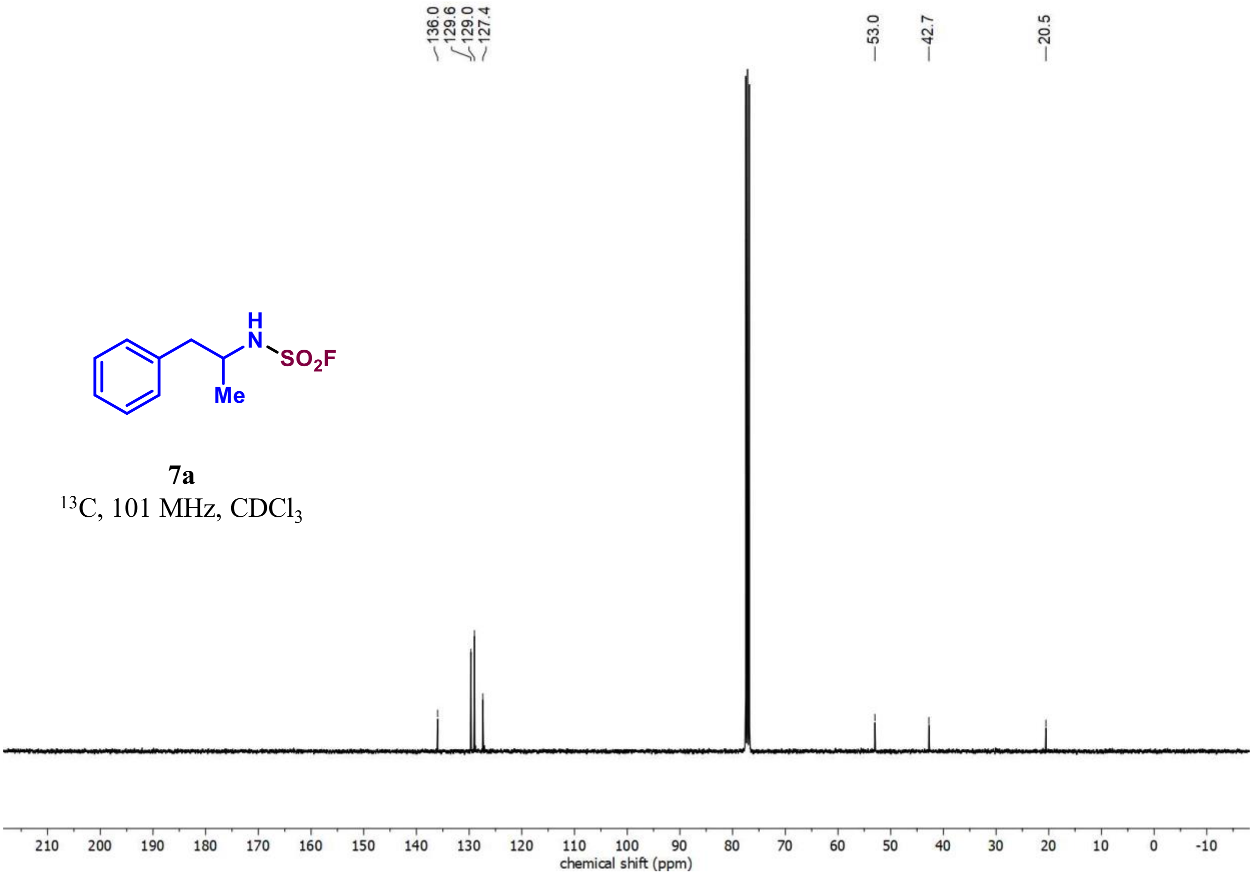

7a
^{13}C, 101 MHz, $CDCl_3$

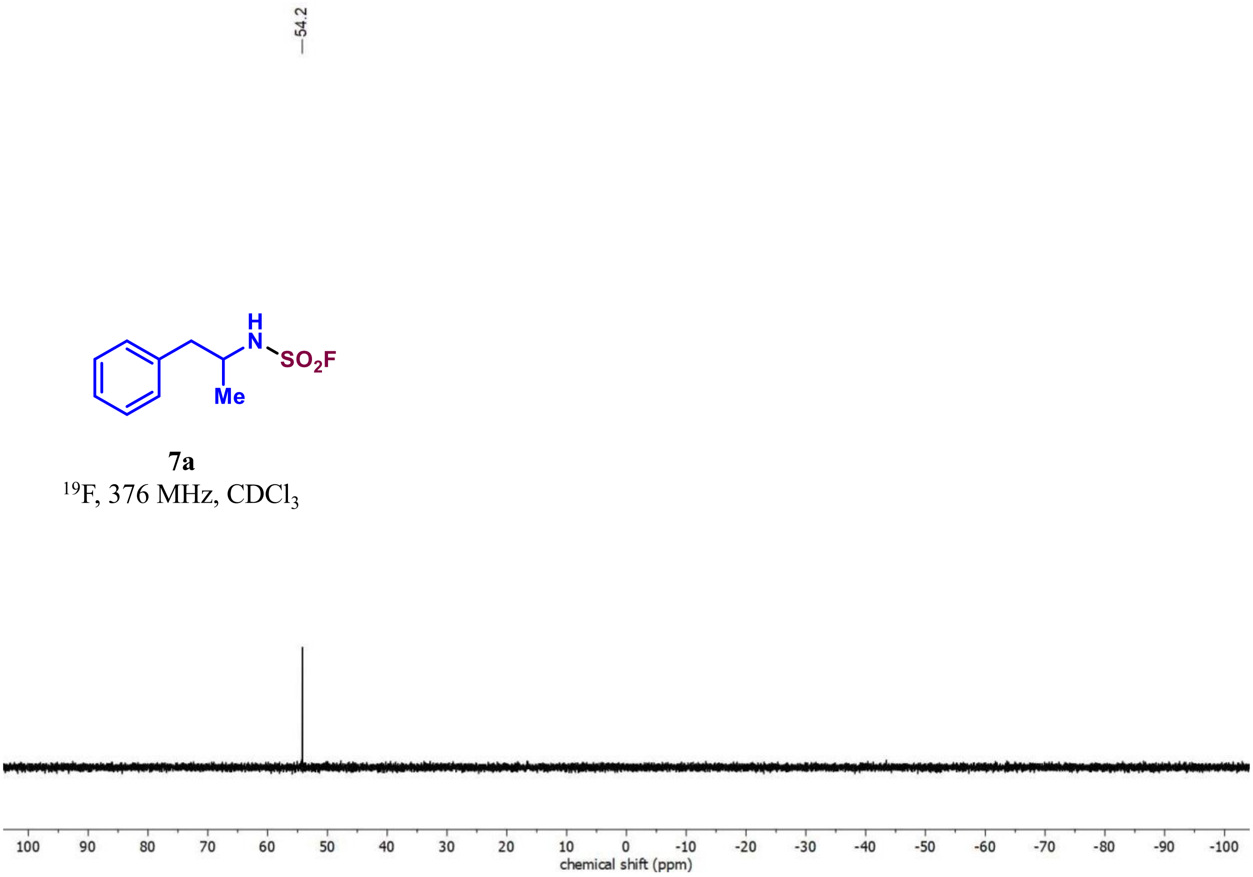

7a

^{19}F, 376 MHz, CDCl$_3$

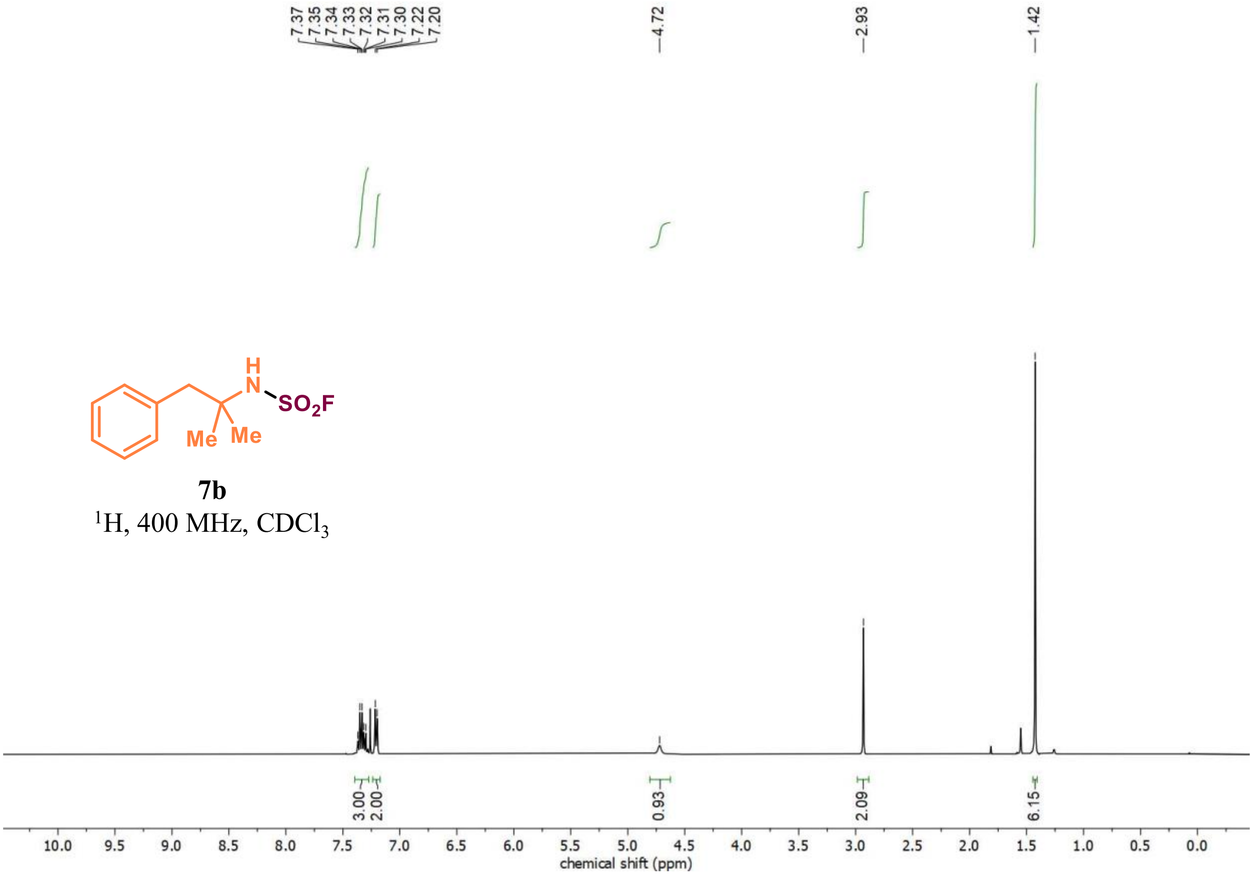

7b

^{1}H, 400 MHz, CDCl$_3$

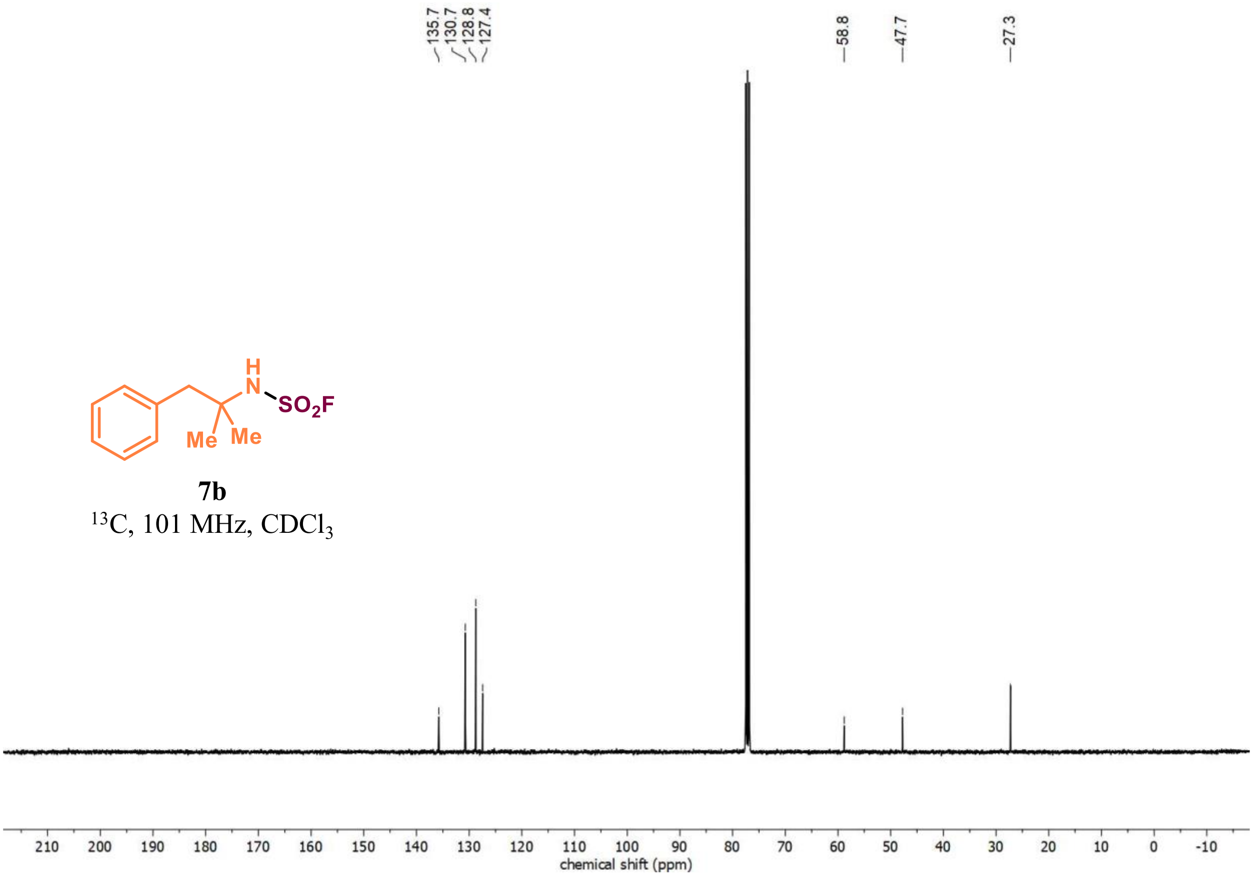

7b

^{13}C, 101 MHz, $CDCl_3$

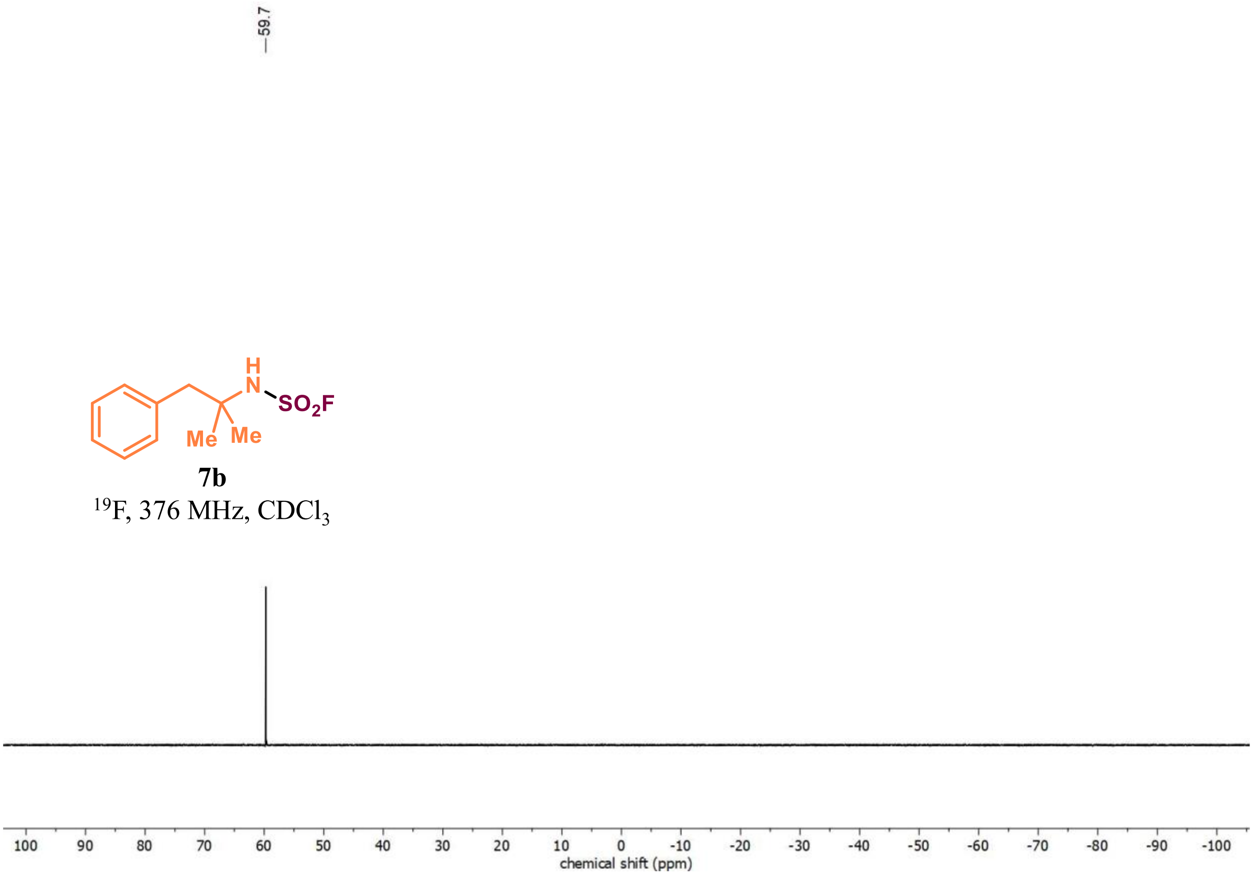

7b

^{19}F, 376 MHz, $CDCl_3$

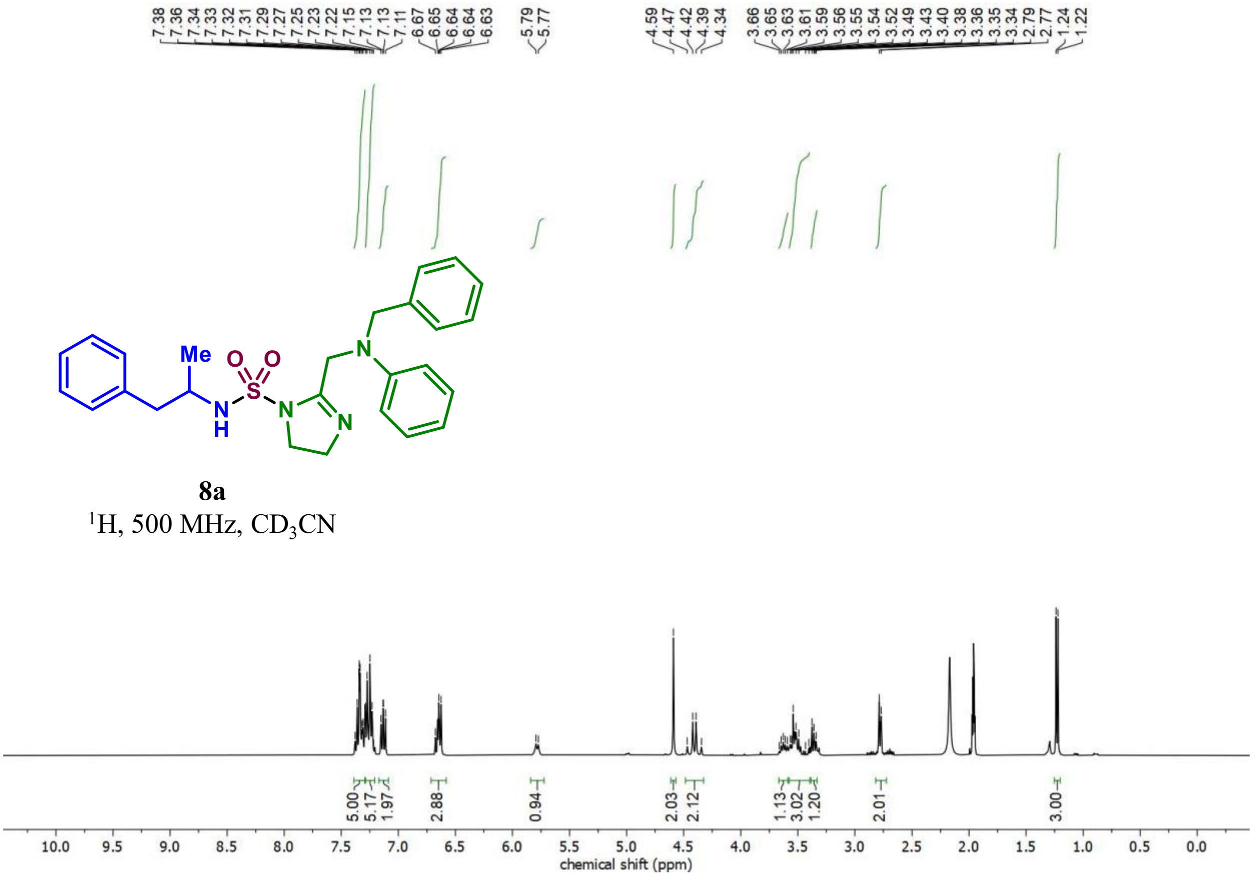

8a

^{1}H, 500 MHz, CD_3CN

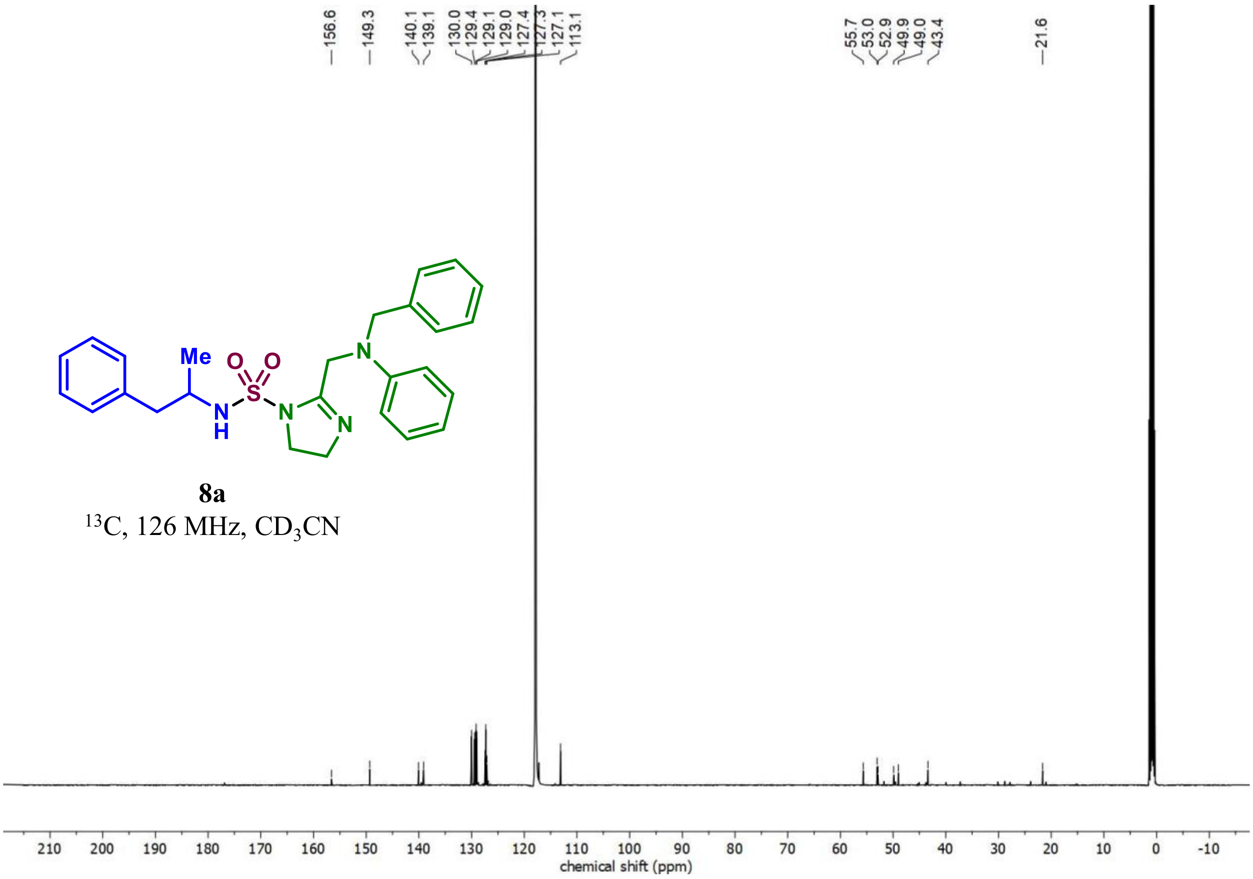

8a

^{13}C, 126 MHz, CD_3CN

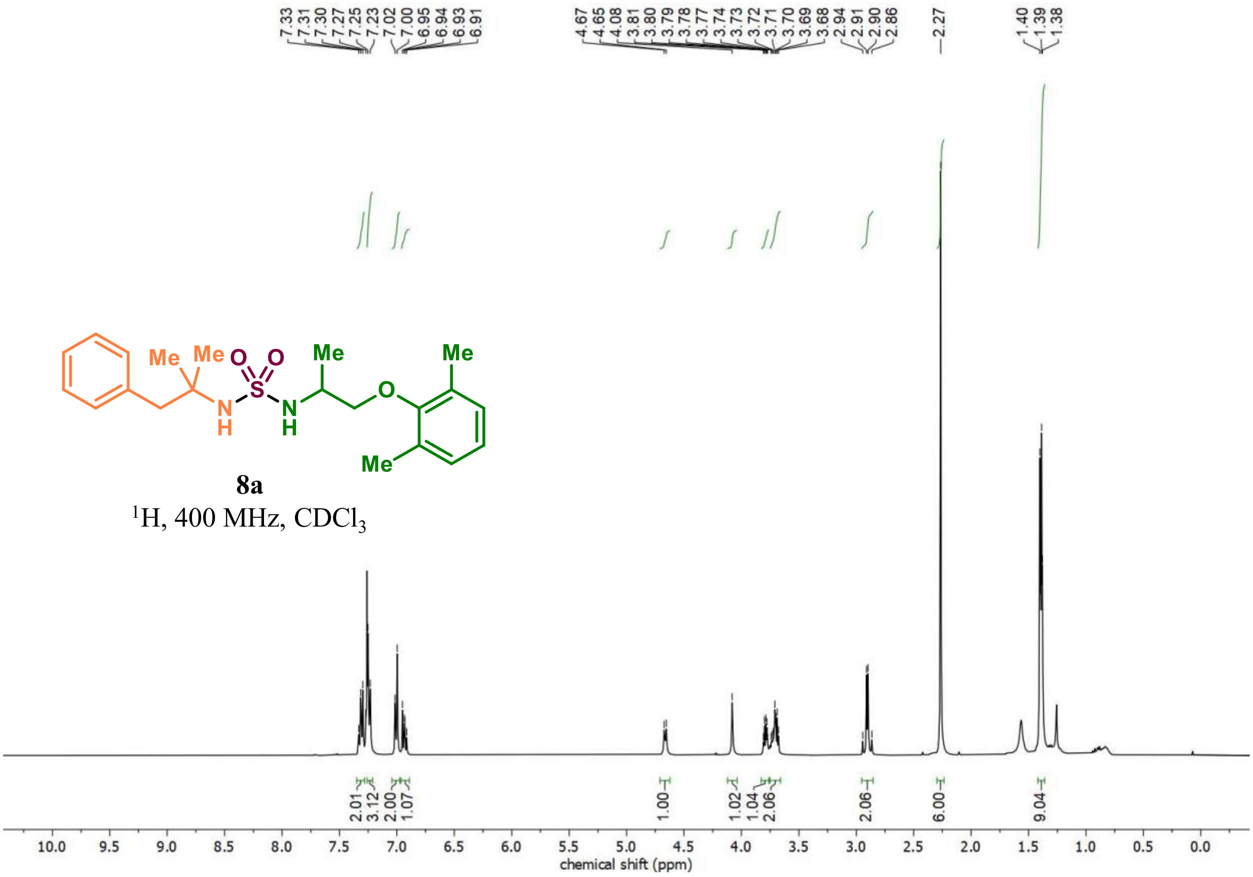

Me
Me
O
O
S
N
H
N
H
Me
O
Me
Me
8a
^{1}H, 400 MHz, $CDCl_3$
chemical shift (ppm)

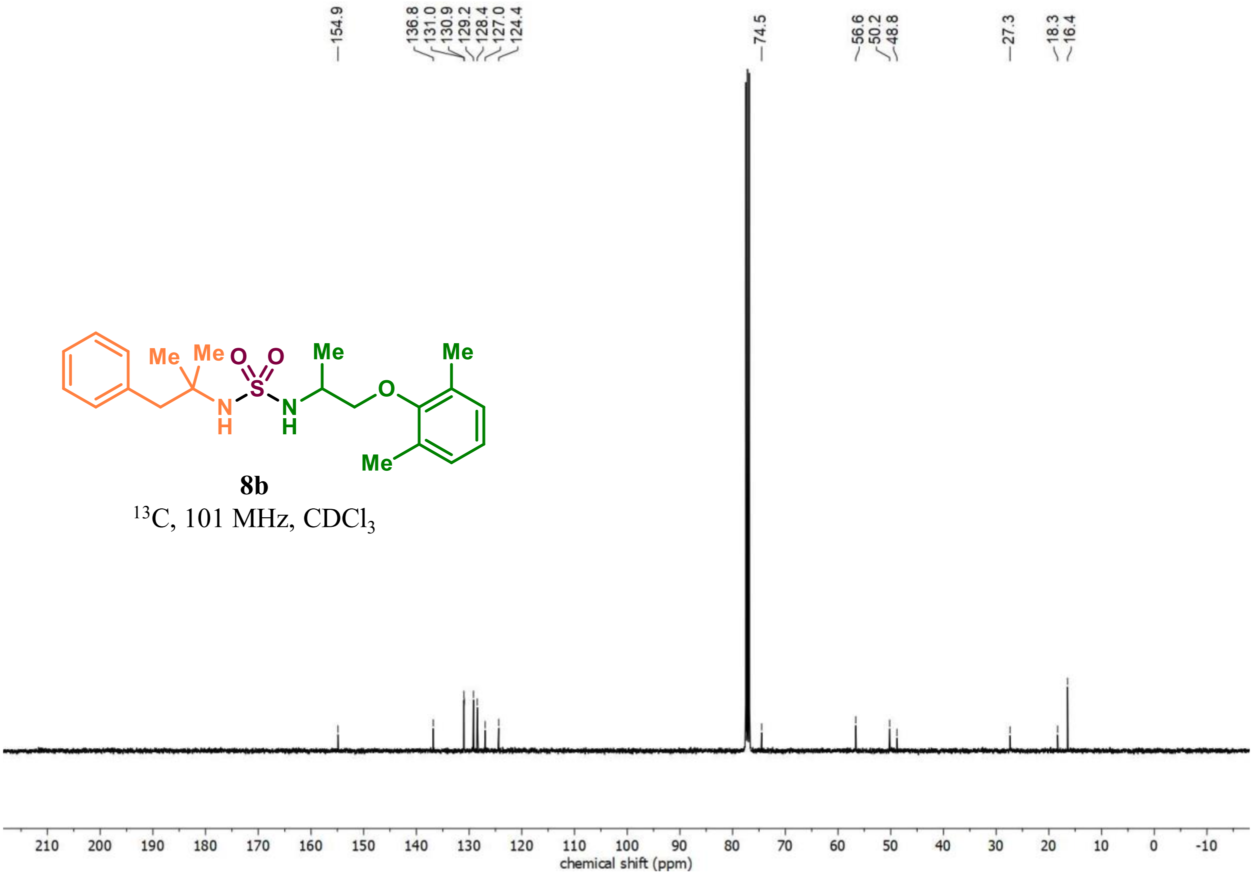

Me
Me
O
O
S
N
H
N
H
Me
O
Me
Me
8b
^{13}C, 101 MHz, $CDCl_3$
chemical shift (ppm)

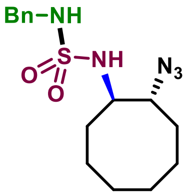

8c

^{1}H, 400 MHz, $CDCl_3$

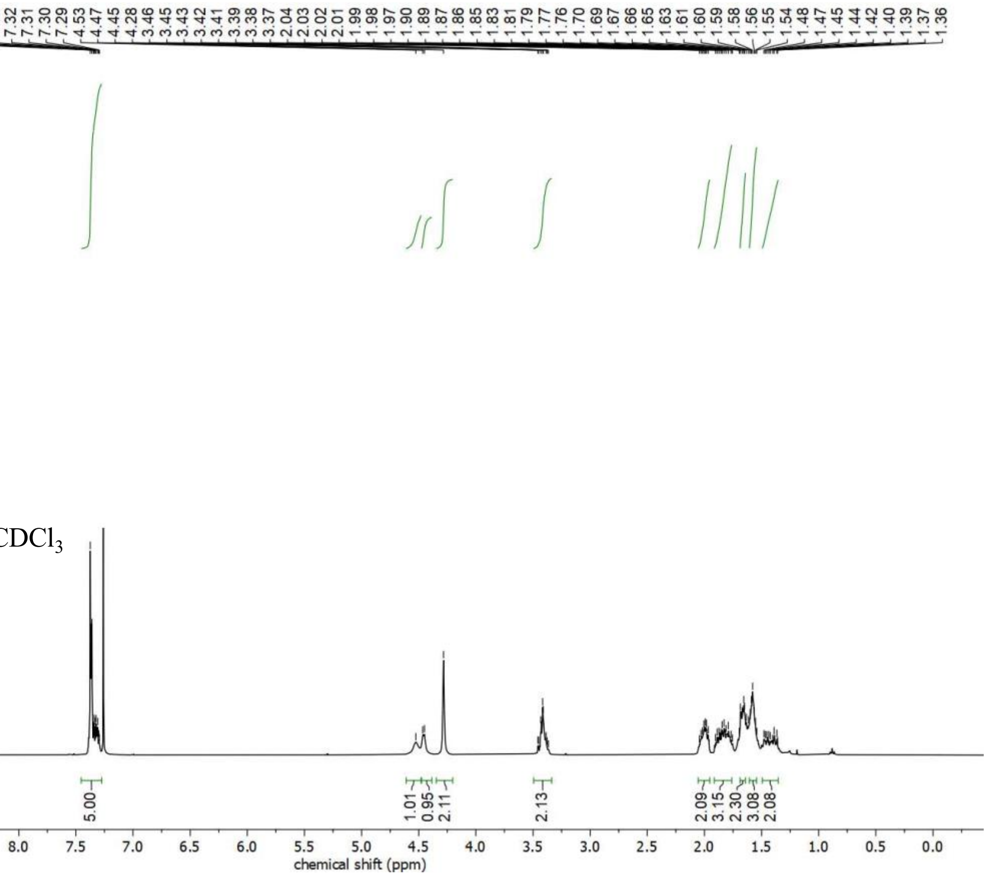

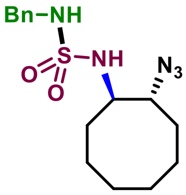

8c

^{13}C, 101 MHz, $CDCl_3$

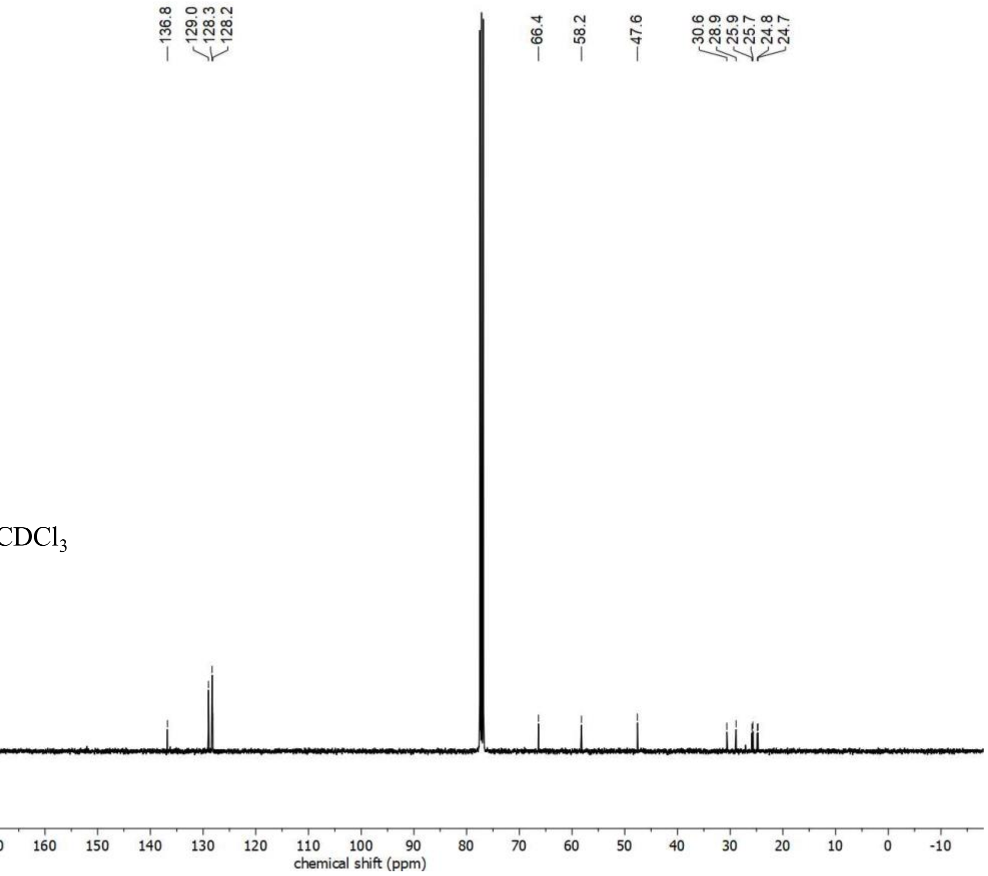

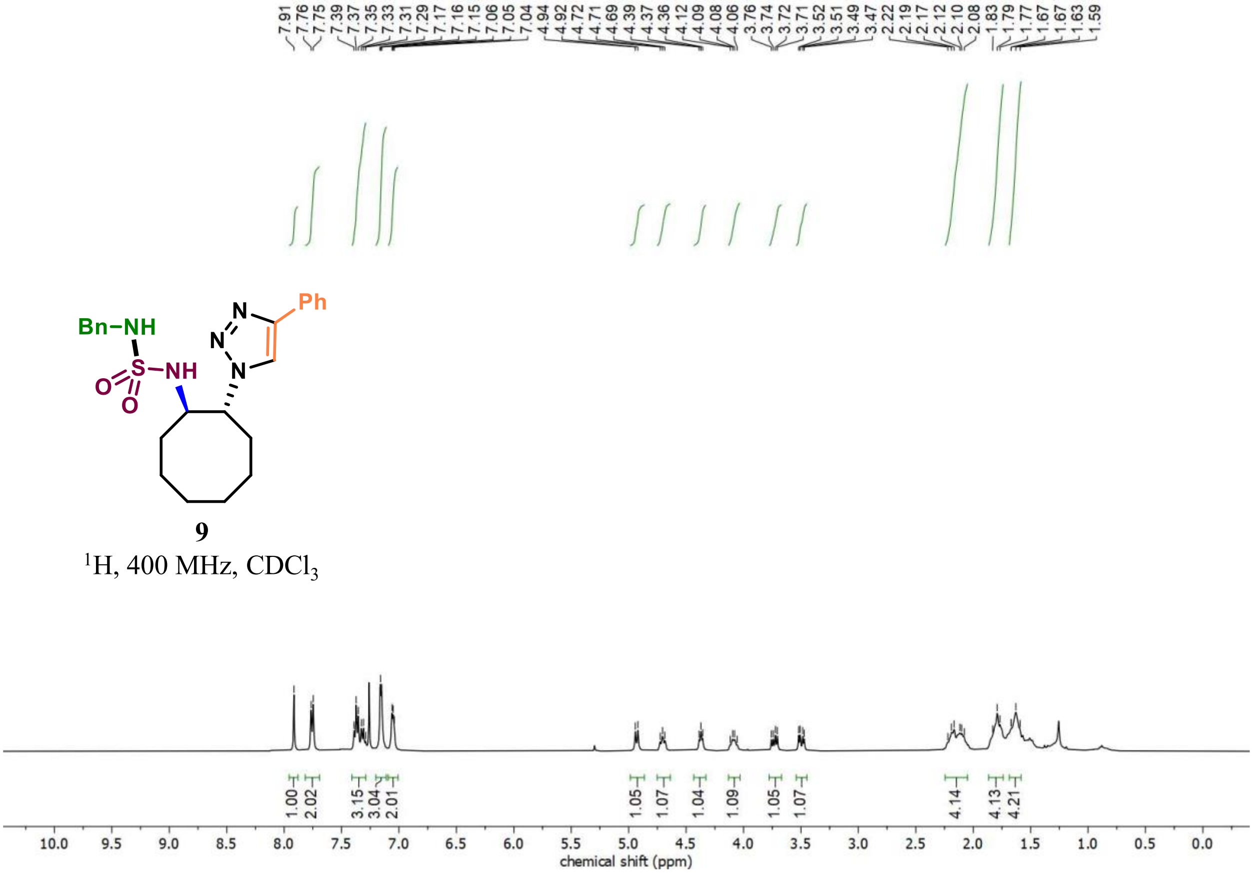

9

^{1}H, 400 MHz, $CDCl_3$

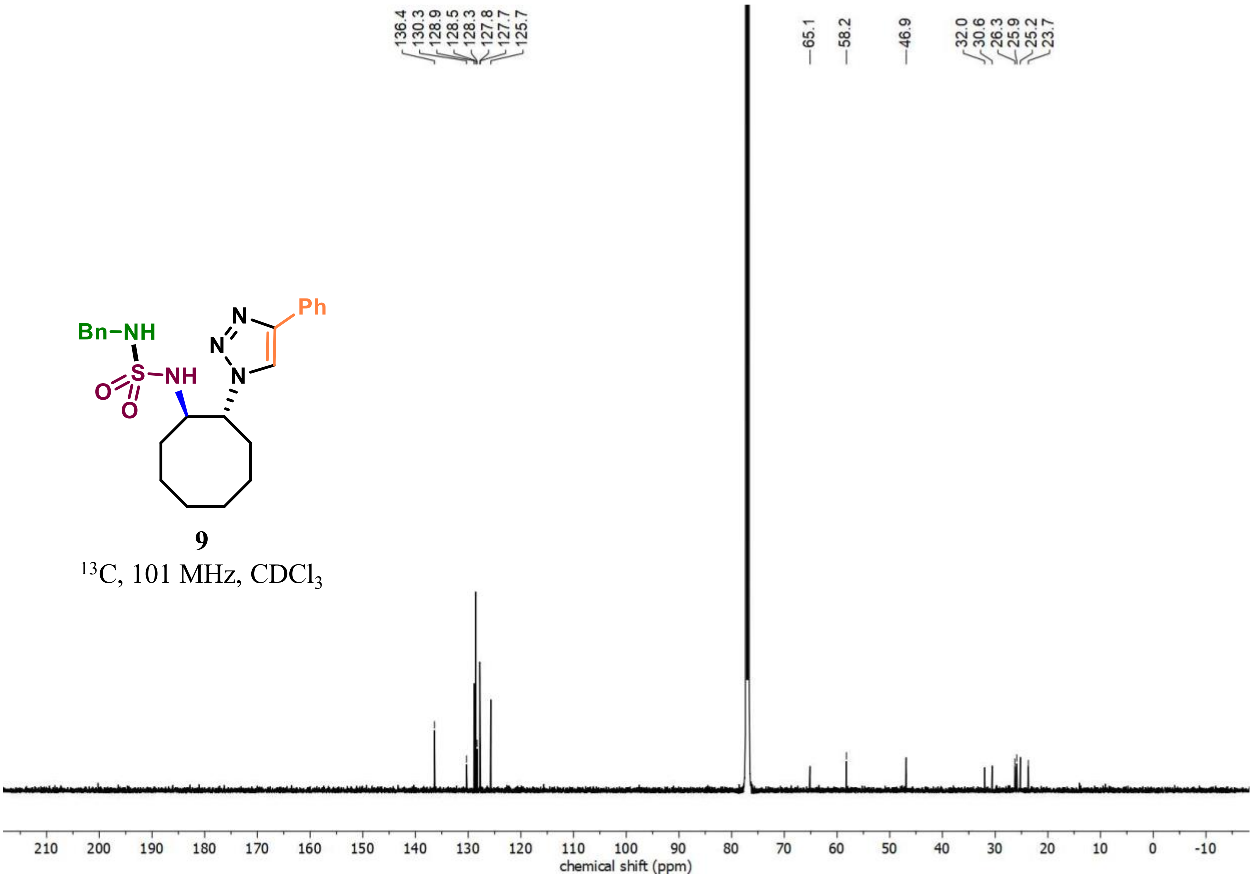

9

^{13}C, 101 MHz, $CDCl_3$

References

[1] H. Yang; R. G. Carter, *Org. Lett.* **2010**, *12*, 3108–3111.
[2] X. Li, S. He, Q. Song, *Org. Lett.* **2021**, *23*, 2994–2999.
[3] T. Mies, A. J. P. White, P. J. Parsons, A. G. M. Barrett, *J. Org. Chem.* **2021**, *86*, 1802–1817.
[4] T. Guo, G. Meng; X. Zhan, Q. Yang, T. Ma; L. Xu, K. B. Sharpless, J. Dong, *Angew. Chem., Int. Ed.* **2018**, *57*, 2605–2610.
[5] Y. Liu, H. Wu, Y. Guo, J. C. Xiao, Q. Y. Chen, C. Liu, *Angew. Chem., Int. Ed.* **2017**, *56*, 15432–15435.
[6] G. Meng,T. Guo, T. Ma, J. Zhang; Y. Shen, K. B. Sharpless, J. Dong, *Nature* **2019**, *574*, 86–89.
[7] V. Boquet, A. Nasrallah, A. L. Dana, E. Brunard, P. H. Di Chenna, F. J. Duran, P. Retailleau, B. Darses, M. Sircoglou, P. Dauban, *J. Am. Chem. Soc.* **2022**, *144*, 17156–17164.
[8] K. Guthikonda, J. Du Bois, *J. Am. Chem. Soc.* **2002**, *124*, 13672–13673.
